# Supplementary material for: Evolution of DNA Methylation Across Ecdysozoa
Source: J Mol Evol. 2022 Jan 28;90(1):56–72. doi: 10.1007/s00239-021-10042-0 (PMC8821070; doi:10.1007/s00239-021-10042-0)
Supplement: Supplementary file 1 — Additional file 1 (DOCX 36202 kb) [file 239_2021_10042_MOESM1_ESM.pdf]

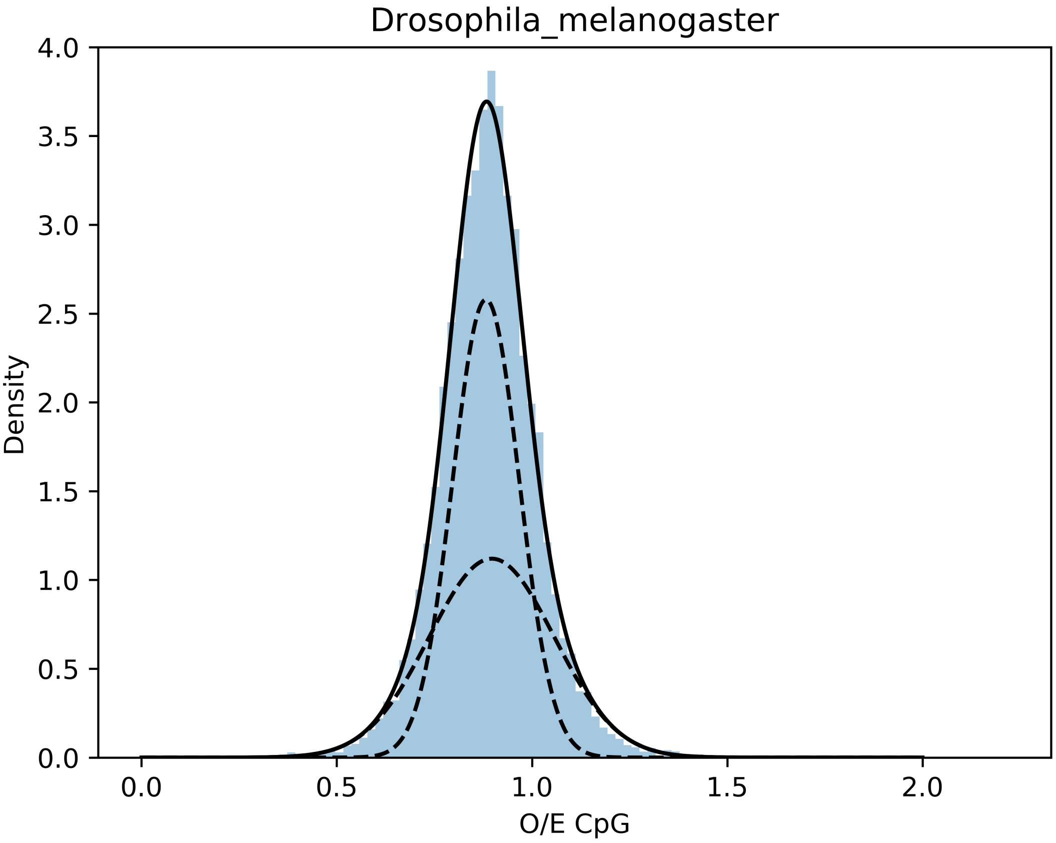
Drosophila_melanogaster
Density
O/E CpG
0.0
0.5
1.0
1.5
2.0
2.5
3.0
3.5
4.0
0.0
0.5
1.0
1.5
2.0

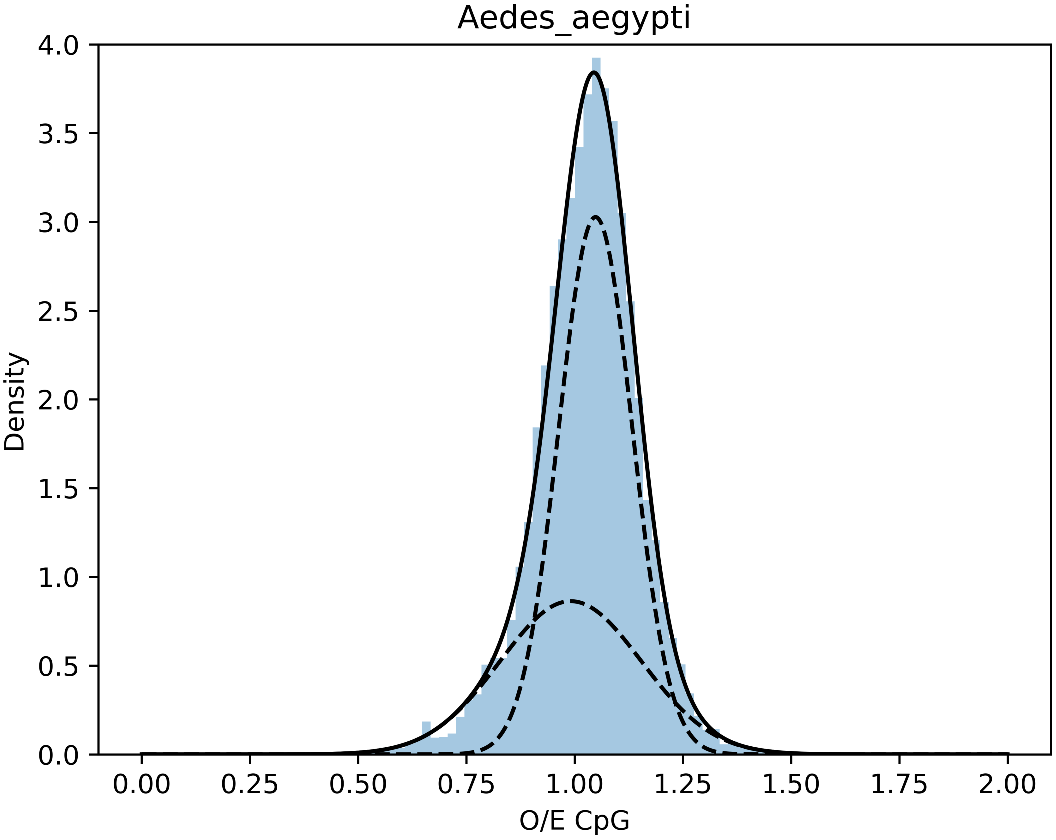
Aedes_aegypti
Density
O/E CpG
4.0
3.5
3.0
2.5
2.0
1.5
1.0
0.5
0.0
0.00
0.25
0.50
0.75
1.00
1.25
1.50
1.75
2.00

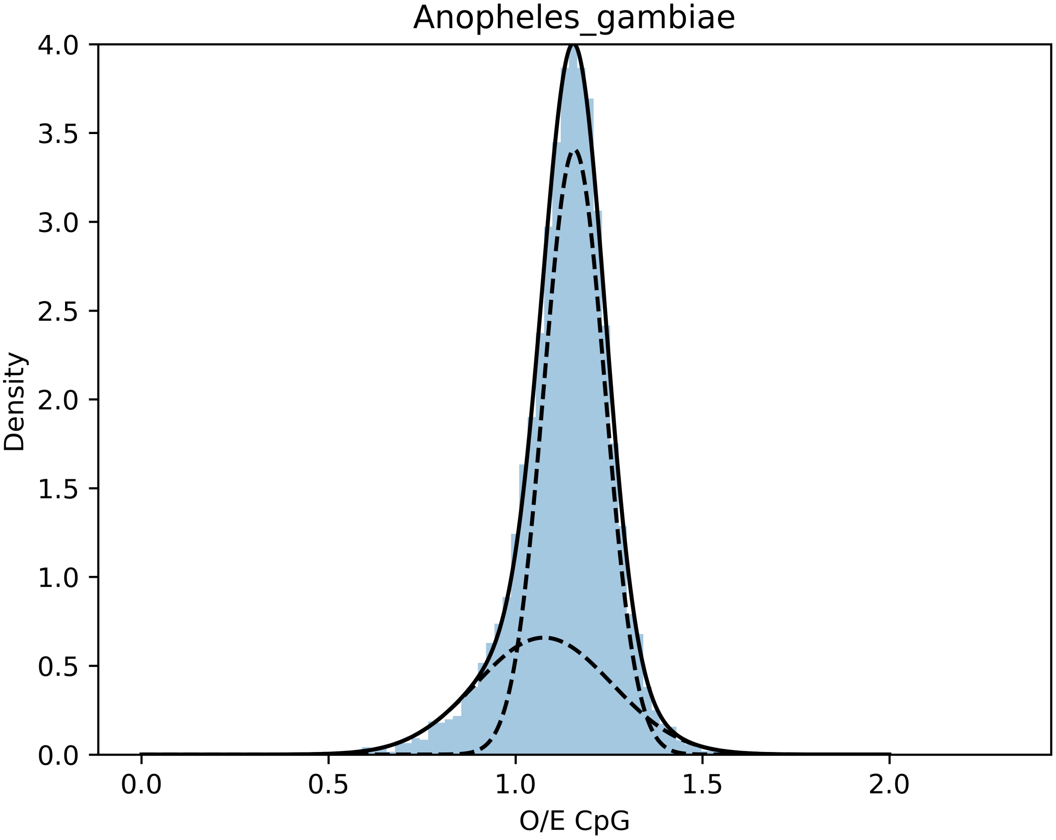
Anopheles_gambiae
Density
O/E CpG
0.0
0.5
1.0
1.5
2.0
2.5
3.0
3.5
4.0
0.0
0.5
1.0
1.5
2.0

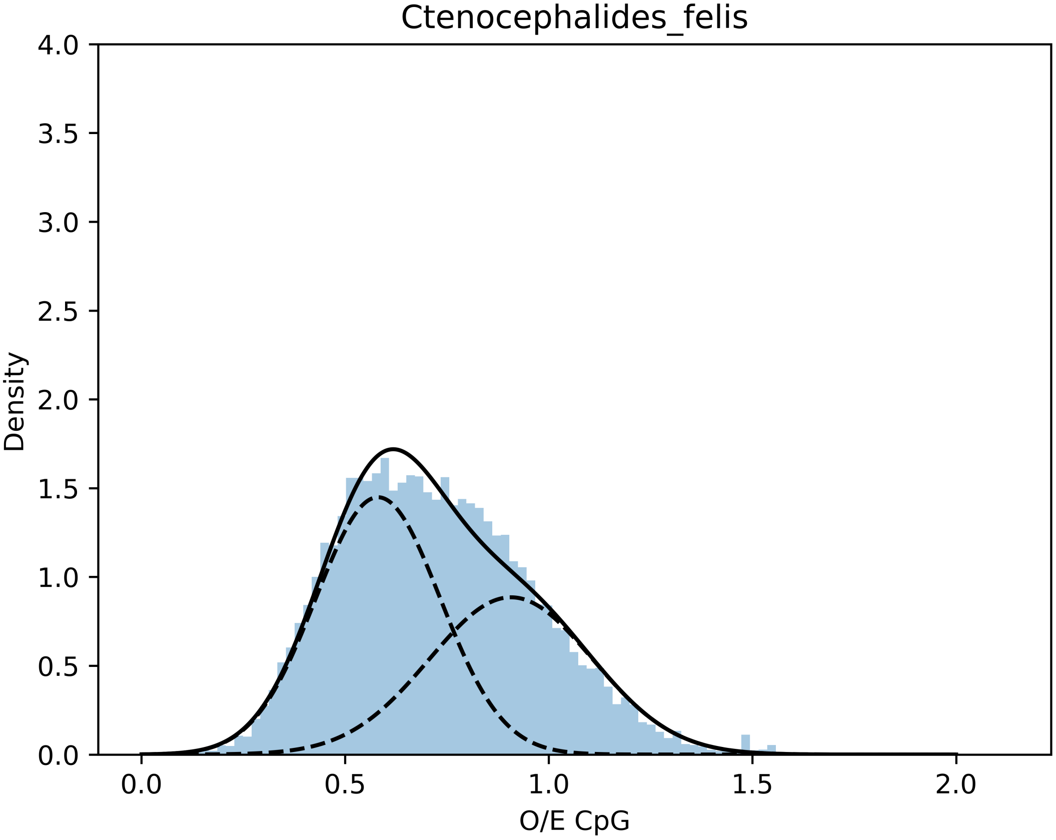

Ctenocephalides_felis
Density
O/E CpG
4.0
3.5
3.0
2.5
2.0
1.5
1.0
0.5
0.0
0.0
0.5
1.0
1.5
2.0

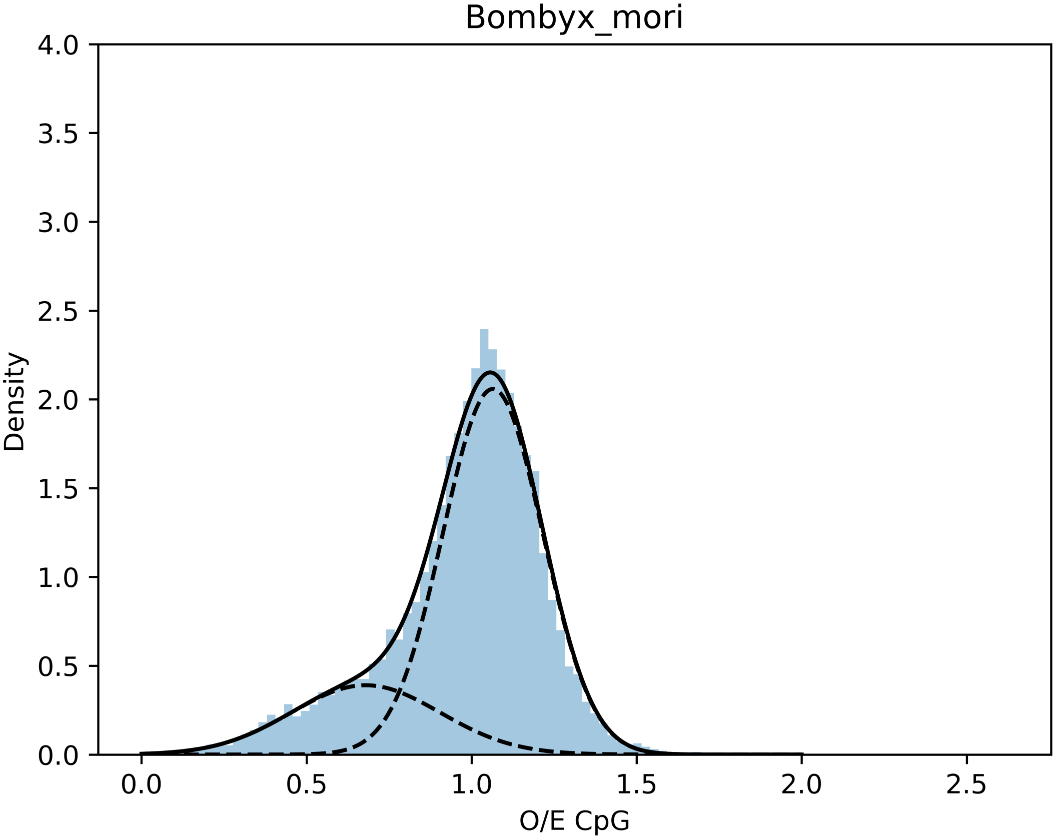
Bombyx_mori
Density
O/E CpG
0.0
0.5
1.0
1.5
2.0
2.5
3.0
3.5
4.0
0.0
0.5
1.0
1.5
2.0
2.5

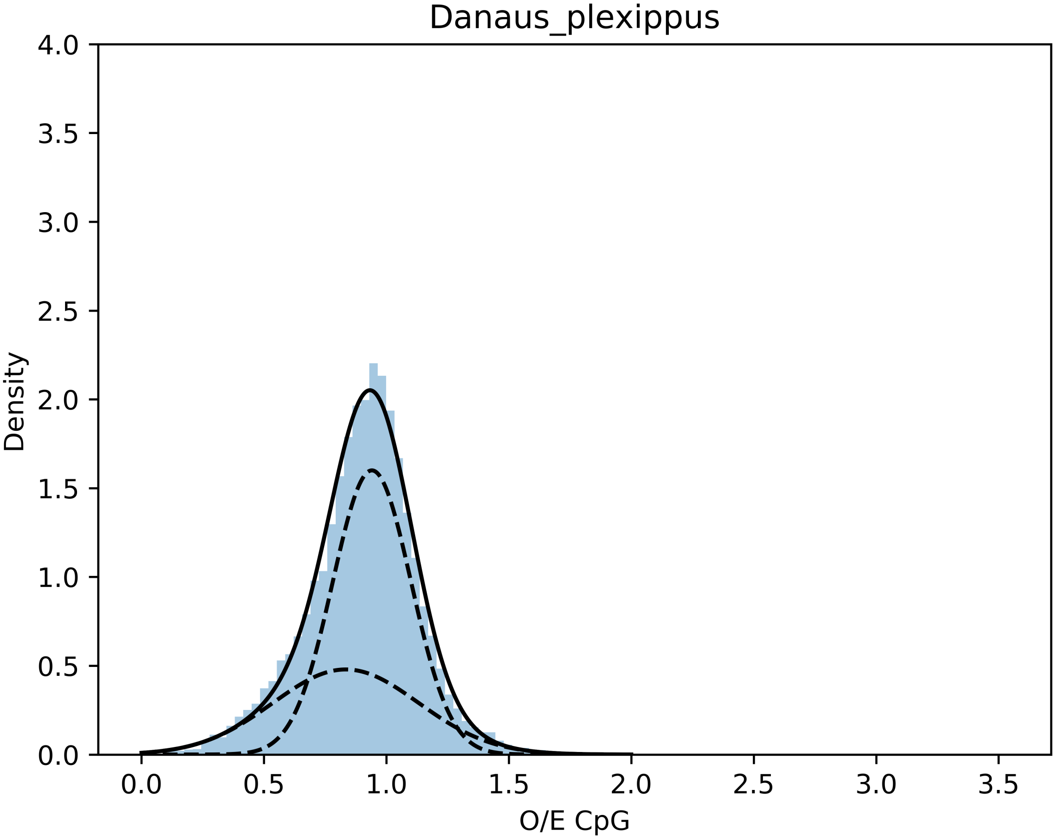

Danaus_plexippus
Density
O/E CpG
0.0
0.5
1.0
1.5
2.0
2.5
3.0
3.5
4.0

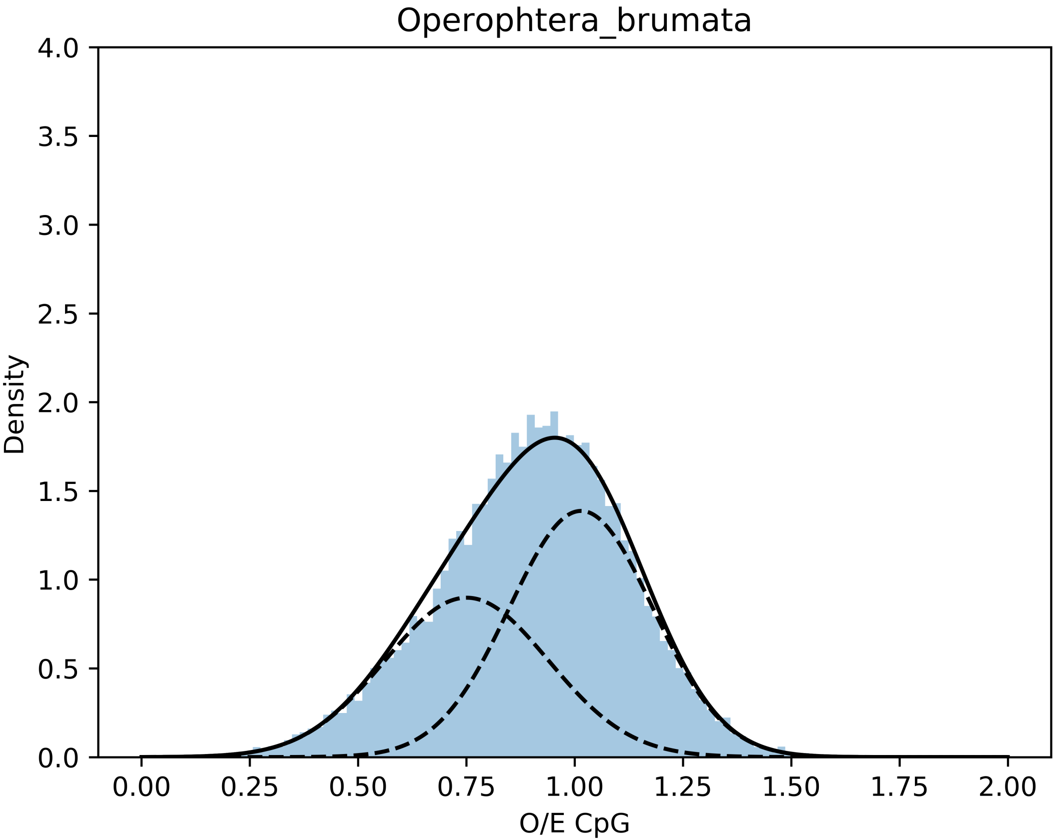
Operophtera_brumata
Density
O/E CpG
0.0
0.5
1.0
1.5
2.0
2.5
3.0
3.5
4.0
0.00
0.25
0.50
0.75
1.00
1.25
1.50
1.75
2.00

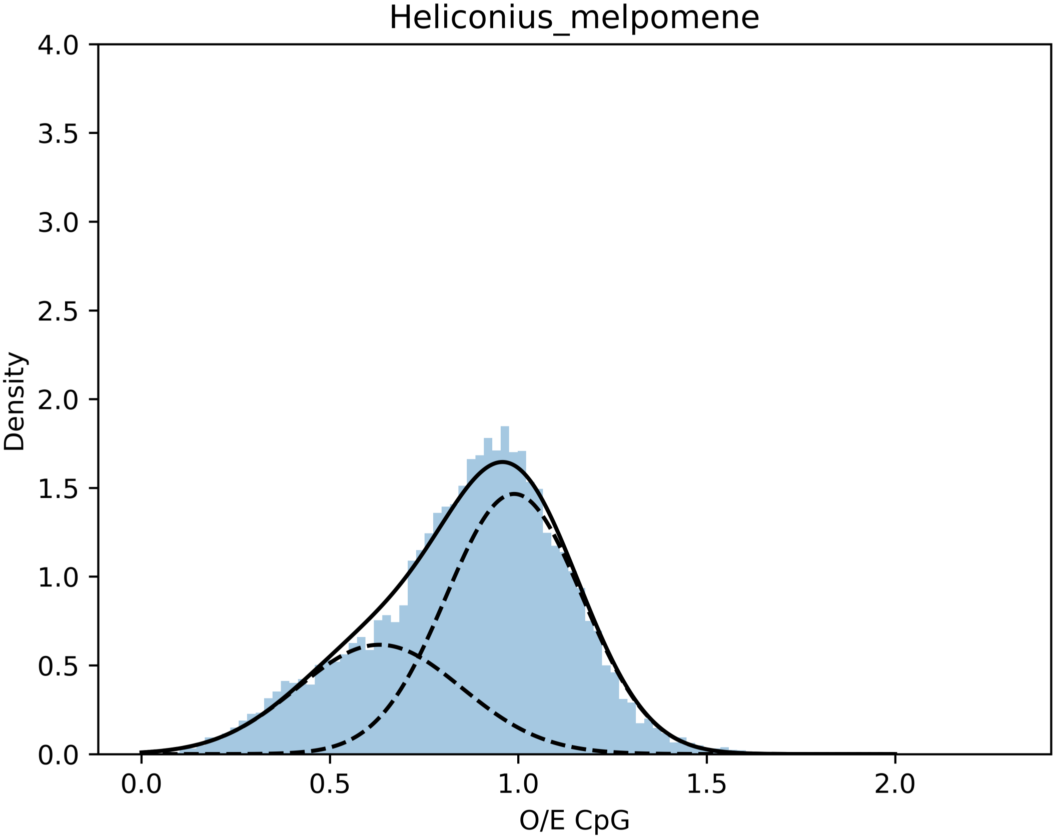
Heliconius_melpomene
Density
O/E CpG
0.0
0.5
1.0
1.5
2.0
2.5
3.0
3.5
4.0
0.0
0.5
1.0
1.5
2.0

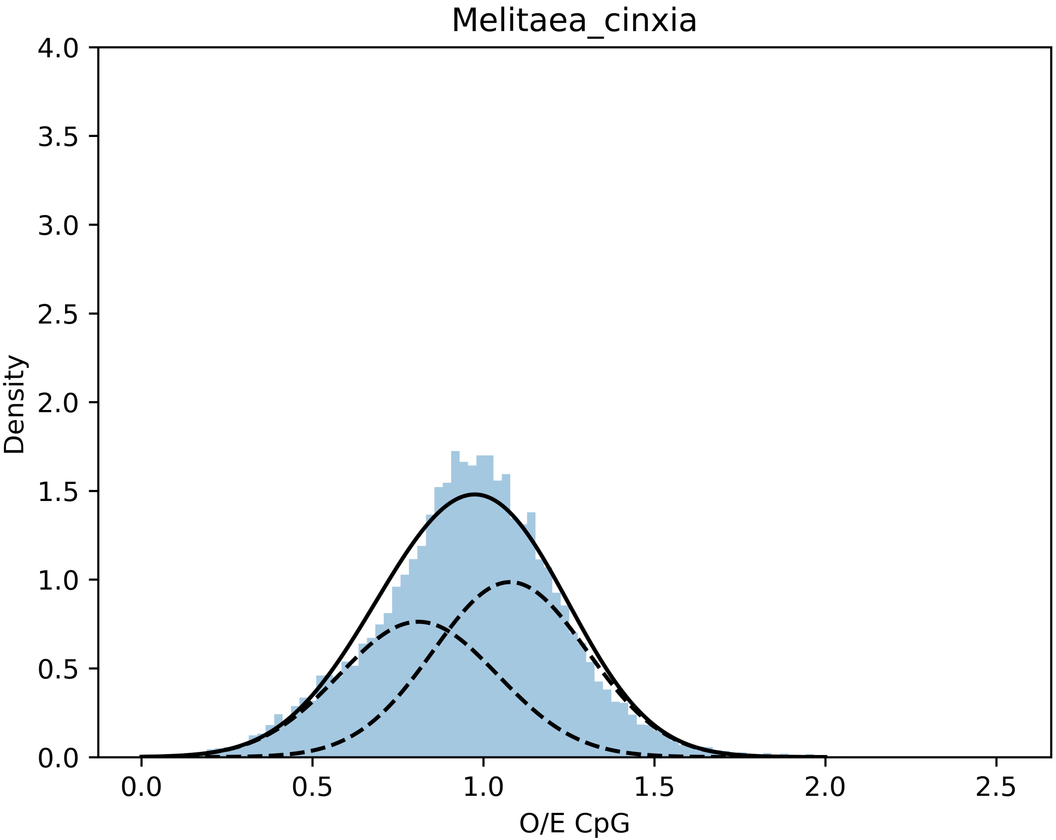
Melitaea_cinxia
Density
O/E CpG
0.0
0.5
1.0
1.5
2.0
2.5
3.0
3.5
4.0
0.0
0.5
1.0
1.5
2.0
2.5

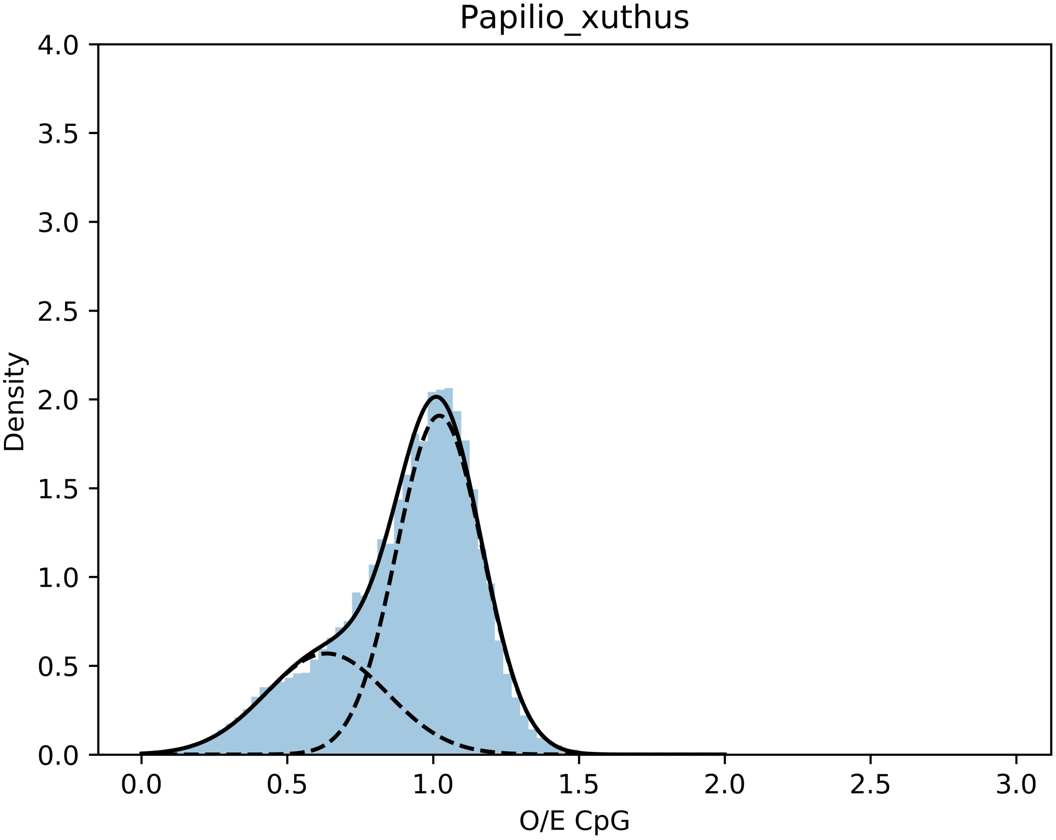

Papilio_xuthus
Density
O/E CpG
4.0
3.5
3.0
2.5
2.0
1.5
1.0
0.5
0.0
0.0
0.5
1.0
1.5
2.0
2.5
3.0

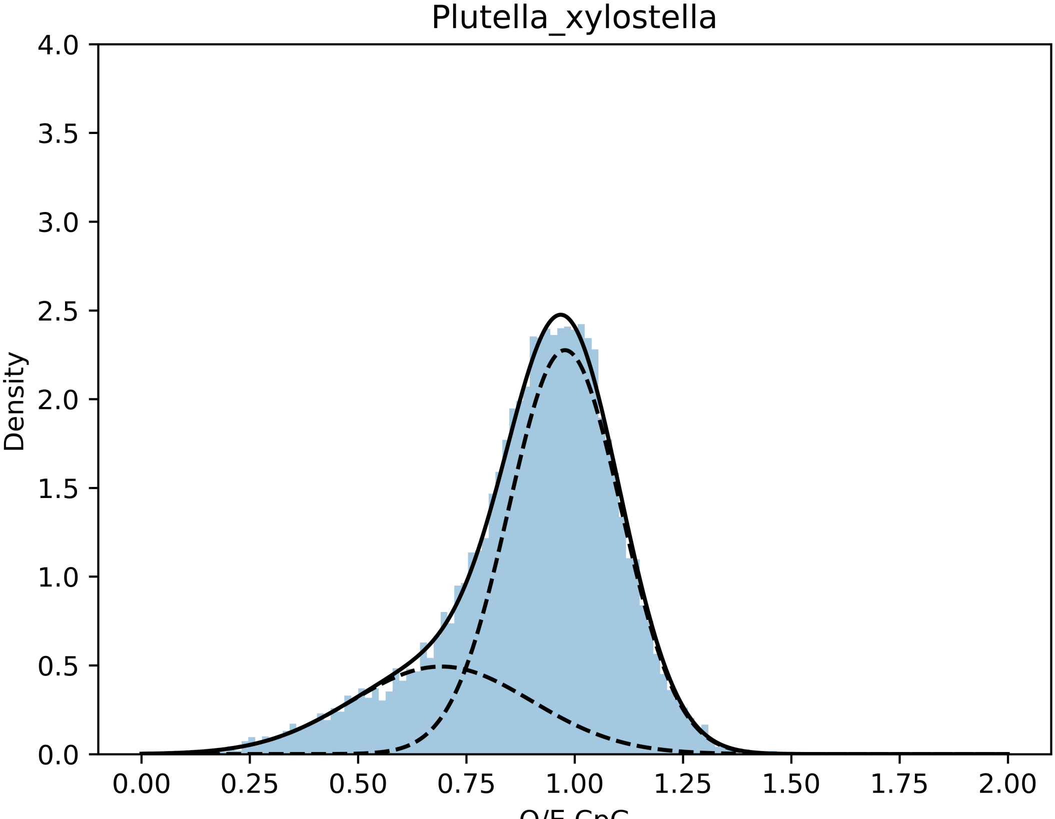

Plutella_xylostella
Density
O/E CpG
0.0
0.5
1.0
1.5
2.0
2.5
3.0
3.5
4.0
0.00
0.25
0.50
0.75
1.00
1.25
1.50
1.75
2.00

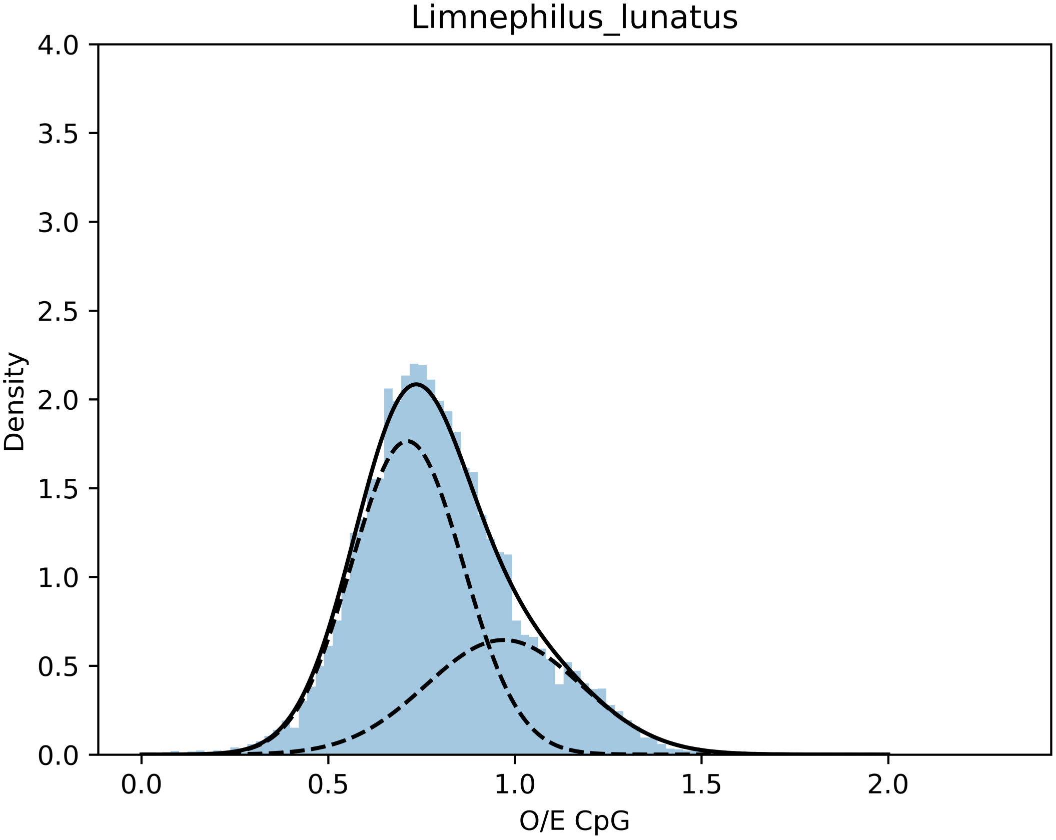

Limnephilus_lunatus
Density
O/E CpG
0.0
0.5
1.0
1.5
2.0
2.5
3.0
3.5
4.0

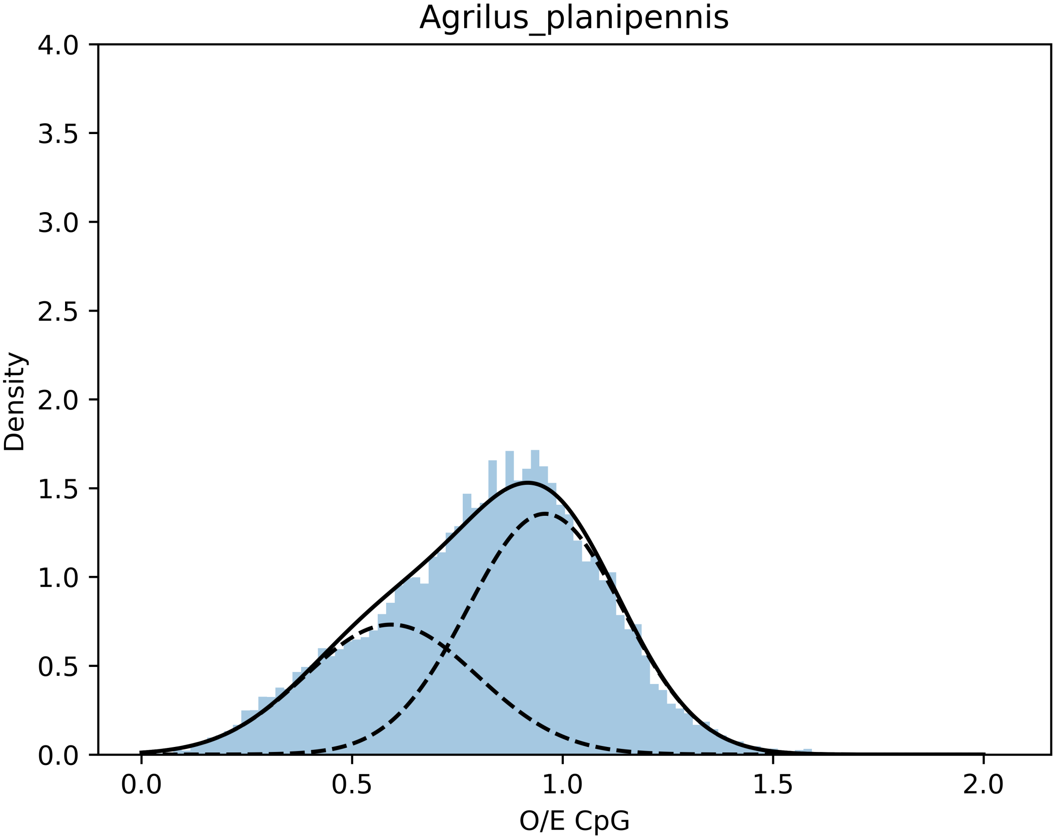

Agrilus_planipennis
Density
O/E CpG
4.0
3.5
3.0
2.5
2.0
1.5
1.0
0.5
0.0
0.0
0.5
1.0
1.5
2.0

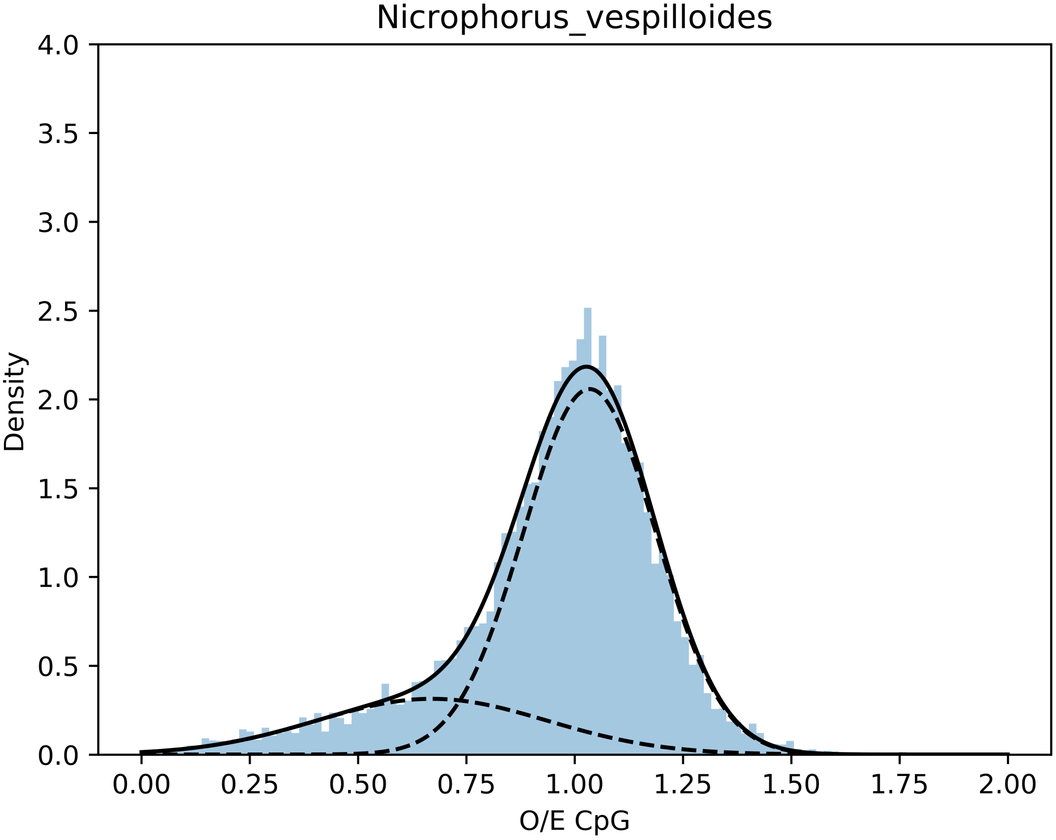

Nicrophorus_vespilloides
Density
O/E CpG
0.0
0.5
1.0
1.5
2.0
2.5
3.0
3.5
4.0
0.00
0.25
0.50
0.75
1.00
1.25
1.50
1.75
2.00

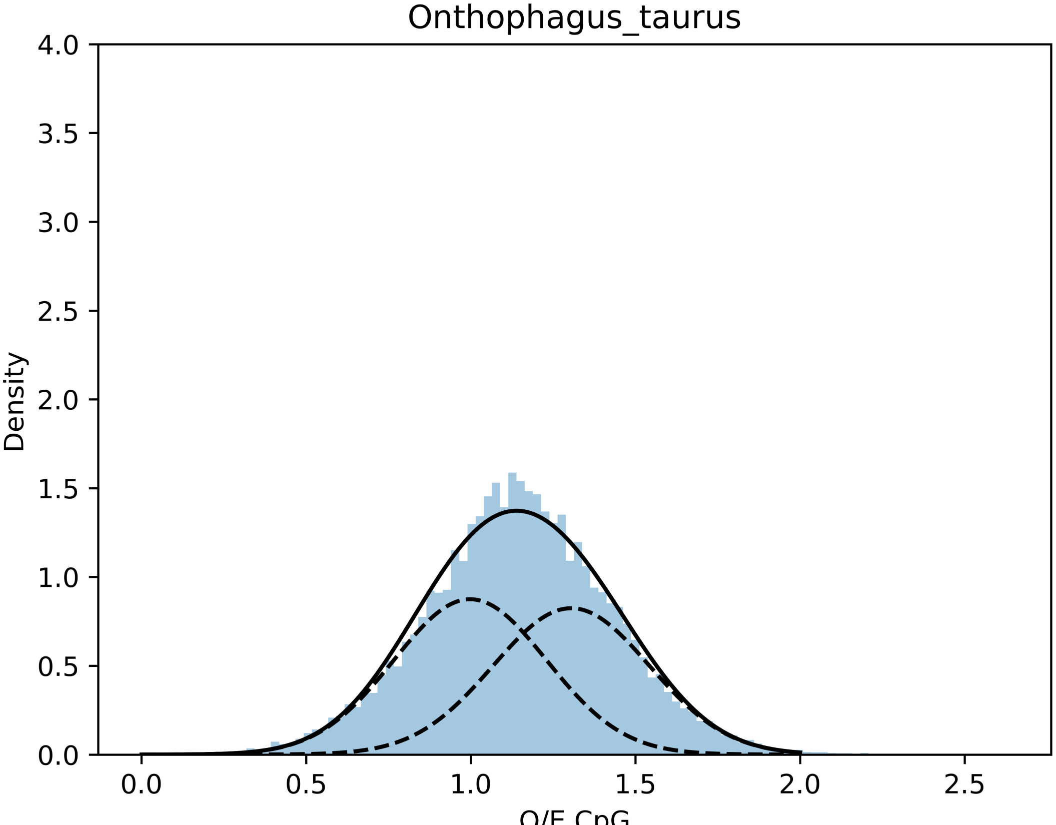
Onthophagus_taurus
Density
O/E CpG
0.0
0.5
1.0
1.5
2.0
2.5
3.0
3.5
4.0
0.0
0.5
1.0
1.5
2.0
2.5

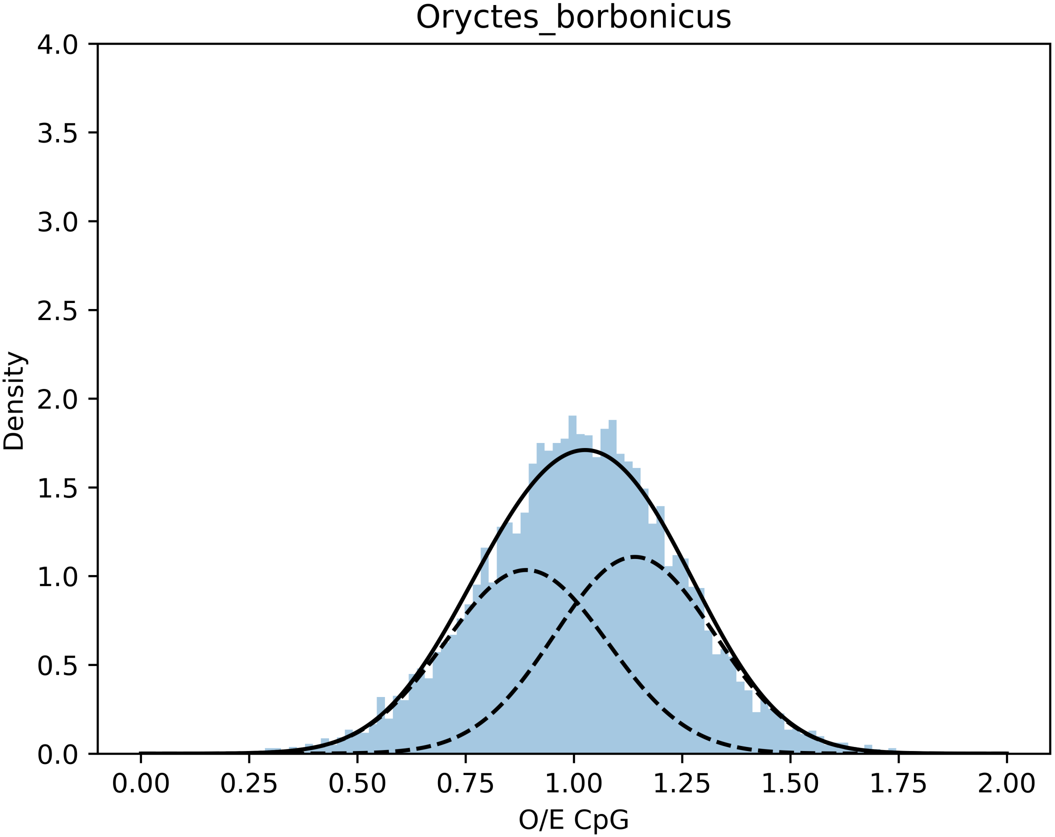
Oryctes_borbonicus
Density
O/E CpG
0.0
0.5
1.0
1.5
2.0
2.5
3.0
3.5
4.0
0.00
0.25
0.50
0.75
1.00
1.25
1.50
1.75
2.00

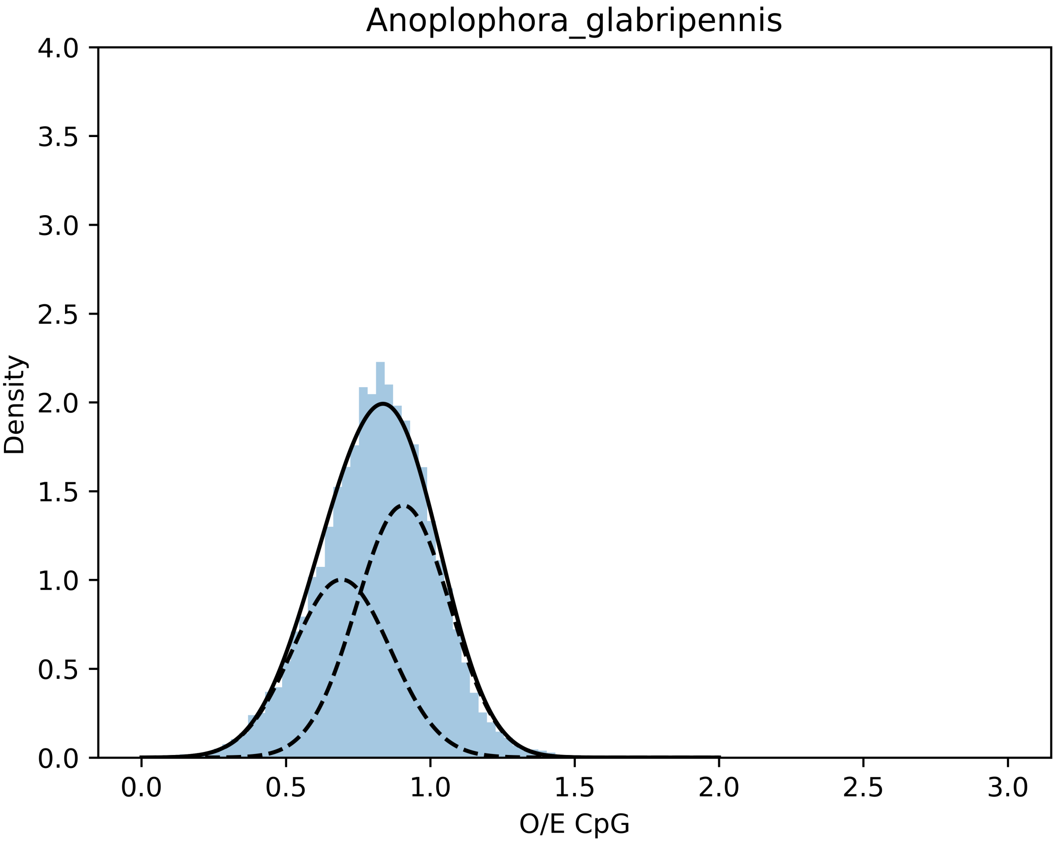
Anoplophora_glabripennis
Density
O/E CpG
0.0
0.5
1.0
1.5
2.0
2.5
3.0
3.5
4.0
0.0
0.5
1.0
1.5
2.0
2.5
3.0

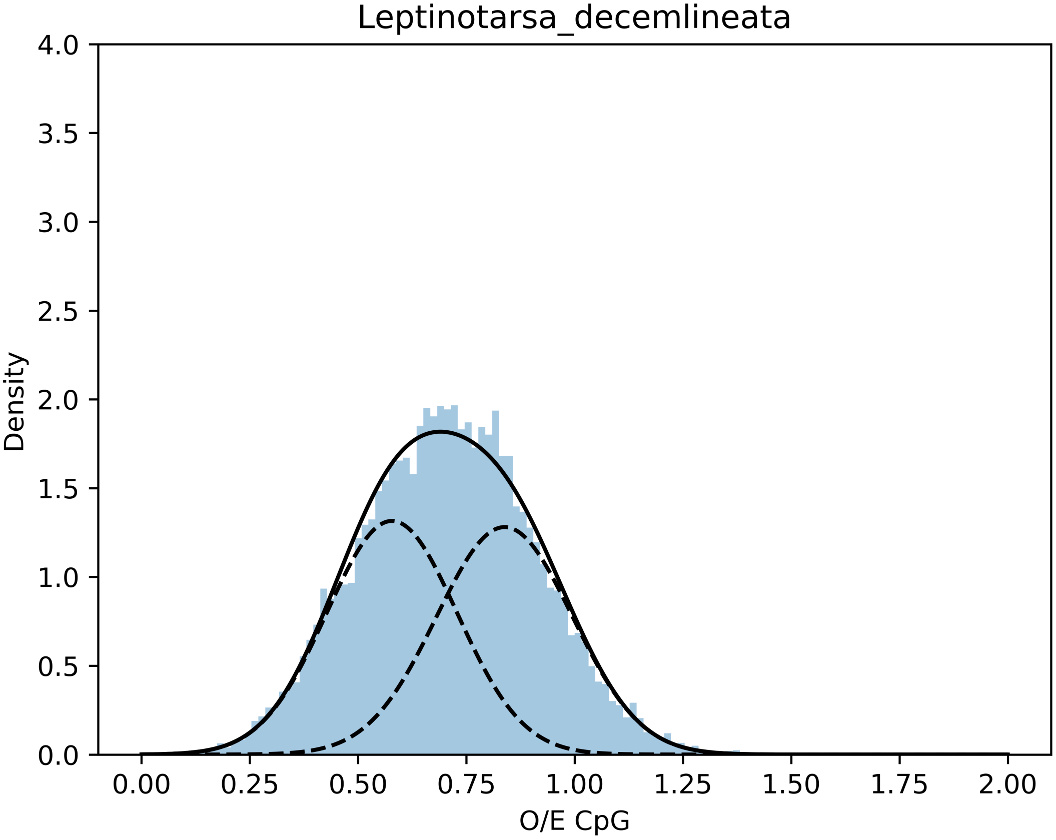
Leptinotarsa_decemlineata
Density
O/E CpG
0.0
0.5
1.0
1.5
2.0
2.5
3.0
3.5
4.0
0.00
0.25
0.50
0.75
1.00
1.25
1.50
1.75
2.00

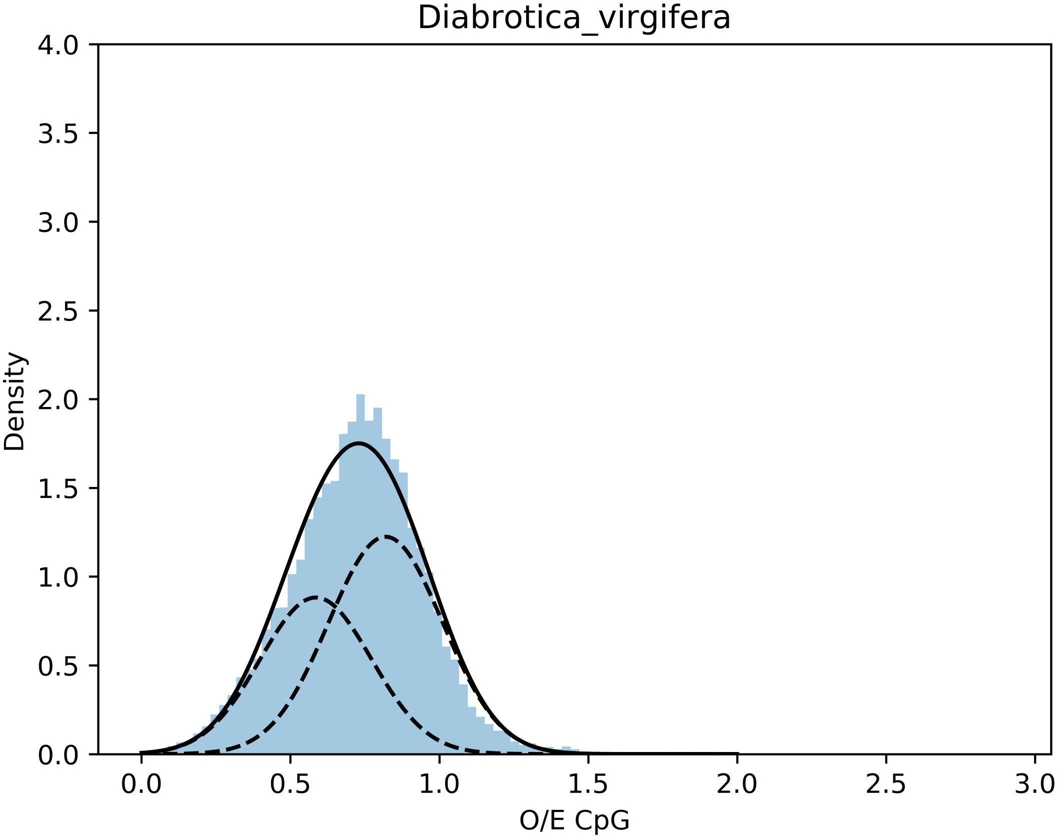
Diabrotica_virgifera
Density
O/E CpG
0.0
0.5
1.0
1.5
2.0
2.5
3.0
3.5
4.0

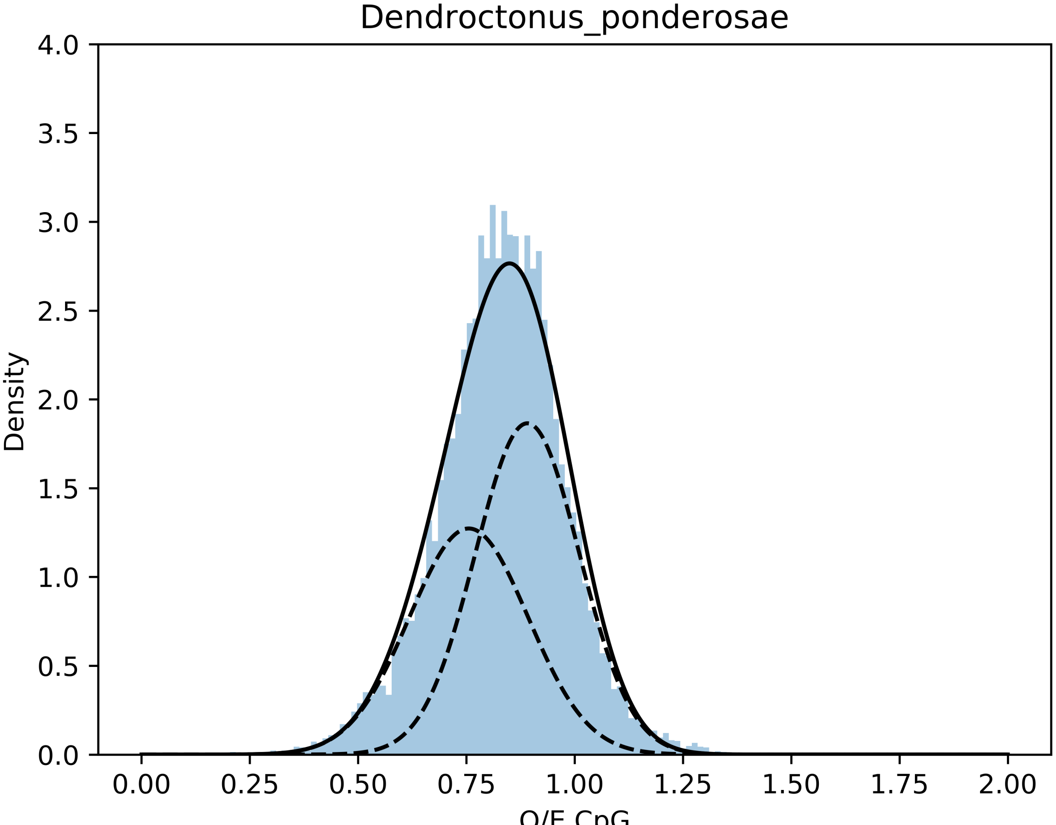

Dendroctonus_ponderosae
Density
O/E CpG
0.0
0.5
1.0
1.5
2.0
2.5
3.0
3.5
4.0
0.00
0.25
0.50
0.75
1.00
1.25
1.50
1.75
2.00

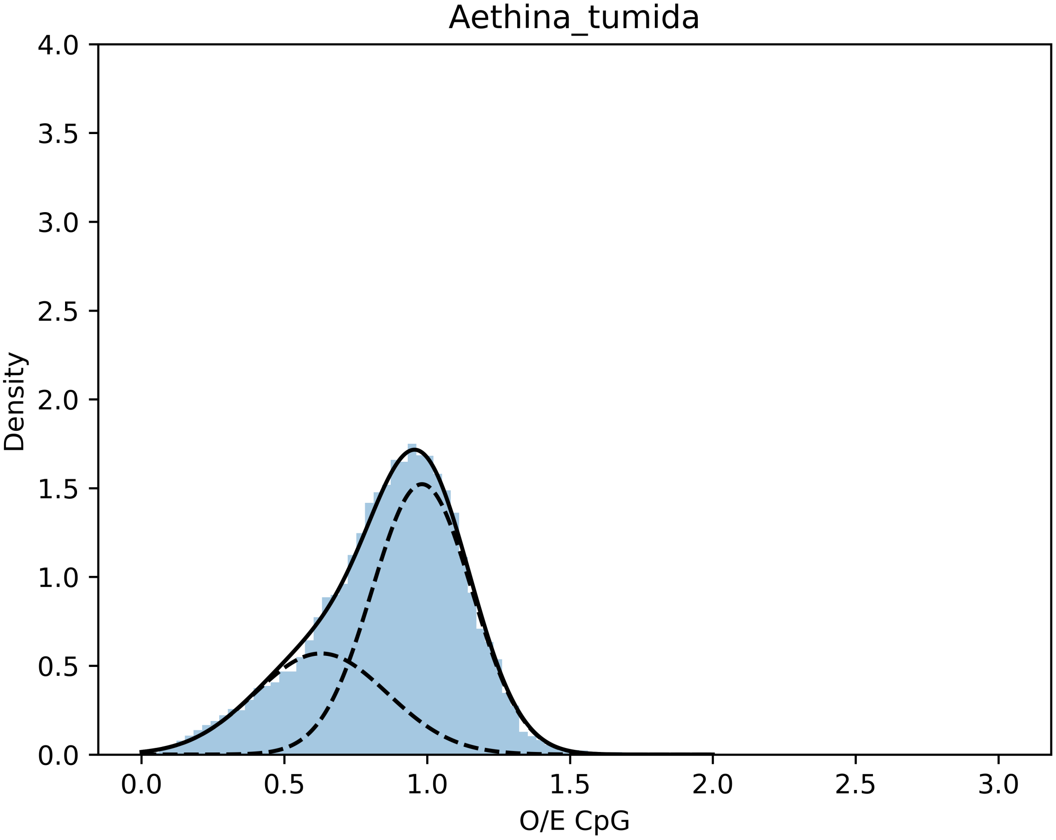

Aethina_tumida
Density
O/E CpG
0.0
0.5
1.0
1.5
2.0
2.5
3.0
3.5
4.0

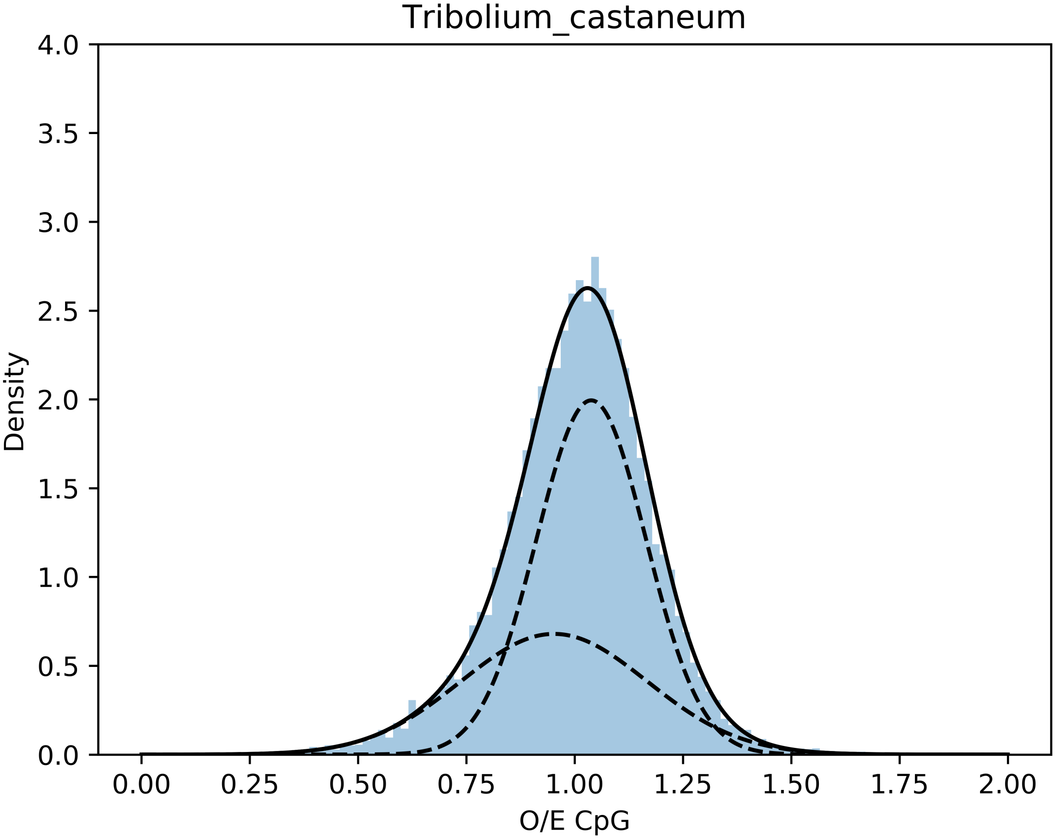

Tribolium_castaneum
Density
O/E CpG
0.0
0.5
1.0
1.5
2.0
2.5
3.0
3.5
4.0
0.00
0.25
0.50
0.75
1.00
1.25
1.50
1.75
2.00

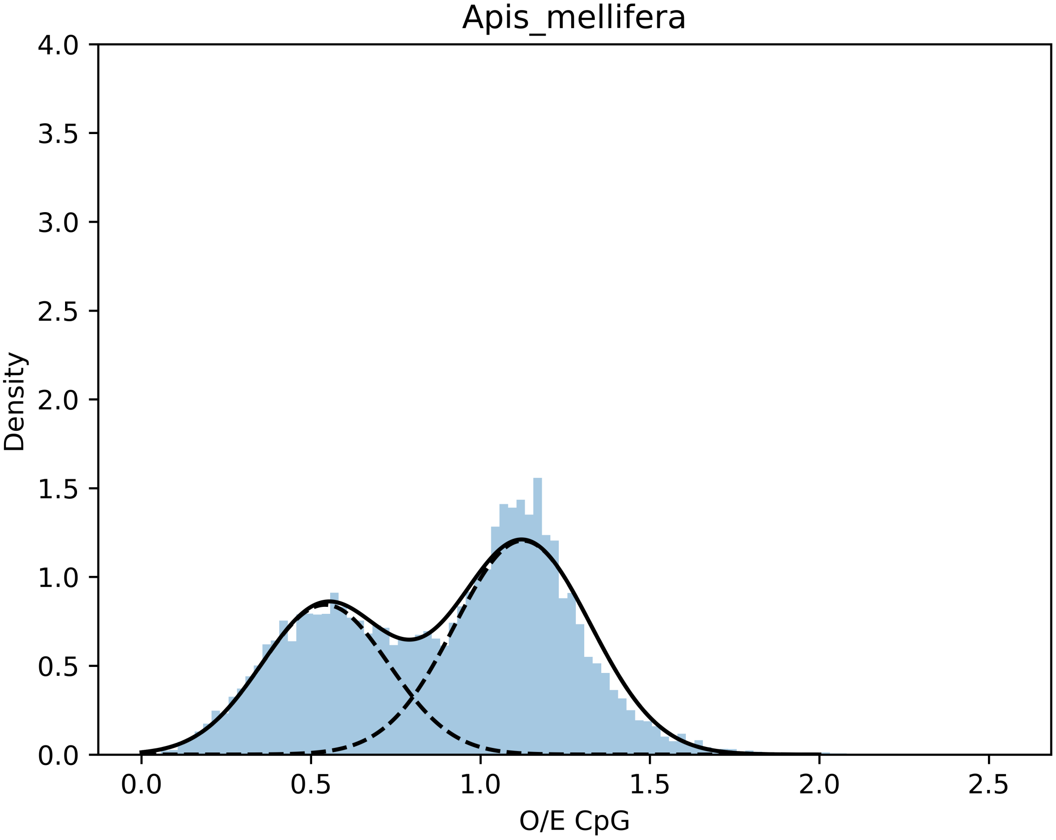

Apis_mellifera
Density
O/E CpG
0.0
0.5
1.0
1.5
2.0
2.5
3.0
3.5
4.0
0.0
0.5
1.0
1.5
2.0
2.5

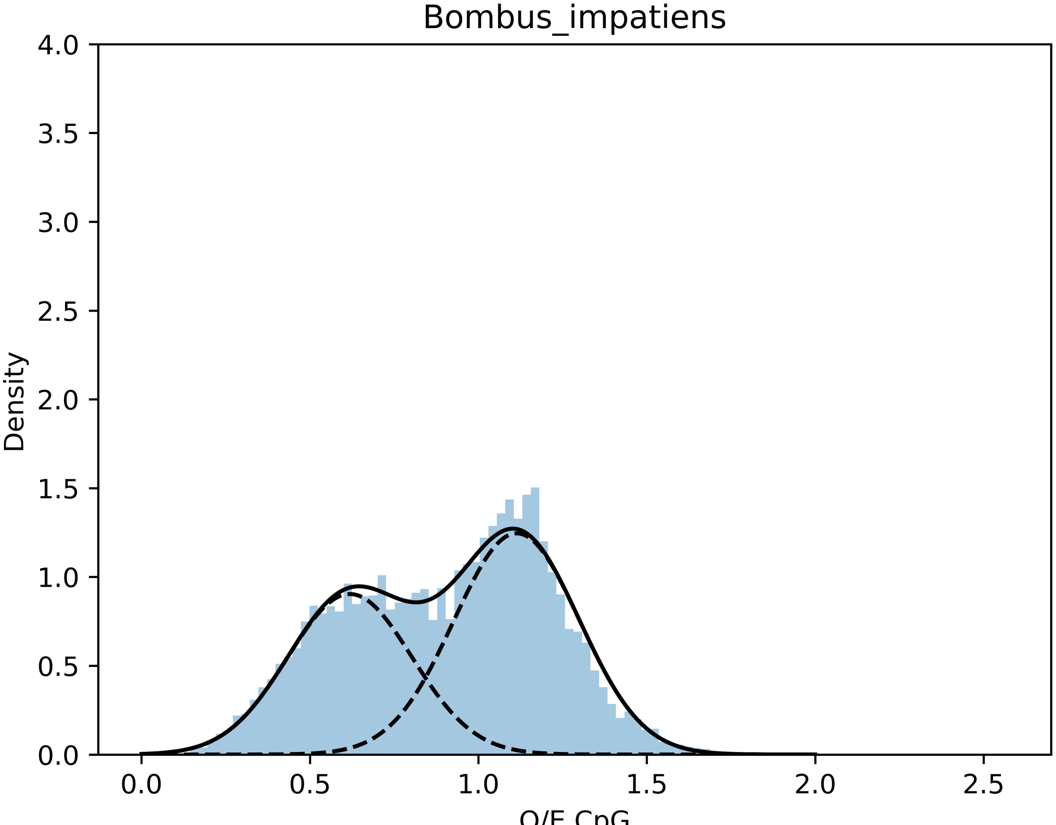

Bombus_impatiens
Density
O/E CpG
0.0
0.5
1.0
1.5
2.0
2.5
3.0
3.5
4.0
0.0
0.5
1.0
1.5
2.0
2.5

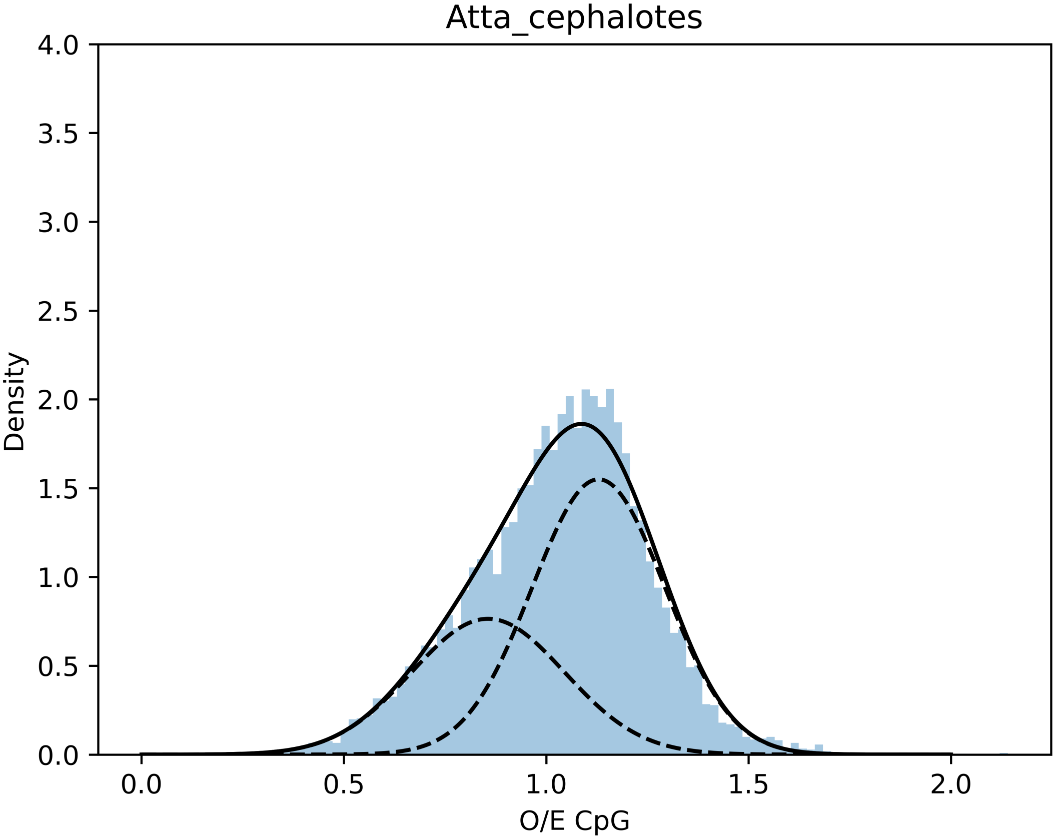
Atta_cephalotes
Density
O/E CpG
0.0
0.5
1.0
1.5
2.0
2.5
3.0
3.5
4.0

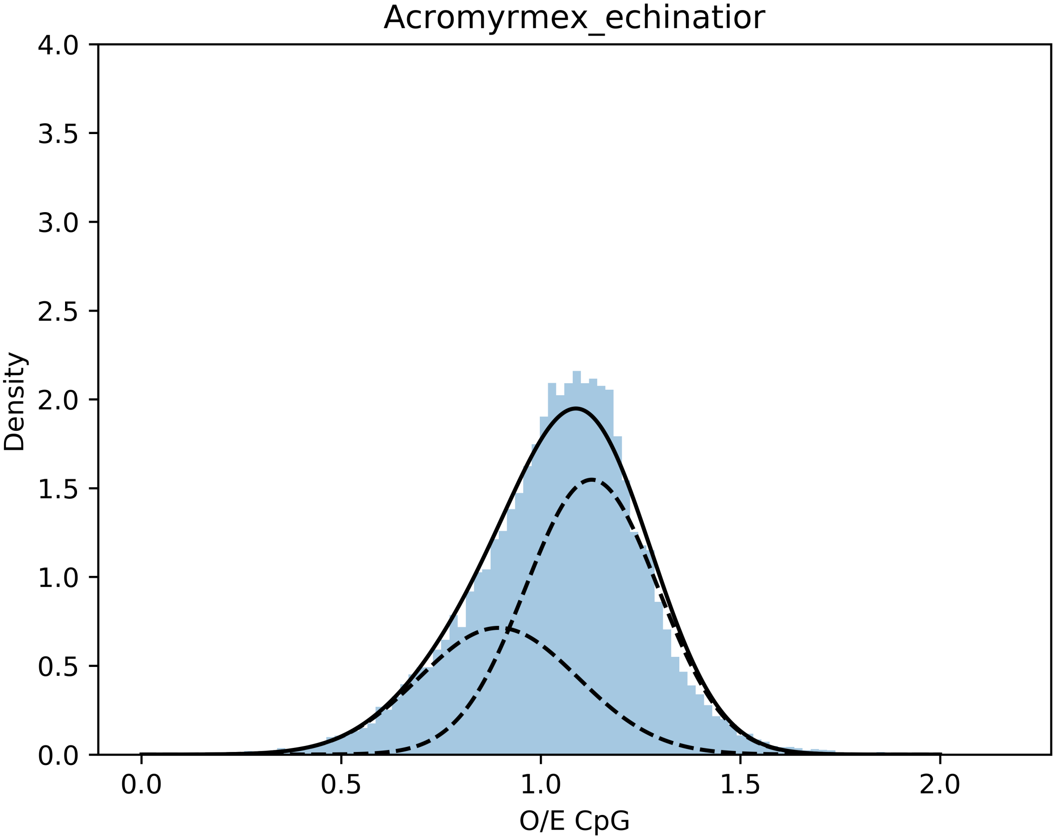

Acromyrmex_echinatior
Density
O/E CpG
4.0
3.5
3.0
2.5
2.0
1.5
1.0
0.5
0.0
0.0
0.5
1.0
1.5
2.0

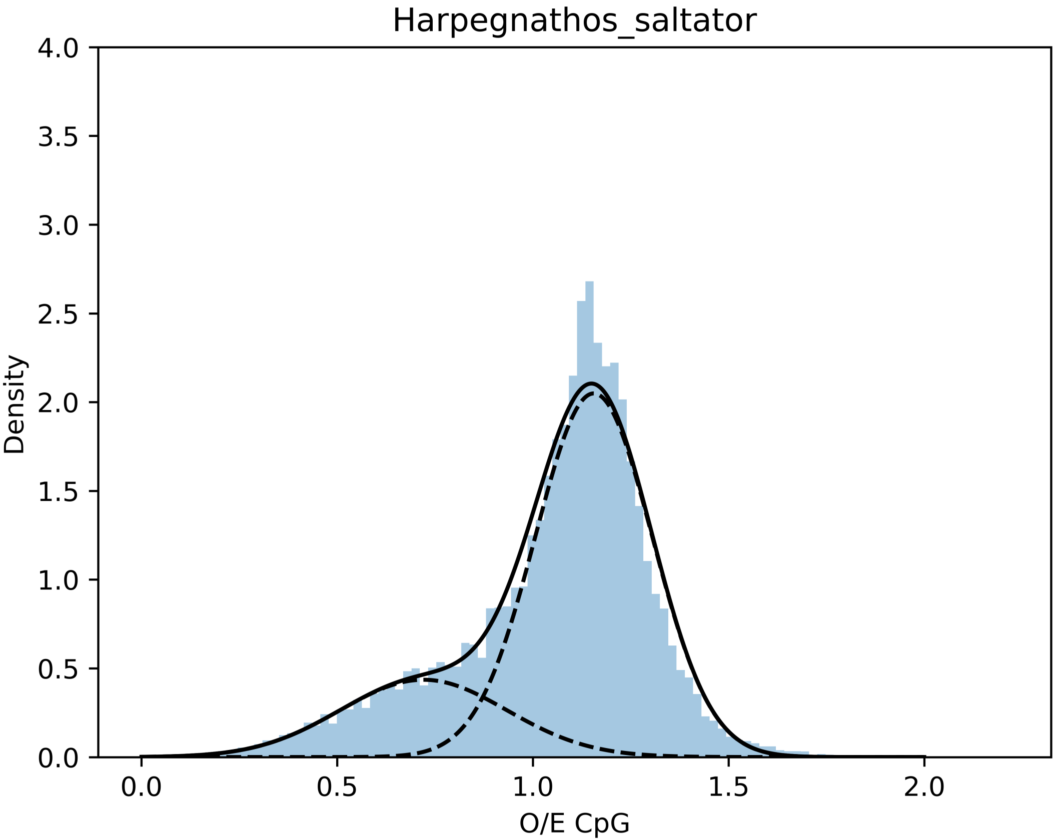
Harpegnathos_saltator
Density
O/E CpG
4.0
3.5
3.0
2.5
2.0
1.5
1.0
0.5
0.0
0.0
0.5
1.0
1.5
2.0

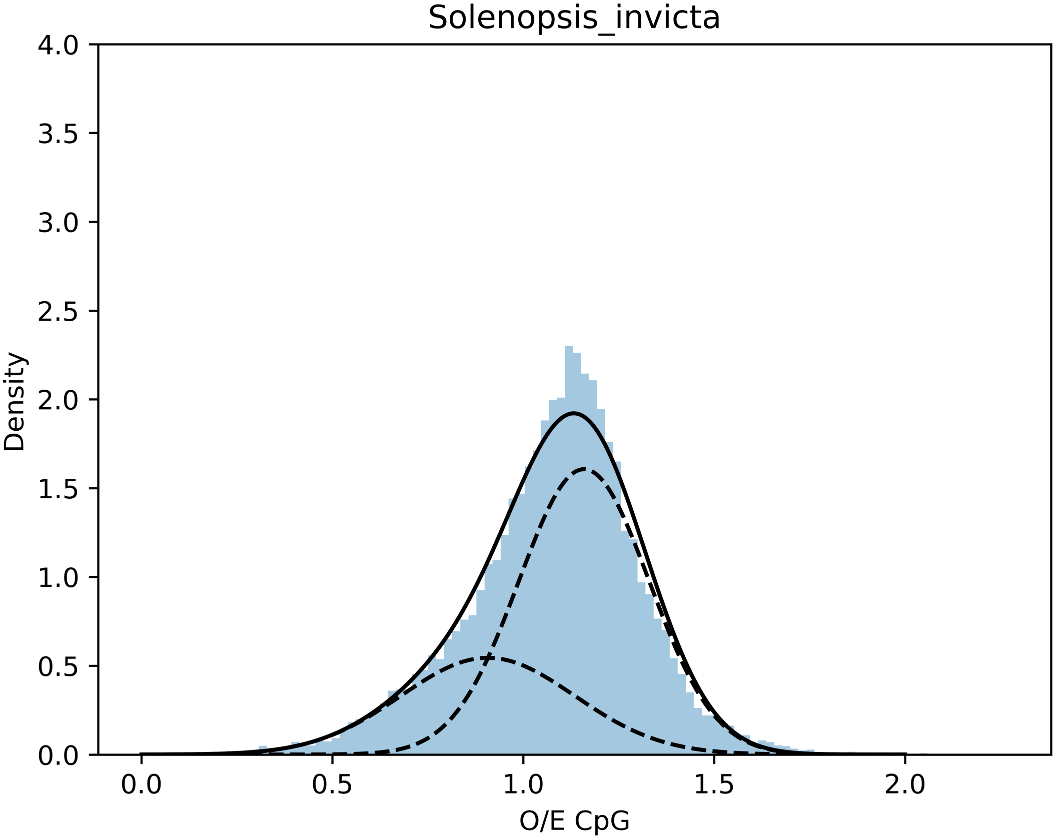
Solenopsis_invicta
Density
O/E CpG
0.0
0.5
1.0
1.5
2.0
2.5
3.0
3.5
4.0
0.0
0.5
1.0
1.5
2.0

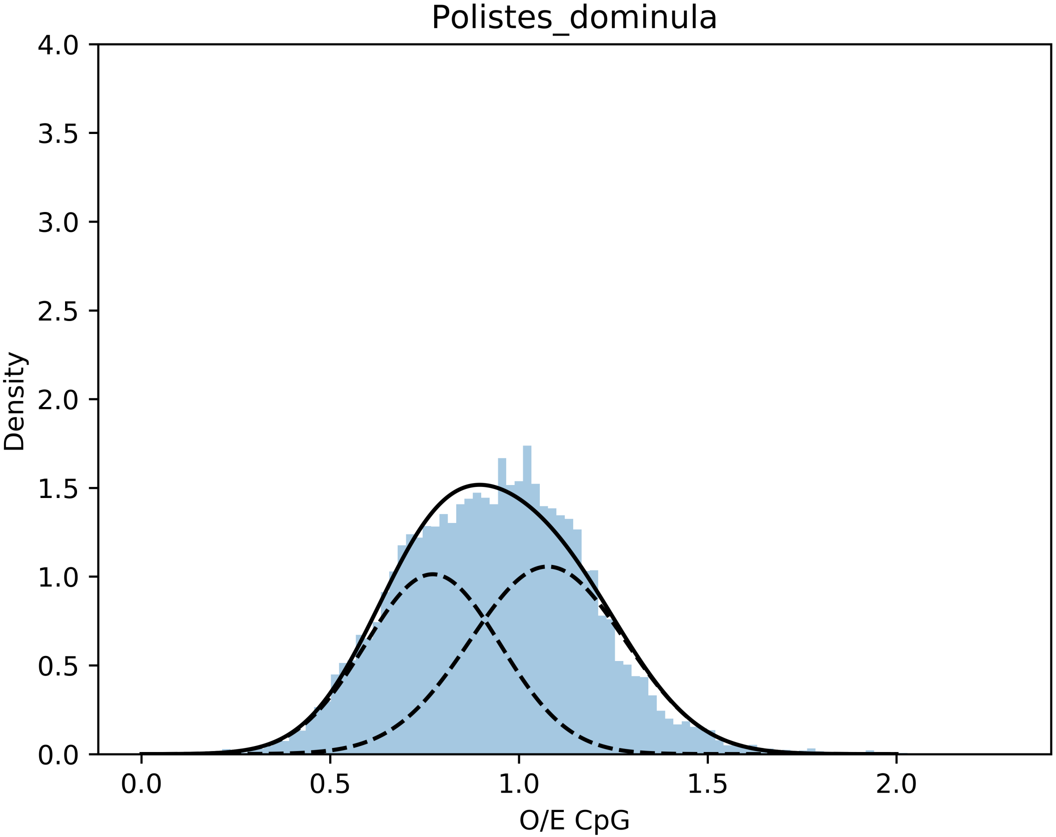

Polistes_dominula
Density
O/E CpG
0.0
0.5
1.0
1.5
2.0
2.5
3.0
3.5
4.0
0.0
0.5
1.0
1.5
2.0

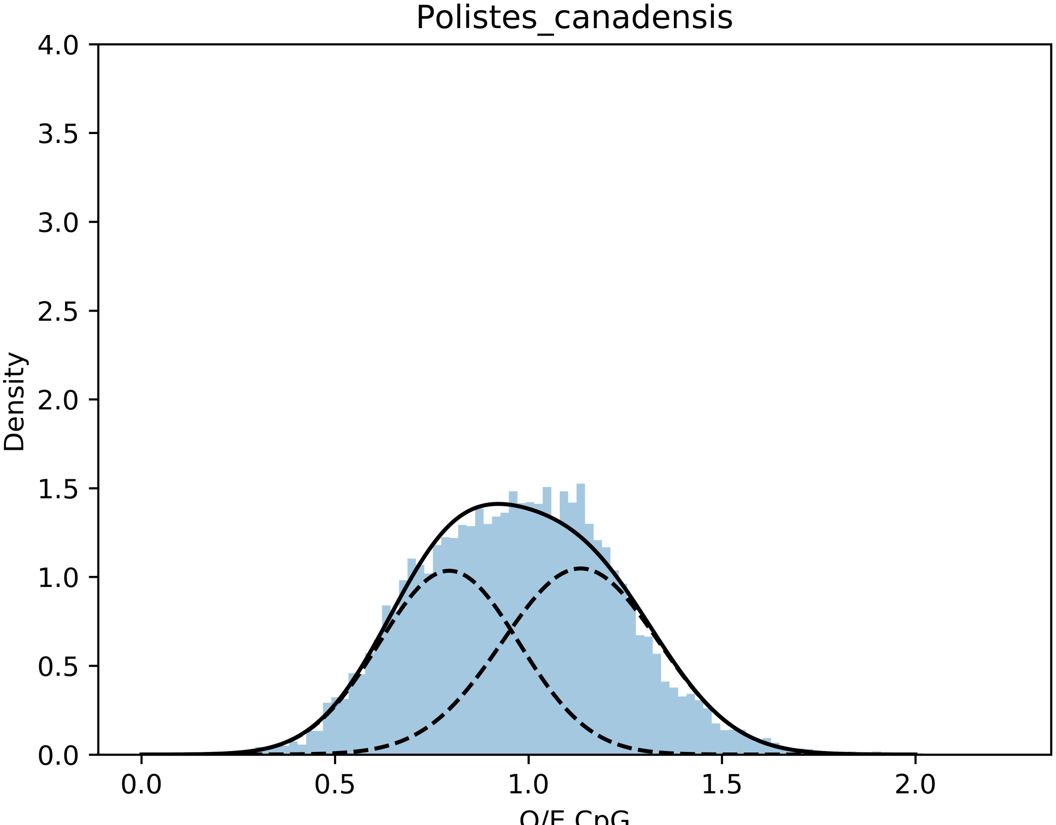
Polistes_canadensis
Density
O/E CpG
0.0
0.5
1.0
1.5
2.0
2.5
3.0
3.5
4.0
0.0
0.5
1.0
1.5
2.0

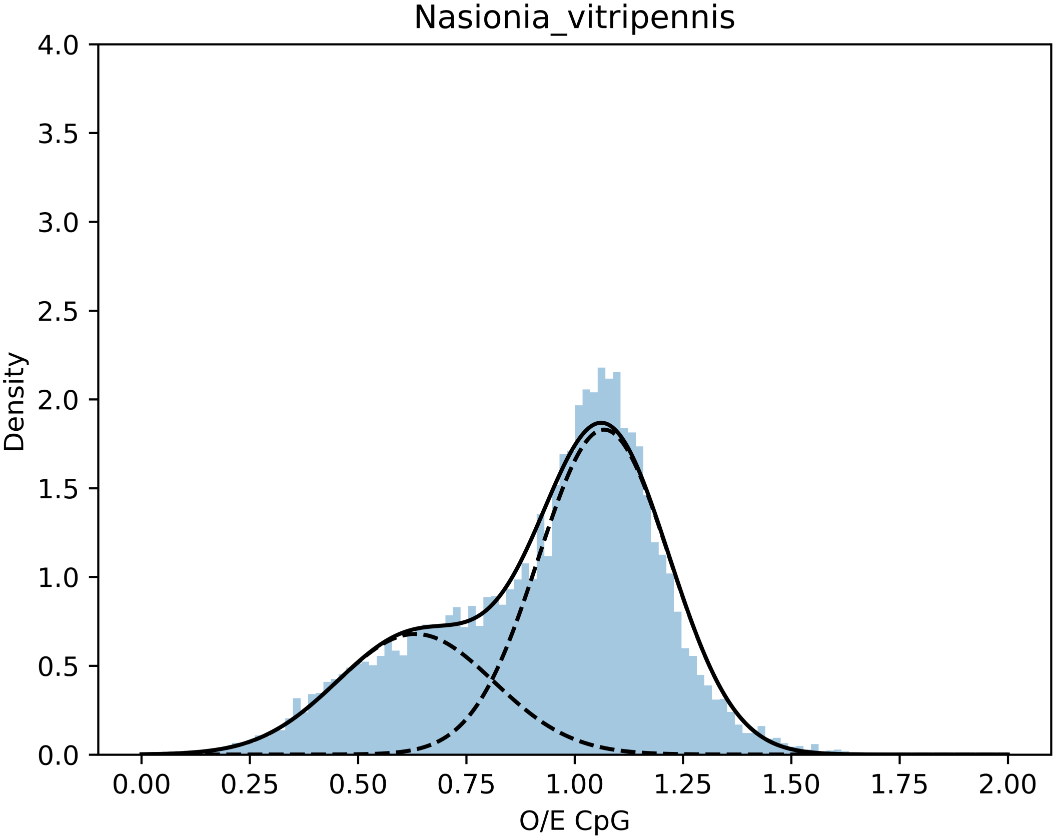
Nasionia_vitripennis
Density
O/E CpG
0.0
0.5
1.0
1.5
2.0
2.5
3.0
3.5
4.0
0.00
0.25
0.50
0.75
1.00
1.25
1.50
1.75
2.00

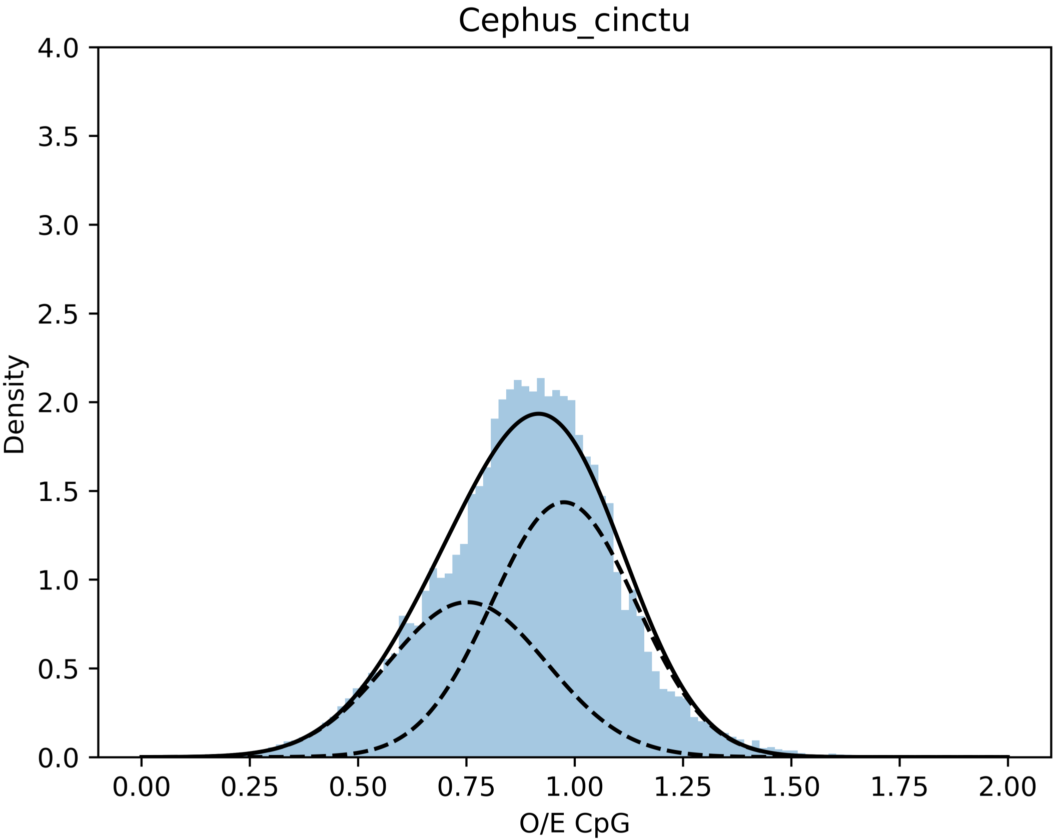
Cephus_cinctu
Density
O/E CpG
0.0
0.5
1.0
1.5
2.0
2.5
3.0
3.5
4.0
0.00
0.25
0.50
0.75
1.00
1.25
1.50
1.75
2.00

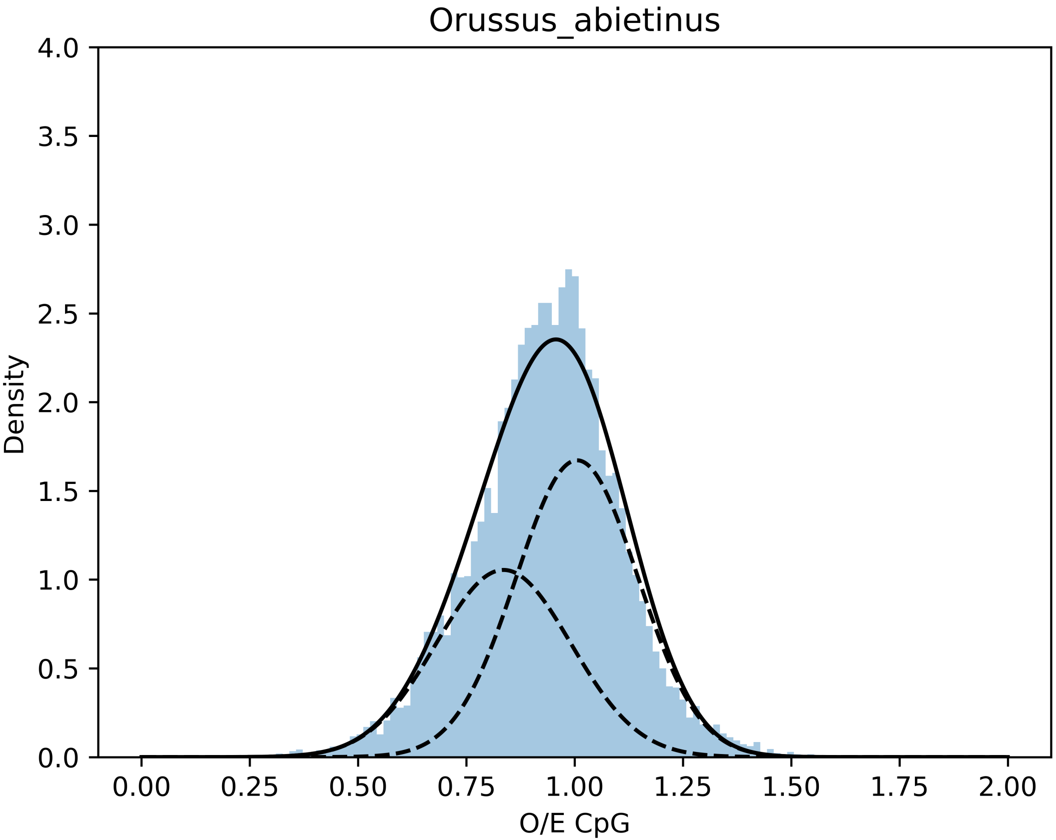

Orussus_abietinus
Density
O/E CpG
0.0
0.5
1.0
1.5
2.0
2.5
3.0
3.5
4.0
0.00
0.25
0.50
0.75
1.00
1.25
1.50
1.75
2.00

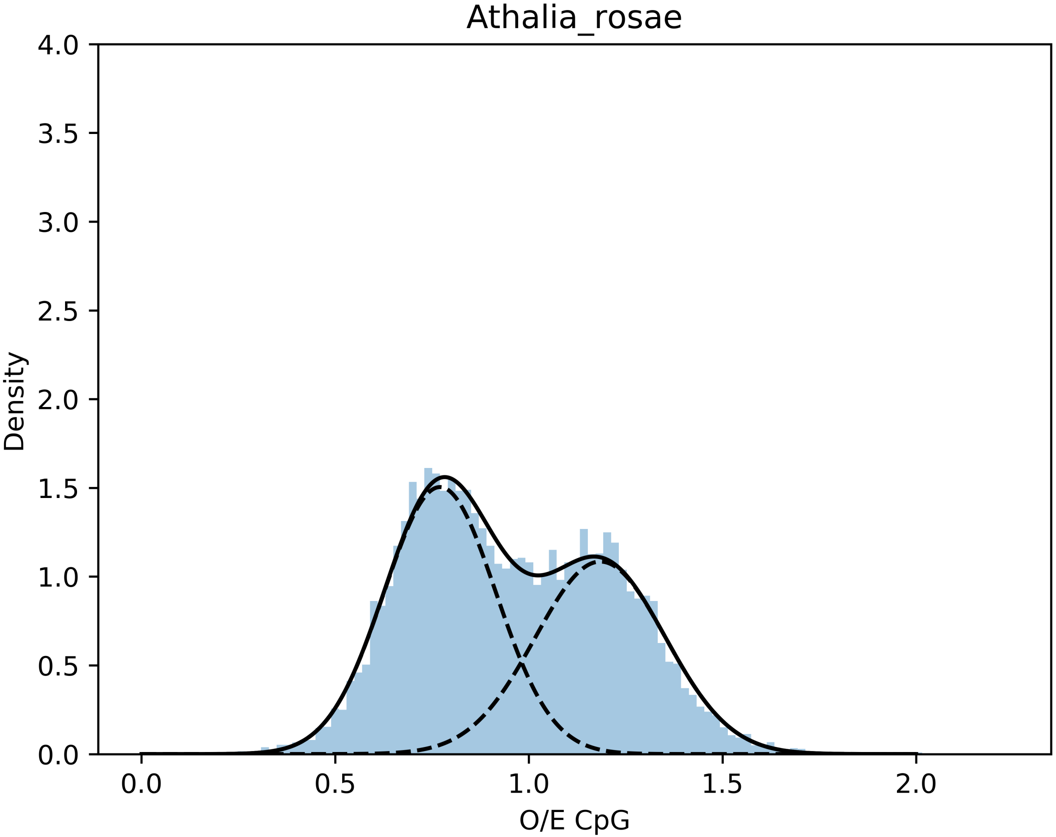
Athalia_rosae
Density
O/E CpG
0.0
0.5
1.0
1.5
2.0
2.5
3.0
3.5
4.0
0.0
0.5
1.0
1.5
2.0

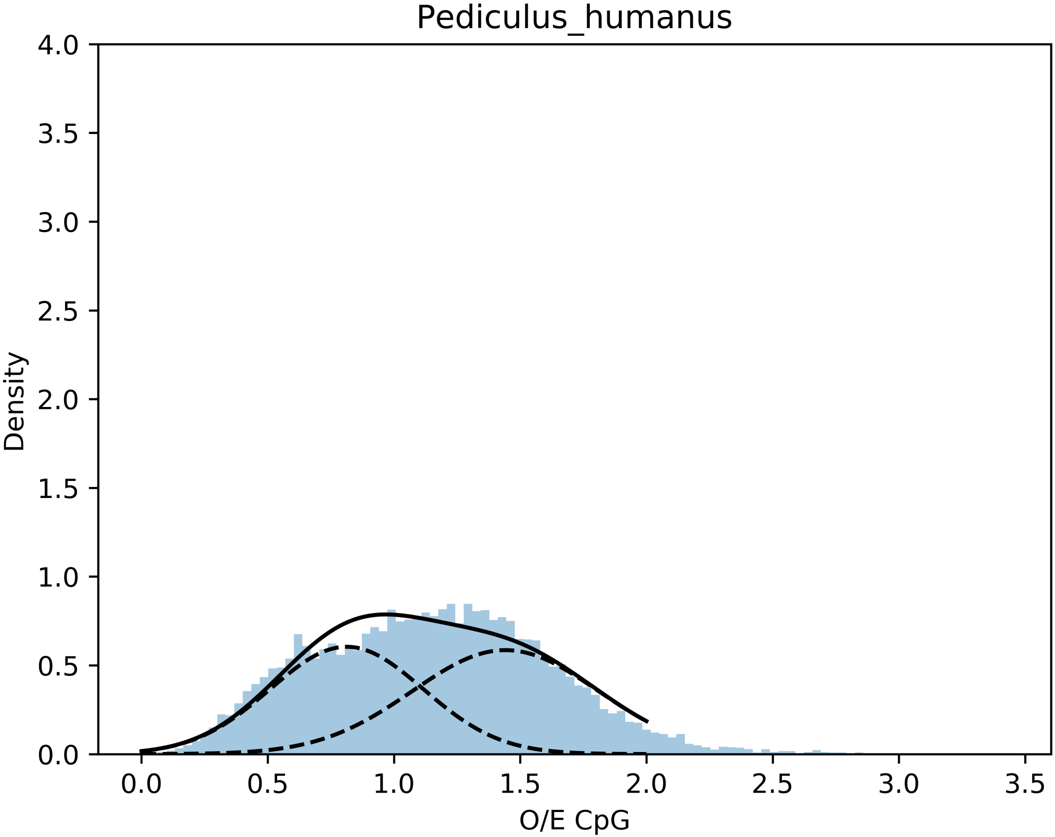

Pediculus_humanus
Density
O/E CpG
4.0
3.5
3.0
2.5
2.0
1.5
1.0
0.5
0.0
0.0
0.5
1.0
1.5
2.0
2.5
3.0
3.5

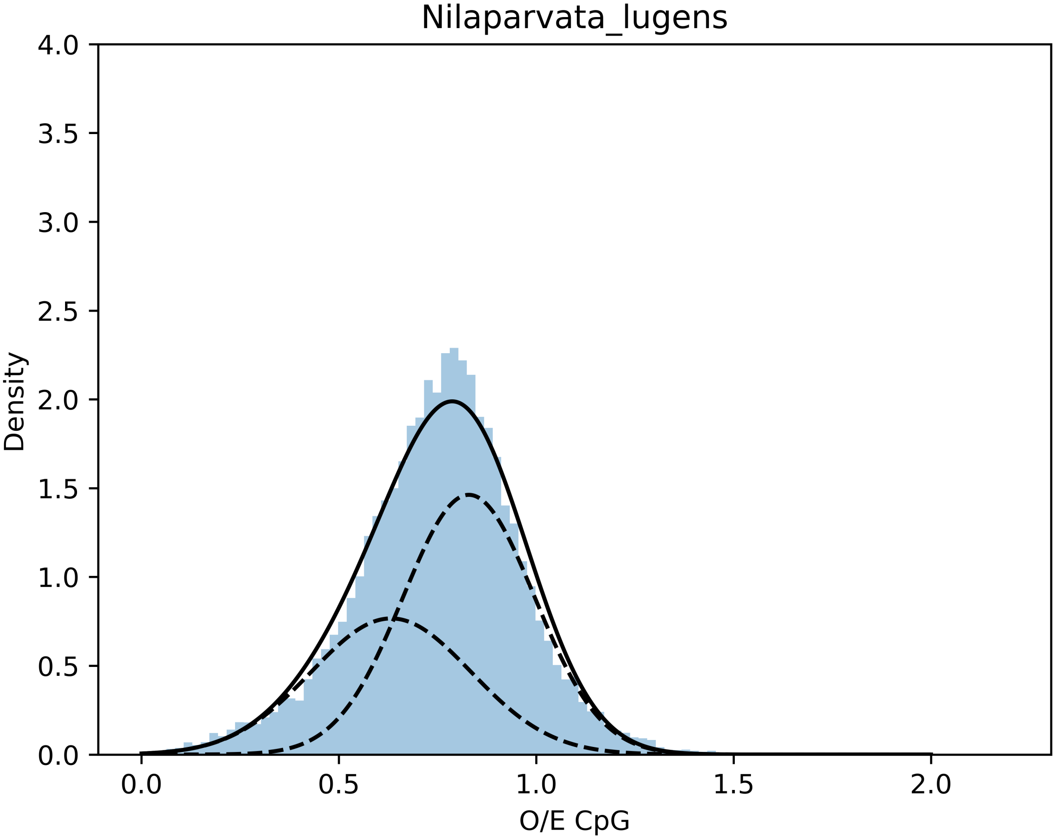

Nilaparvata_lugens
Density
O/E CpG
0.0
0.5
1.0
1.5
2.0
2.5
3.0
3.5
4.0
0.0
0.5
1.0
1.5
2.0

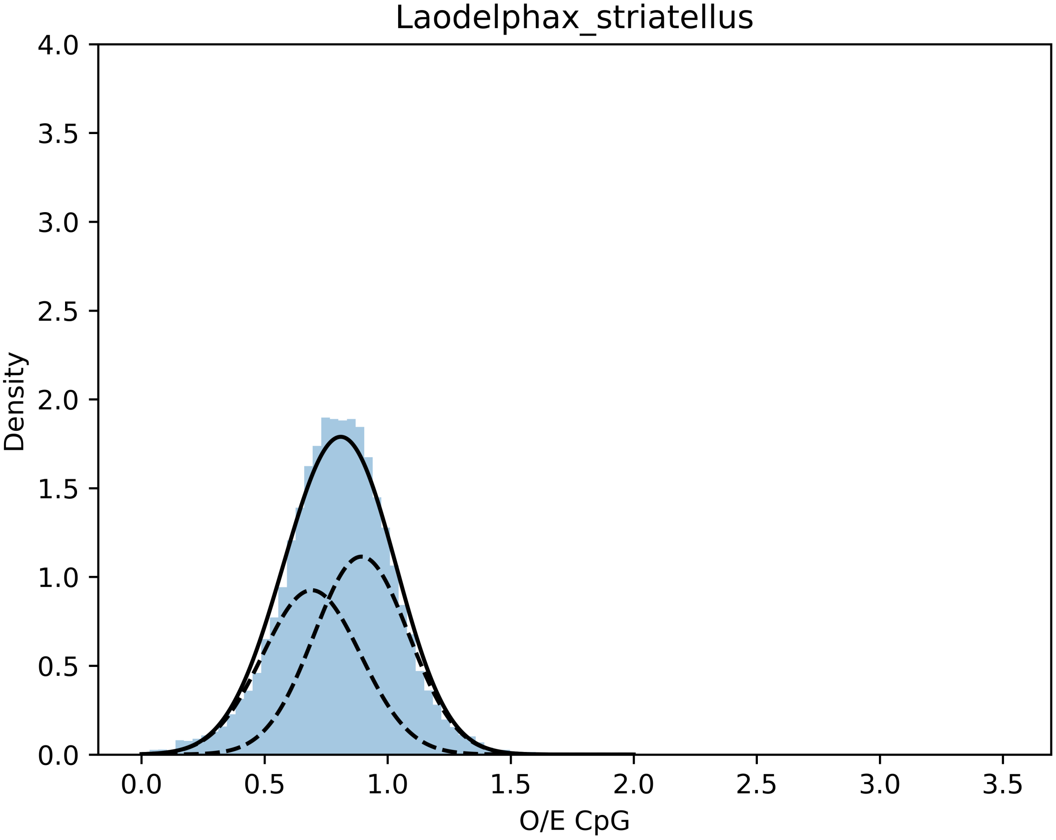
Laodelphax_striatellus
Density
O/E CpG
4.0
3.5
3.0
2.5
2.0
1.5
1.0
0.5
0.0
0.0
0.5
1.0
1.5
2.0
2.5
3.0
3.5

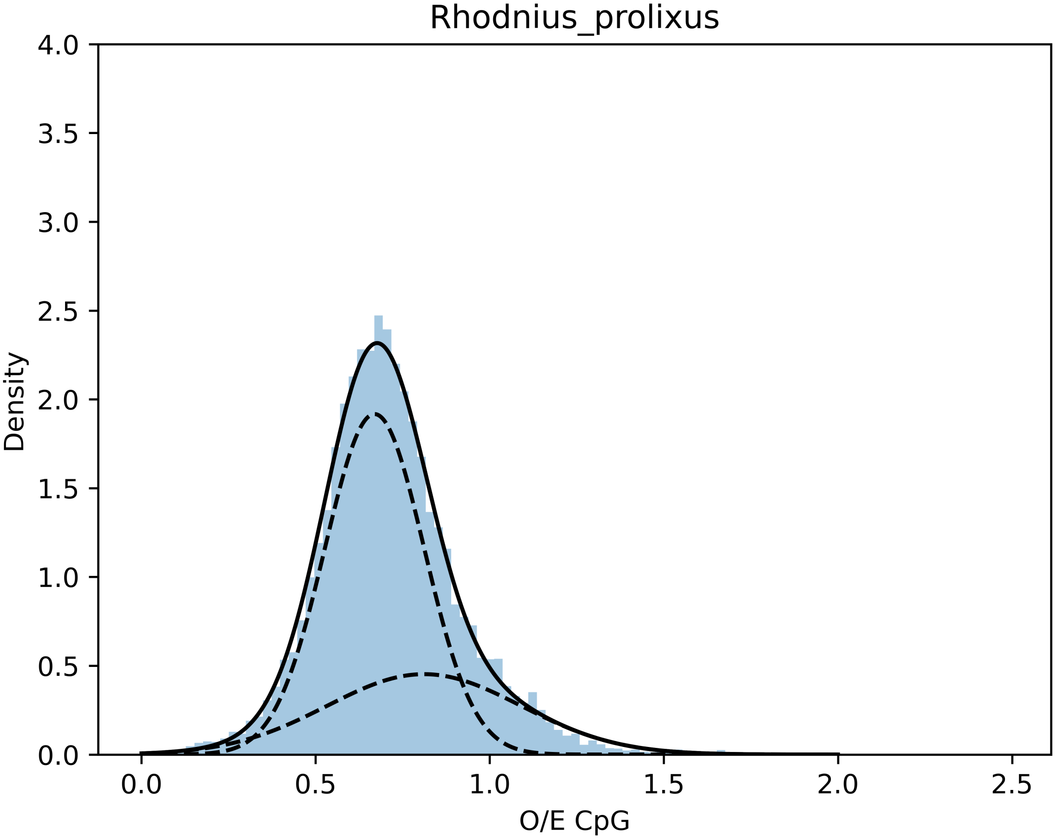
Rhodnius_prolixus
Density
O/E CpG
4.0
3.5
3.0
2.5
2.0
1.5
1.0
0.5
0.0
0.0
0.5
1.0
1.5
2.0
2.5

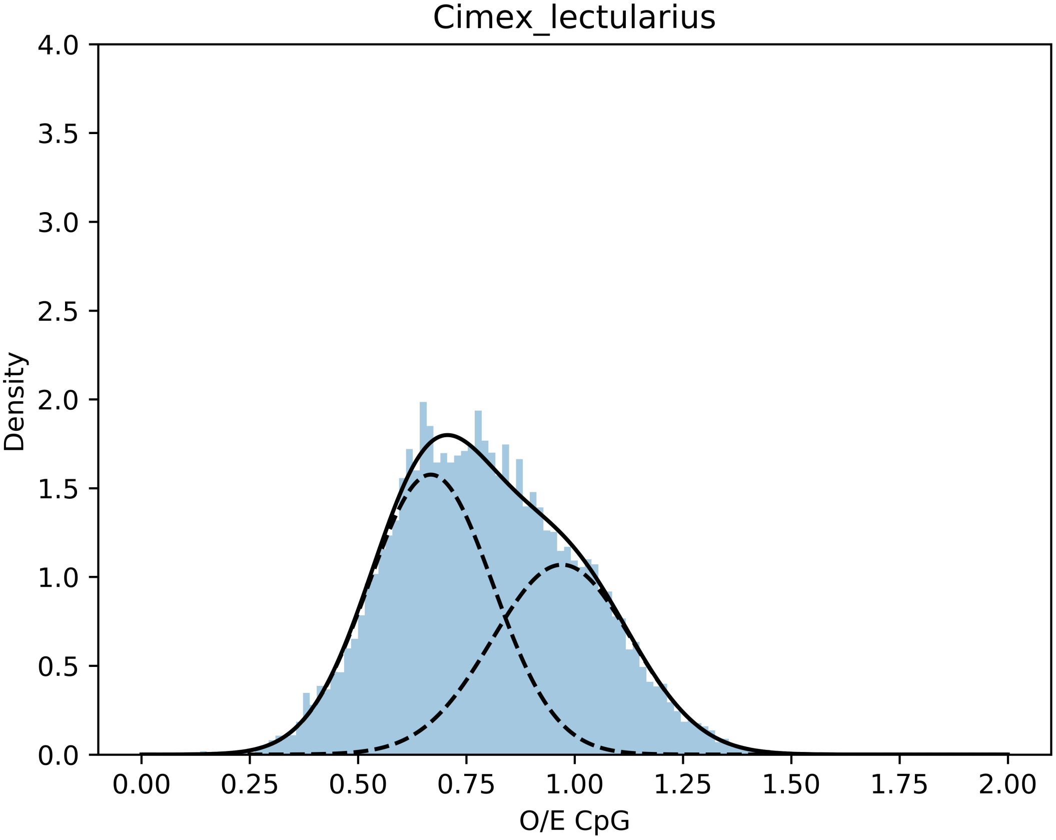

Cimex_lectularius
Density
O/E CpG
4.0
3.5
3.0
2.5
2.0
1.5
1.0
0.5
0.0
0.00
0.25
0.50
0.75
1.00
1.25
1.50
1.75
2.00

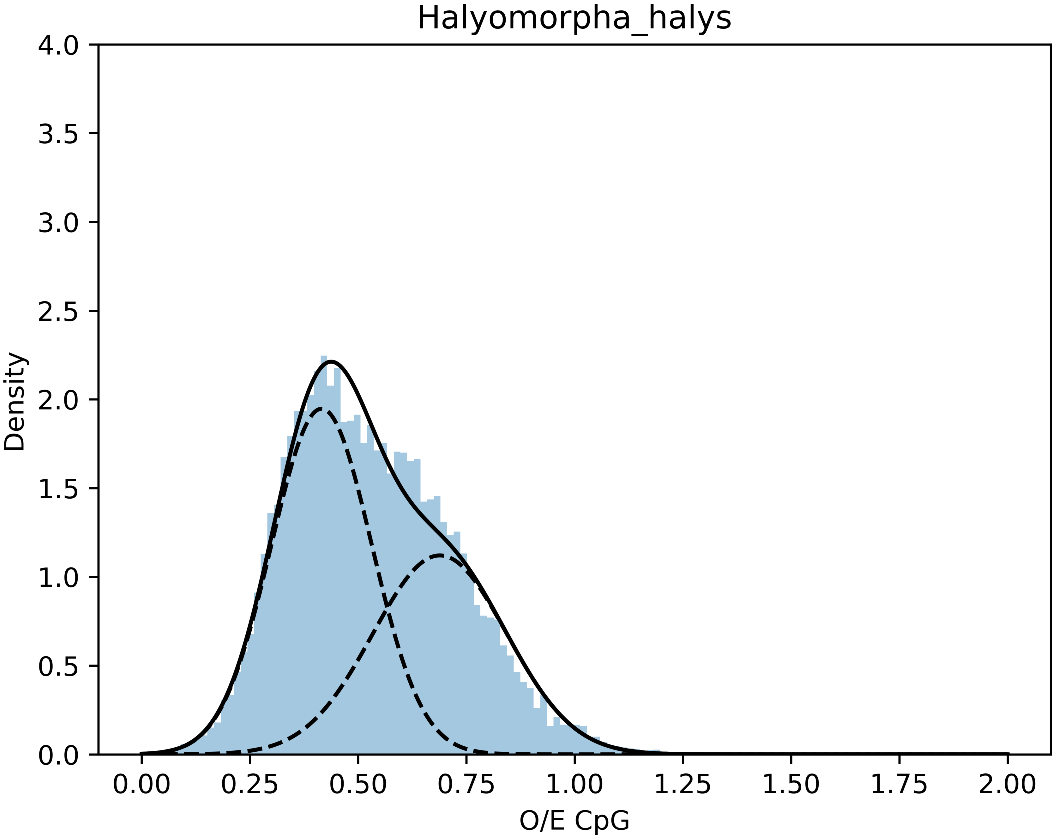

Halyomorpha_halys
Density
O/E CpG
4.0
3.5
3.0
2.5
2.0
1.5
1.0
0.5
0.0
0.00
0.25
0.50
0.75
1.00
1.25
1.50
1.75
2.00

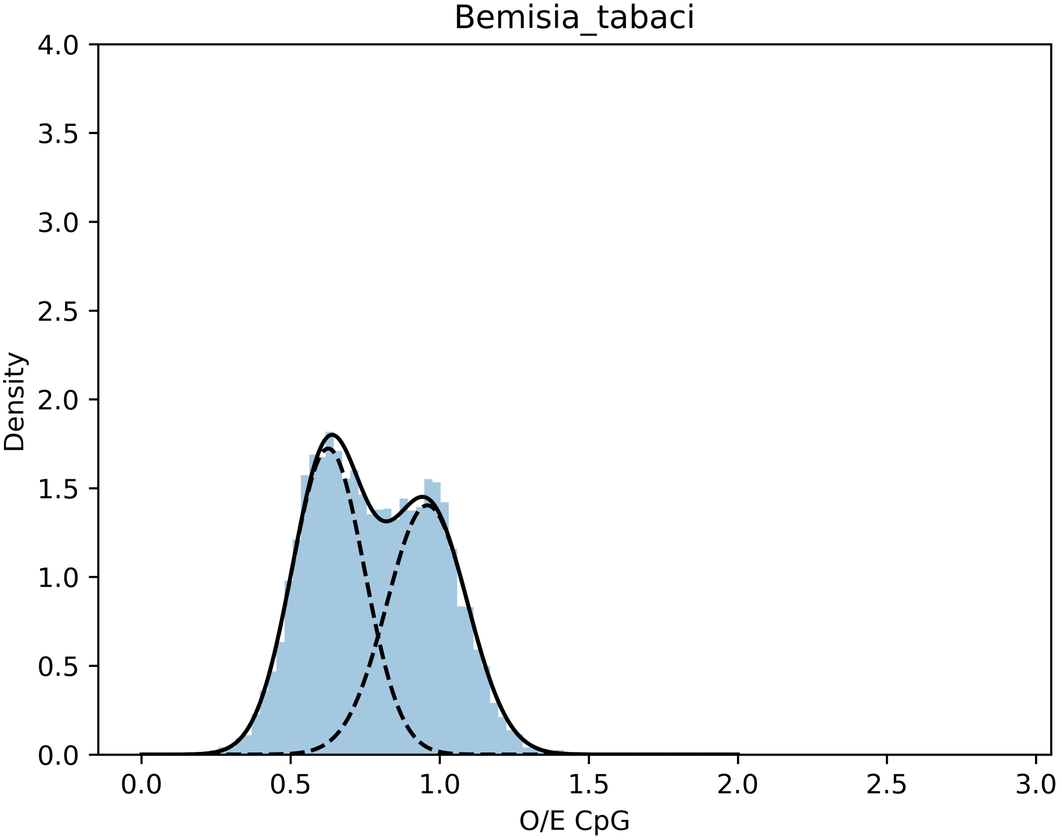
Bemisia_tabaci
Density
O/E CpG
0.0
0.5
1.0
1.5
2.0
2.5
3.0
3.5
4.0

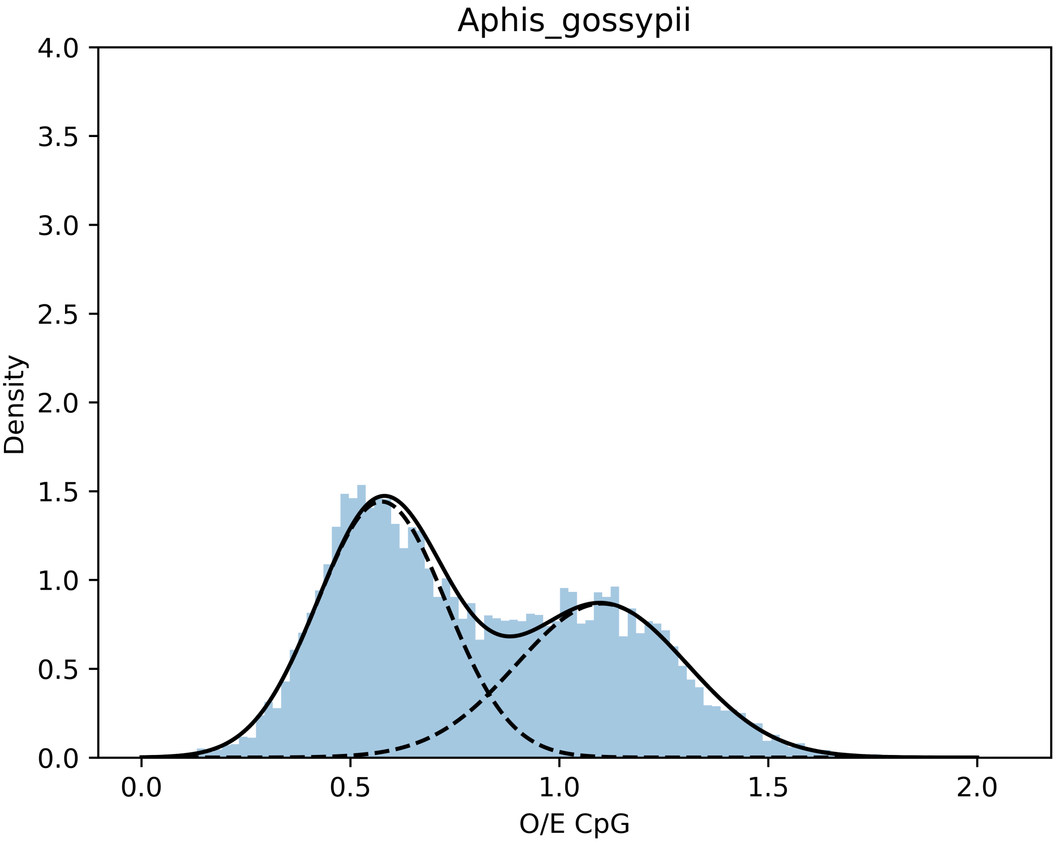

Aphis_gossypii
Density
O/E CpG
4.0
3.5
3.0
2.5
2.0
1.5
1.0
0.5
0.0
0.0
0.5
1.0
1.5
2.0

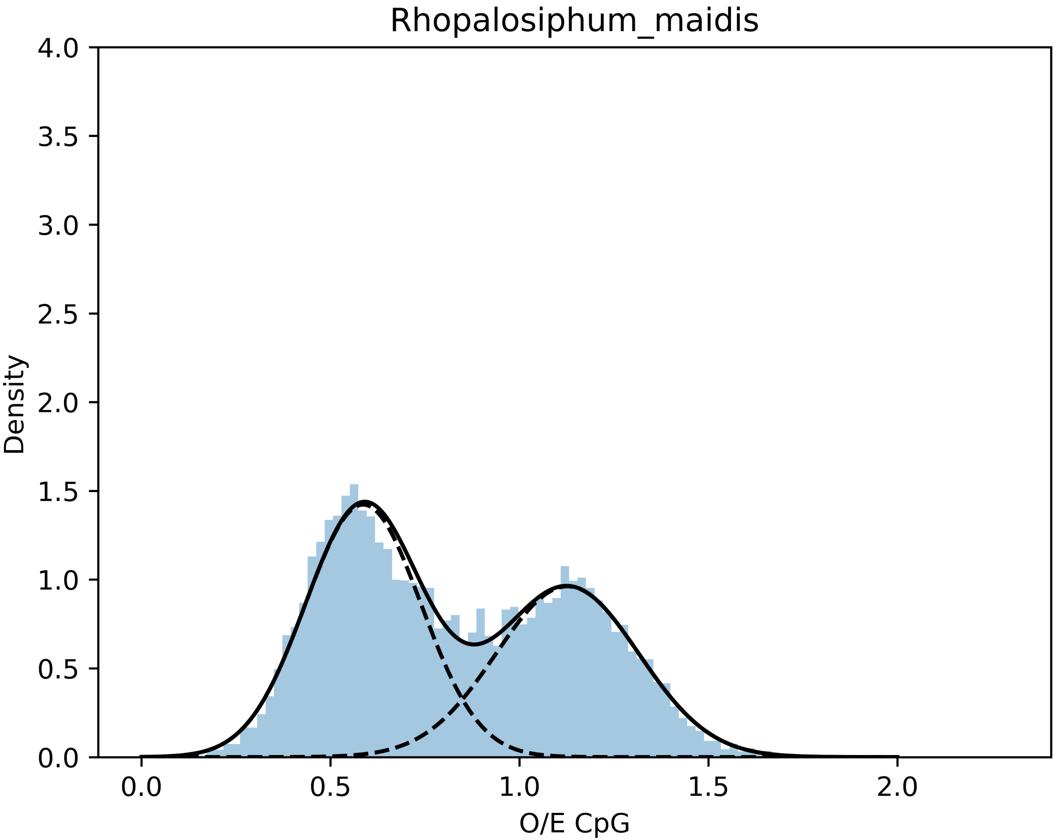

Rhopalosiphum_maidis
Density
O/E CpG
0.0
0.5
1.0
1.5
2.0
2.5
3.0
3.5
4.0
0.0
0.5
1.0
1.5
2.0

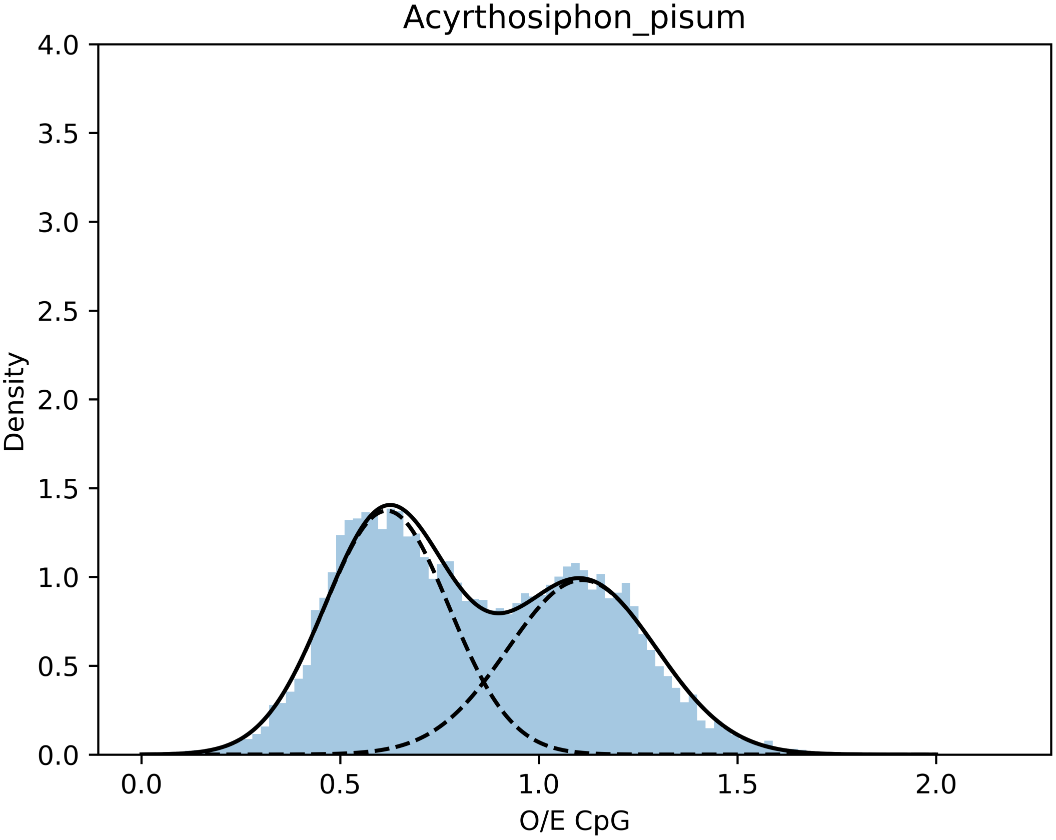
Acyrthosiphon_pisum
Density
O/E CpG
0.0
0.5
1.0
1.5
2.0
2.5
3.0
3.5
4.0
0.0
0.5
1.0
1.5
2.0

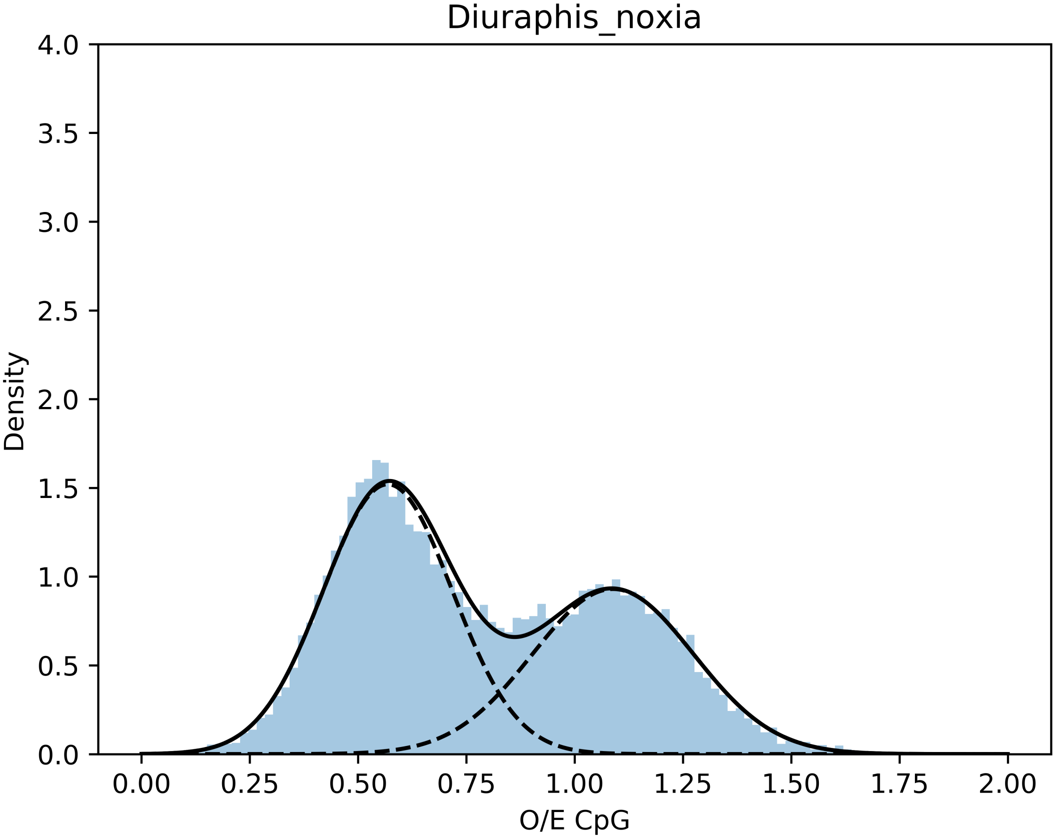
Diuraphis_noxia
Density
O/E CpG
4.0
3.5
3.0
2.5
2.0
1.5
1.0
0.5
0.0
0.00
0.25
0.50
0.75
1.00
1.25
1.50
1.75
2.00

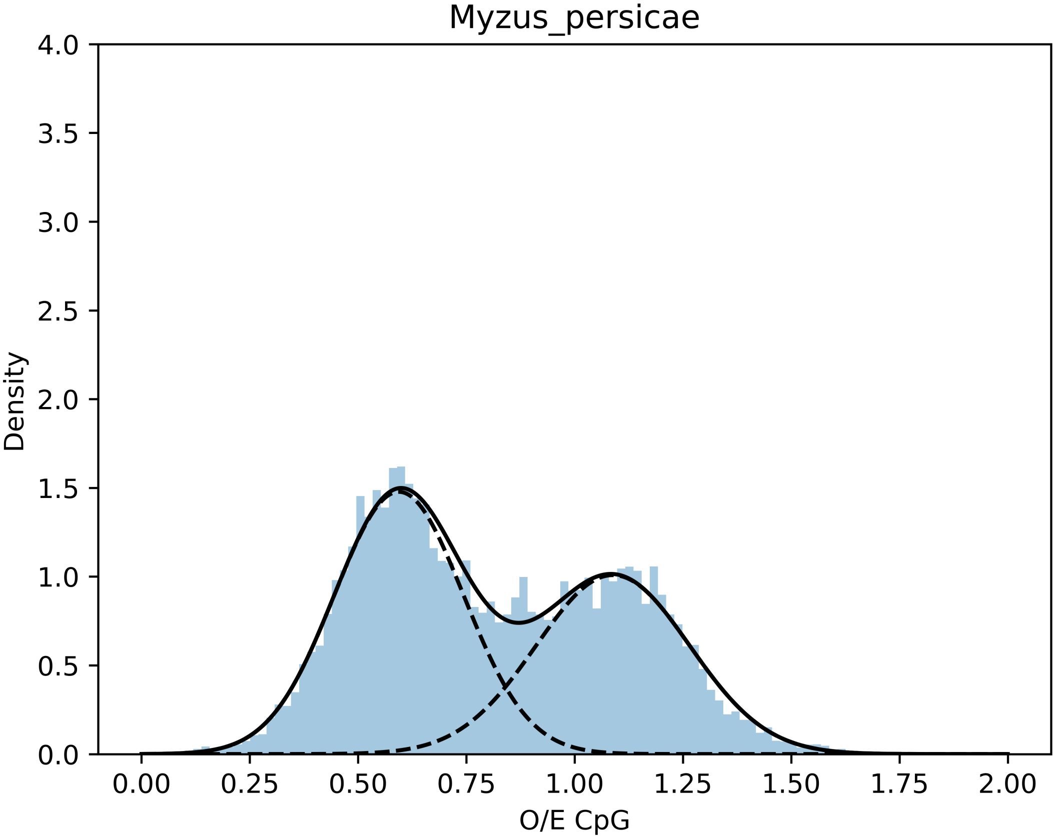

Myzus_persicae
Density
O/E CpG
4.0
3.5
3.0
2.5
2.0
1.5
1.0
0.5
0.0
0.00
0.25
0.50
0.75
1.00
1.25
1.50
1.75
2.00

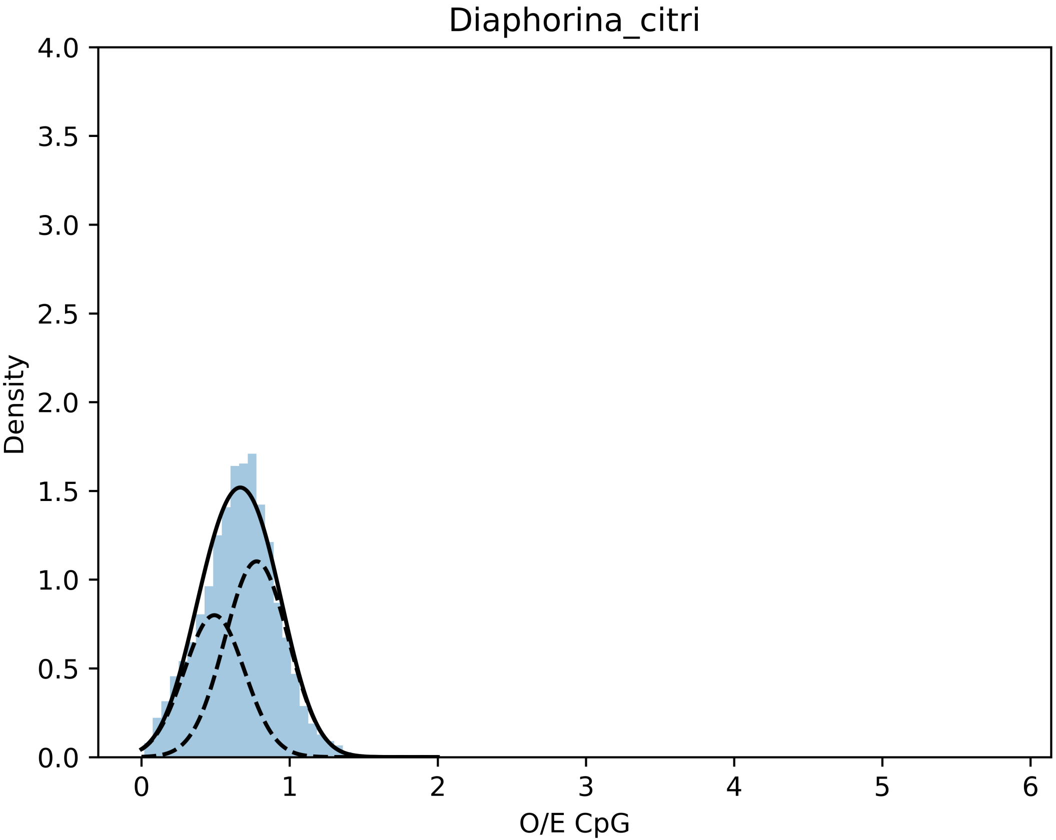
Diaphorina_citri
Density
O/E CpG
4.0
3.5
3.0
2.5
2.0
1.5
1.0
0.5
0.0
0
1
2
3
4
5
6

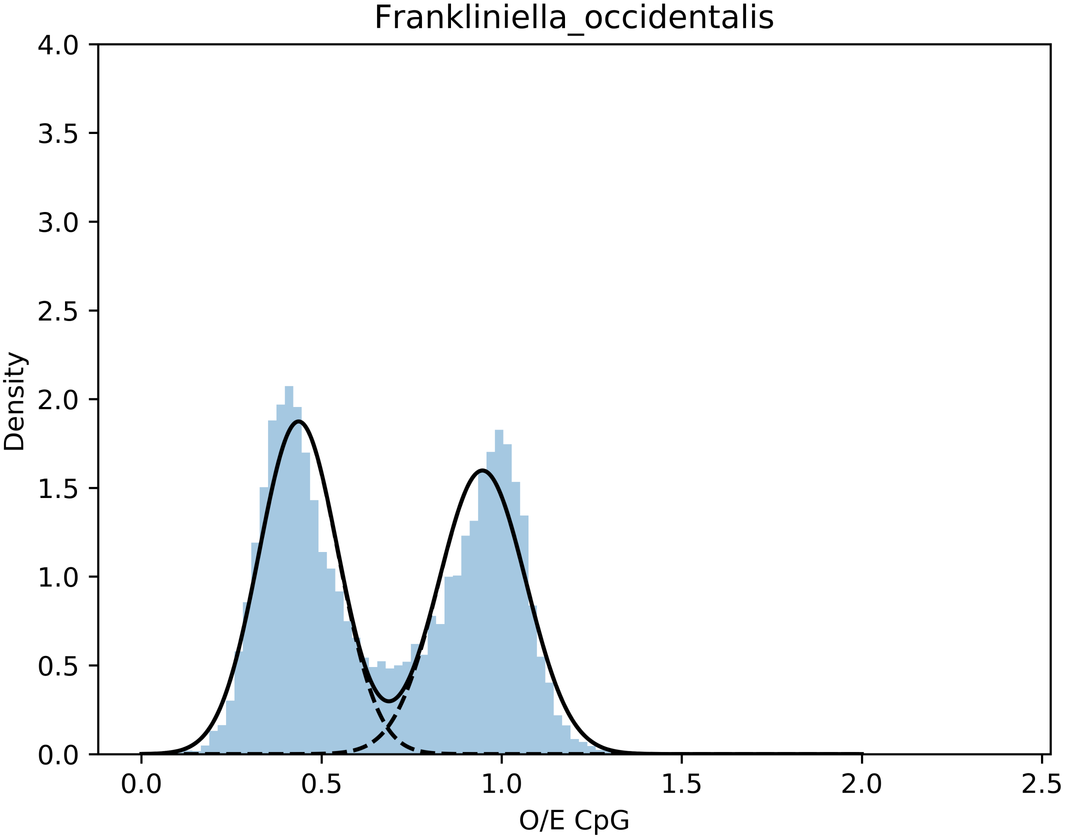

Frankliniella_occidentalis
Density
O/E CpG
0.0
0.5
1.0
1.5
2.0
2.5
3.0
3.5
4.0
0.0
0.5
1.0
1.5
2.0
2.5

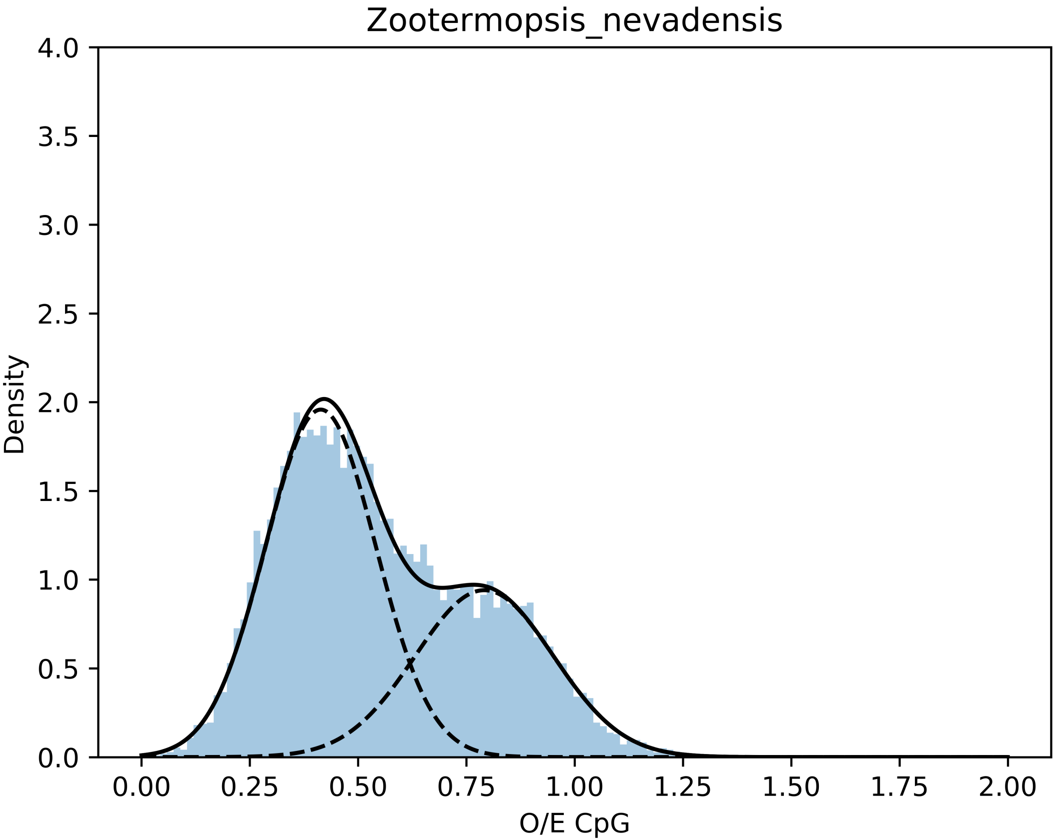
Zootermopsis_nevadensis
Density
O/E CpG
0.0
0.5
1.0
1.5
2.0
2.5
3.0
3.5
4.0
0.00
0.25
0.50
0.75
1.00
1.25
1.50
1.75
2.00

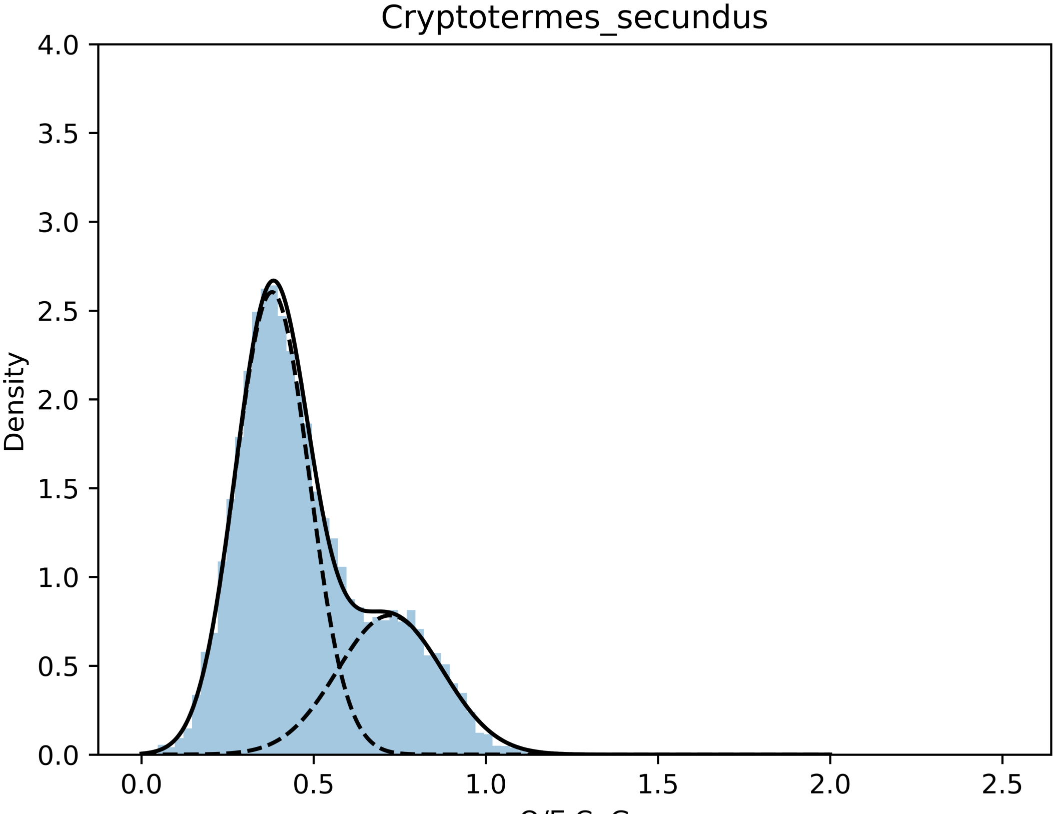
Cryptotermes_secundus
Density
O/E CpG
0.0
0.5
1.0
1.5
2.0
2.5
3.0
3.5
4.0

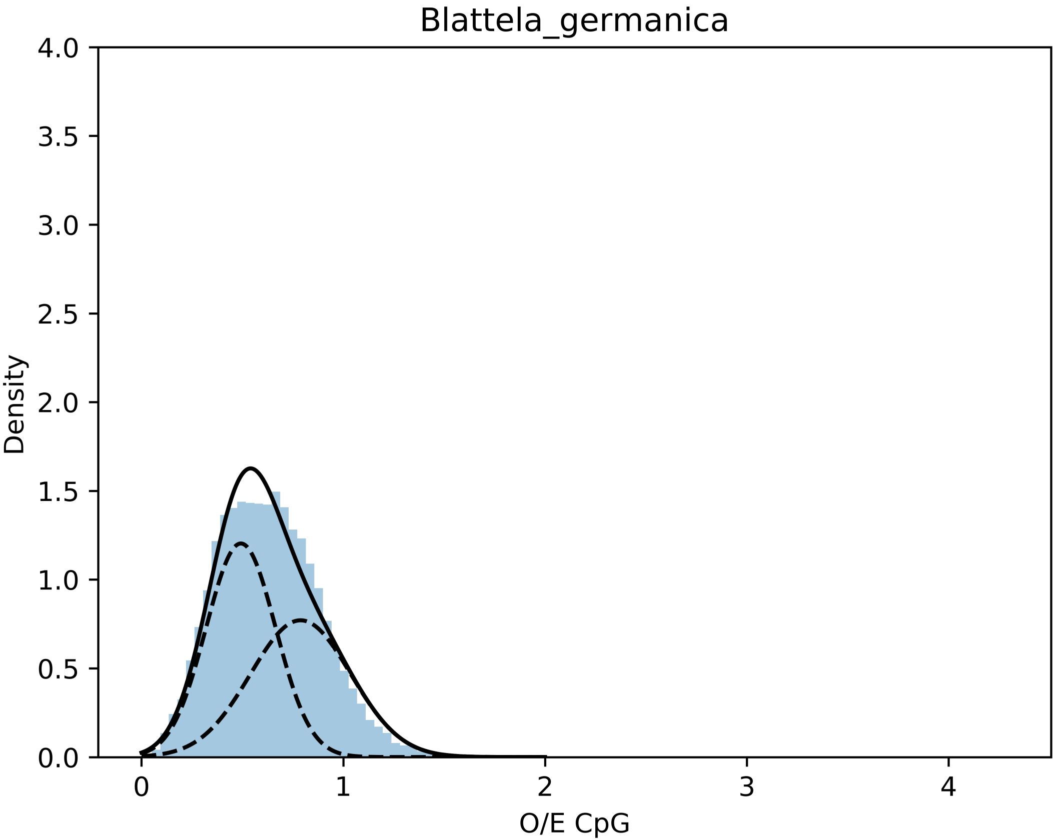

Blattela_germanica
Density
O/E CpG
4.0
3.5
3.0
2.5
2.0
1.5
1.0
0.5
0.0
0
1
2
3
4

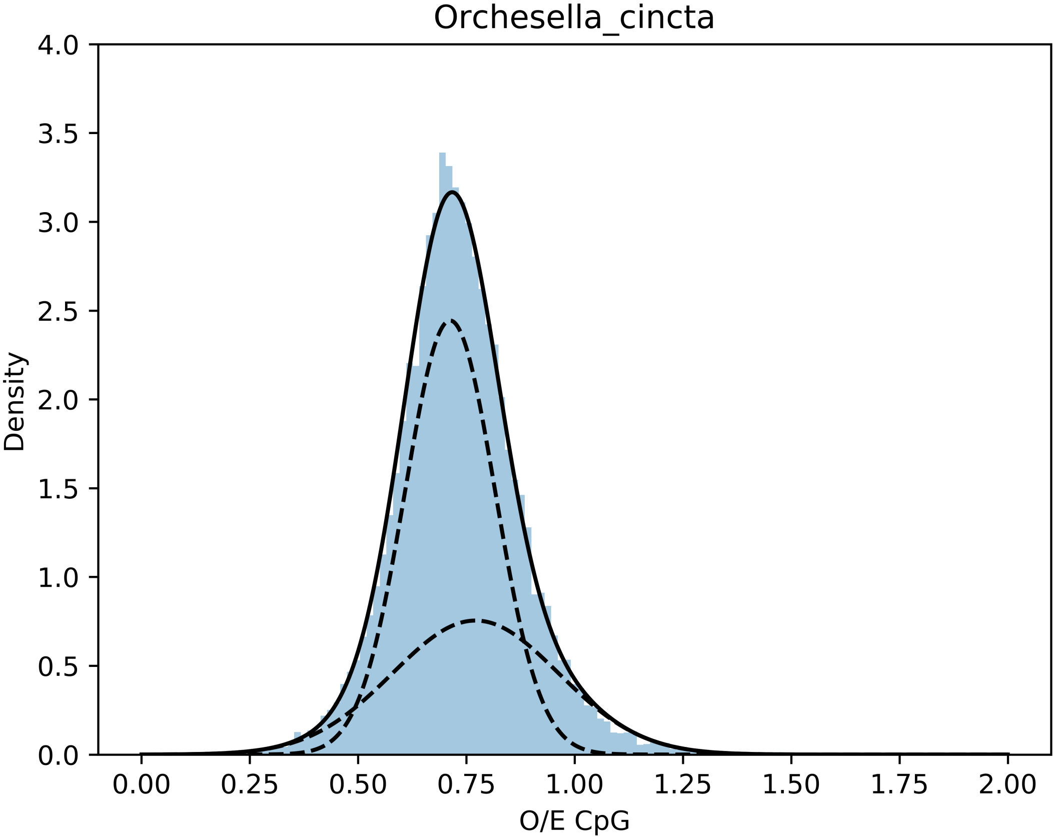
Orchesella_cincta
Density
O/E CpG
0.0
0.5
1.0
1.5
2.0
2.5
3.0
3.5
4.0
0.00
0.25
0.50
0.75
1.00
1.25
1.50
1.75
2.00

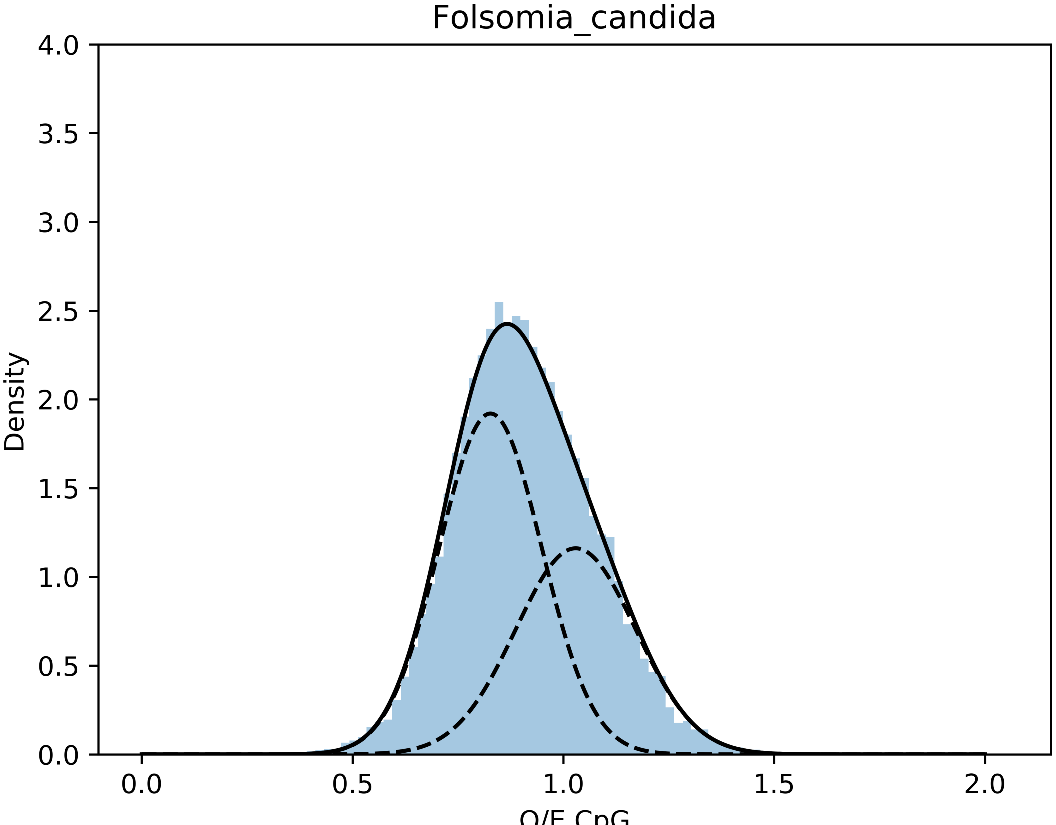

Folsomia_candida
Density
O/E CpG
0.0
0.5
1.0
1.5
2.0
2.5
3.0
3.5
4.0
0.0
0.5
1.0
1.5
2.0

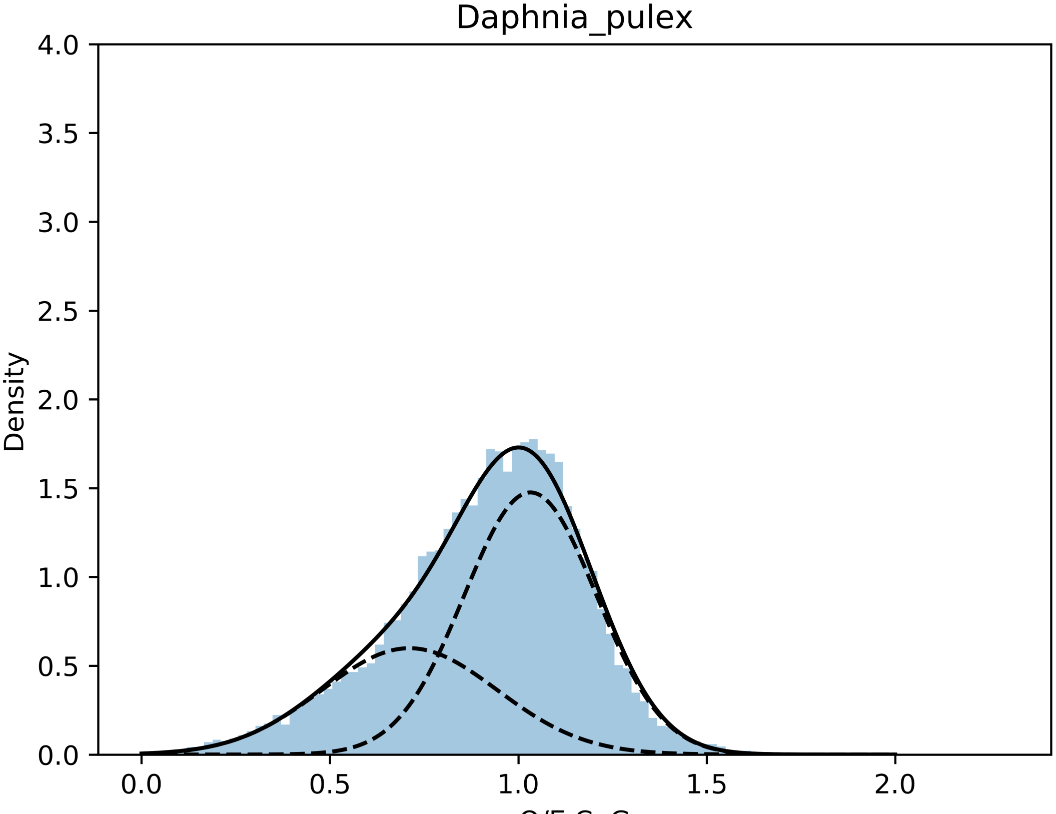

Daphnia_pulex
Density
O/E CpG
0.0
0.5
1.0
1.5
2.0
2.5
3.0
3.5
4.0
0.0
0.5
1.0
1.5
2.0

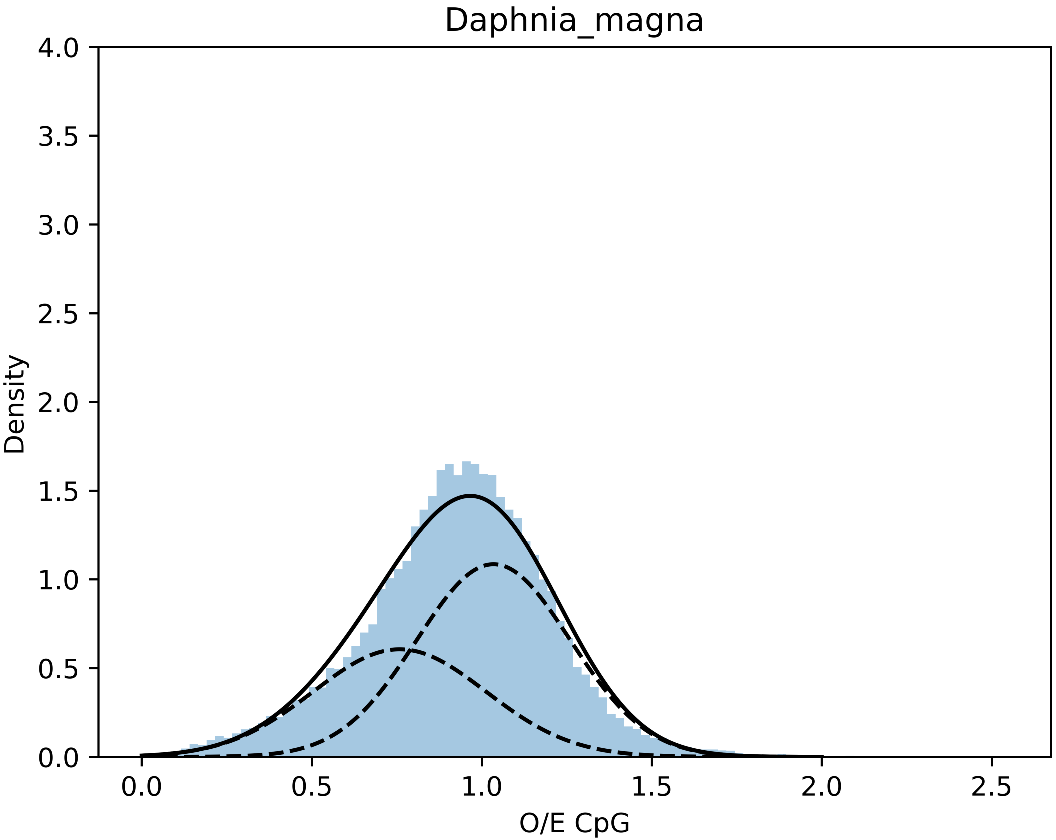

Daphnia_magna
Density
O/E CpG
0.0
0.5
1.0
1.5
2.0
2.5
3.0
3.5
4.0
0.0
0.5
1.0
1.5
2.0
2.5

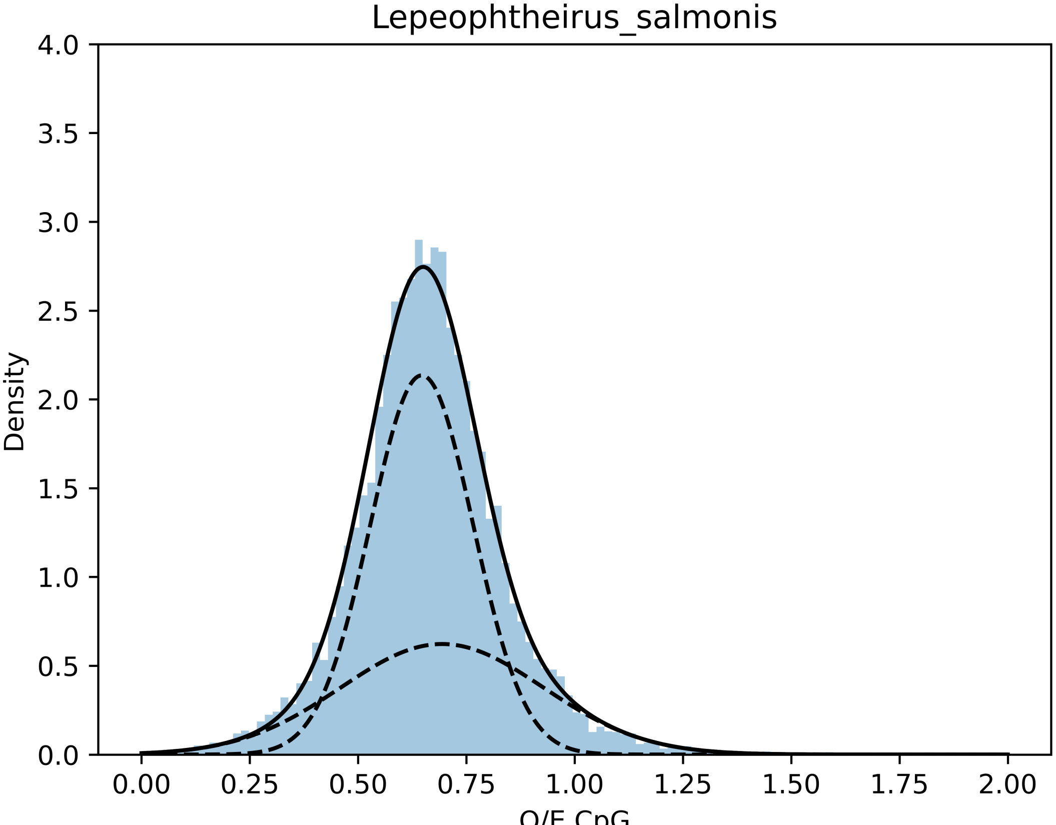
Lepeophtheirus_salmonis
Density
O/E CpG
0.0
0.5
1.0
1.5
2.0
2.5
3.0
3.5
4.0
0.00
0.25
0.50
0.75
1.00
1.25
1.50
1.75
2.00

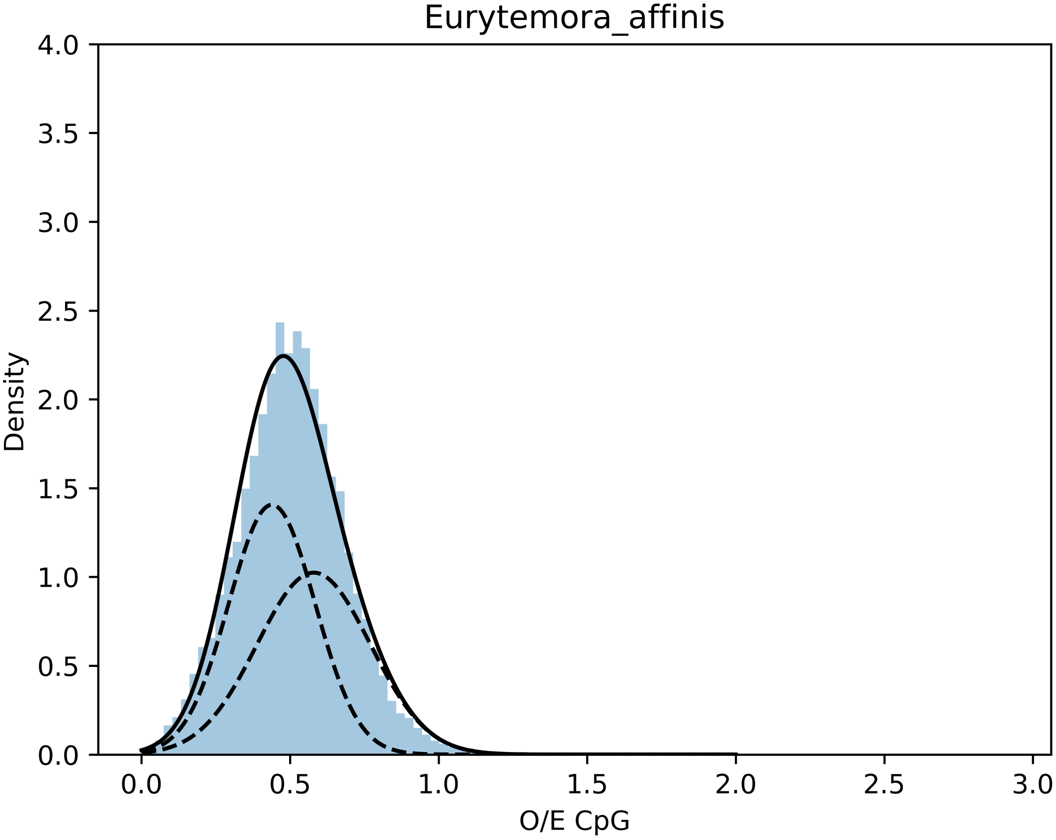
Eurytemora_affinis
Density
O/E CpG
0.0
0.5
1.0
1.5
2.0
2.5
3.0
3.5
4.0

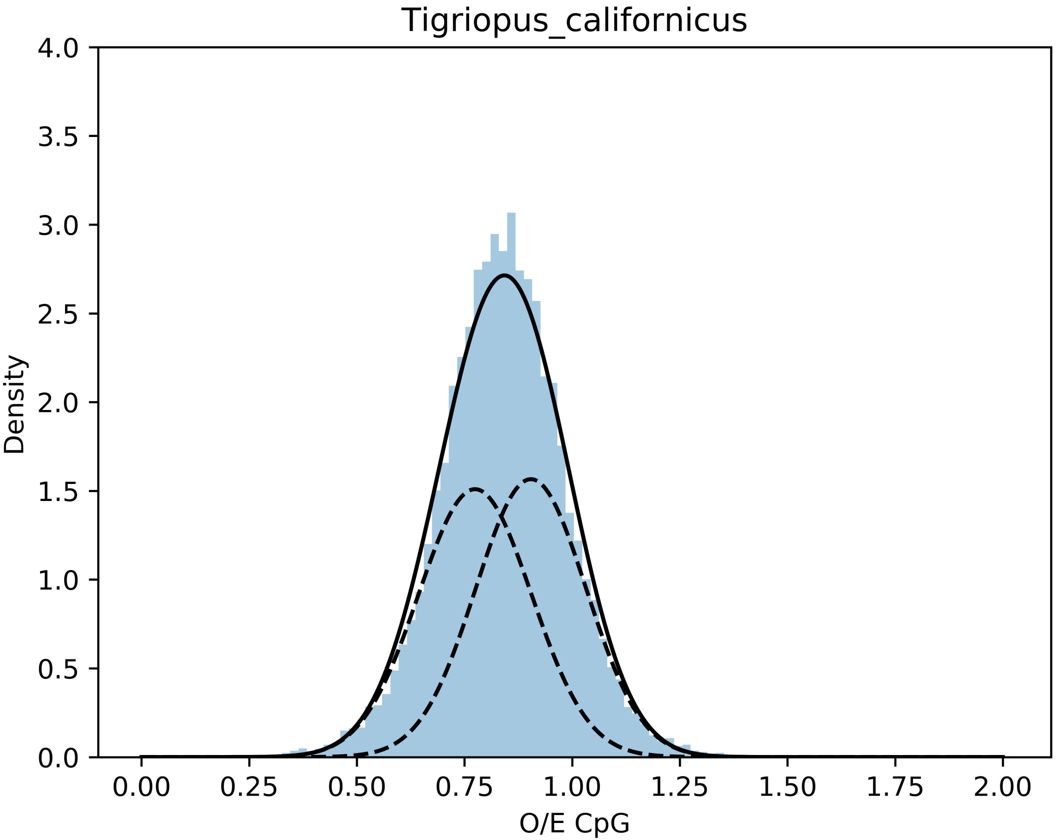

Tigriopus_californicus
Density
O/E CpG
0.0
0.5
1.0
1.5
2.0
2.5
3.0
3.5
4.0
0.00
0.25
0.50
0.75
1.00
1.25
1.50
1.75
2.00

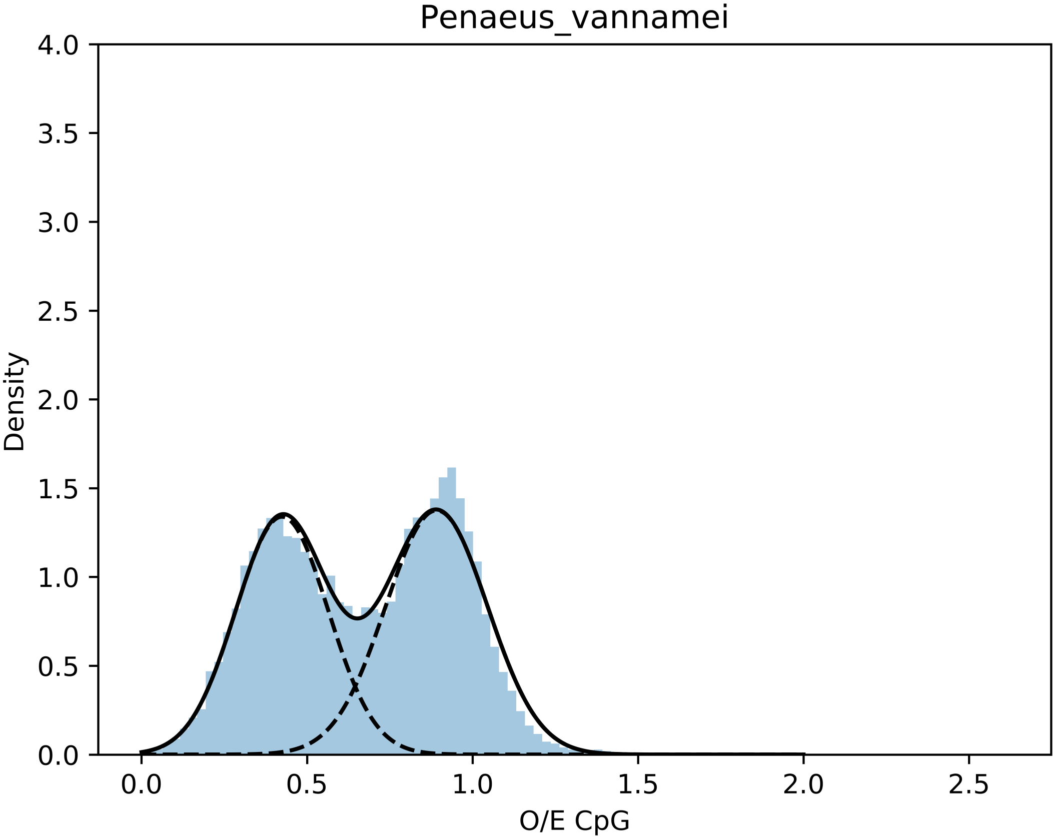

Penaeus_vannamei
Density
O/E CpG
4.0
3.5
3.0
2.5
2.0
1.5
1.0
0.5
0.0
0.0
0.5
1.0
1.5
2.0
2.5

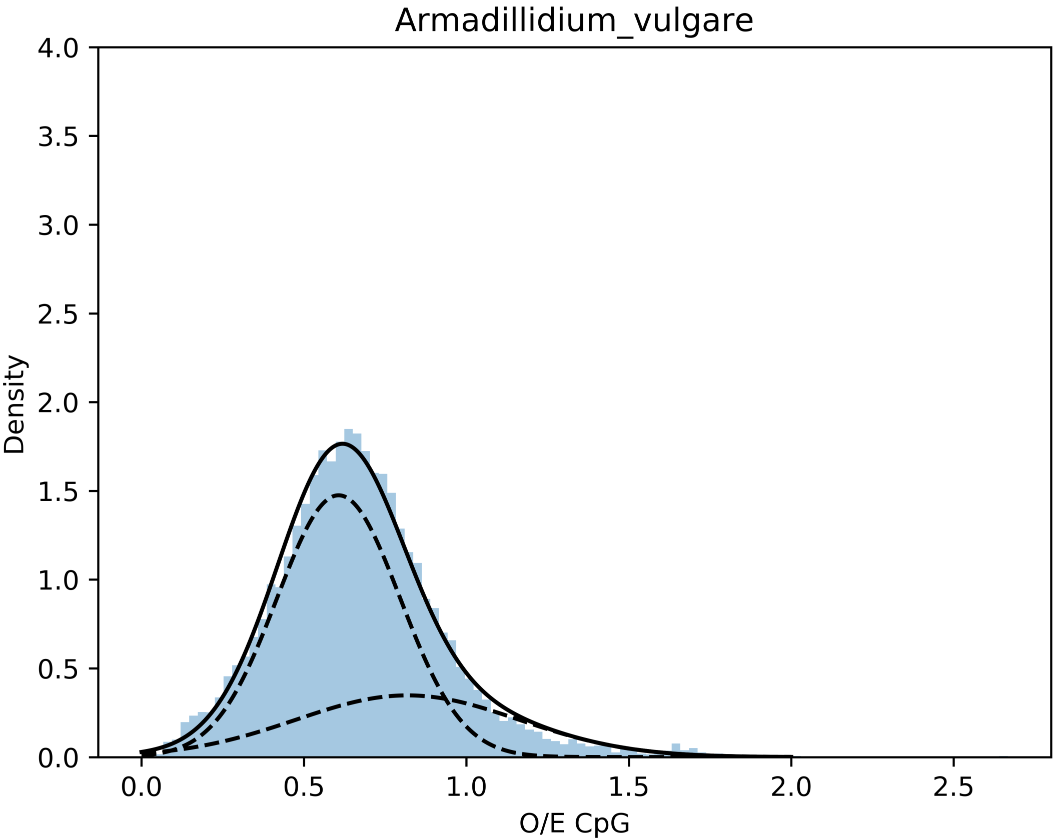
Armadillidium_vulgare
Density
O/E CpG
0.0
0.5
1.0
1.5
2.0
2.5
3.0
3.5
4.0
0.0
0.5
1.0
1.5
2.0
2.5

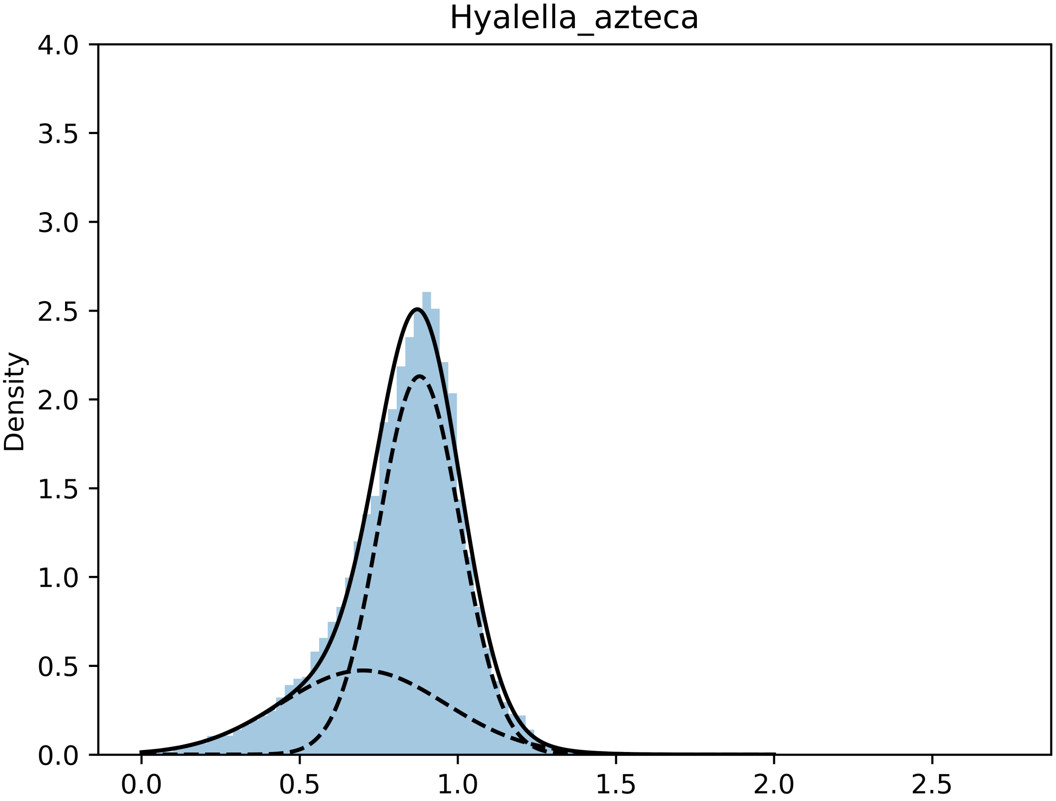
Hyalella_azteca
Density
O/E CpG
4.0
3.5
3.0
2.5
2.0
1.5
1.0
0.5
0.0
0.0
0.5
1.0
1.5
2.0
2.5

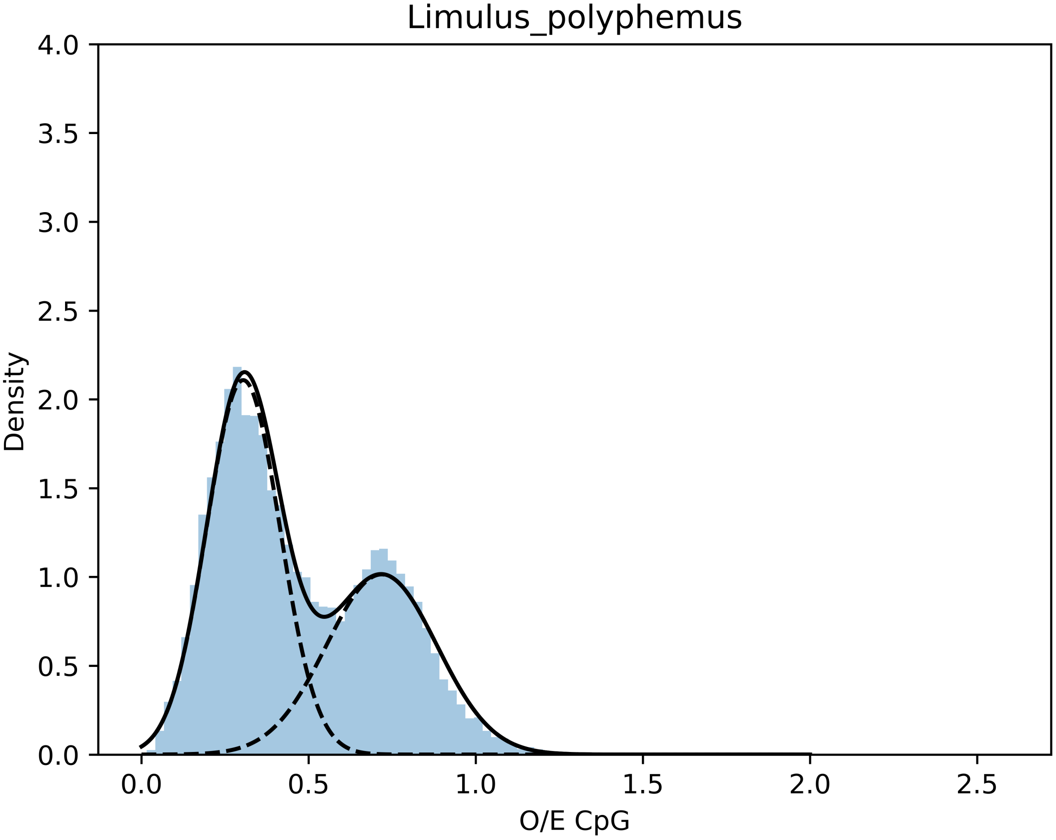
Limulus_polyphemus
Density
O/E CpG
0.0
0.5
1.0
1.5
2.0
2.5
3.0
3.5
4.0
0.0
0.5
1.0
1.5
2.0
2.5

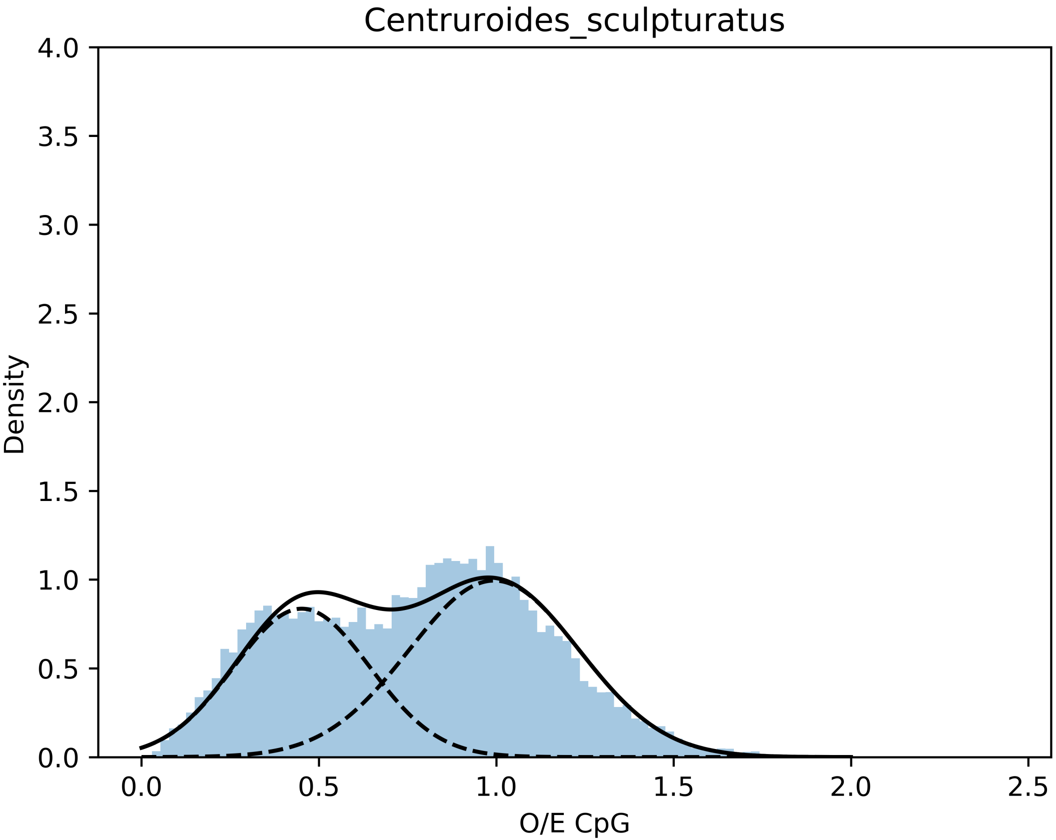

Centruroides_sculpturatus
Density
O/E CpG
4.0
3.5
3.0
2.5
2.0
1.5
1.0
0.5
0.0
0.0
0.5
1.0
1.5
2.0
2.5

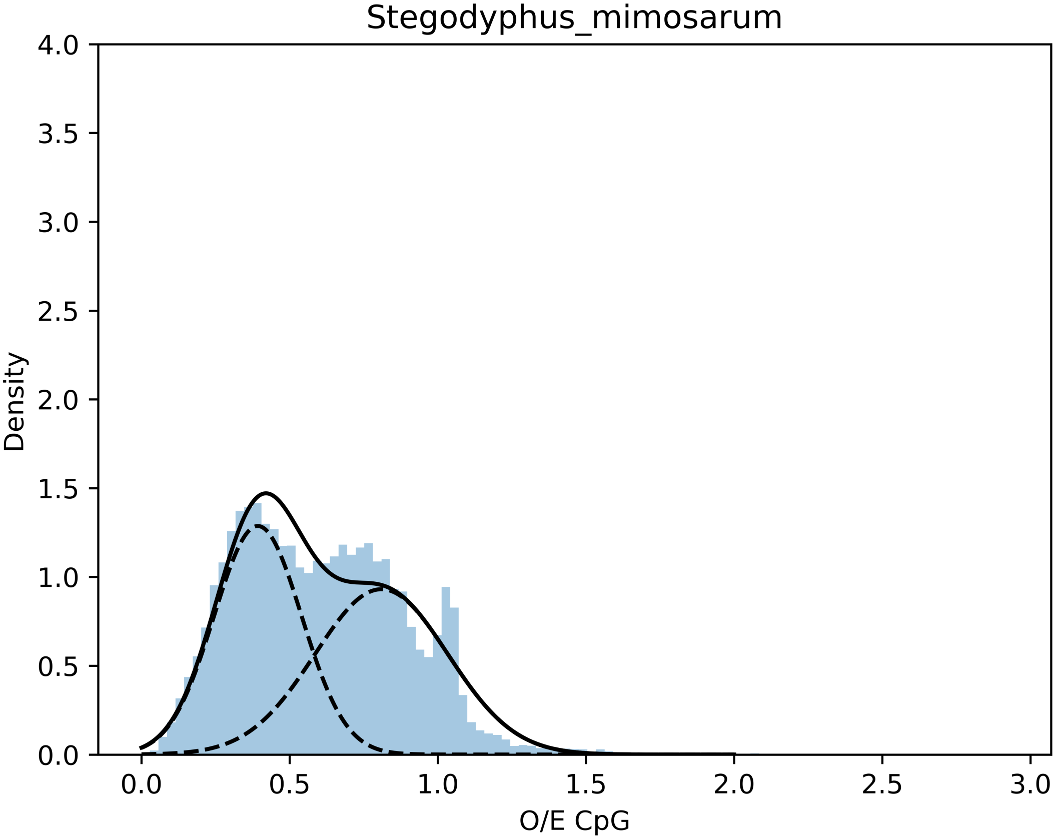

Stegodyphus_mimosarum
Density
O/E CpG
0.0
0.5
1.0
1.5
2.0
2.5
3.0
3.5
4.0

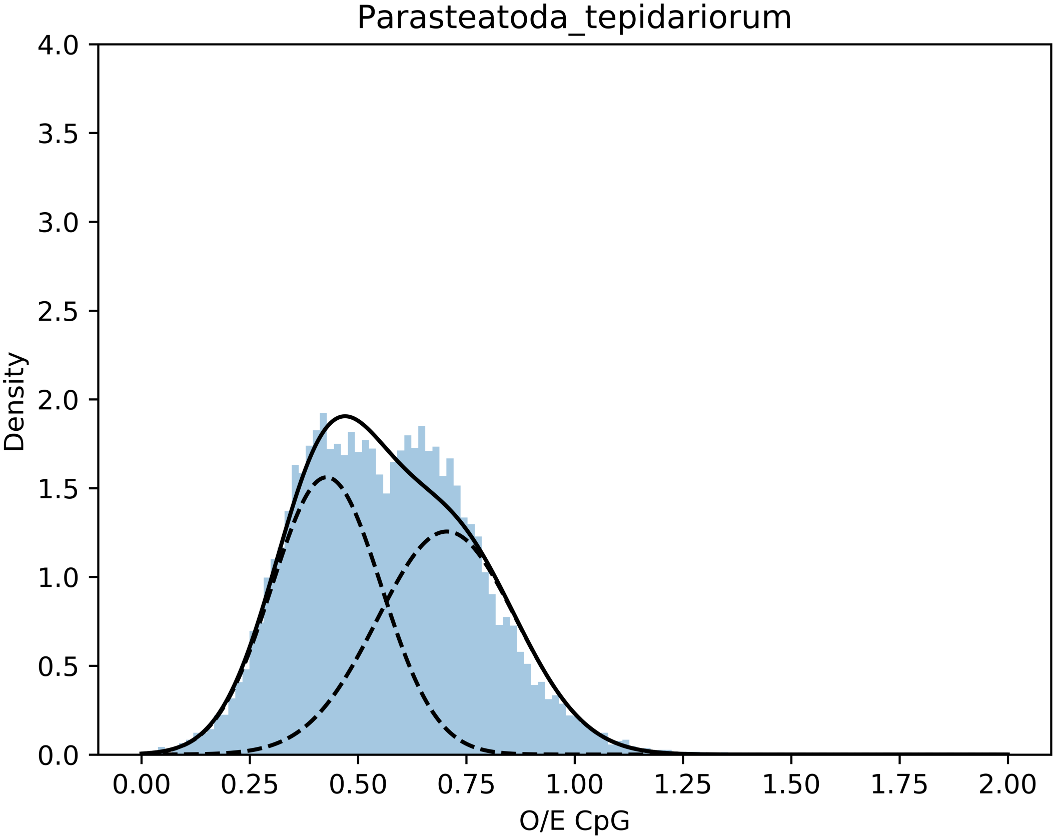

Parasteatoda_tepidariorum
Density
O/E CpG
0.0
0.5
1.0
1.5
2.0
2.5
3.0
3.5
4.0
0.00
0.25
0.50
0.75
1.00
1.25
1.50
1.75
2.00

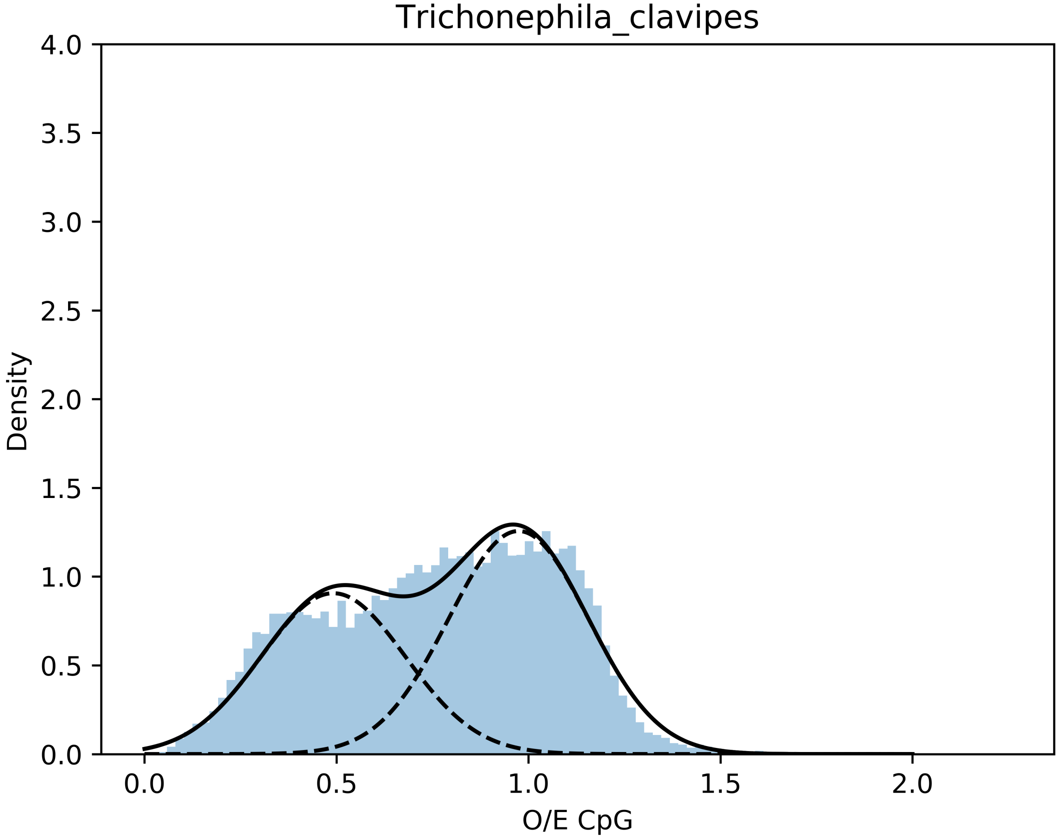
Trichonephila_clavipes
4.0
3.5
3.0
2.5
2.0
1.5
1.0
0.5
0.0
Density
0.0
0.5
1.0
1.5
2.0
O/E CpG

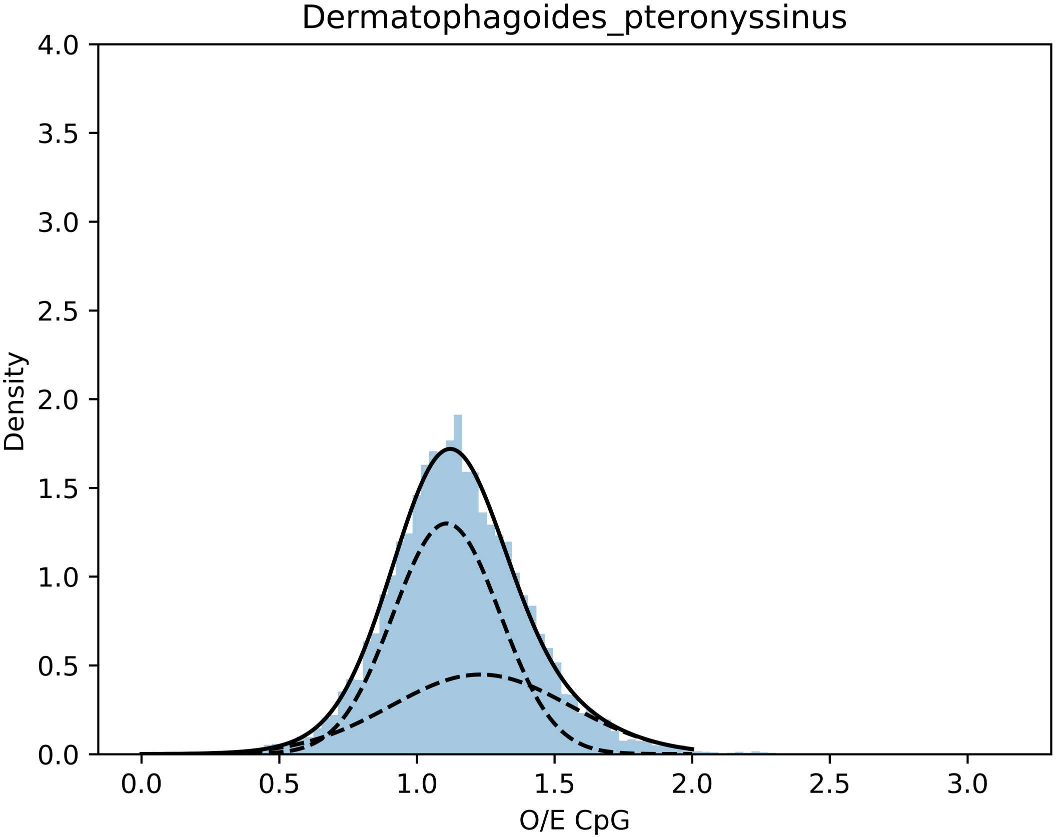

Dermatophagoides_pteronyssinus
Density
O/E CpG
0.0
0.5
1.0
1.5
2.0
2.5
3.0
3.5
4.0

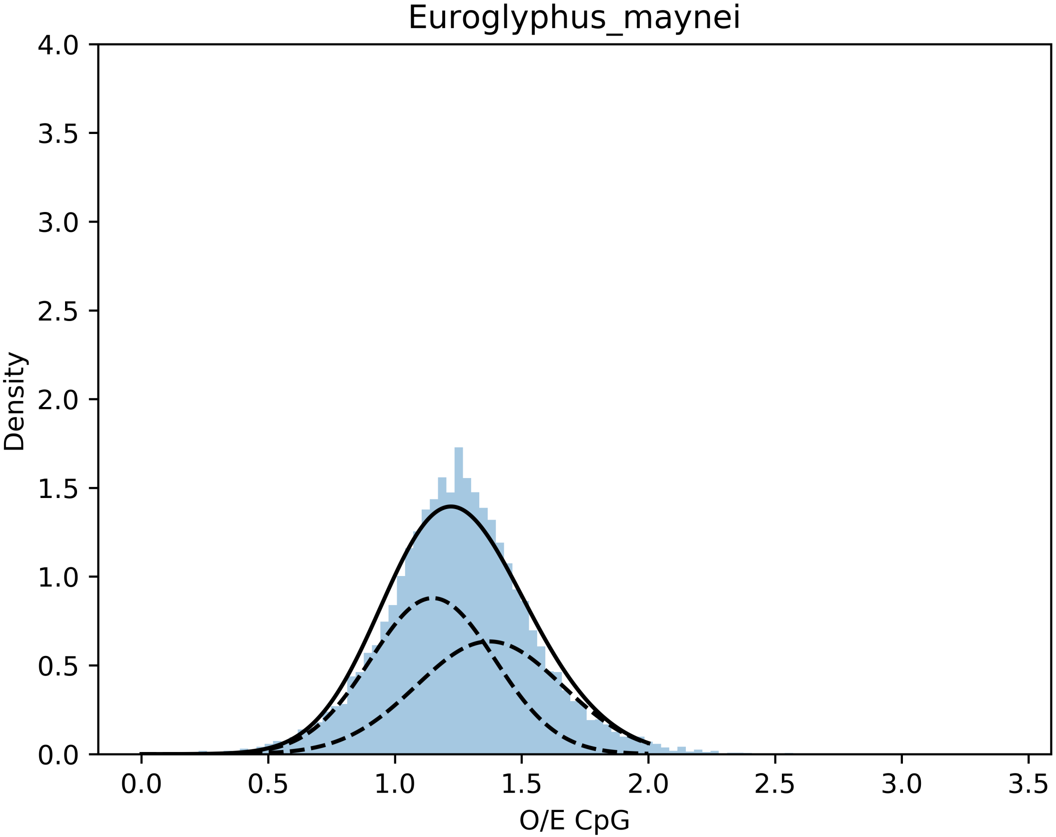
Euroglyphus_maynei
Density
O/E CpG
0.0
0.5
1.0
1.5
2.0
2.5
3.0
3.5
4.0

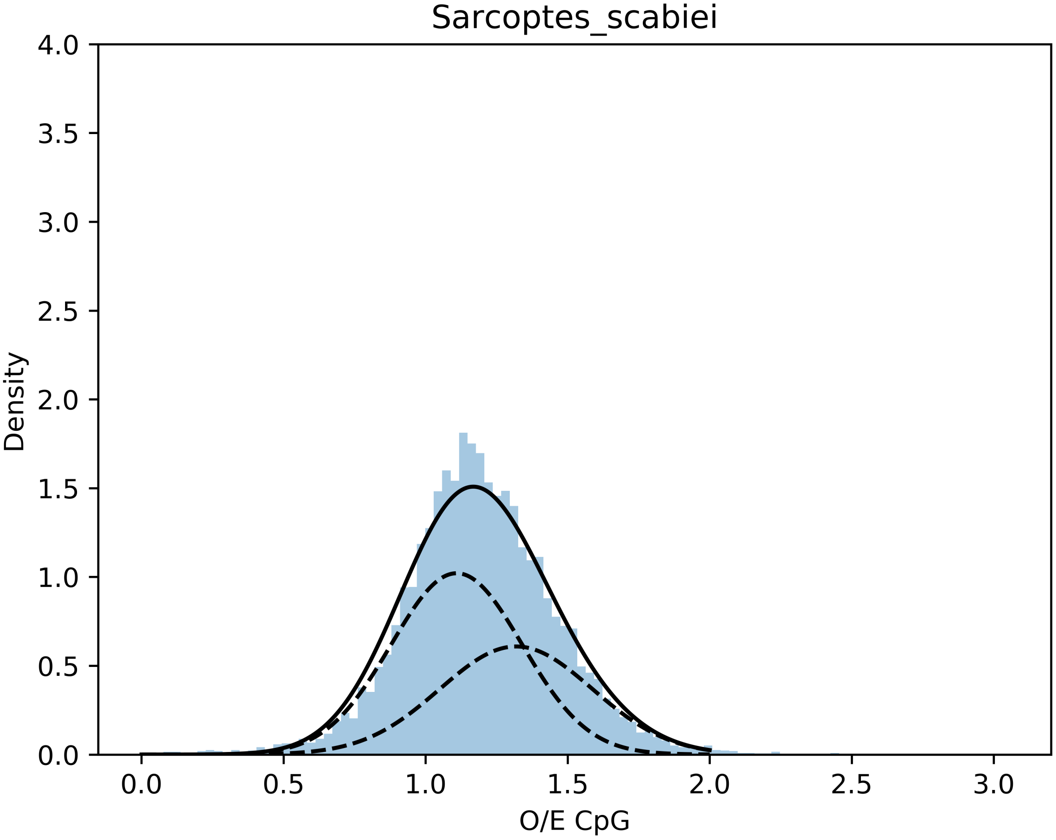

Sarcoptes_scabiei
Density
O/E CpG
0.0
0.5
1.0
1.5
2.0
2.5
3.0
3.5
4.0
0.0
0.5
1.0
1.5
2.0
2.5
3.0

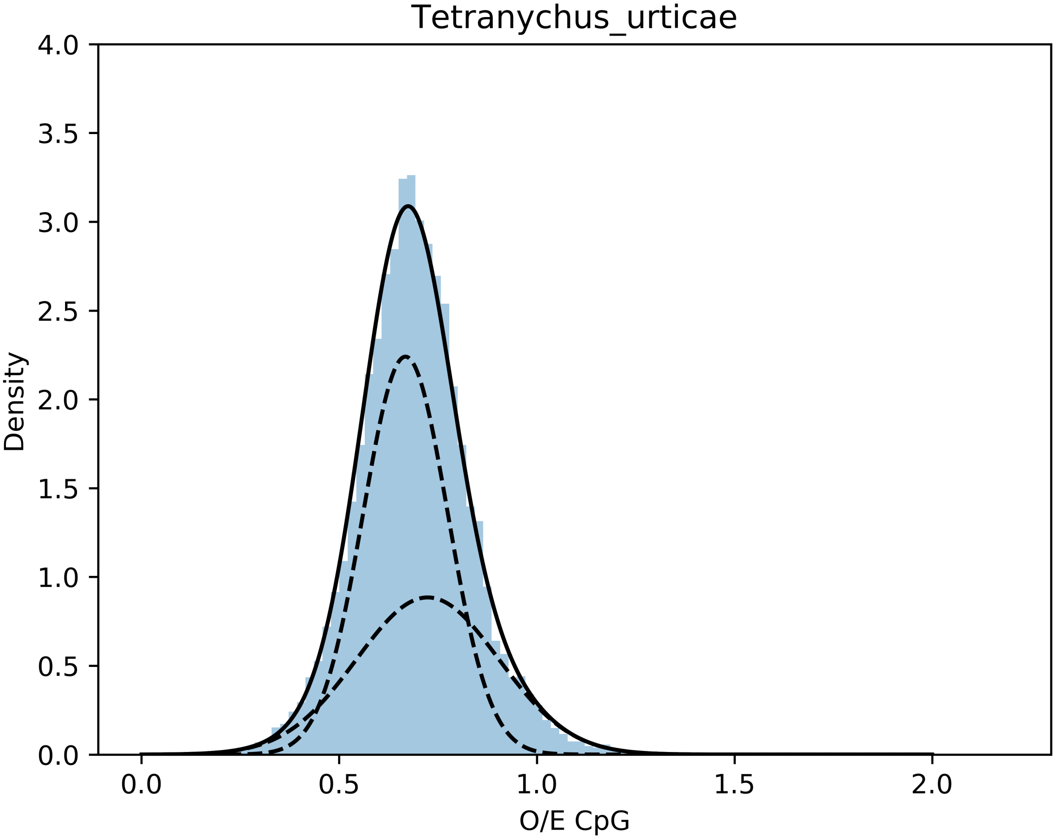
Tetranychus_urticae
Density
O/E CpG
4.0
3.5
3.0
2.5
2.0
1.5
1.0
0.5
0.0
0.0
0.5
1.0
1.5
2.0

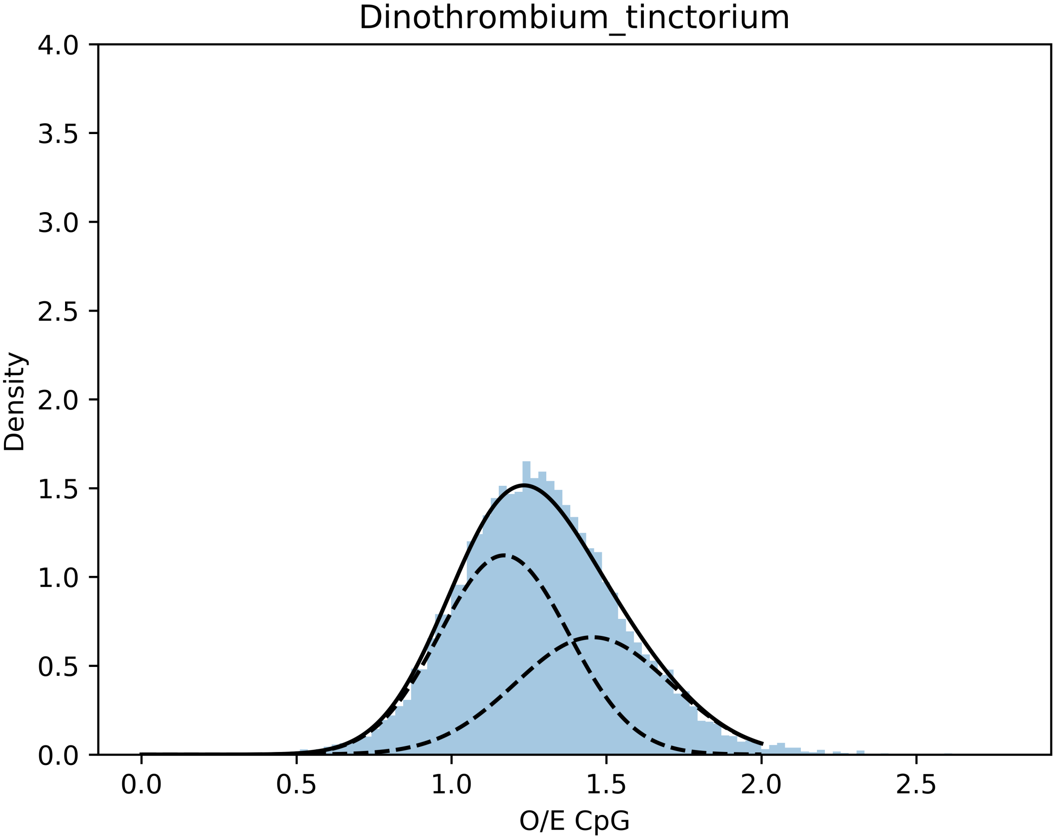

Dinothrombium_tinctorium
Density
4.0
3.5
3.0
2.5
2.0
1.5
1.0
0.5
0.0
0.0
0.5
1.0
1.5
2.0
2.5
O/E CpG

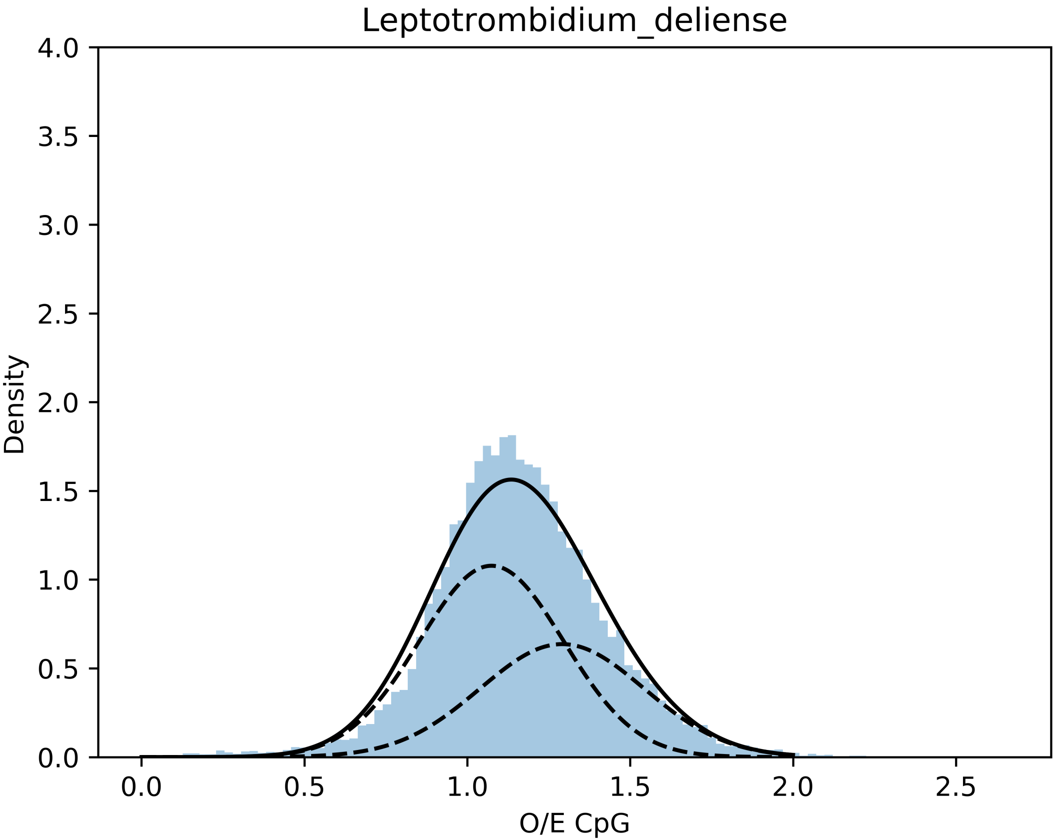
Leptotrombidium_deliense
Density
O/E CpG
0.0
0.5
1.0
1.5
2.0
2.5
3.0
3.5
4.0
0.0
0.5
1.0
1.5
2.0
2.5

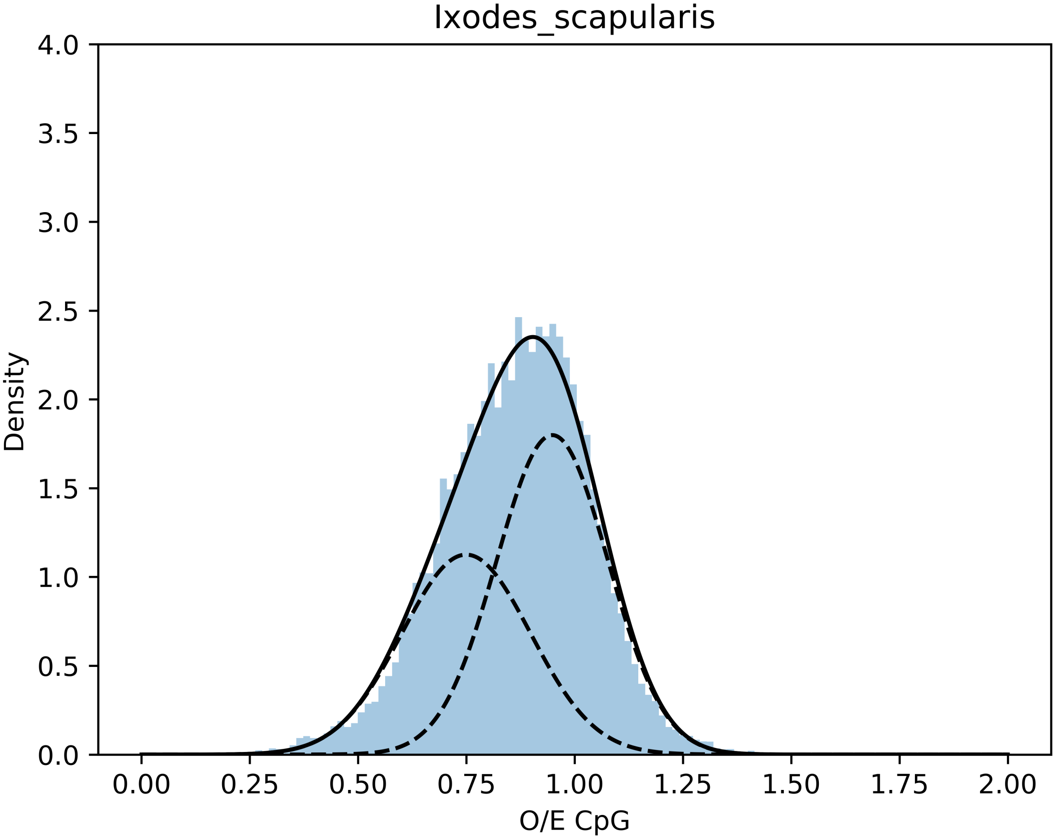
Ixodes_scapularis
Density
4.0
3.5
3.0
2.5
2.0
1.5
1.0
0.5
0.0
0.00
0.25
0.50
0.75
1.00
1.25
1.50
1.75
2.00
O/E CpG

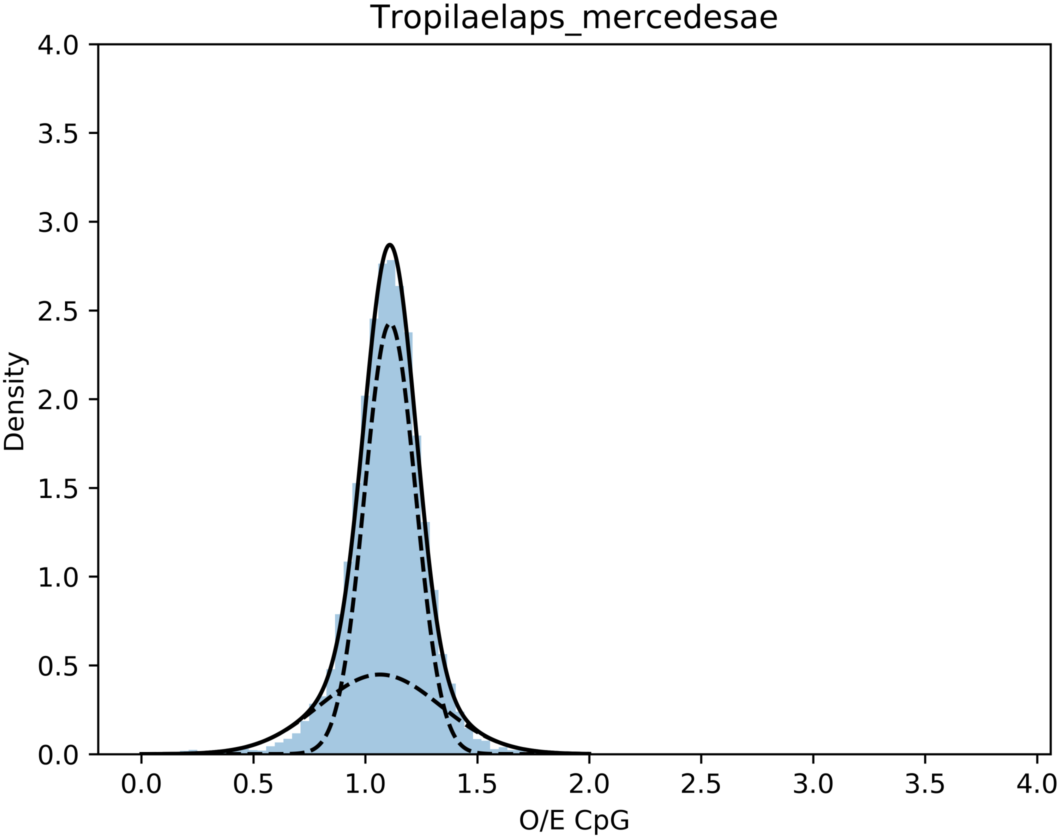
Tropilaelaps_mercedesae
Density
O/E CpG
0.0
0.5
1.0
1.5
2.0
2.5
3.0
3.5
4.0

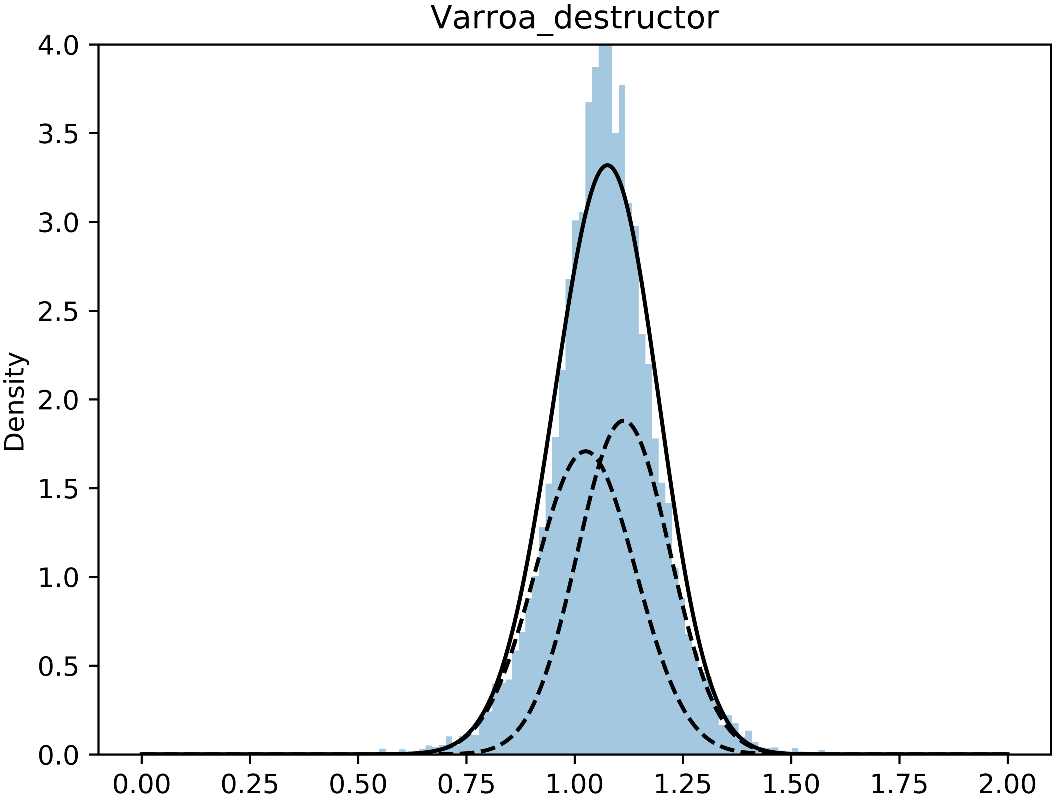
Varroa_destructor
Density
4.0
3.5
3.0
2.5
2.0
1.5
1.0
0.5
0.0
0.00
0.25
0.50
0.75
1.00
1.25
1.50
1.75
2.00
O/E CpG

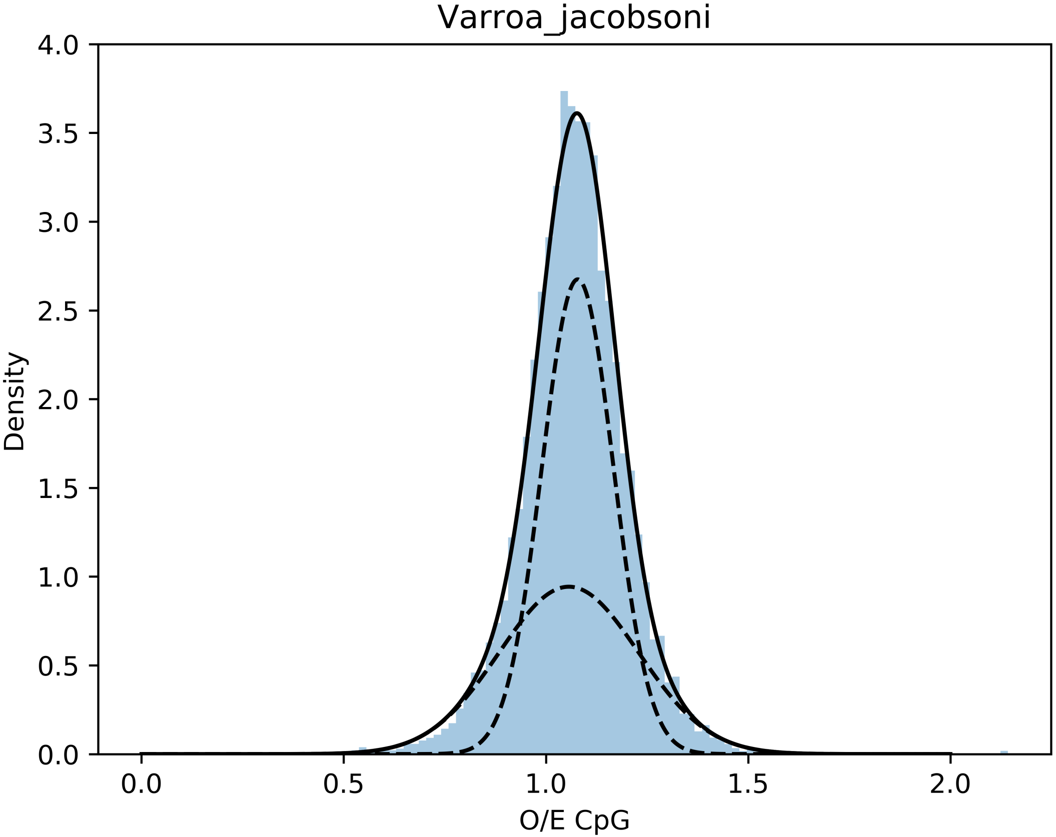

Varroa_jacobsoni
Density
O/E CpG
4.0
3.5
3.0
2.5
2.0
1.5
1.0
0.5
0.0
0.0
0.5
1.0
1.5
2.0

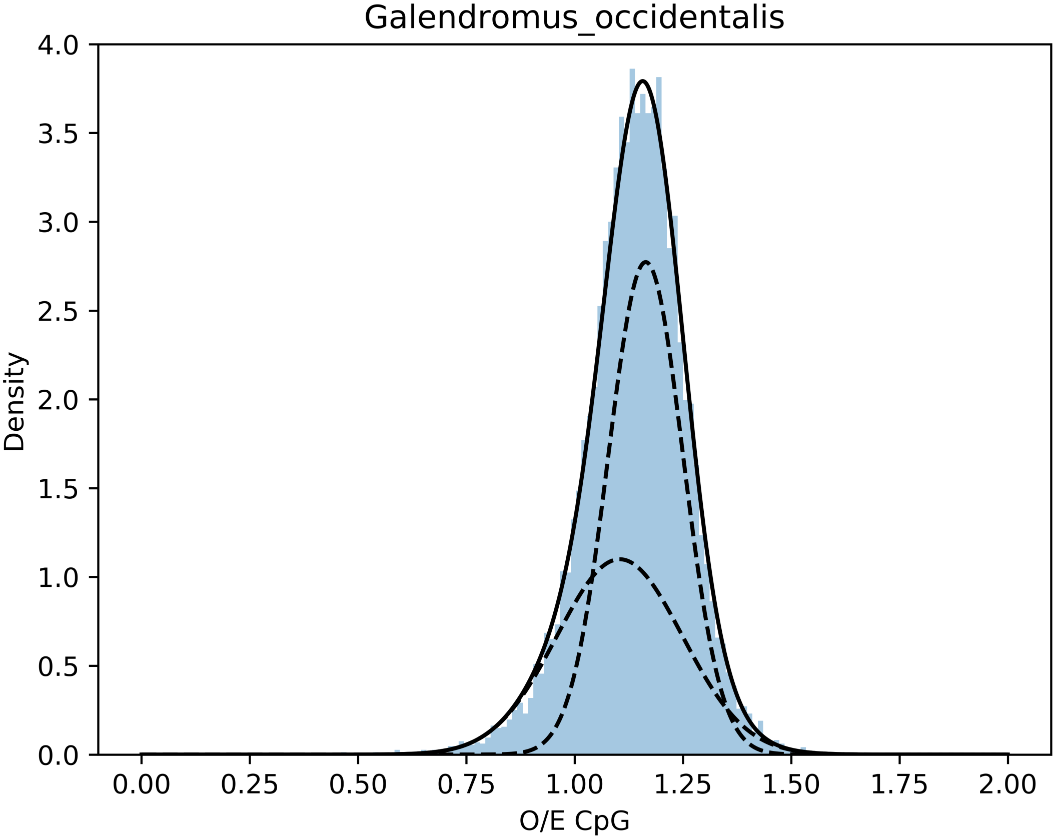

Galendromus_occidentalis
Density
O/E CpG
0.0
0.5
1.0
1.5
2.0
2.5
3.0
3.5
4.0
0.00
0.25
0.50
0.75
1.00
1.25
1.50
1.75
2.00

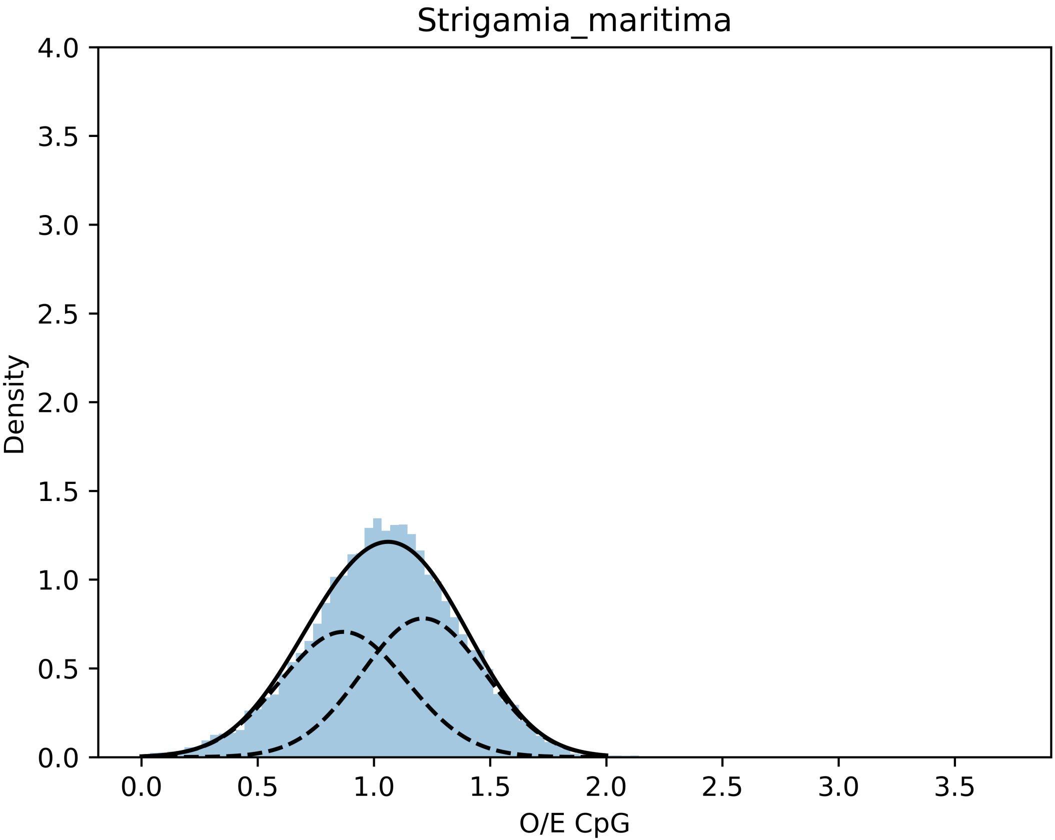

Strigamia_maritima
Density
O/E CpG
0.0
0.5
1.0
1.5
2.0
2.5
3.0
3.5
4.0

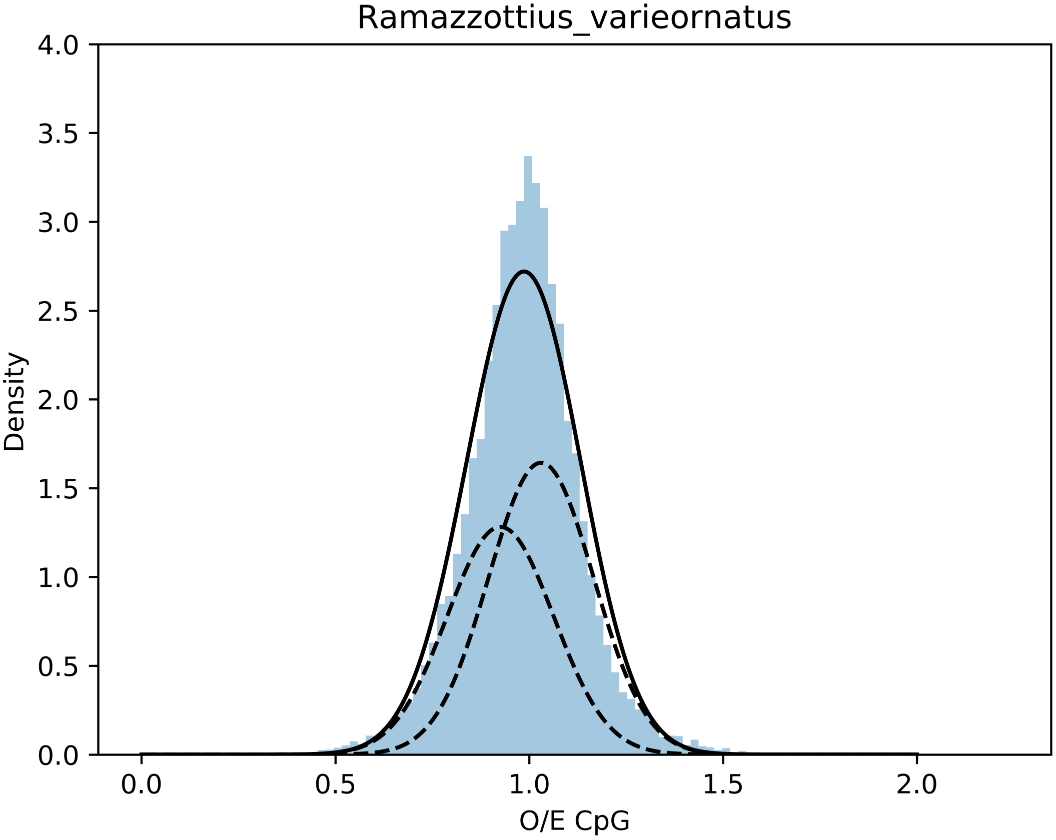
Ramazzottius_varieornatus
Density
O/E CpG
4.0
3.5
3.0
2.5
2.0
1.5
1.0
0.5
0.0
0.0
0.5
1.0
1.5
2.0

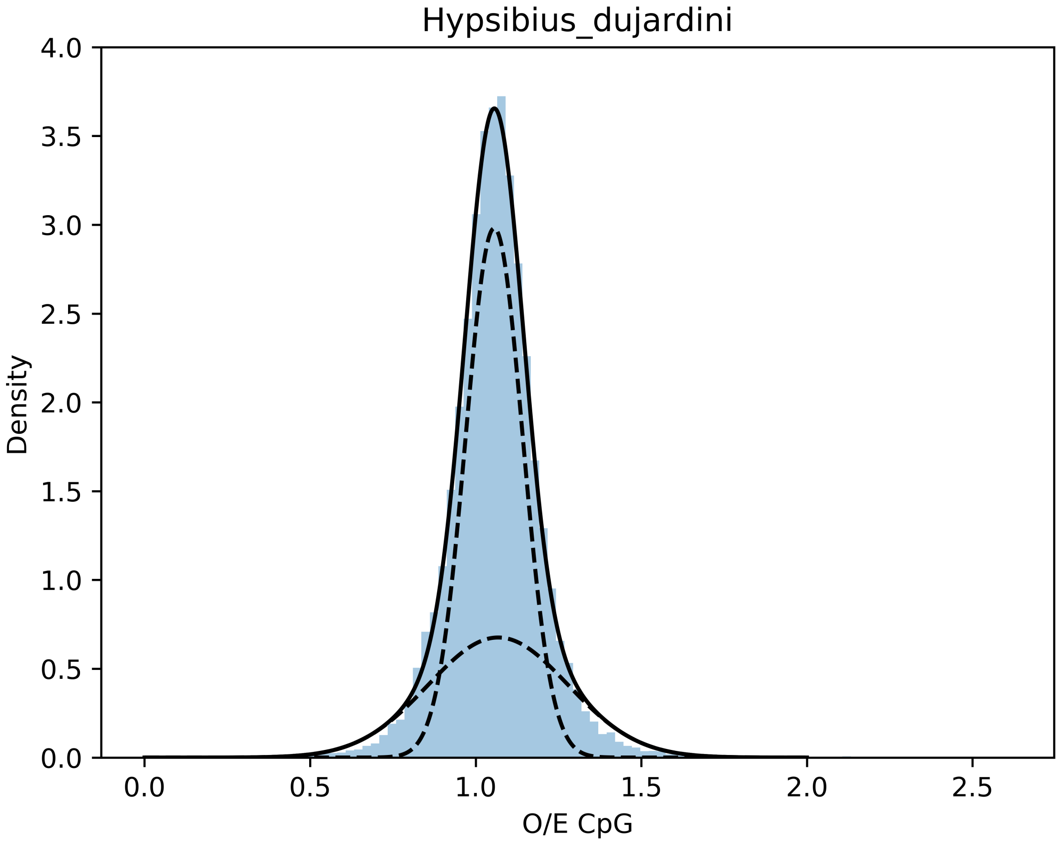
Hypsibius_dujardini
4.0
3.5
3.0
2.5
2.0
1.5
1.0
0.5
0.0
Density
0.0
0.5
1.0
1.5
2.0
2.5
O/E CpG

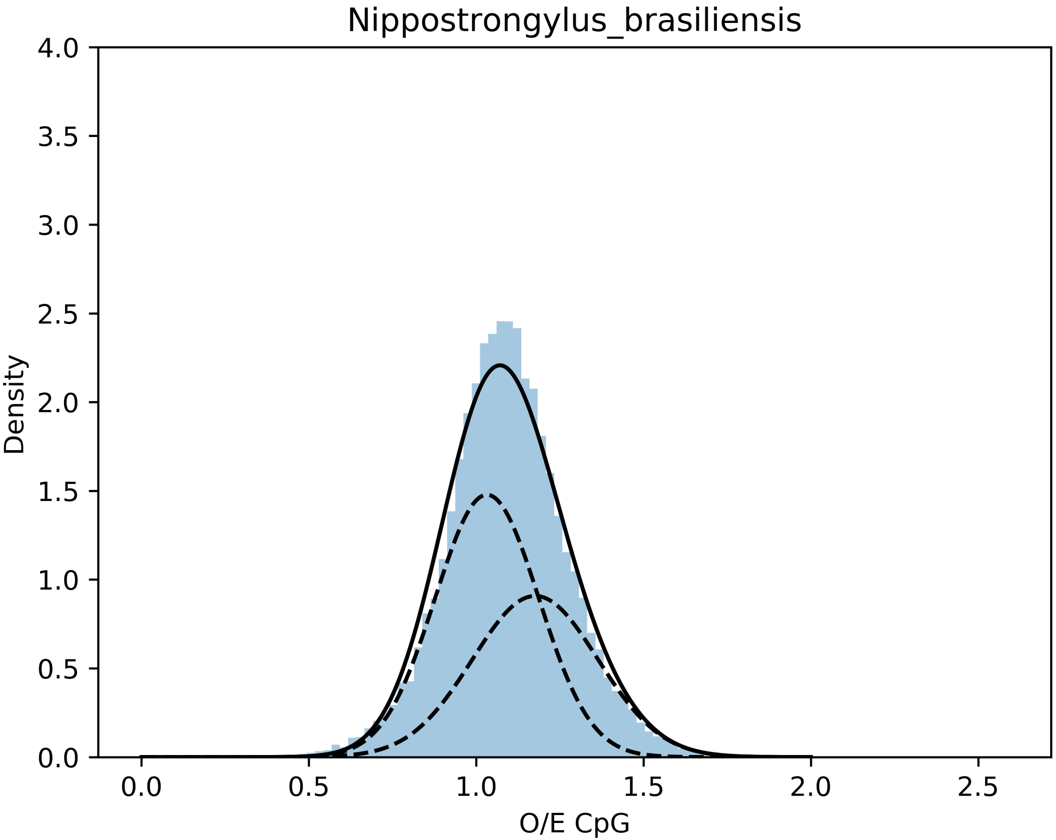
Nippostrongylus_brasiliensis
Density
O/E CpG
0.0
0.5
1.0
1.5
2.0
2.5
3.0
3.5
4.0
0.0
0.5
1.0
1.5
2.0
2.5

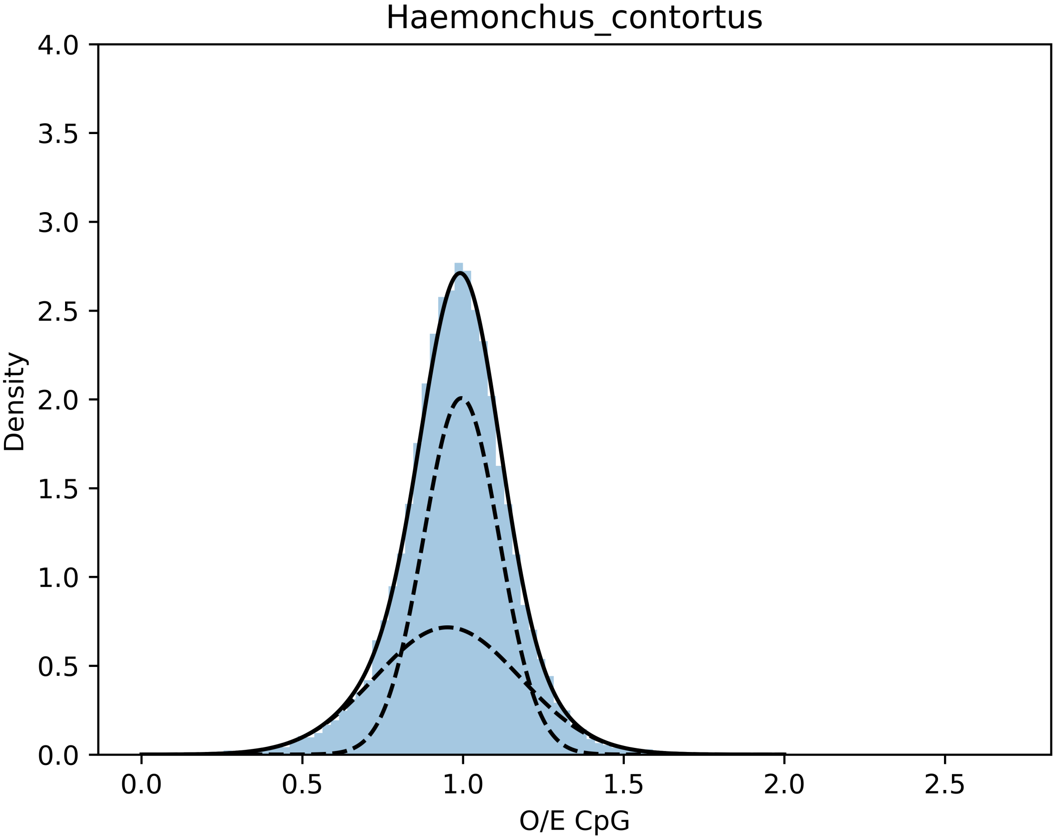
Haemonchus_contortus
Density
4.0
3.5
3.0
2.5
2.0
1.5
1.0
0.5
0.0
0.0
0.5
1.0
1.5
2.0
2.5
O/E CpG

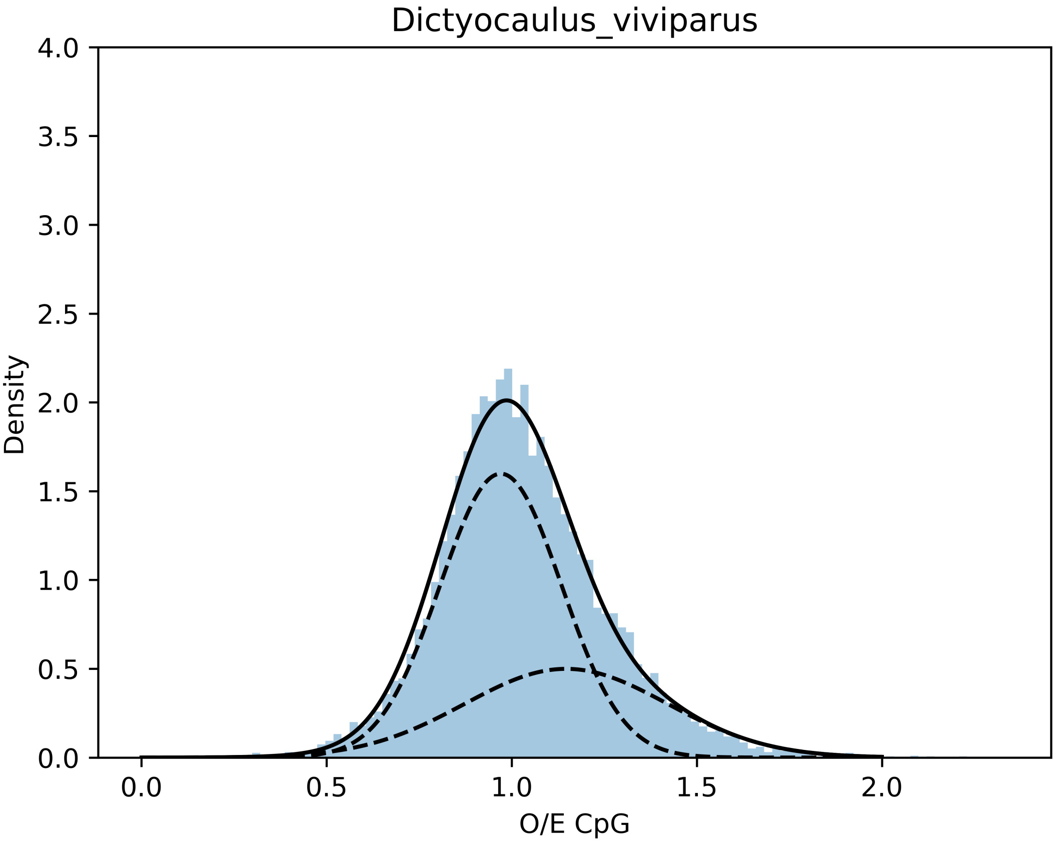
Dictyocaulus_viviparus
Density
O/E CpG
0.0
0.5
1.0
1.5
2.0
2.5
3.0
3.5
4.0
0.0
0.5
1.0
1.5
2.0

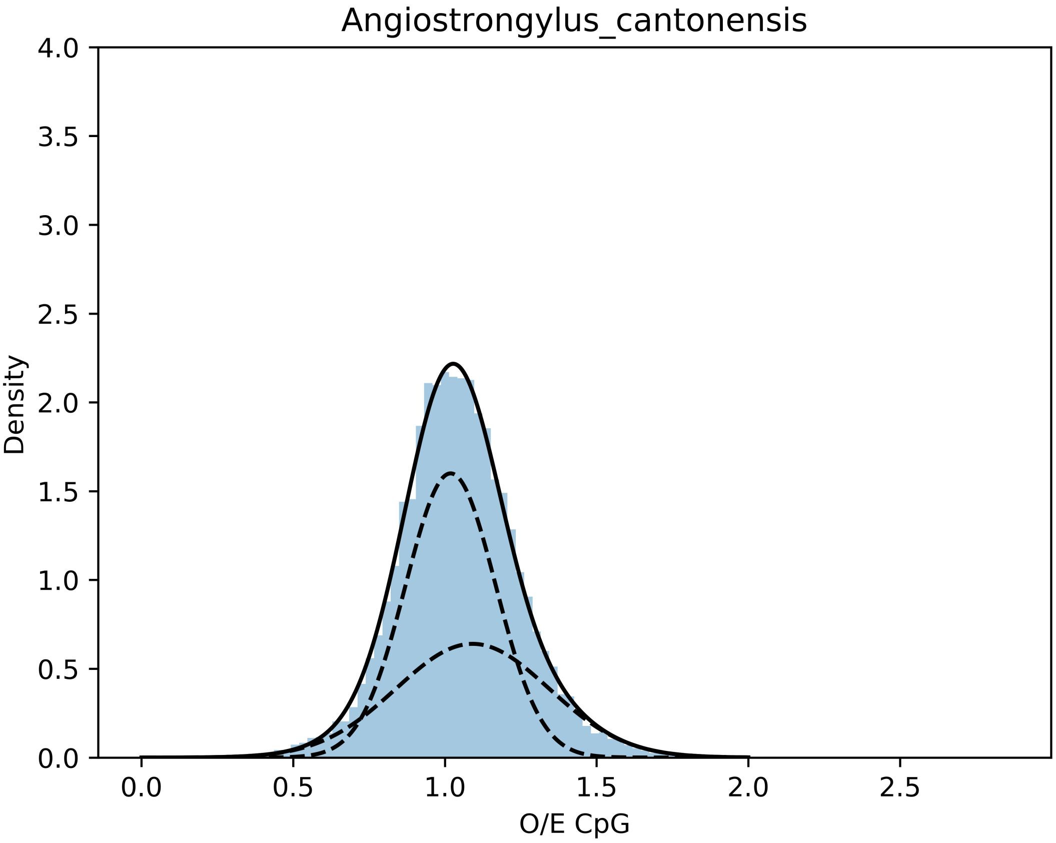
Angiostrongylus_cantonensis
Density
O/E CpG
0.0
0.5
1.0
1.5
2.0
2.5
3.0
3.5
4.0

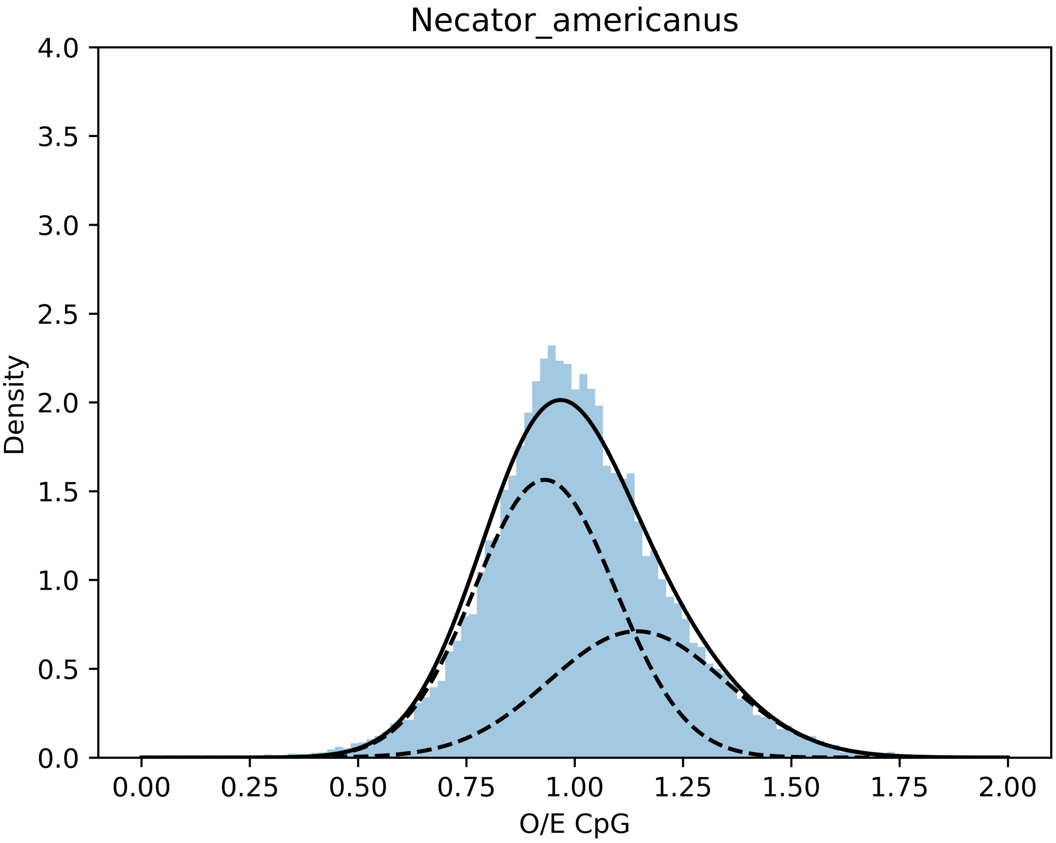

Necator_americanus
Density
O/E CpG
0.0
0.5
1.0
1.5
2.0
2.5
3.0
3.5
4.0
0.00
0.25
0.50
0.75
1.00
1.25
1.50
1.75
2.00

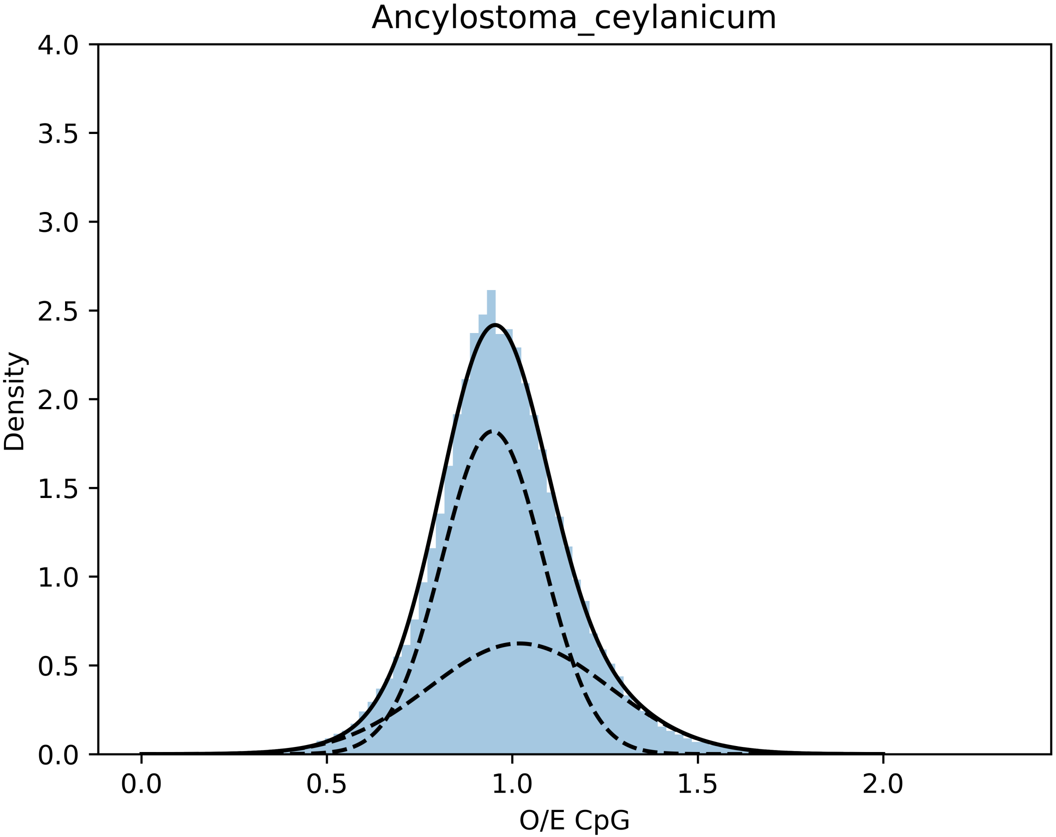

Ancylostoma_ceylanicum
Density
O/E CpG
4.0
3.5
3.0
2.5
2.0
1.5
1.0
0.5
0.0
0.0
0.5
1.0
1.5
2.0

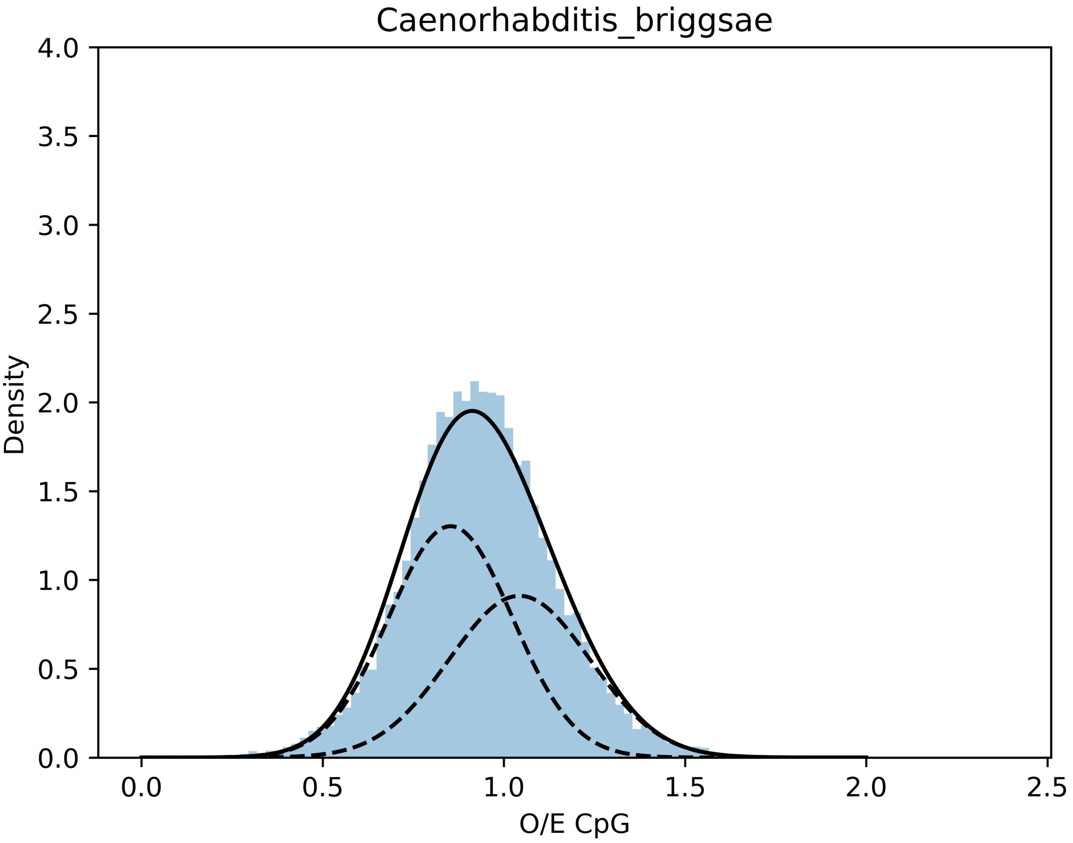

Caenorhabditis_briggsae
Density
O/E CpG
4.0
3.5
3.0
2.5
2.0
1.5
1.0
0.5
0.0
0.0
0.5
1.0
1.5
2.0
2.5

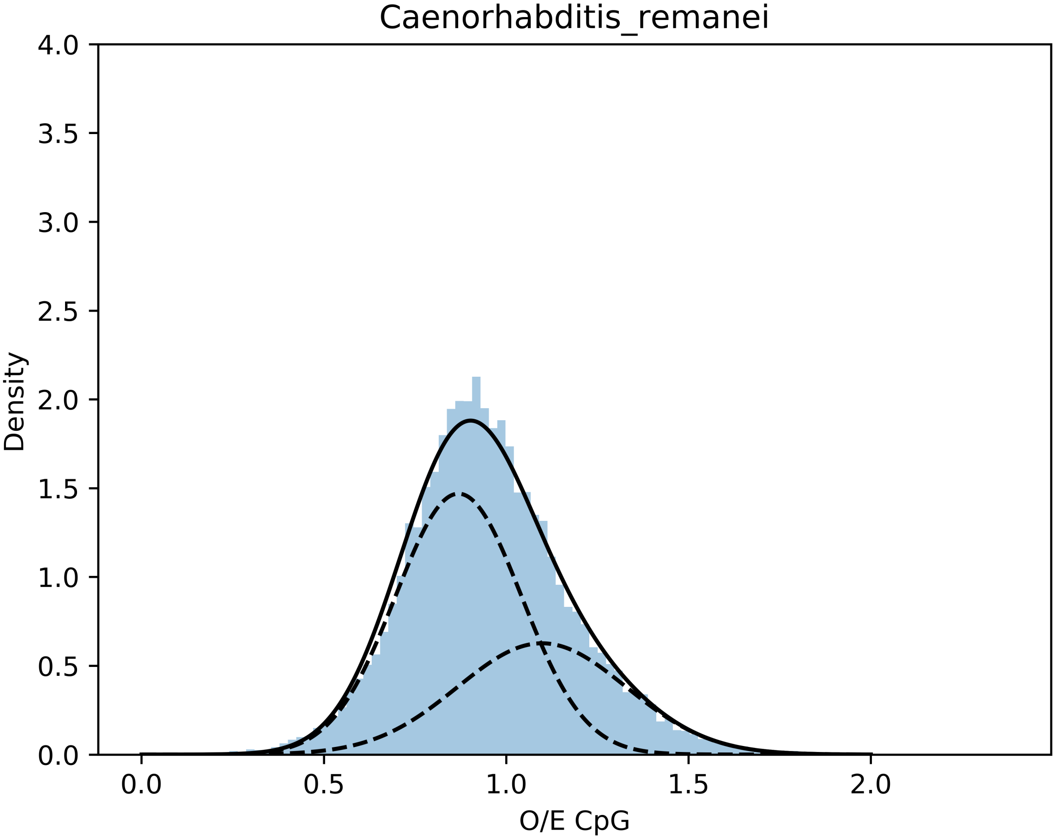
Caenorhabditis_remanei
Density
O/E CpG
4.0
3.5
3.0
2.5
2.0
1.5
1.0
0.5
0.0
0.0
0.5
1.0
1.5
2.0

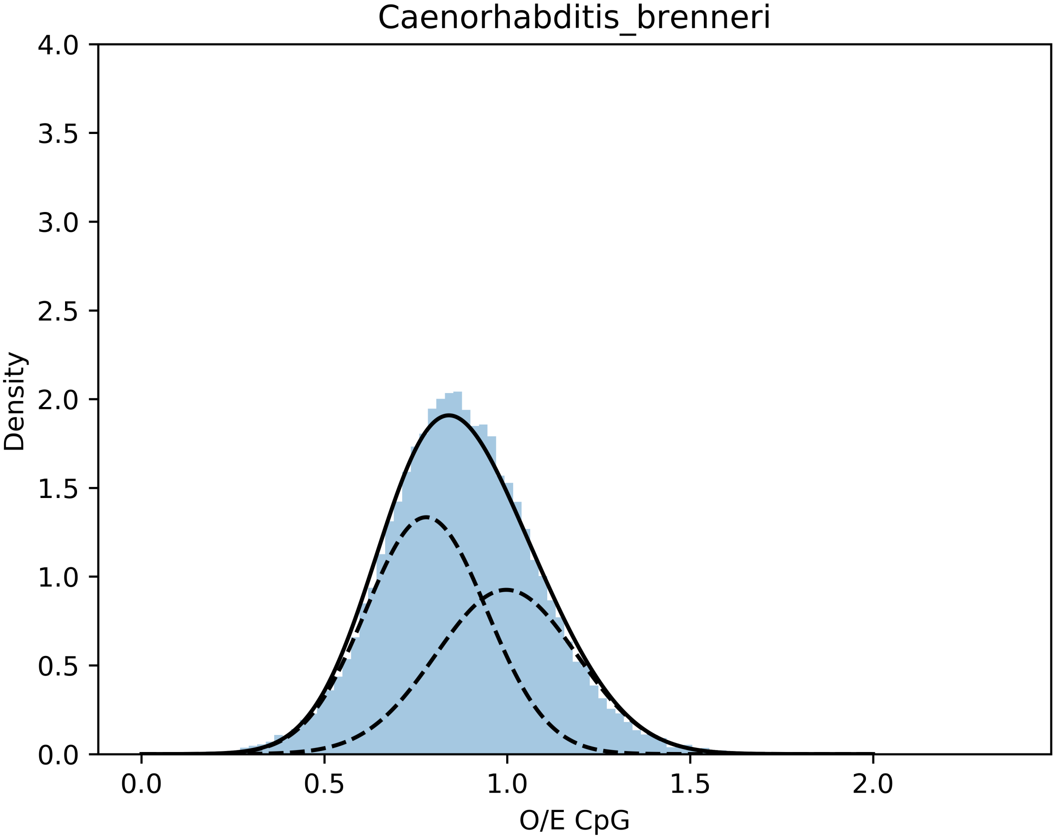
Caenorhabditis_brenneri
Density
O/E CpG
0.0
0.5
1.0
1.5
2.0
2.5
3.0
3.5
4.0
0.0
0.5
1.0
1.5
2.0

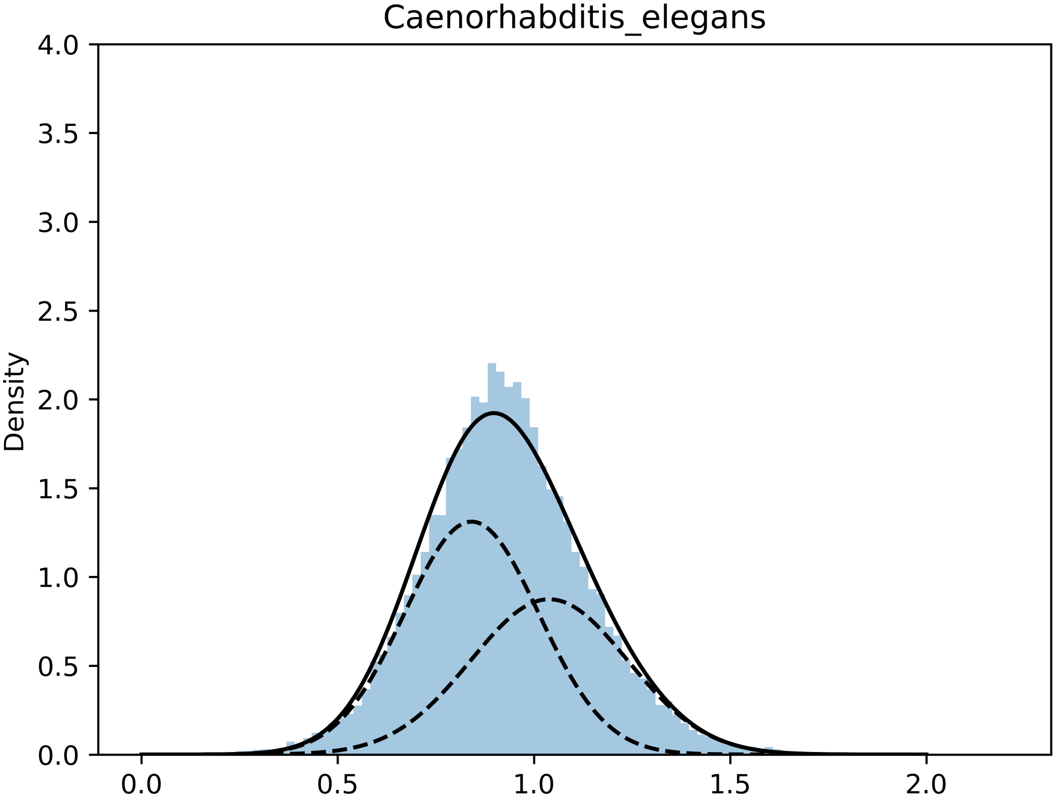
Caenorhabditis_elegans
Density
O/E CpG
0.0
0.5
1.0
1.5
2.0
2.5
3.0
3.5
4.0
0.0
0.5
1.0
1.5
2.0

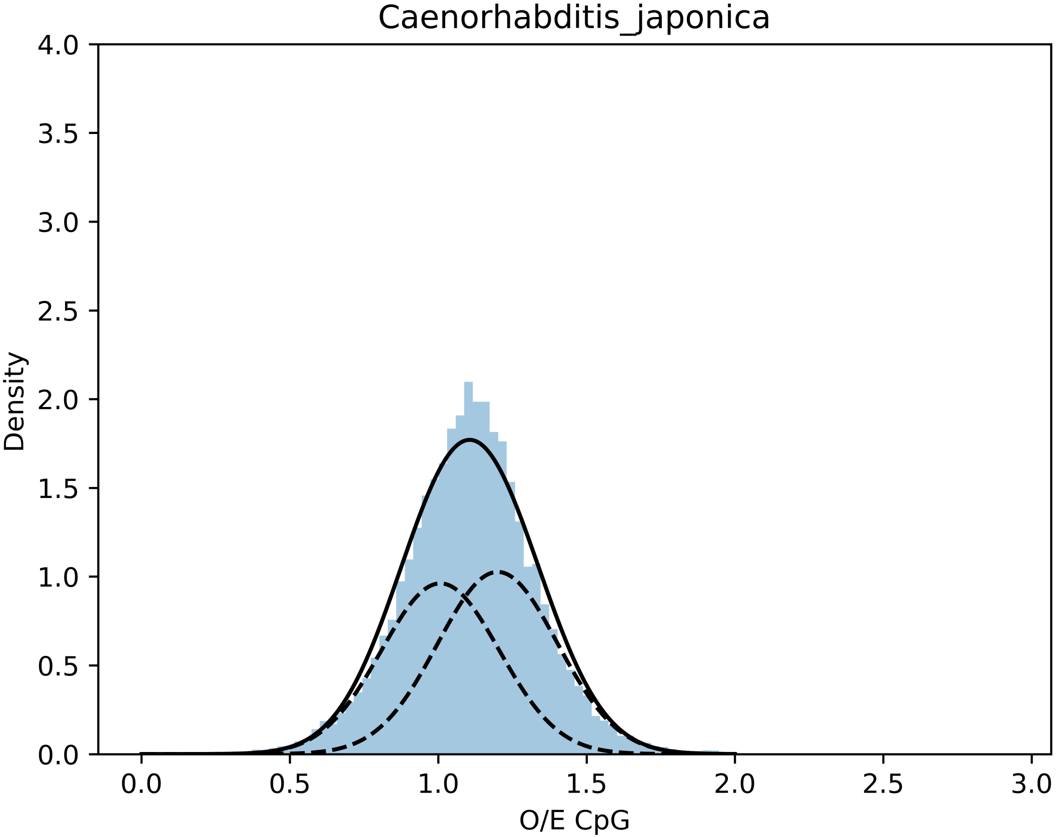
Caenorhabditis_japonica
Density
O/E CpG
4.0
3.5
3.0
2.5
2.0
1.5
1.0
0.5
0.0
0.0
0.5
1.0
1.5
2.0
2.5
3.0

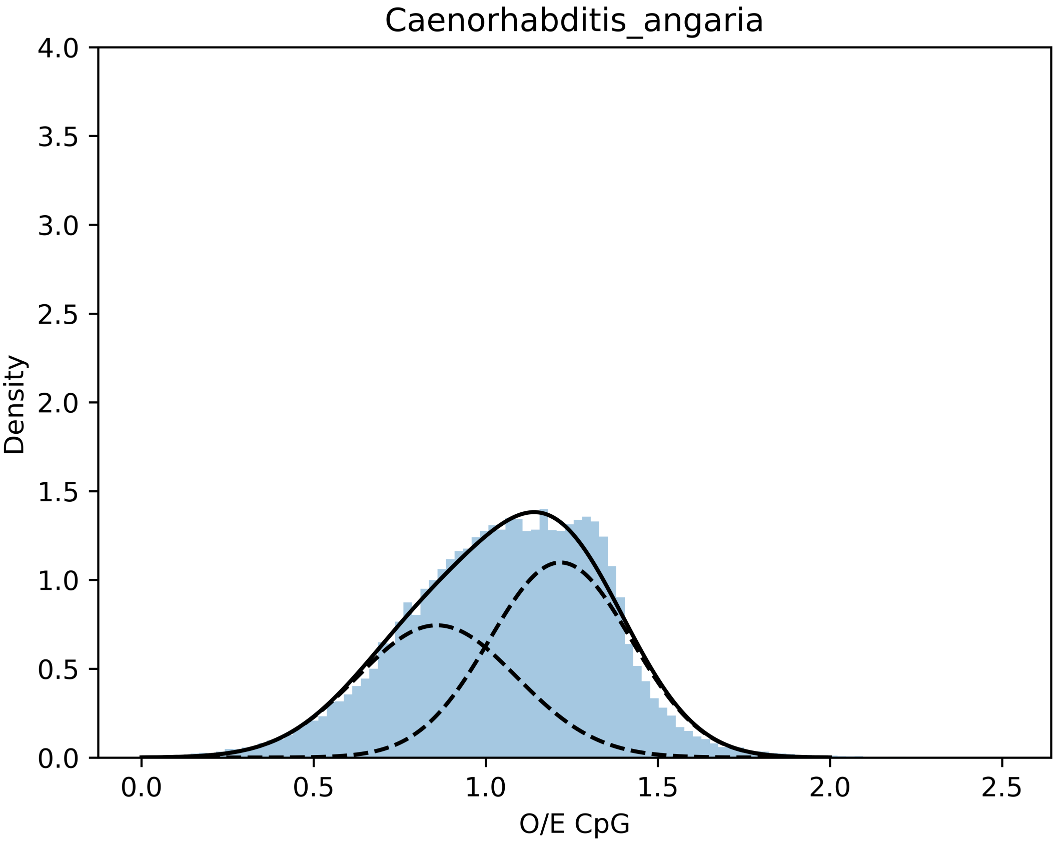
Caenorhabditis_angaria
Density
O/E CpG
0.0
0.5
1.0
1.5
2.0
2.5
3.0
3.5
4.0
0.0
0.5
1.0
1.5
2.0
2.5

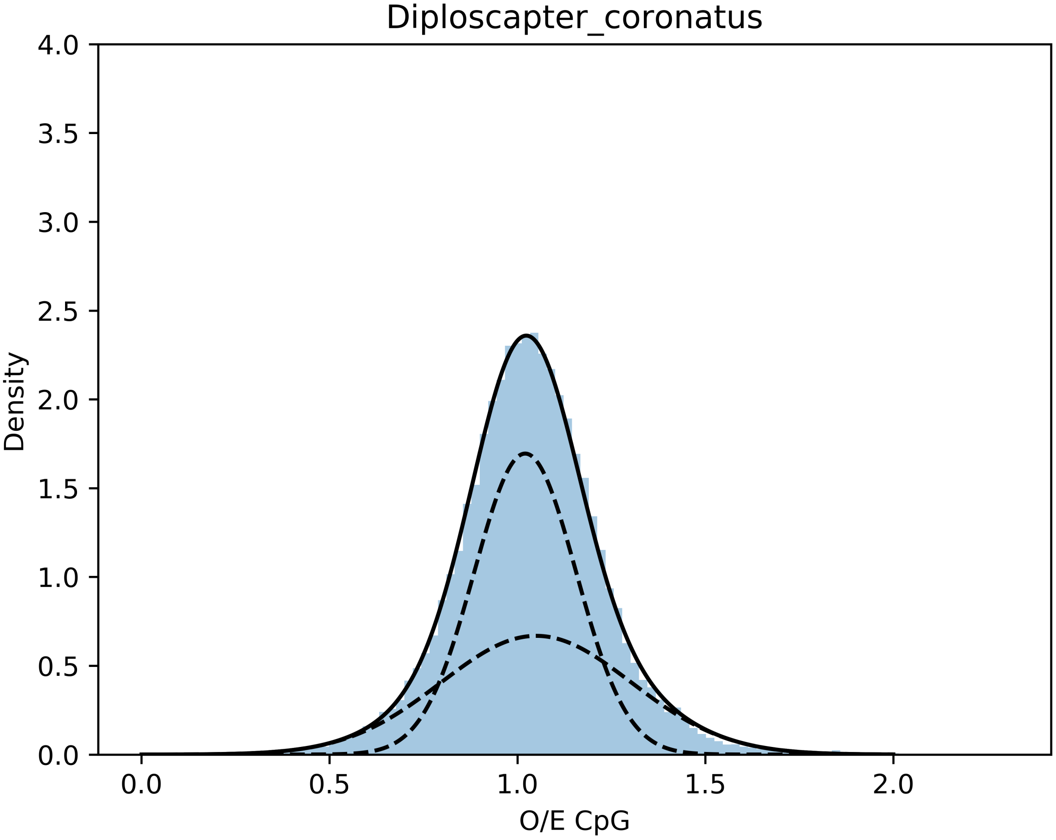
Diploscapter_coronatus
Density
O/E CpG
4.0
3.5
3.0
2.5
2.0
1.5
1.0
0.5
0.0
0.0
0.5
1.0
1.5
2.0

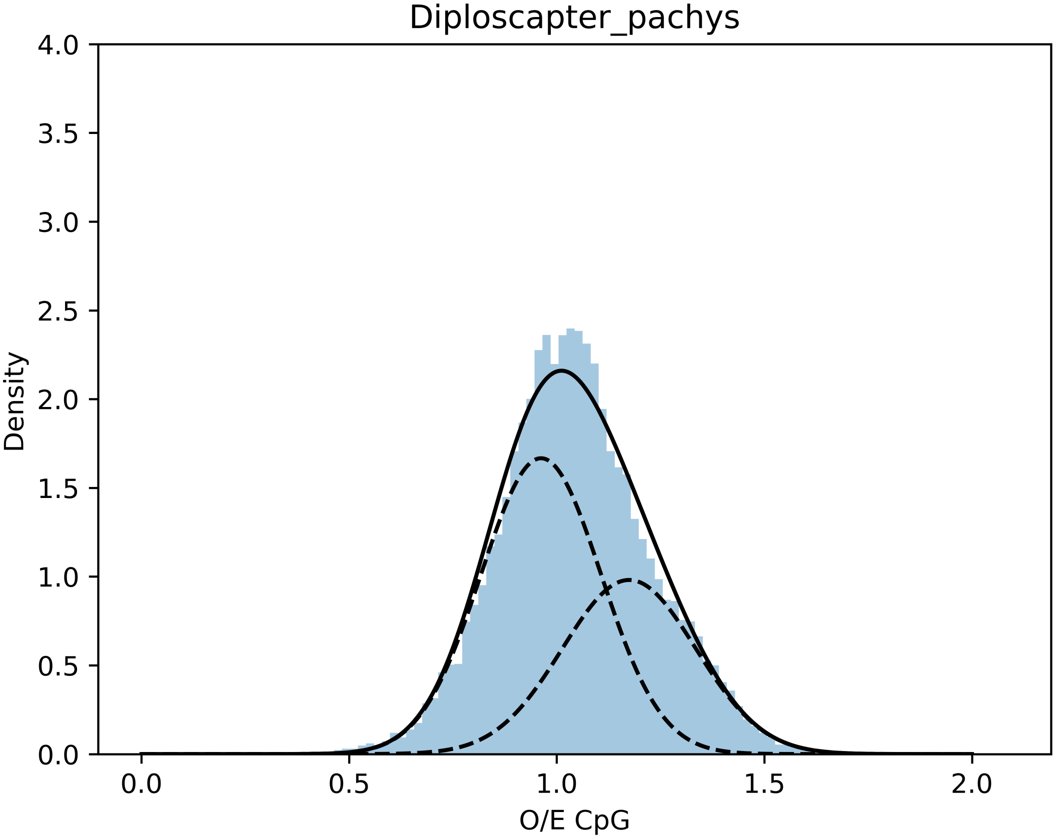
Diploscapter_pachys
Density
O/E CpG
0.0
0.5
1.0
1.5
2.0
2.5
3.0
3.5
4.0
0.0
0.5
1.0
1.5
2.0

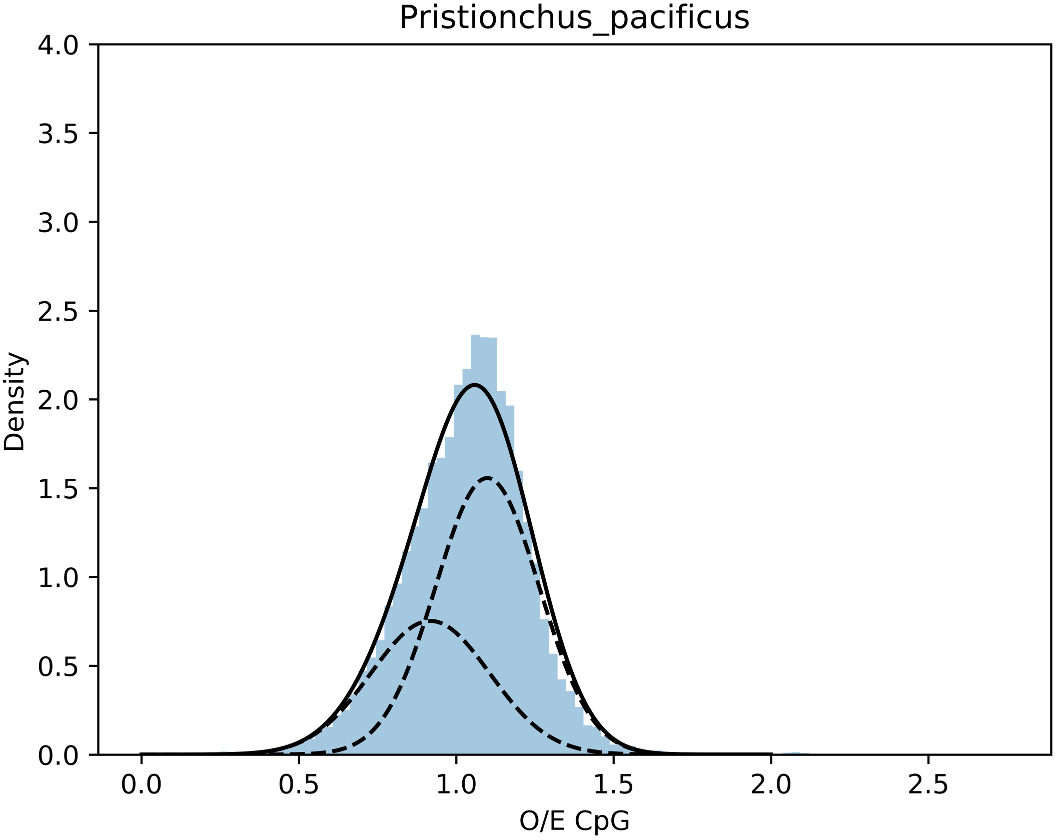
Pristionchus_pacificus
4.0
3.5
3.0
2.5
2.0
1.5
1.0
0.5
0.0
Density
0.0
0.5
1.0
1.5
2.0
2.5
O/E CpG

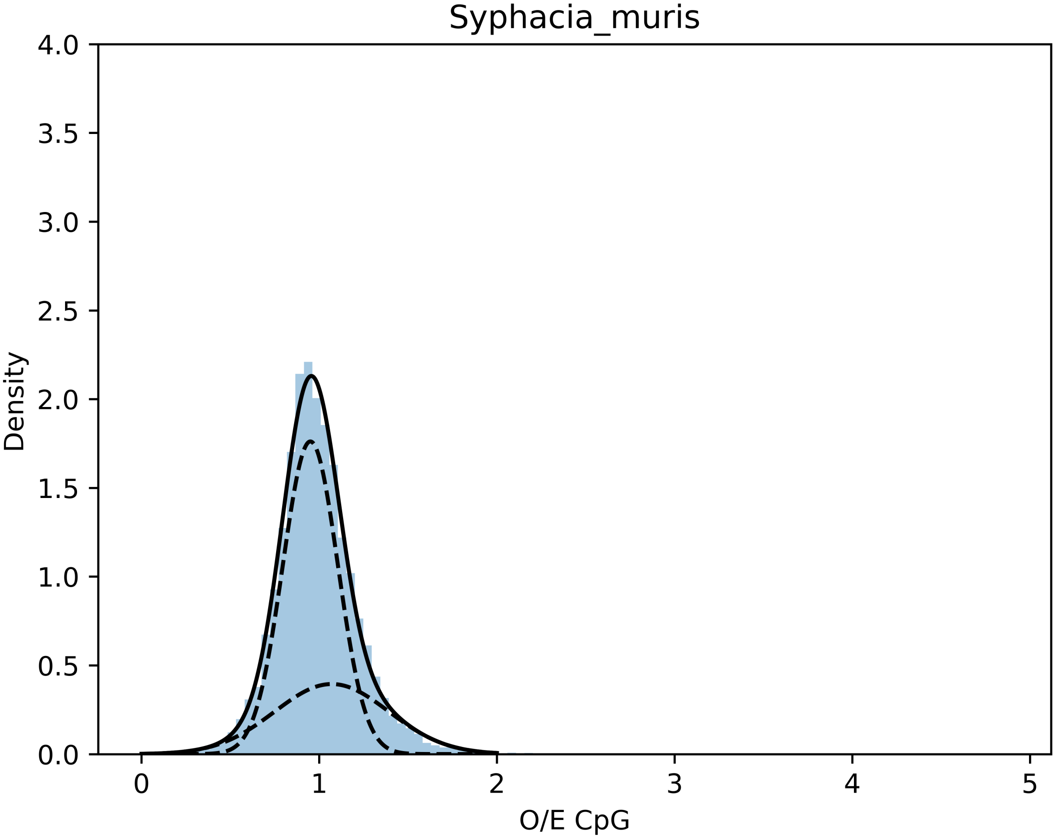

Syphacia_muris
Density
O/E CpG
0.0
0.5
1.0
1.5
2.0
2.5
3.0
3.5
4.0
0
1
2
3
4
5

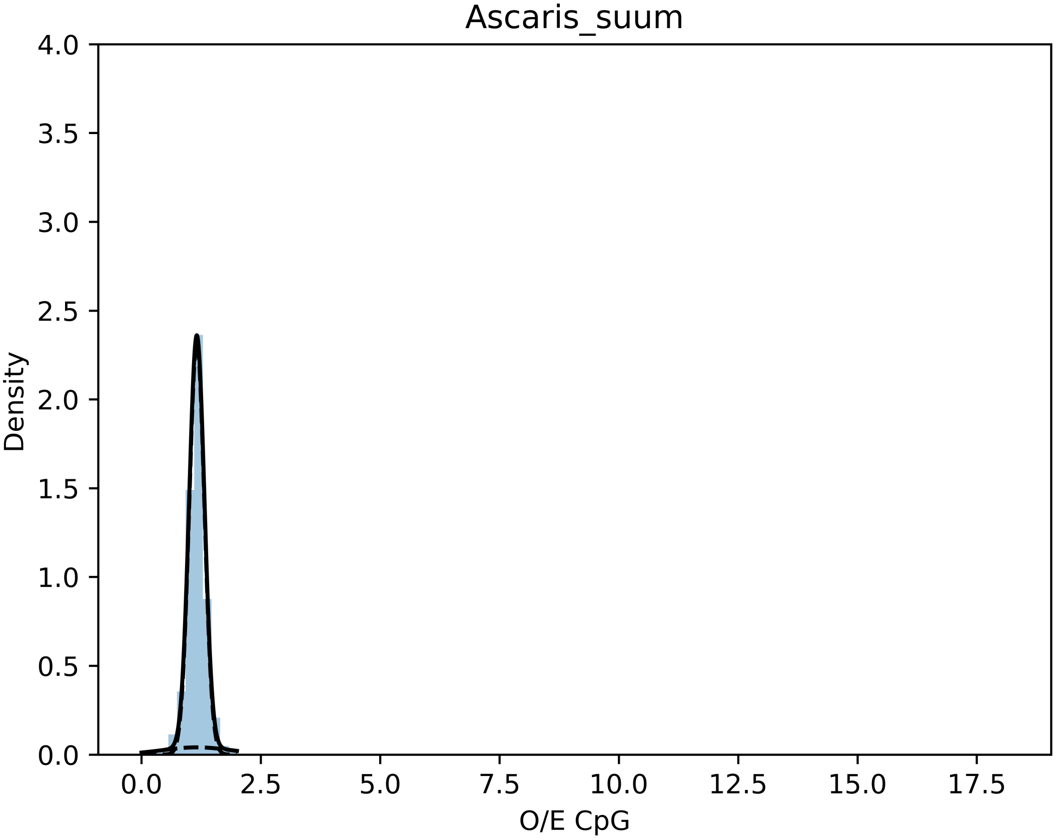
Ascaris_suum
Density
O/E CpG
4.0
3.5
3.0
2.5
2.0
1.5
1.0
0.5
0.0
0.0
2.5
5.0
7.5
10.0
12.5
15.0
17.5

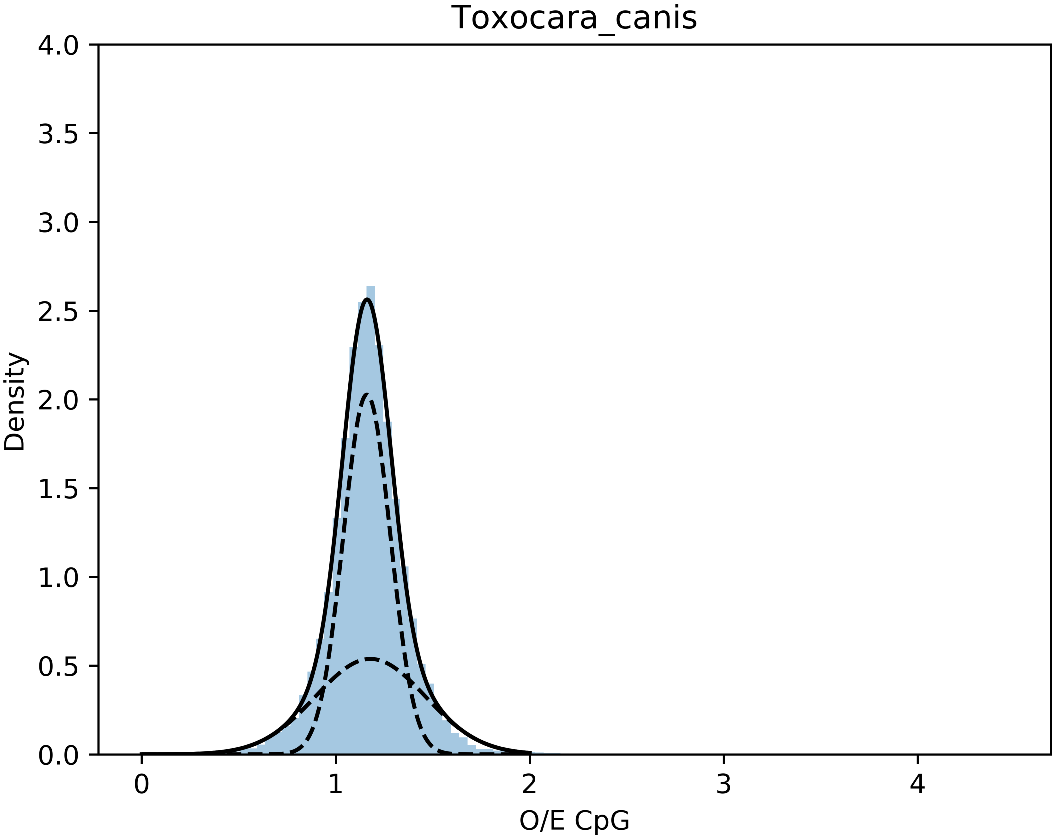

Toxocara_canis
Density
O/E CpG
0.0
0.5
1.0
1.5
2.0
2.5
3.0
3.5
4.0
0
1
2
3
4

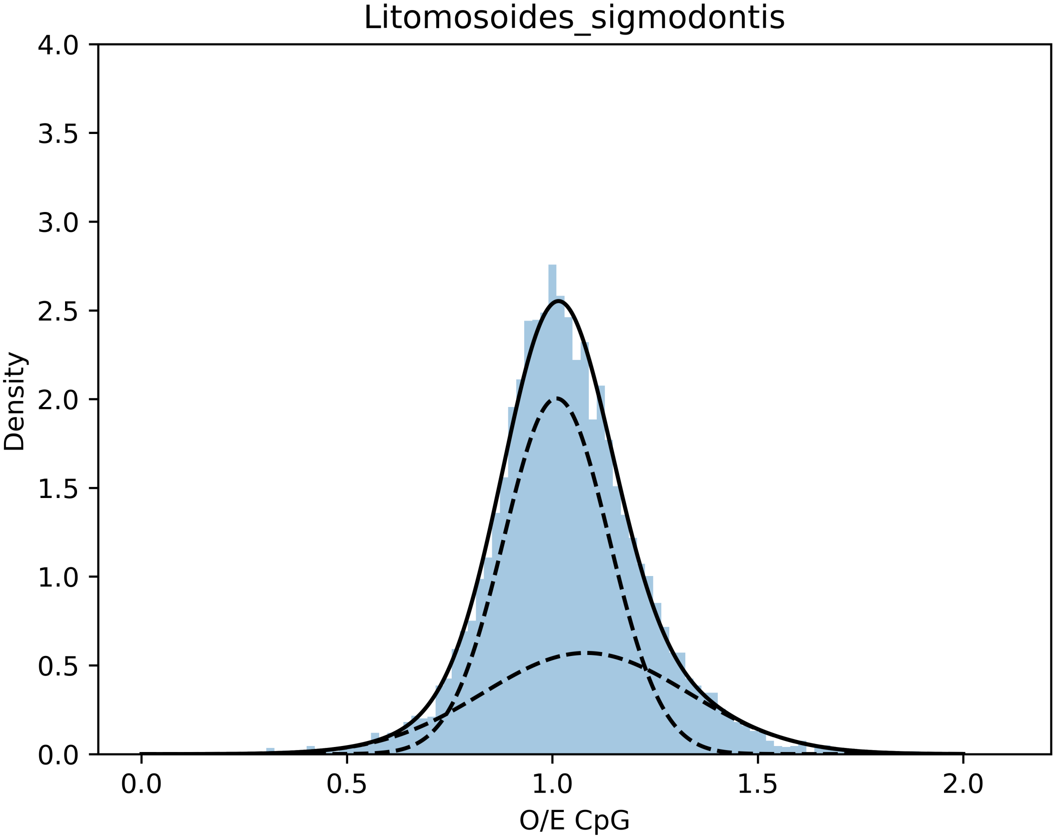

Litomosoides_sigmodontis
Density
O/E CpG
0.0
0.5
1.0
1.5
2.0
2.5
3.0
3.5
4.0
0.0
0.5
1.0
1.5
2.0

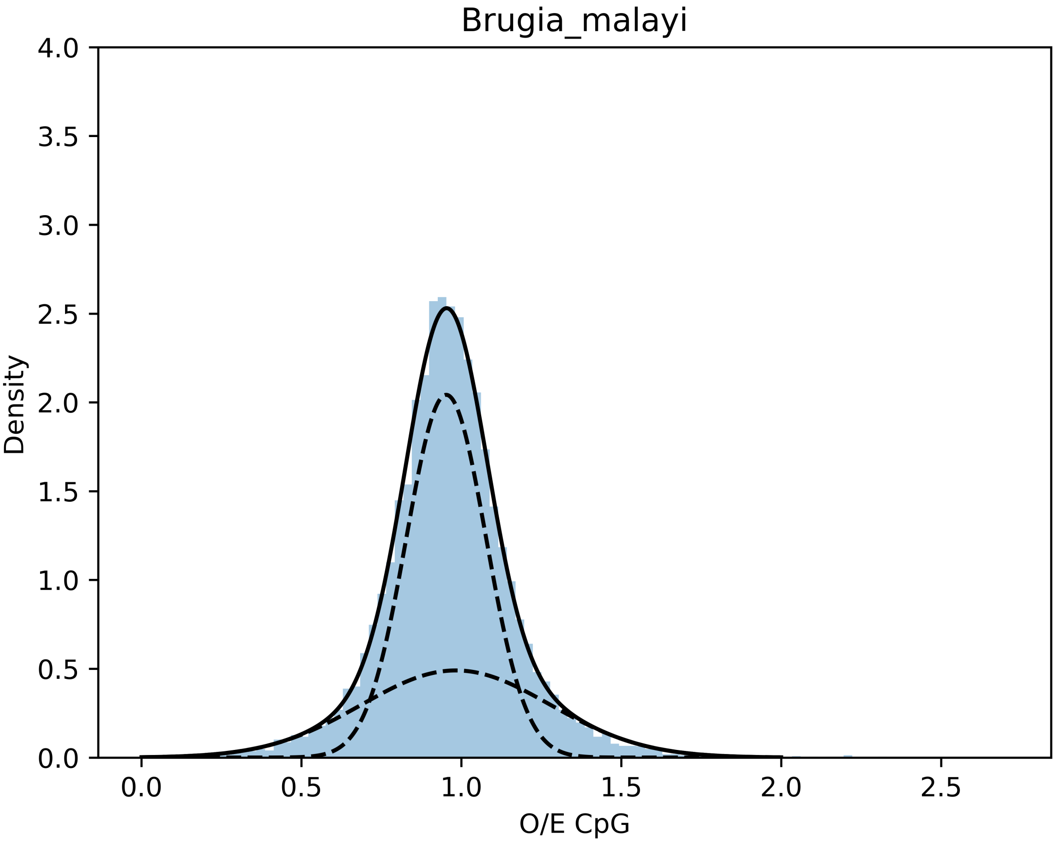
Brugia_malayi
4.0
3.5
3.0
2.5
2.0
1.5
1.0
0.5
0.0
Density
0.0
0.5
1.0
1.5
2.0
2.5
O/E CpG

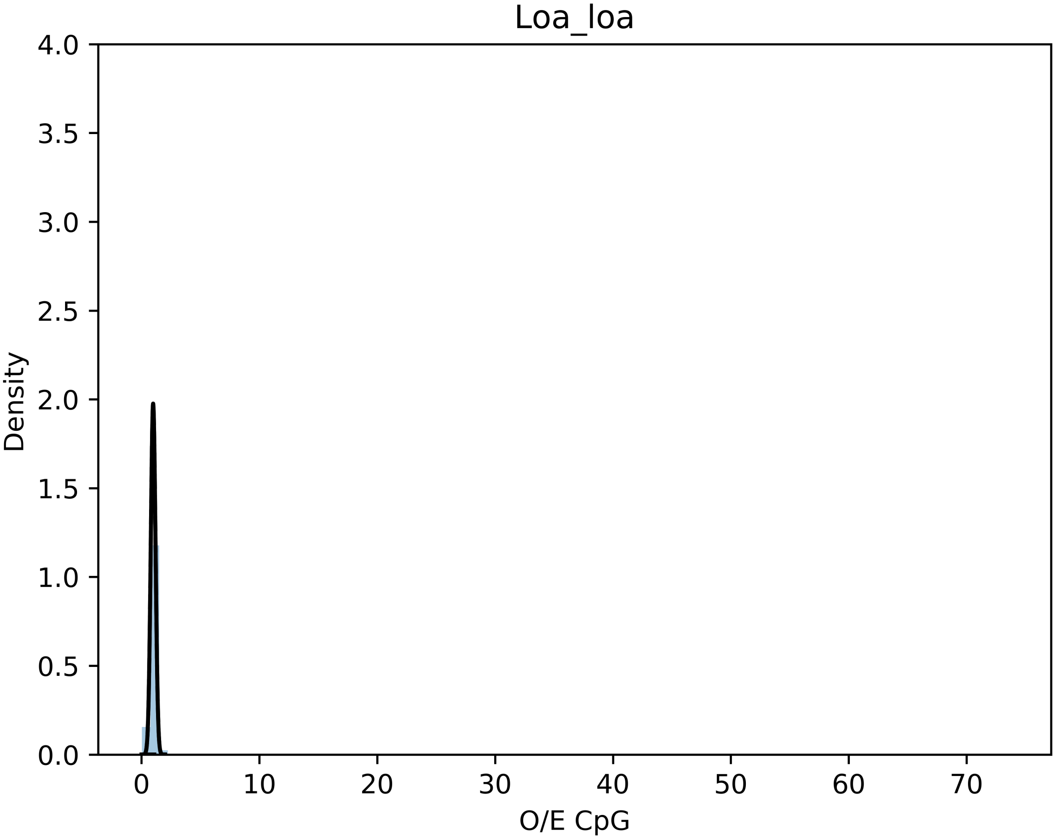
Loa_loa
Density
O/E CpG
4.0
3.5
3.0
2.5
2.0
1.5
1.0
0.5
0.0
0
10
20
30
40
50
60
70

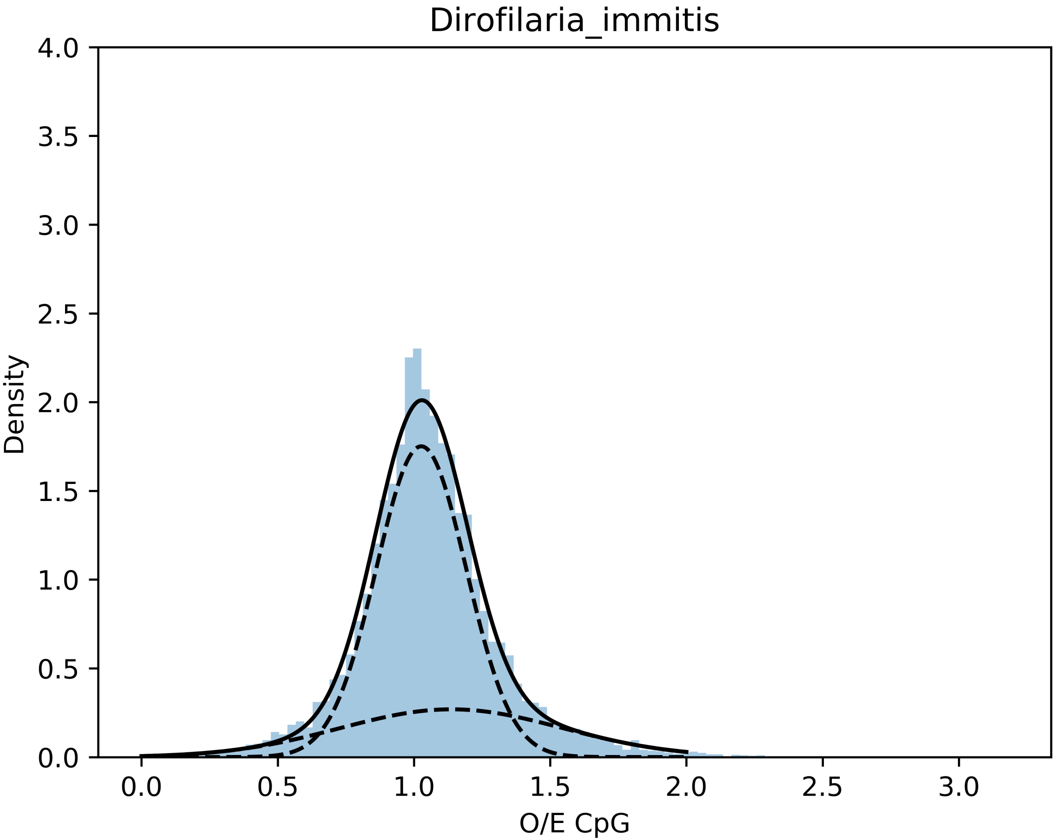
Dirofilaria_immitis
Density
O/E CpG
0.0
0.5
1.0
1.5
2.0
2.5
3.0
3.5
4.0

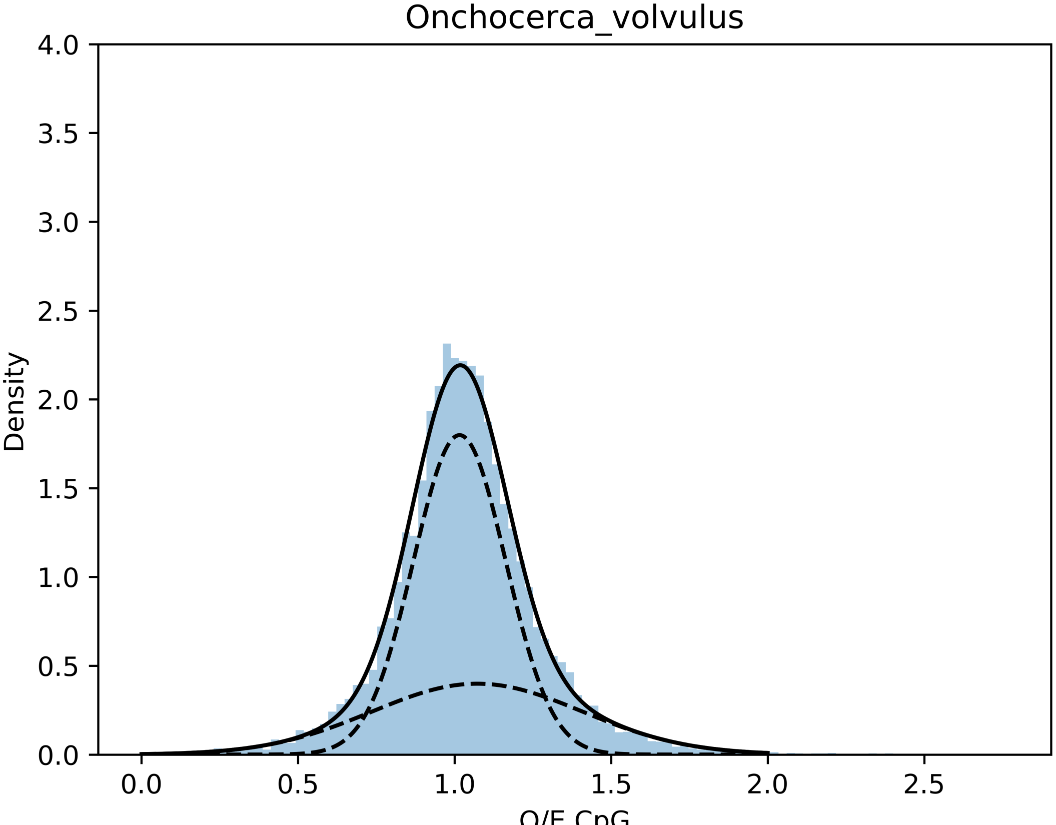
Onchocerca_volvulus
Density
O/E CpG
0.0
0.5
1.0
1.5
2.0
2.5
3.0
3.5
4.0
0.0
0.5
1.0
1.5
2.0
2.5

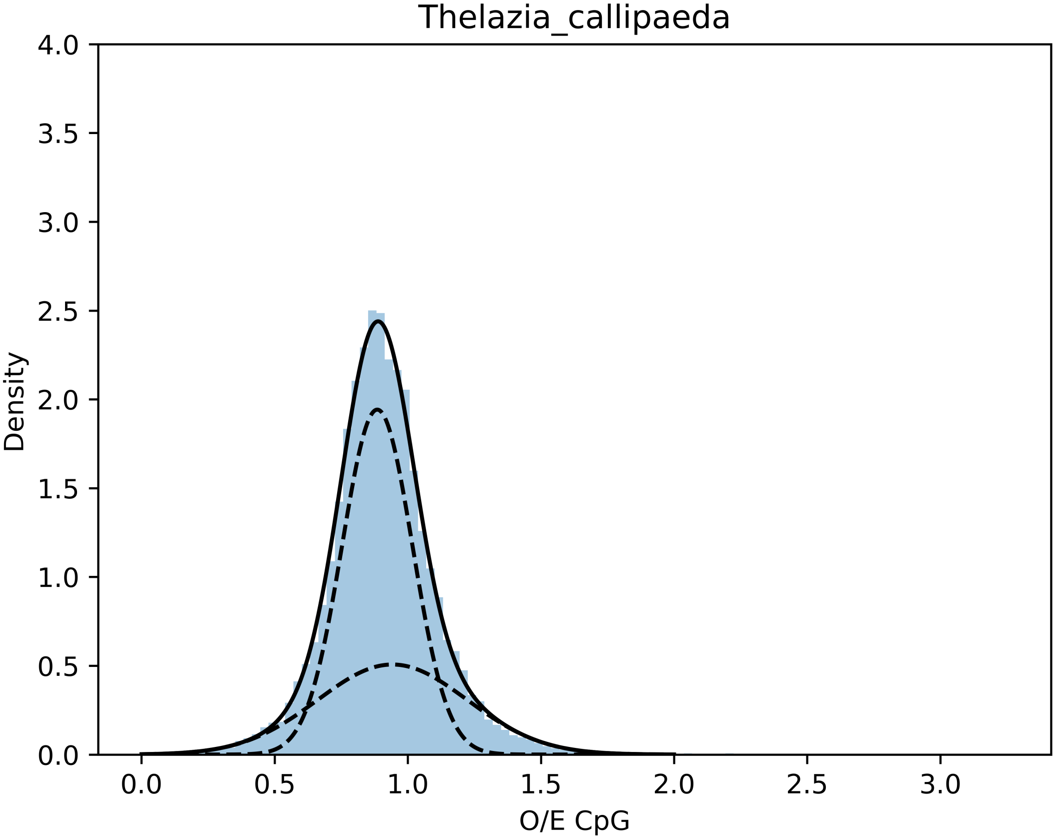
Thelazia_callipaeda
Density
O/E CpG
0.0
0.5
1.0
1.5
2.0
2.5
3.0
3.5
4.0

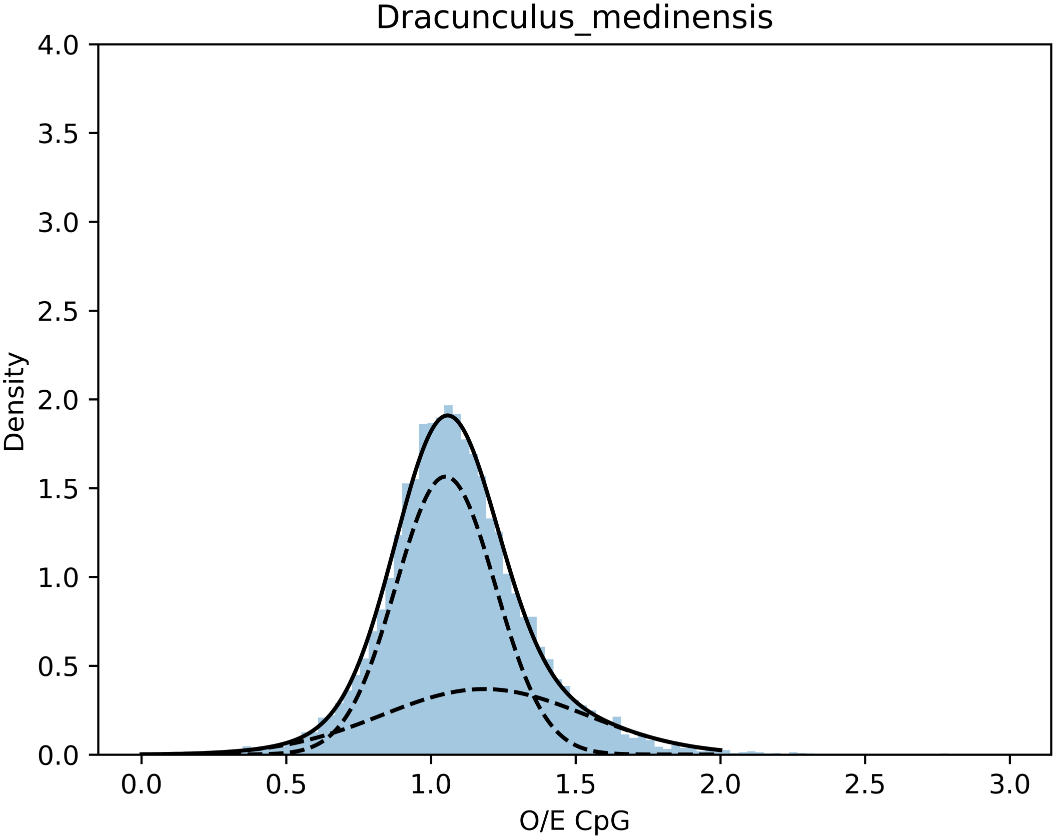
Dracunculus_medinensis
Density
O/E CpG
0.0
0.5
1.0
1.5
2.0
2.5
3.0
3.5
4.0

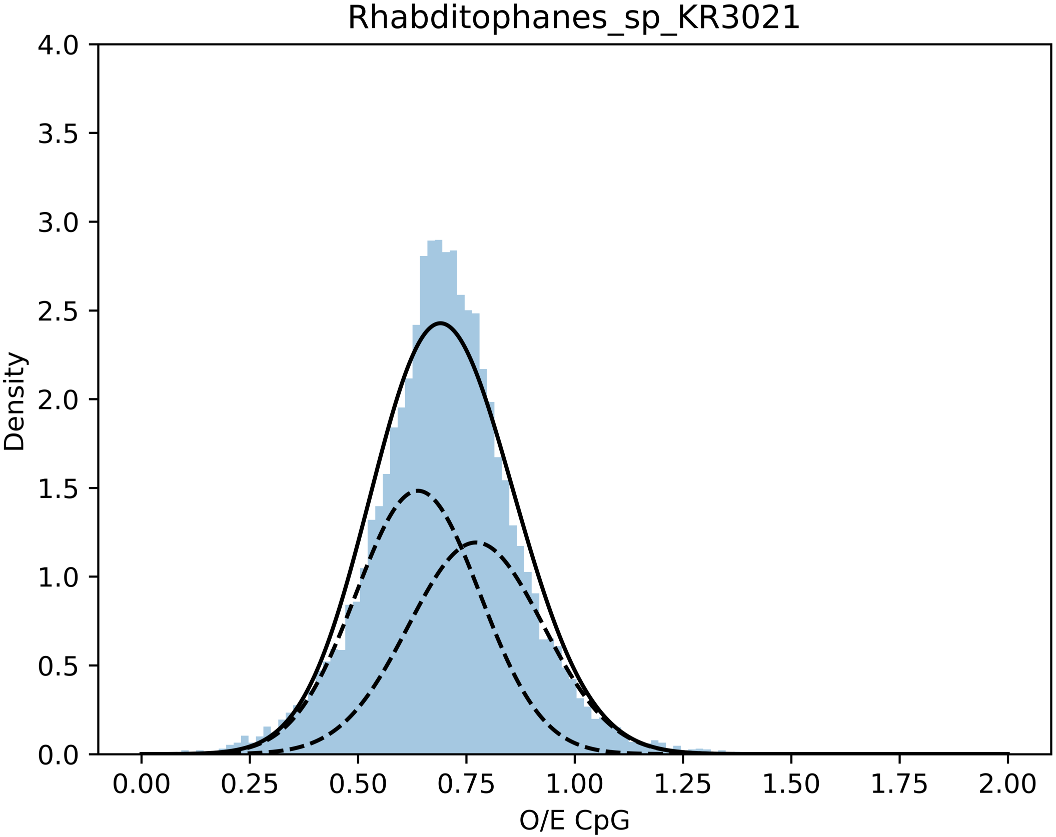

Rhabditophanes_sp_KR3021
Density
O/E CpG
0.0
0.5
1.0
1.5
2.0
2.5
3.0
3.5
4.0
0.00
0.25
0.50
0.75
1.00
1.25
1.50
1.75
2.00

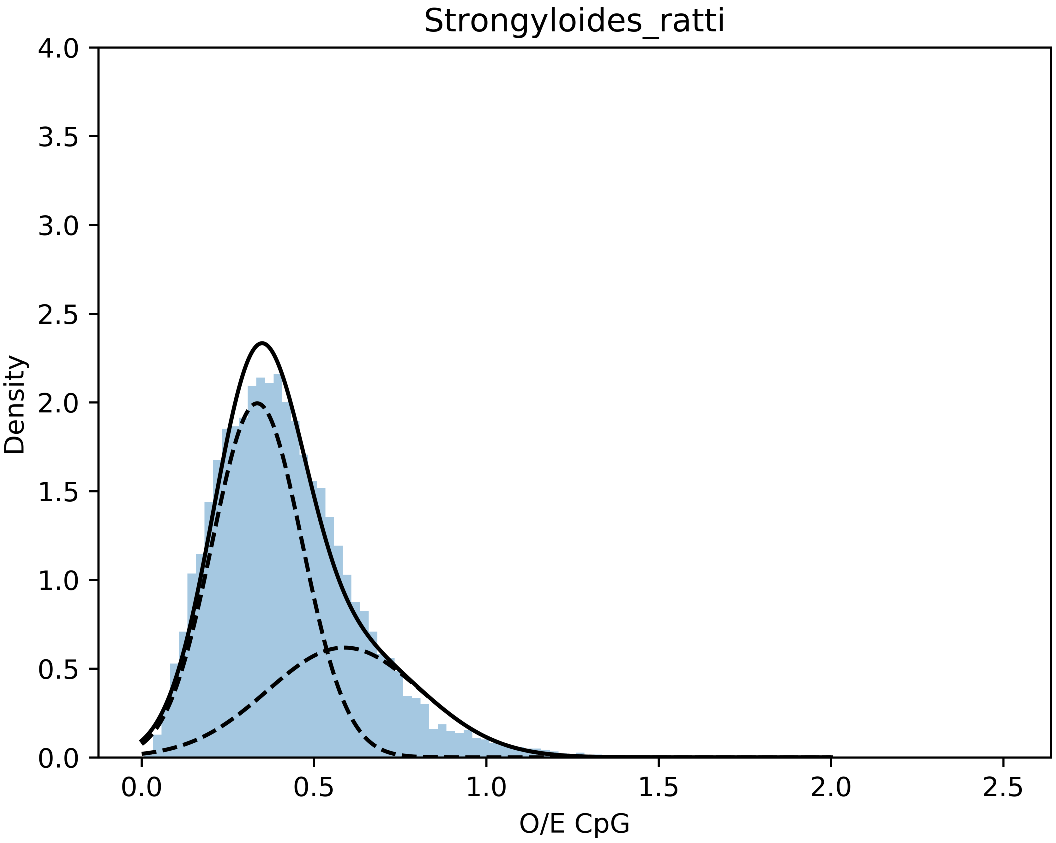
Strongyloides_ratti
Density
O/E CpG
0.0
0.5
1.0
1.5
2.0
2.5
3.0
3.5
4.0
0.0
0.5
1.0
1.5
2.0
2.5

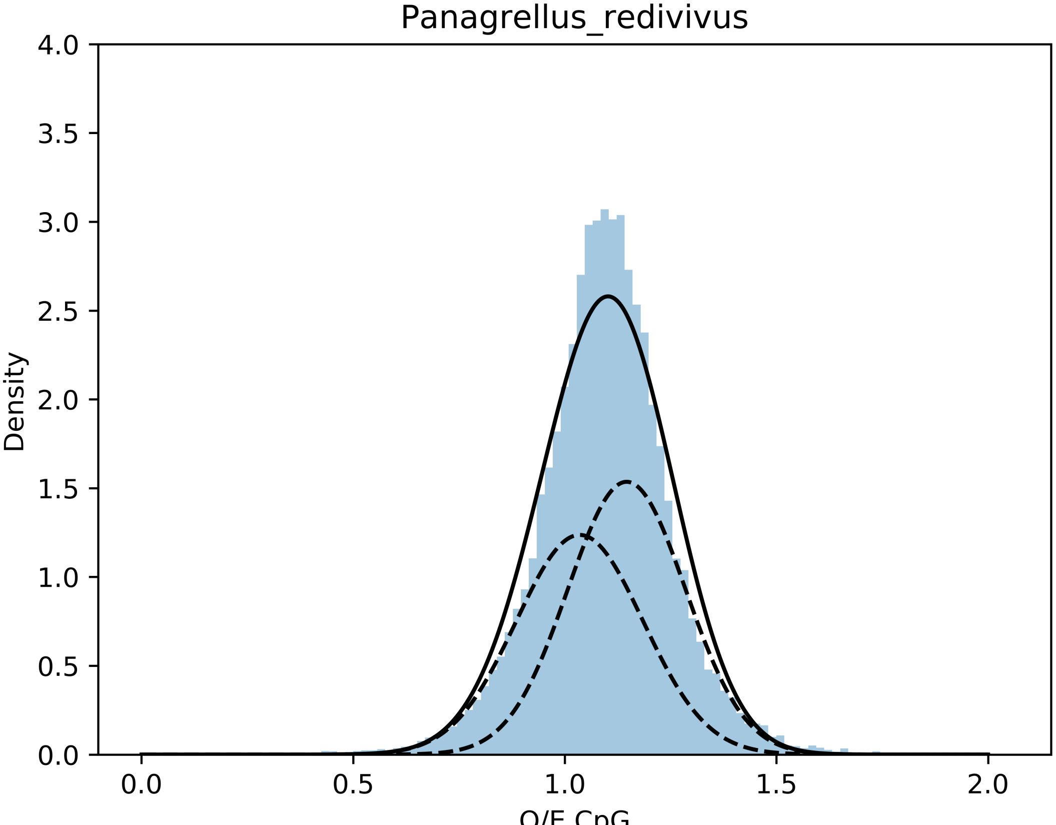

Panagrellus_redivivus
Density
O/E CpG
0.0
0.5
1.0
1.5
2.0
2.5
3.0
3.5
4.0
0.0
0.5
1.0
1.5
2.0

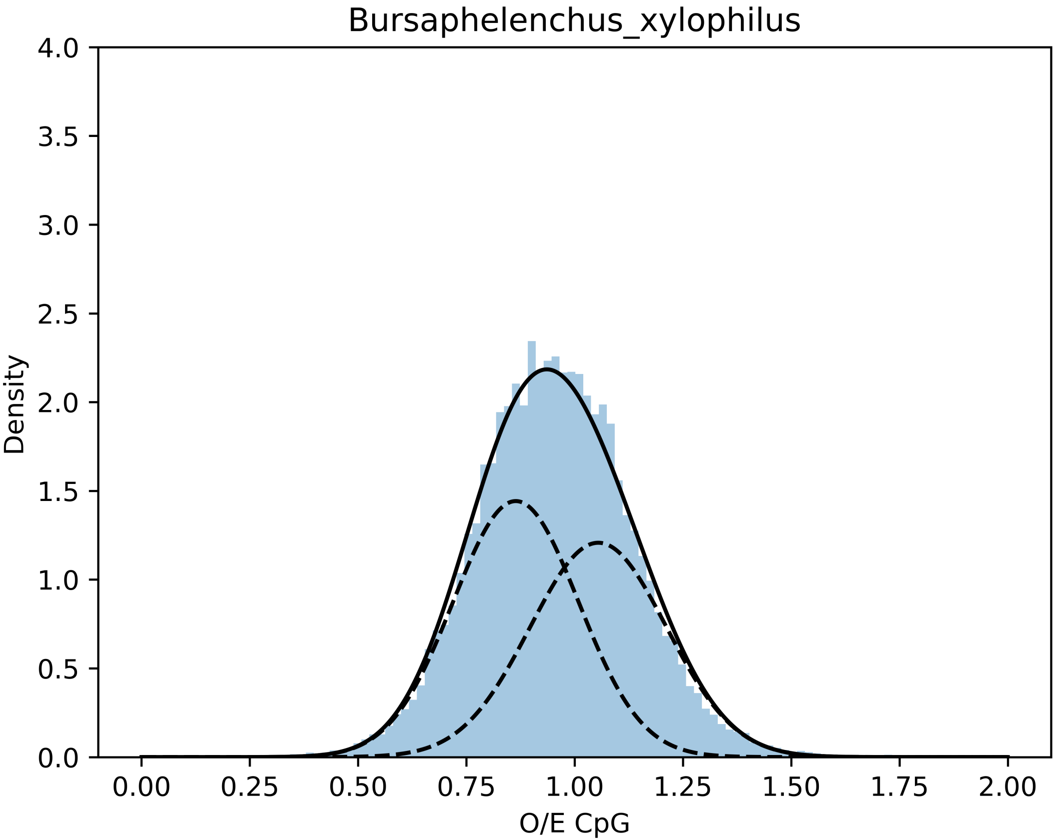

Bursaphelenchus_xylophilus
Density
O/E CpG
0.0
0.5
1.0
1.5
2.0
2.5
3.0
3.5
4.0
0.00
0.25
0.50
0.75
1.00
1.25
1.50
1.75
2.00

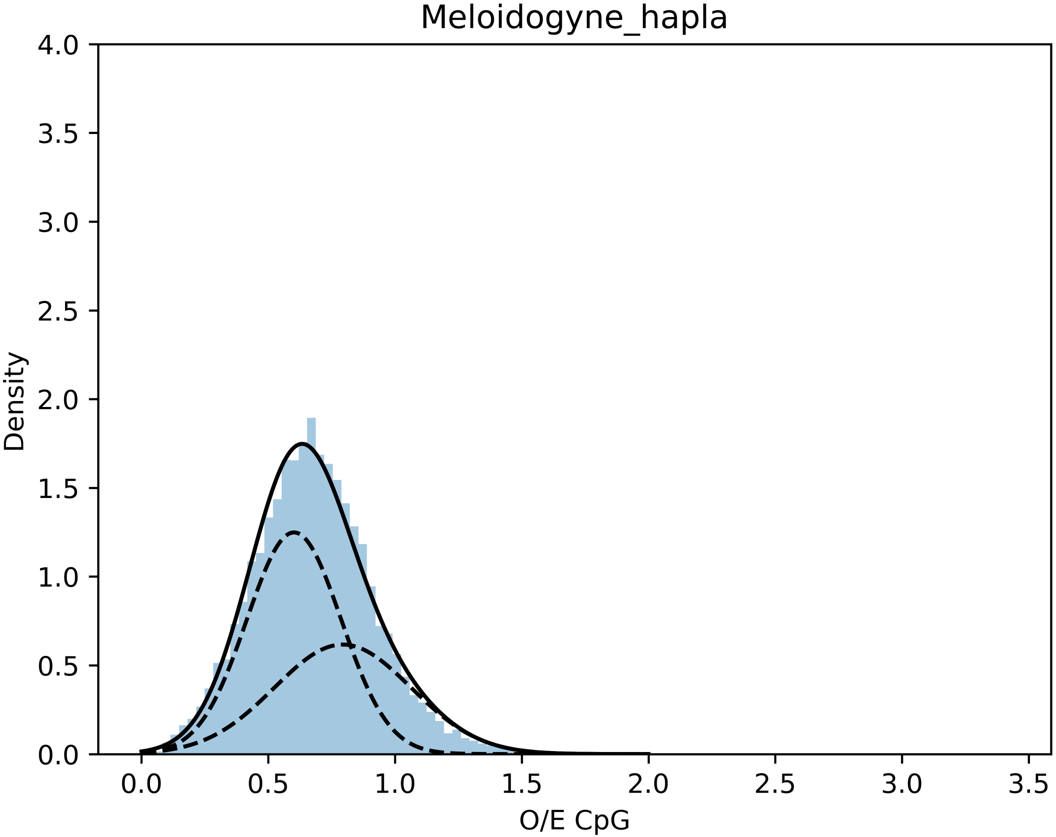
Meloidogyne_hapla
Density
O/E CpG
0.0
0.5
1.0
1.5
2.0
2.5
3.0
3.5
4.0

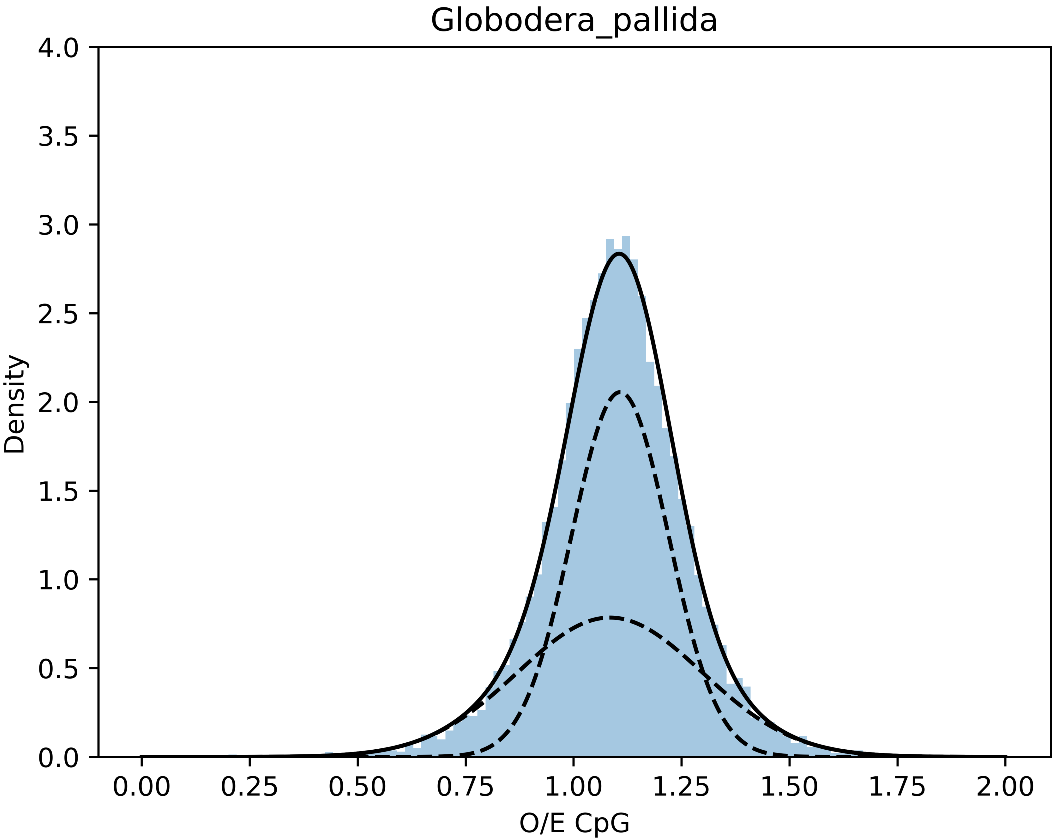

Globodera_pallida
Density
O/E CpG
4.0
3.5
3.0
2.5
2.0
1.5
1.0
0.5
0.0
0.00
0.25
0.50
0.75
1.00
1.25
1.50
1.75
2.00

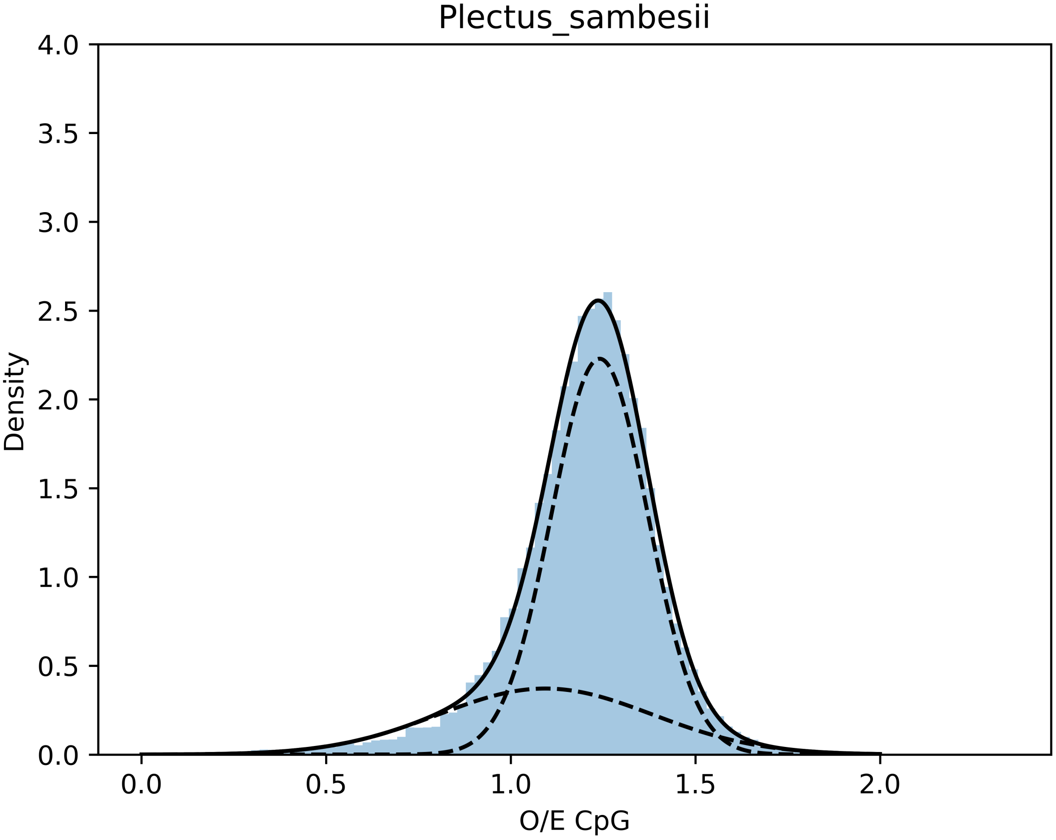
Plectus_sambesii
Density
O/E CpG
0.0
0.5
1.0
1.5
2.0
2.5
3.0
3.5
4.0
0.0
0.5
1.0
1.5
2.0

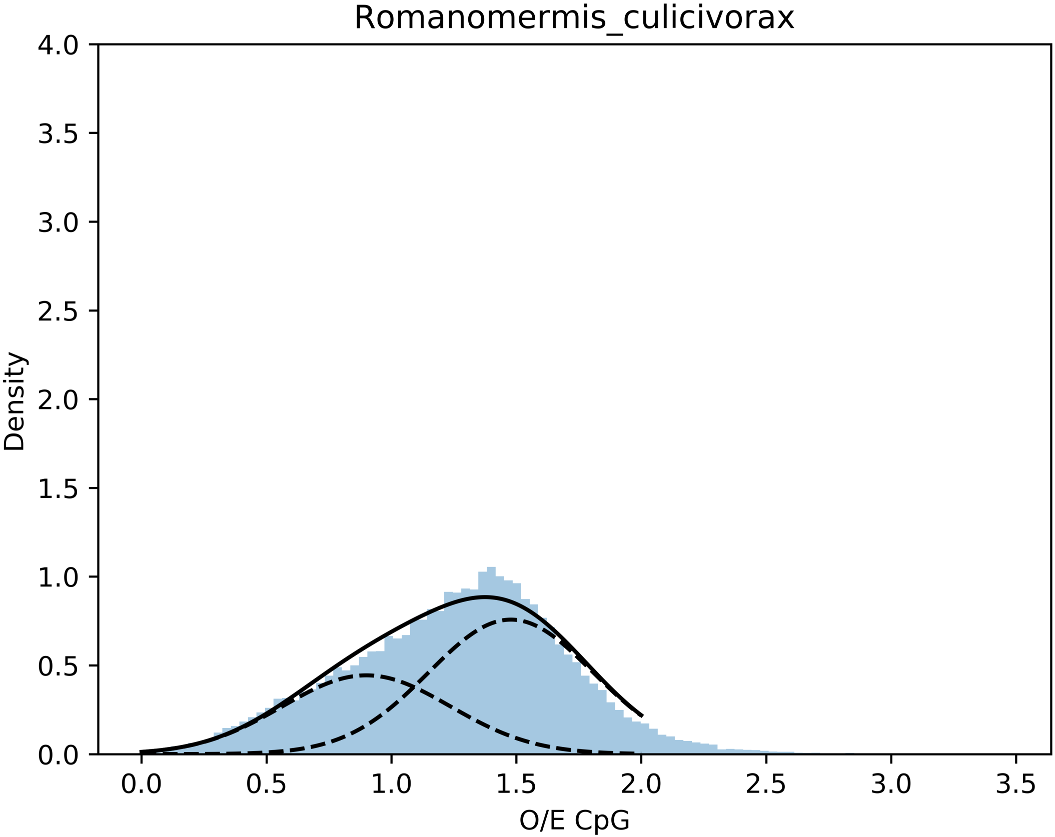

Romanomermis_culicivorax
Density
O/E CpG
4.0
3.5
3.0
2.5
2.0
1.5
1.0
0.5
0.0
0.0
0.5
1.0
1.5
2.0
2.5
3.0
3.5

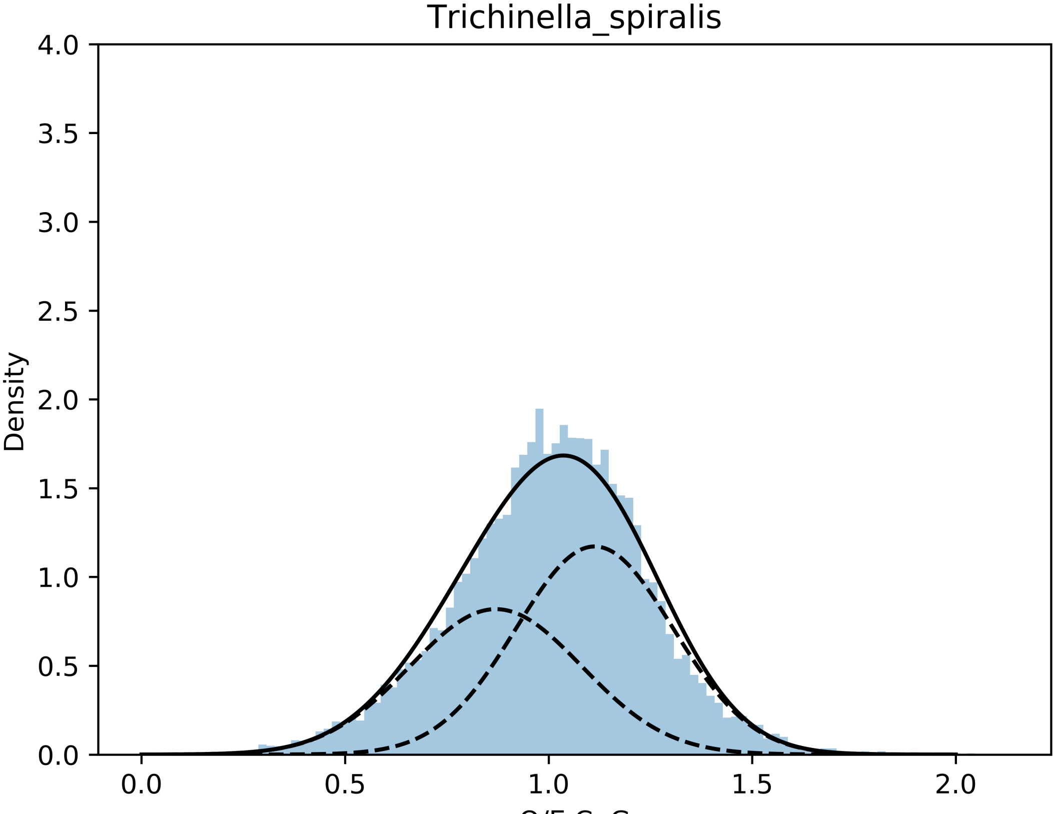
Trichinella_spiralis
Density
O/E CpG
4.0
3.5
3.0
2.5
2.0
1.5
1.0
0.5
0.0
0.0
0.5
1.0
1.5
2.0

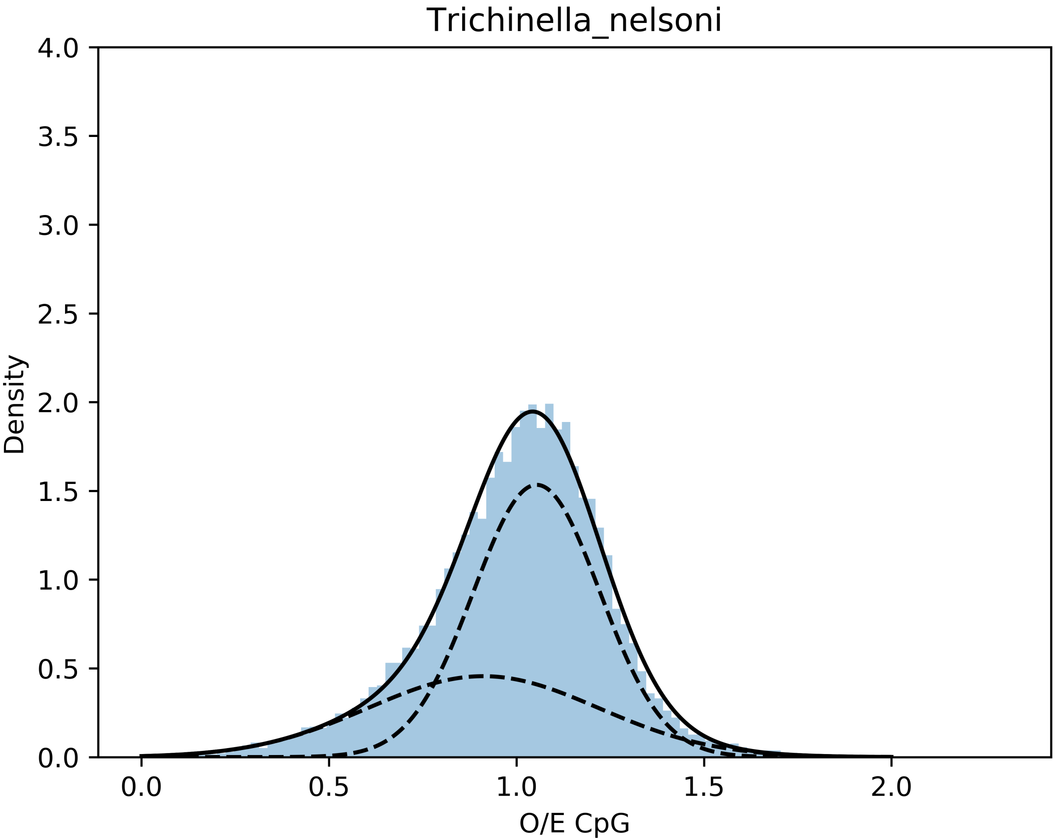
Trichinella_nelsoni
Density
O/E CpG
0.0
0.5
1.0
1.5
2.0
2.5
3.0
3.5
4.0
0.0
0.5
1.0
1.5
2.0

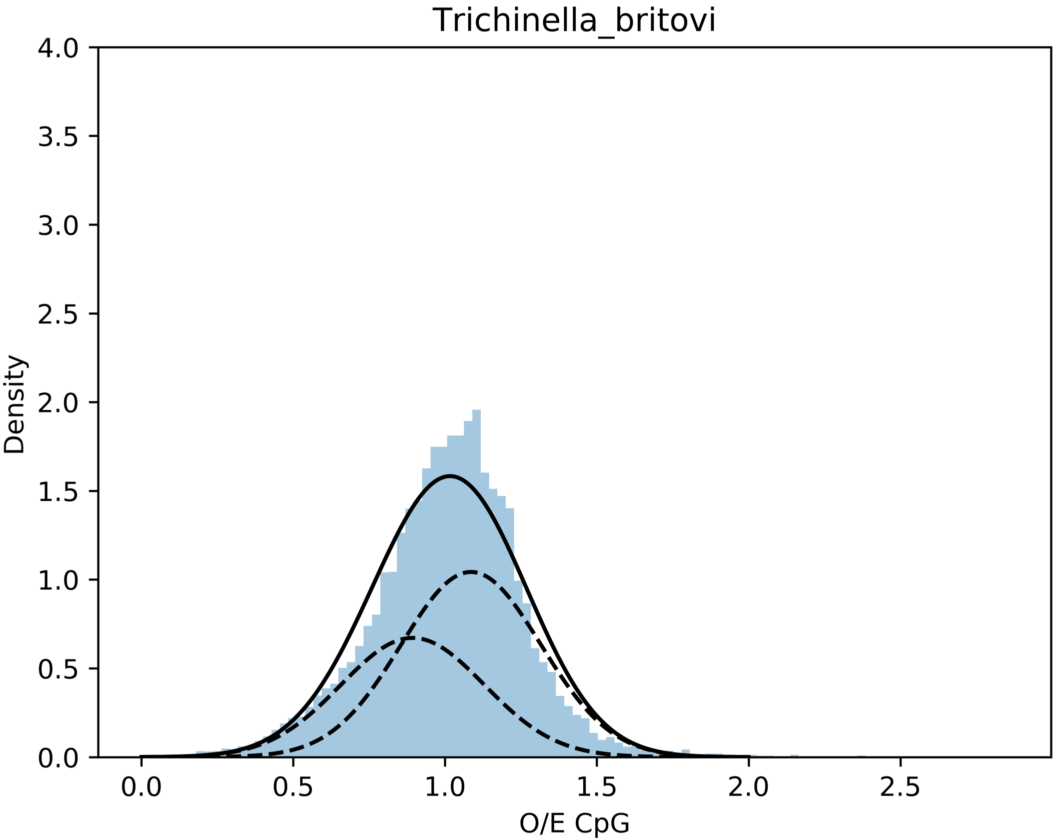
Trichinella_britovi
Density
O/E CpG
4.0
3.5
3.0
2.5
2.0
1.5
1.0
0.5
0.0
0.0
0.5
1.0
1.5
2.0
2.5

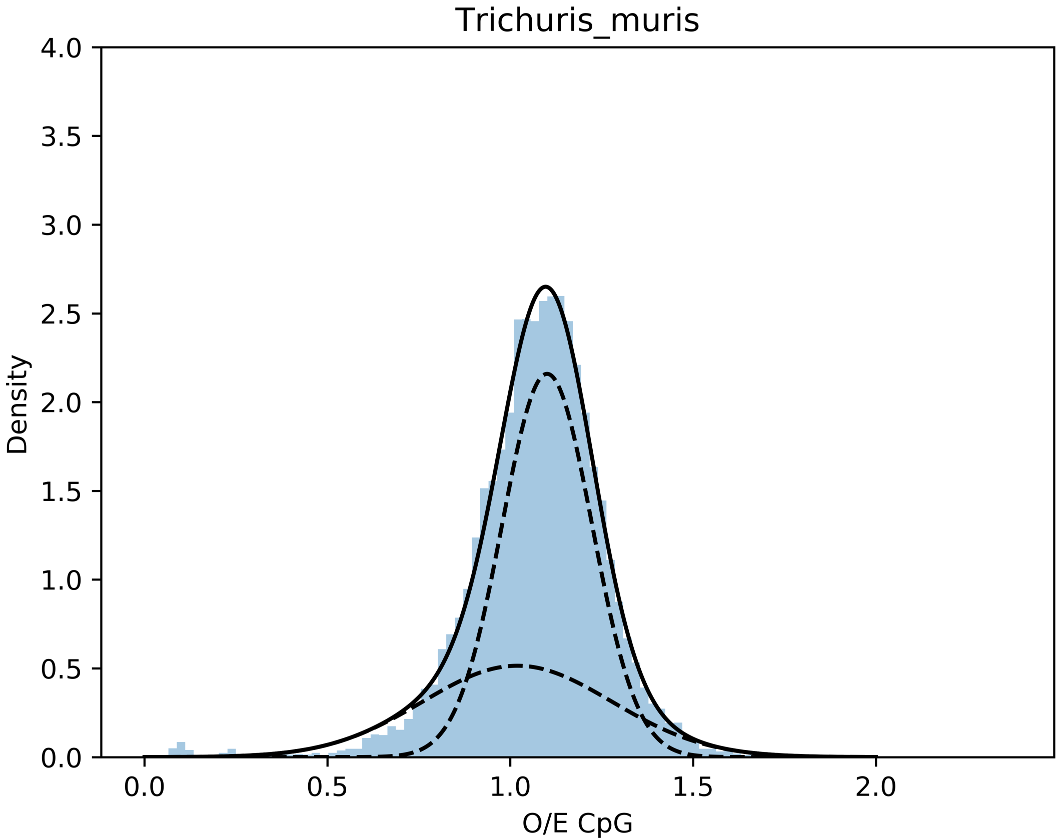

Trichuris_muris
Density
O/E CpG
0.0
0.5
1.0
1.5
2.0
2.5
3.0
3.5
4.0
0.0
0.5
1.0
1.5
2.0

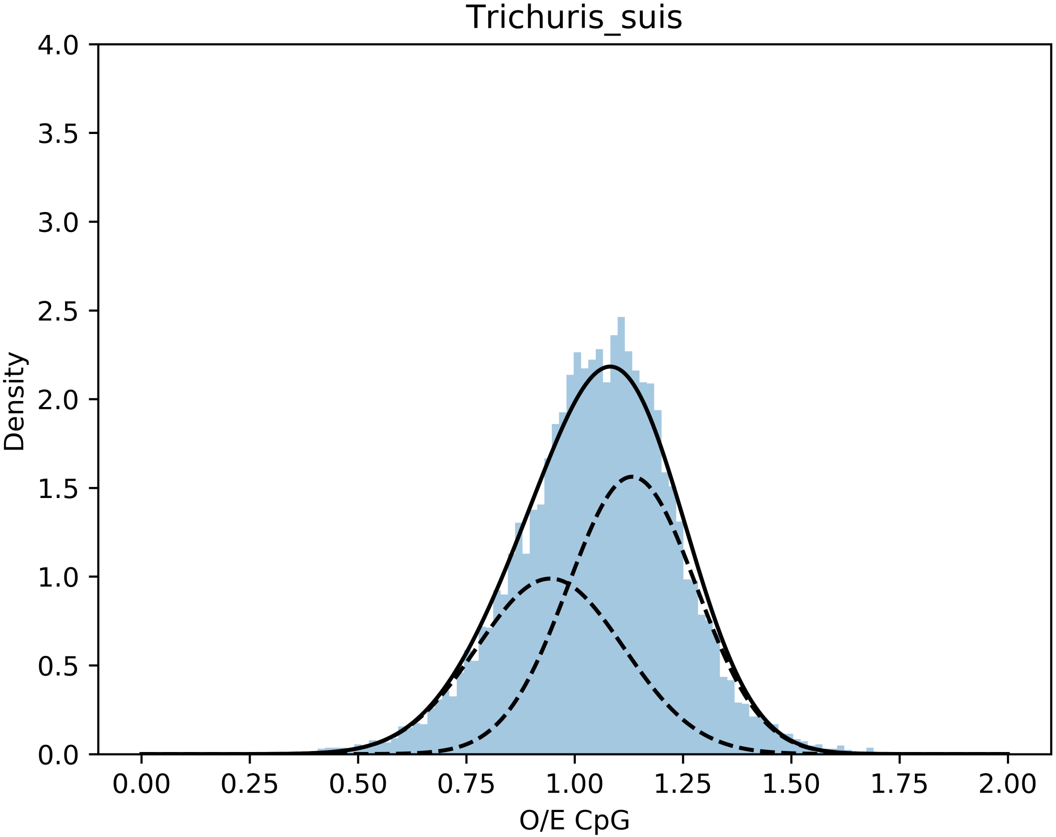
Trichuris_suis
Density
O/E CpG
0.0
0.5
1.0
1.5
2.0
2.5
3.0
3.5
4.0
0.00
0.25
0.50
0.75
1.00
1.25
1.50
1.75
2.00

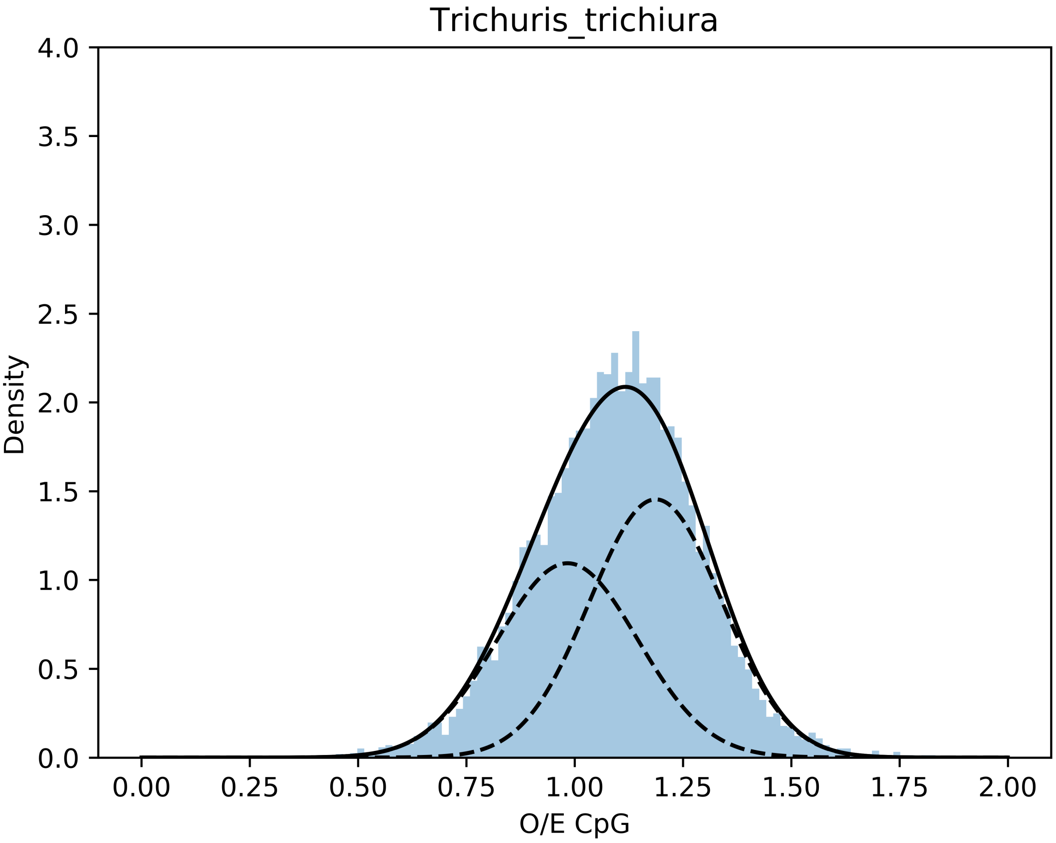
Trichuris_trichiura
Density
O/E CpG
0.0
0.5
1.0
1.5
2.0
2.5
3.0
3.5
4.0
0.00
0.25
0.50
0.75
1.00
1.25
1.50
1.75
2.00

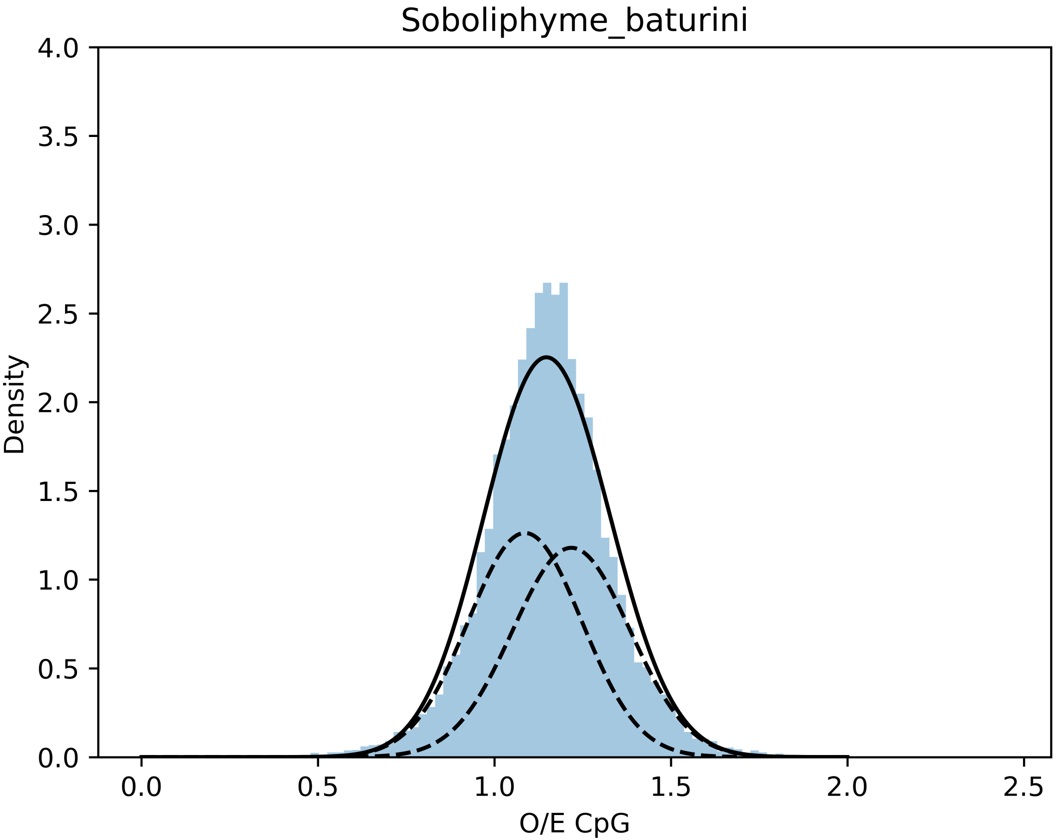
Soboliphyme_baturini
Density
O/E CpG
0.0
0.5
1.0
1.5
2.0
2.5
3.0
3.5
4.0
0.0
0.5
1.0
1.5
2.0
2.5

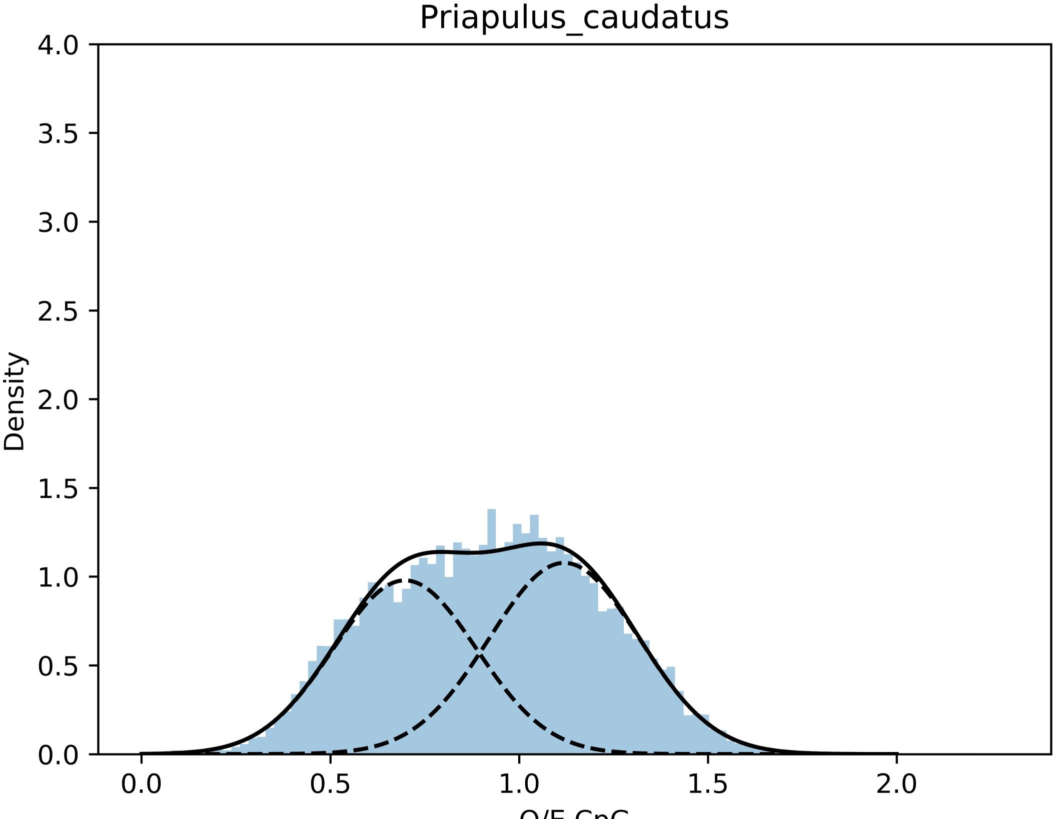
Priapulus_caudatus
Density
O/E CpG
0.0
0.5
1.0
1.5
2.0
2.5
3.0
3.5
4.0
0.0
0.5
1.0
1.5
2.0

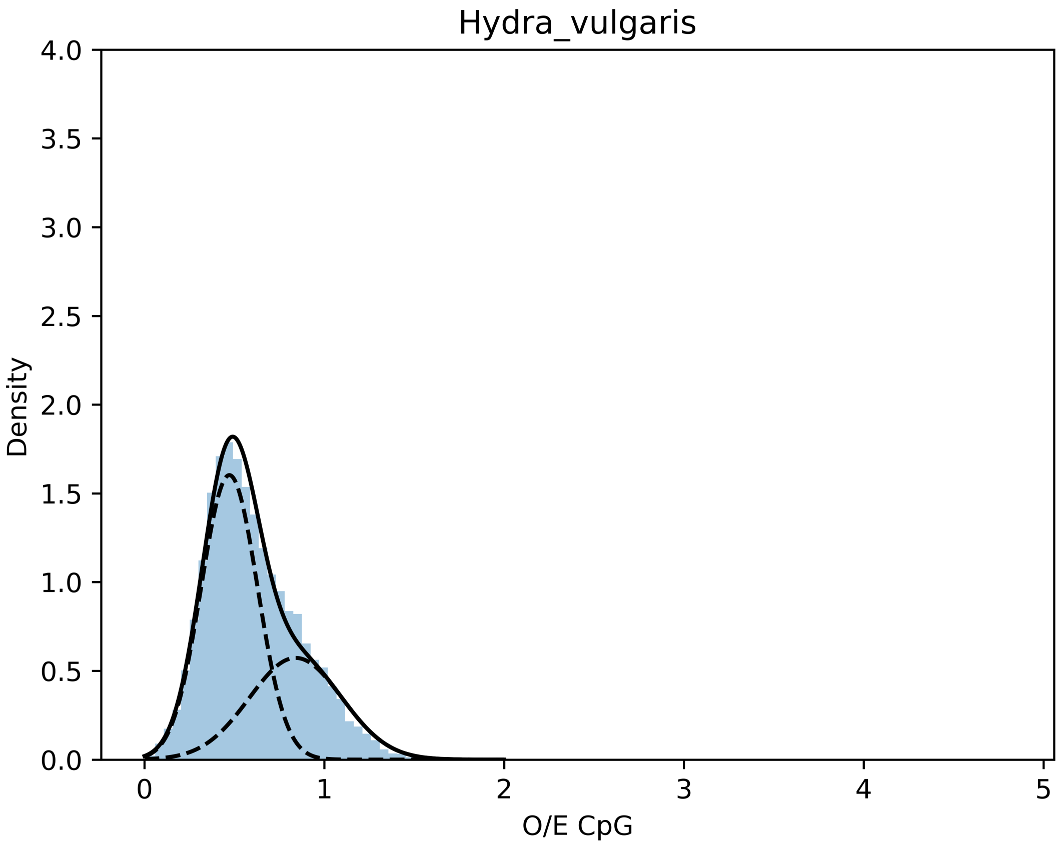

Hydra_vulgaris
Density
O/E CpG
4.0
3.5
3.0
2.5
2.0
1.5
1.0
0.5
0.0
0
1
2
3
4
5

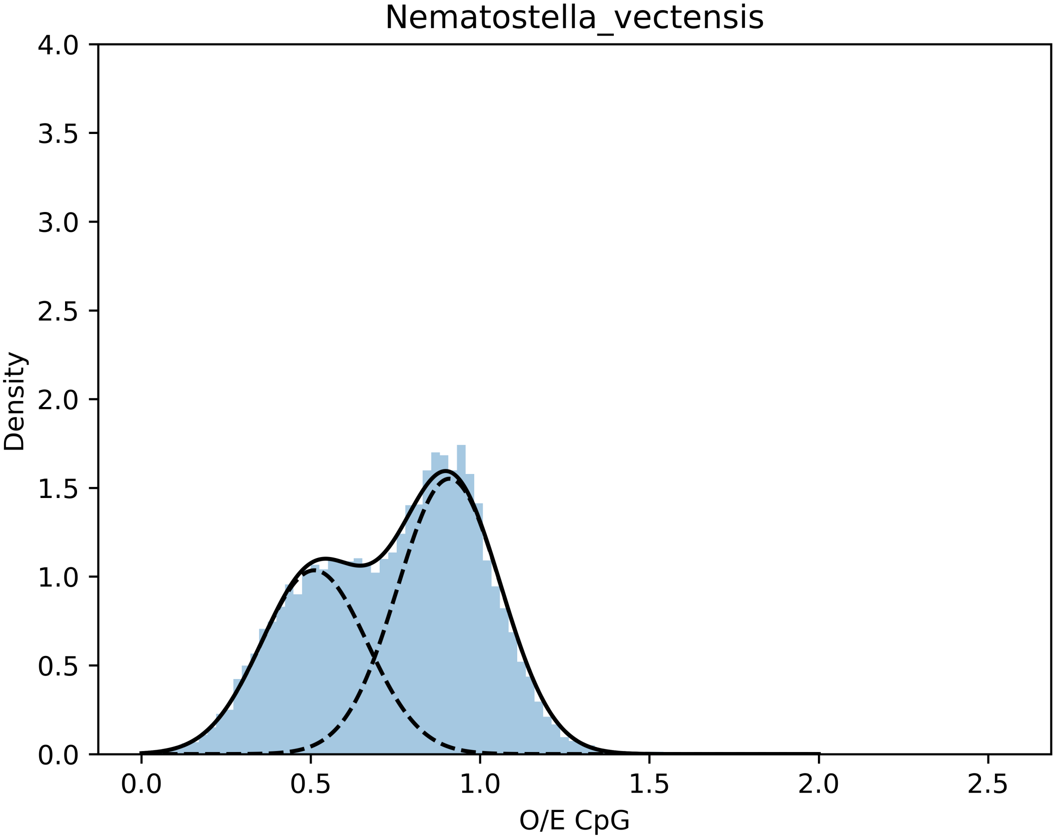

Nematostella_vectensis
Density
O/E CpG
0.0
0.5
1.0
1.5
2.0
2.5
3.0
3.5
4.0
0.0
0.5
1.0
1.5
2.0
2.5

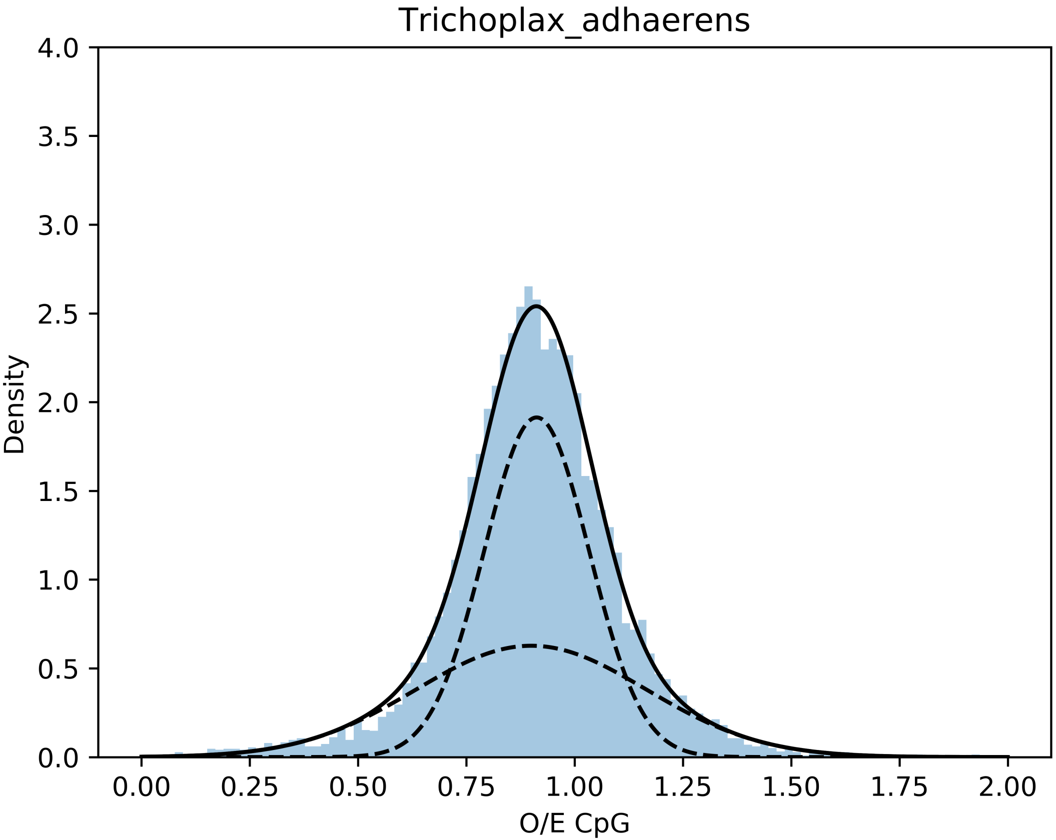
Trichoplax_adhaerens
Density
O/E CpG
0.0
0.5
1.0
1.5
2.0
2.5
3.0
3.5
4.0
0.00
0.25
0.50
0.75
1.00
1.25
1.50
1.75
2.00

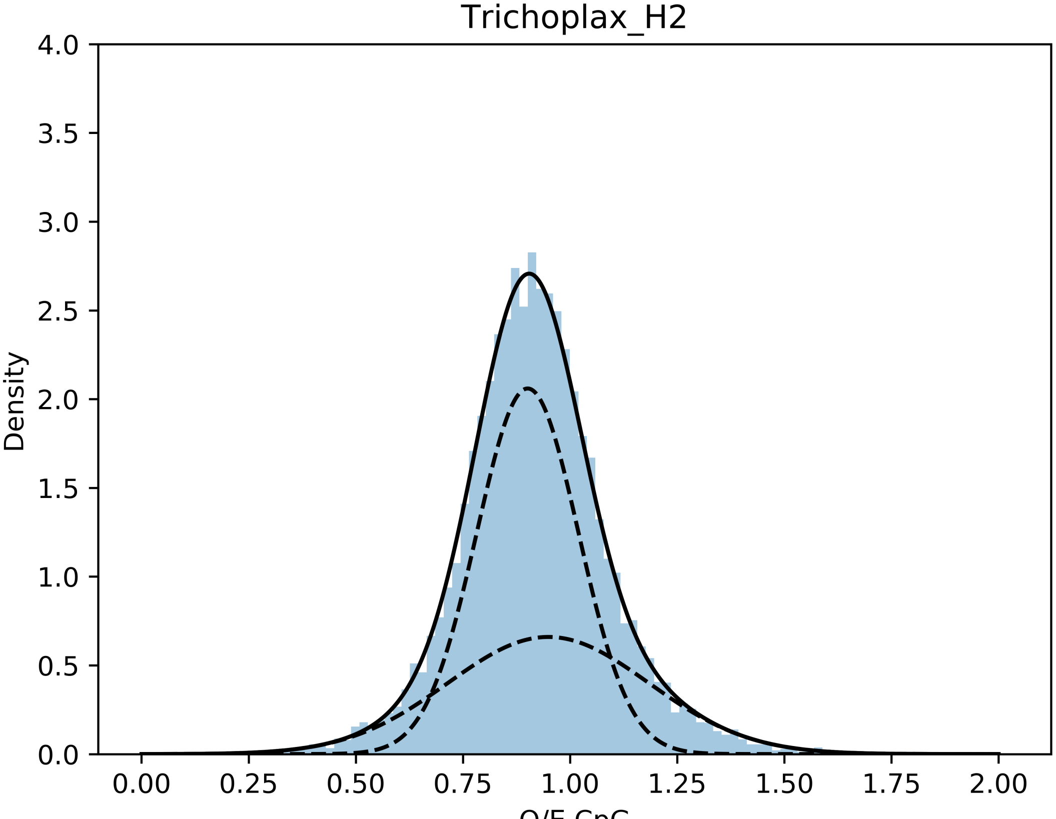
Trichoplax_H2
Density
O/E CpG
0.0
0.5
1.0
1.5
2.0
2.5
3.0
3.5
4.0
0.00
0.25
0.50
0.75
1.00
1.25
1.50
1.75
2.00

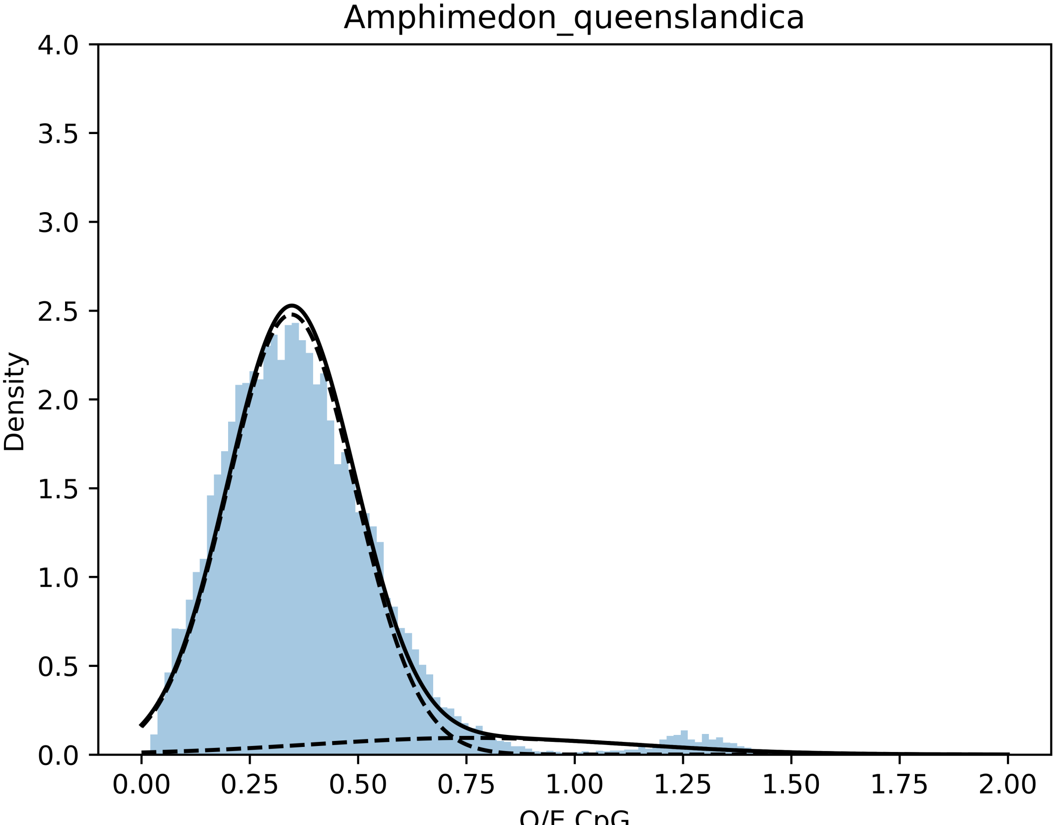
Amphimedon_queenslandica
Density
O/E CpG
0.0
0.5
1.0
1.5
2.0
2.5
3.0
3.5
4.0
0.00
0.25
0.50
0.75
1.00
1.25
1.50
1.75
2.00
