# Supplementary material for: Evolution of DNA Methylation Across Ecdysozoa
Source: J Mol Evol. 2022 Jan 28;90(1):56–72. doi: 10.1007/s00239-021-10042-0 (PMC8821070; doi:10.1007/s00239-021-10042-0)
Supplement: Supplementary file 2 — Additional file 2 (DOCX 1927 kb) [file 239_2021_10042_MOESM2_ESM.pdf]

Supporting Information

Additional Figures

Figure 1: Presence and absence of DNMT family members and prediction of DNA methylation status of all species analyzed.

Figure 2: Correlation of two parameters of the distrubtions resulting from Gaussian Mixture Modelling (GMM): mean of the component with the lower mean and the relative amount of data points in this components.

Figure 3: Correlation of two parameters of the distrubtions resulting from Gaussian Mixture Modelling (GMM): mean of the component with the higher mean and the relative amount of data points in the component with the lower mean.

Figure 4: Correlation of two parameters of the distrubtions resulting from Gaussian Mixture Modelling (GMM): distance of the means of both components and the standard deviation of the whole distribution.

Figure 5: Correlation of two parameters of the distrubtions resulting from Gaussian Mixture Modelling (GMM): distance of the means of both components and the relative amount of data points in the component with the lower mean.

Figure 6: Correlation of two parameters of the distrubtions resulting from Gaussian Mixture Modelling (GMM): distance of the means of both components and the mean of the whole distribution.

Figure: Due to their size, the graphical representation of the Gaussian Mixture Modelling for each species is stored in a separate file: Supplement-AddFig-CpG_Distributions.pdf

Additional Tables

Table 1: A list of all species analyzed and the source of the data.

Table 2: For all species a list of parameters of the Gaussian Mixture Modelling (GMM) and the resulting distributions.

Table 3: For all species a list of additional parameters of the Gaussian Mixture Modelling (GMM) and the resulting distributions.

Table 4: A comparison of our finding of the presence and absence of DNMT family members and prediction of DNA methylation status to existing literature in Nematoda.

Table 5: A comparison of our finding of the presence and absence of DNMT family members and prediction of DNA methylation status to existing literature in Arthropoda.

Table 7: A comparision of our finding of the presence and absence of DNMT family members and prediction of DNA methylation status to existing literature in Arthropoda, Nematoda and early-branching Metazoa.

Table 8: Overview of the classification of DNMT candidates according to the detected domains.

[1]To whom correspondence should be addressed.
jan.engelhardt@bioinf.uni-leipzig.de

Sequence data

The amino acid sequence of all predicted DNMT candidates is stored in the file 'DNMT-cands.fa'.

It can be downloaded as well: `www.bioinf.uni-leipzig.de/~jane/Engelhardt2020_DNMT-cands.fa`

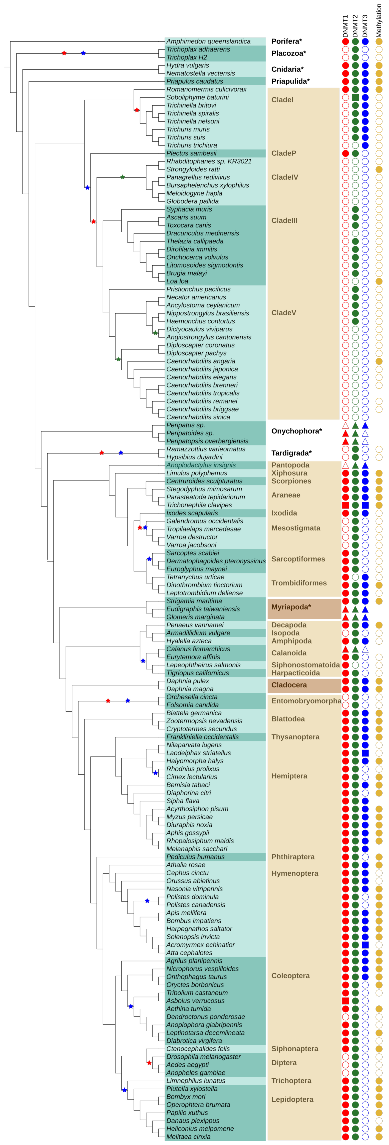

Figure 1: Presence and absence of DNMT family members in Metazoa indicated by filled and open symbols, respectively for DNMT1 (red), DNMT2 (green), and DNMT3 (blue). Data sources are indicated by symbol shape: proteome ◯, genome □, transcriptome △. The rightmost column (golden circles) shows the presence and absence of DNA methylation as predicted from the O/E CpG ratio. Absence of golden circle indicates missing data. The species list is given on turquoise background with alternating shades indicating the order membership. The name of the order (or suitable higher group marked with an asterisk *) is given in bold. Alternating shades of brown indicate (from top to bottom) Nematoda, Chelicerata, Myriapoda, Multicrustacea, Branchiopoda, and Hexapoda. Stars in the species tree denote proposed loss events inferred from absence of a DNMT in all species of a subtree comprising at least two leaves, disregarding absences in species with transcriptomic data only.

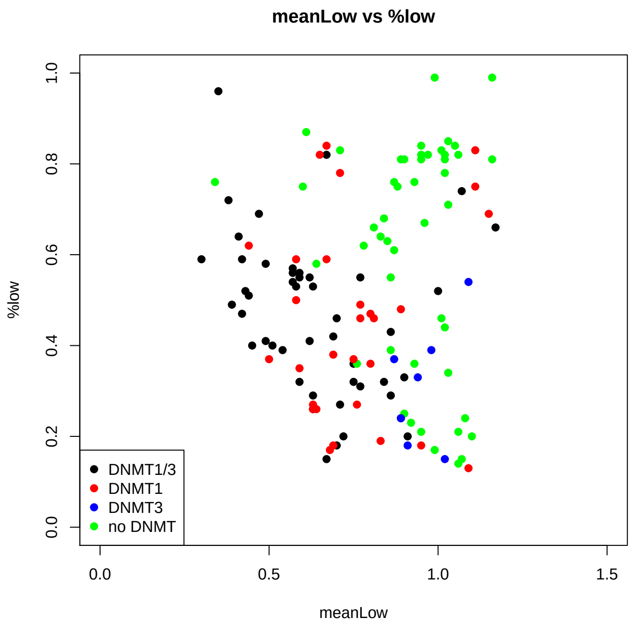

Figure 2: Each point shows one species analyzed by Gaussian Mixture Modelling (GMM). The x-axis is the mean of the component with the lower mean and the y-axis the relative amount of data points in this component. The values are shown in Tab. 2. The color represents if neither DNMT1 or DNMT3 (green) have been found in the species, only DNMT1 (red), only DNMT3 (blue) or both (black).

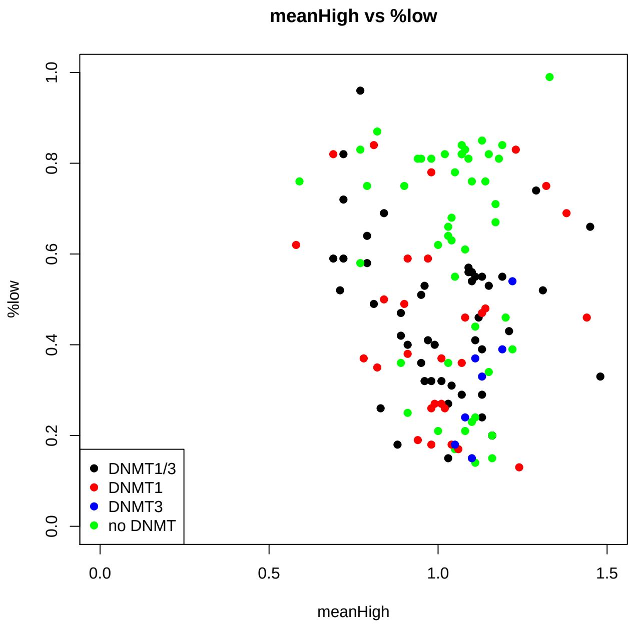

Figure 3: The x-axis is the mean of the component with the higher mean and the y-axis the relative amount of data points in the component with the lower mean. For more details see Fig. 2

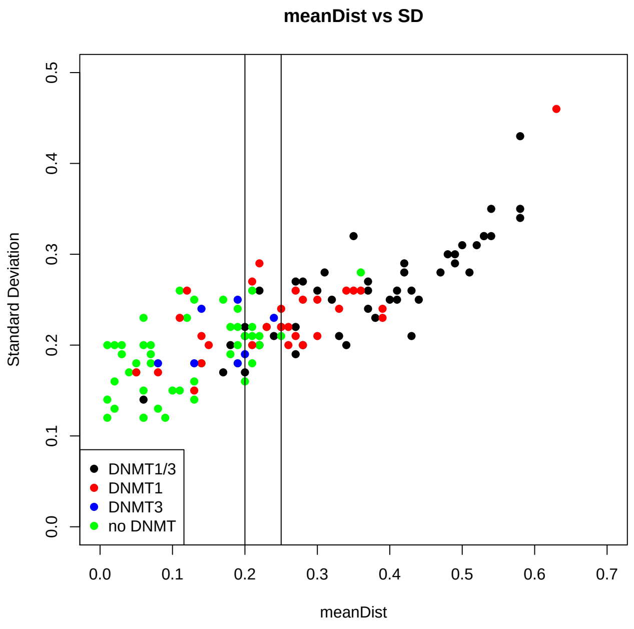

Figure 4: The x-axis is the distance of the means of both components and the y-axis the standard deviation (SD) of the whole distribution. For more details see Fig. 2

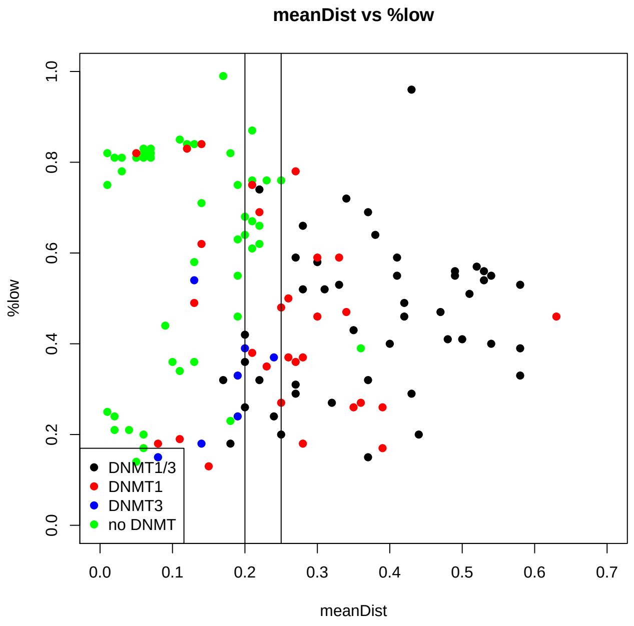

Figure 5: The x-axis is the distance of the means of both components and the y-axis the relative amount of data points in the component with the lower mean. For more details see Fig. 2

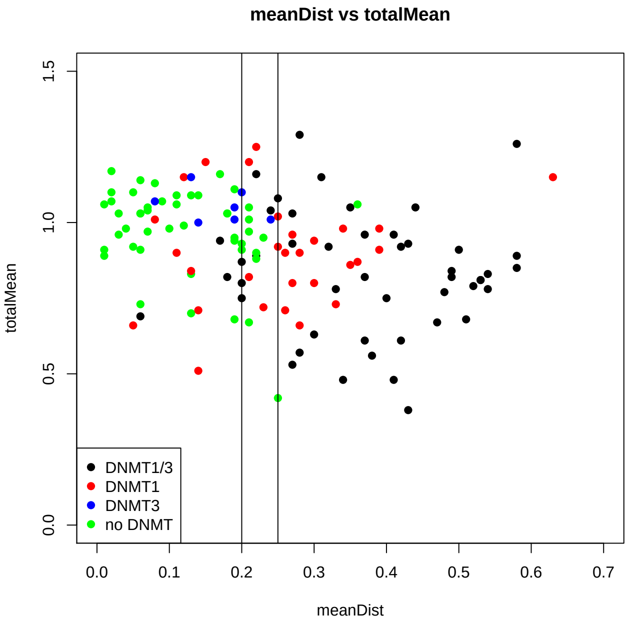

Figure 6: The x-axis is the distance of the means of both components and the y-axis the mean of the whole distribution. For more details see Fig. 2

Species	Database	Download link	N50
Drosophila melanogaster	N	`../GCF/000/001/215/GCF_000001215.4_Release_6_plus_ISO1_MT/GCF_000001215.4_Release_6_plus_ISO1_MT_protein.faa.gz`	25286936
Aedes aegypti	N	`../GCF/002/204/515/GCF_002204515.2_AaegL5.0/GCF_002204515.2_AaegL5.0_protein.faa.gz`	409777670
Anopheles gambiae	N	`../GCF/000/005/575/GCF_000005575.2_AgamP3/GCF_000005575.2_AgamP3_protein.faa.gz`	49364325
Ctenocephalides felis	N	`../GCF/003/426/905/GCF_003426905.1_ASM342690v1/GCF_003426905.1_ASM342690v1_protein.faa.gz`	71713785
Bombyx mori	N	`../GCF/000/151/625/GCF_000151625.1_ASM15162v1/GCF_000151625.1_ASM15162v1_protein.faa.gz`	4008358
Danaus plexippus	N	`../GCA/000/235/995/GCA_000235995.2_Dpv3/GCA_000235995.2_Dpv3_protein.faa.gz`	715714
Operophtera brumata	N	`../GCA/001/266/575/GCA_001266575.1_ASM126657v1/GCA_001266575.1_ASM126657v1_protein.faa.gz`	65630
Heliconius melpomene	E	`../heliconius_melpomene/pep/Heliconius_melpomene.Hmel1.pep.all.fa.gz`	194302
Melitaea cinxia	E	`../melitaea_cinxia/pep/Melitaea_cinxia.MelCinx1.0.pep.all.fa.gz`	119328
Papilio xuthus	N	`../GCF/000/836/235/GCF_000836235.1_Pxut_1.0/GCF_000836235.1_Pxut_1.0_protein.faa.gz`	6198915
Plutella xylostella	N	`../GCF/000/330/985/GCF_000330985.1_DBM_FJ_V1.1/GCF_000330985.1_DBM_FJ_V1.1_protein.faa.gz`	737182
Limnephilus lunatus	O	`http://download.lepbase.org/v4/sequence/Limnephilus_lunatus_v1_-_proteins.fa.gz`	54650
Agrilus planipennis	N	`../GCF/000/699/045/GCF_000699045.2_Apla_2.0/GCF_000699045.2_Apla_2.0_protein.faa.gz`	1113421
Nicrophorus vespilloides	N	`../GCF/001/412/225/GCF_001412225.1_Nicve_v1.0/GCF_001412225.1_Nicve_v1.0_protein.faa.gz`	122407
Onthophagus taurus	N	`../GCF/000/648/695/GCF_000648695.1_Otau_2.0/GCF_000648695.1_Otau_2.0_protein.faa.gz`	337157
Oryctes borbonicus	N	`../GCA/001/443/705/GCA_001443705.1_ASM144370v1/GCA_001443705.1_ASM144370v1_protein.faa.gz`	33367
Anoplophora glabripennis	N	`../GCF/000/390/285/GCF_000390285.2_Agla_2.0/GCF_000390285.2_Agla_2.0_protein.faa.gz`	678234
Leptinotarsa decemlineata	N	`../GCF/000/500/325/GCF_000500325.1_Ldec_2.0/GCF_000500325.1_Ldec_2.0_protein.faa.gz`	139046
Diabrotica virgifera	N	`../GCF/003/013/835/GCF_003013835.1_Dvir_v2.0/GCF_003013835.1_Dvir_v2.0_protein.faa.gz`	489108
Dendroctonus ponderosae	N	`../GCF/000/355/655/GCF_000355655.1_DendPond_male_1.0/GCF_000355655.1_DendPond_male_1.0_protein.faa.gz`	628732
Aethina tumida	N	`../GCF/001/937/115/GCF_001937115.1_Atum_1.0/GCF_001937115.1_Atum_1.0_protein.faa.gz`	298879
Tribolium castaneum	N	`../GCF/000/002/335/GCF_000002335.3_Tcas5.2/GCF_000002335.3_Tcas5.2_protein.faa.gz`	15265516
Asbolus verrucosus	N	`../GCA/004/193/795/GCA_004193795.1_BDFB_1.0/GCA_004193795.1_BDFB_1.0_protein.faa.gz`	5726
Apis mellifera	N	`../GCF/003/254/395/GCF_003254395.2_Amel_HAv3.1/GCF_003254395.2_Amel_HAv3.1_protein.faa.gz`	13619445
Bombus impatiens	N	`../GCF/000/188/095/GCF_000188095.3_BIMP_2.2/GCF_000188095.3_BIMP_2.2_protein.faa.gz`	1399493
Atta cephalotes	N	`../GCF/000/143/395/GCF_000143395.1_Attacep1.0/GCF_000143395.1_Attacep1.0_protein.faa.gz`	5154485
Acromyrmex echinatior	N	`../GCF/000/204/515/GCF_000204515.1_Aech_3.9/GCF_000204515.1_Aech_3.9_protein.faa.gz`	1110580
Harpegnathos saltator	N	`../GCF/003/227/715/GCF_003227715.1_Hsal_v8.5/GCF_003227715.1_Hsal_v8.5_protein.faa.gz`	1078644
Solenopsis invicta	N	`../GCF/000/188/075/GCF_000188075.2_Si_gnH/GCF_000188075.2_Si_gnH_protein.faa.gz`	621039
Polistes dominula	N	`../GCF/001/465/965/GCF_001465965.1_Pdom_r1.2/GCF_001465965.1_Pdom_r1.2_protein.faa.gz`	1625592
Polistes canadensis	N	`../GCF/001/313/835/GCF_001313835.1_ASM131383v1/GCF_001313835.1_ASM131383v1_protein.faa.gz`	521566
Nasonia vitripennis	N	`../GCF/000/002/325/GCF_000002325.3_Nvit_2.1/GCF_000002325.3_Nvit_2.1_protein.faa.gz`	897131
Cephus cinctu	N	`../GCF/000/341/935/GCF_000341935.1_Ccin1/GCF_000341935.1_Ccin1_protein.faa.gz`	622163
Orussus abietinus	N	`../GCF/000/612/105/GCF_000612105.2_Oabi_2.0/GCF_000612105.2_Oabi_2.0_protein.faa.gz`	612083
Athalia rosae	N	`../GCF/000/344/095/GCF_000344095.2_Aros_2.0/GCF_000344095.2_Aros_2.0_protein.faa.gz`	943070
Pediculus humanus	N	`../GCF/000/006/295/GCF_000006295.1_JCVI_LOUSE_1.0/GCF_000006295.1_JCVI_LOUSE_1.0_protein.faa.gz`	497057
Nilaparvata lugens	N	`../GCF/000/757/685/GCF_000757685.1_NilLug1.0/GCF_000757685.1_NilLug1.0_protein.faa.gz`	356597
Laodelphax striatellus	N	`../GCA/003/335/185/GCA_003335185.2_ASM333518v2/GCA_003335185.2_ASM333518v2_protein.faa.gz`	1084798
Rhodnius prolixus	E	`../rhodnius_prolixus/pep/Rhodnius_prolixus.RproC3.pep.all.fa.gz`	1088772

Cimex lectularius	N	../GCF/000/648/675/GCF_000648675.2_Clec_2.1/GCF_000648675.2_Clec_2.1_protein.faa.gz	1637644
Halyomorpha halys	N	../GCF/000/696/795/GCF_000696795.2_Hhal_2.0/GCF_000696795.2_Hhal_2.0_protein.faa.gz	393089
Bemisia tabaci	N	../GCF/001/854/935/GCF_001854935.1_ASM185493v1/GCF_001854935.1_ASM185493v1_protein.faa.gz	3232964
Melanaphis sacchari	N	../GCF/002/803/265/GCF_002803265.2_SCAv2.0/GCF_002803265.2_SCAv2.0_protein.faa.gz	3012626
Aphis gossypii	N	../GCF/004/010/815/GCF_004010815.1_ASM401081v1/GCF_004010815.1_ASM401081v1_protein.faa.gz	437960
Rhopalosiphum maidis	N	../GCF/003/676/215/GCF_003676215.2_ASM367621v3/GCF_003676215.2_ASM367621v3_protein.faa.gz	93298903
Acyrthosiphon pisum	N	../GCF/005/508/785/GCF_005508785.1_pea_aphid_22Mar2018_4r6ur/GCF_005508785.1_pea_aphid_22Mar2018_4r6ur_protein.faa.gz	132544852
Diuraphis noxia	N	../GCF/001/186/385/GCF_001186385.1_Dnoxia_1.0/GCF_001186385.1_Dnoxia_1.0_protein.faa.gz	397774
Myzus persicae	N	../GCF/001/856/785/GCF_001856785.1_MPER_G0061.0/GCF_001856785.1_MPER_G0061.0_protein.faa.gz	435781
Sipha flava	N	../GCF/003/268/045/GCF_003268045.1_YSA_version1/GCF_003268045.1_YSA_version1_protein.faa.gz	1686648
Diaphorina citri	N	../GCF/000/475/195/GCF_000475195.1_Diaci_psyllid_genome_assembly_version_1.1/ GCF_000475195.1_Diaci_psyllid_genome_assembly_version_1.1_protein.faa.gz	109898
Frankliniella occidentalis	N	../GCF/000/697/945/GCF_000697945.2_Focc_2.1/GCF_000697945.2_Focc_2.1_protein.faa.gz	438040
Zootermopsis nevadensis	N	../GCF/000/696/155/GCF_000696155.1_ZooNev1.0/GCF_000696155.1_ZooNev1.0_protein.faa.gz	751105
Cryptotermes secundus	N	../GCF/002/891/405/GCF_002891405.2_Csec_1.0/GCF_002891405.2_Csec_1.0_protein.faa.gz	1184893
Blattela germanica	N	../GCA/003/018/175/GCA_003018175.1_Bger_1.1/GCA_003018175.1_Bger_1.1_protein.faa.gz	1056071
Orchesella cincta	N	../GCA/001/718/145/GCA_001718145.1_ASM171814v1/GCA_001718145.1_ASM171814v1_protein.faa.gz	65879
Folsomia candida	N	../GCF/002/217/175/GCF_002217175.1_ASM221717v1/GCF_002217175.1_ASM221717v1_protein.faa.gz	6519406
Daphnia pulex	N	../GCA/000/187/875/GCA_000187875.1_V1.0/GCA_000187875.1_V1.0_protein.faa.gz	642089
Daphnia magna	E	../daphnia_magna/pep/Daphnia_magna.daphmag2.4.pep.all.fa.gz	397658
Tigriopus californicus	N	../GCA/007/210/705/GCA_007210705.1_Tcal_SD_v2.1/GCA_007210705.1_Tcal_SD_v2.1_protein.faa.gz	15806032
Lepeophtheirus salmonis	E	ftp://ftp.ensemblgenomes.org/pub/metazoa/release-43/fasta/ lepeophtheirus_salmonis/pep/Lepeophtheirus_salmonis.LSalAtl2s.pep.all.fa.gz	478276
Eurytemora affinis	N	../GCF/000/591/075/GCF_000591075.1_Eaff_2.0/GCF_000591075.1_Eaff_2.0_protein.faa.gz	252275
Calanus finmarchicus	O	https://datadryad.org/stash/dataset/doi:10.5061/dryad.293kp3d	n/a
Hyalella azteca	N	../GCF/000/764/305/GCF_000764305.1_Hazt_2.0/GCF_000764305.1_Hazt_2.0_protein.faa.gz	215427
Armadillidium vulgare	N	../GCA/004/104/545/GCA_004104545.1_Arma_vul_BF2787/GCA_004104545.1_Arma_vul_BF2787_protein.faa.gz	51088
Penaeus vannamei	N	../GCF/003/789/085/GCF_003789085.1_ASM378908v1/GCF_003789085.1_ASM378908v1_protein.faa.gz	605555
Glomeris marginata	O	https://datadryad.org/stash/dataset/doi:10.5061/dryad.293kp3d	n/a
Eudigraphis taiwaniensis	O	https://datadryad.org/stash/dataset/doi:10.5061/dryad.293kp3d	n/a
Strigamia maritima	E	ftp://ftp.ensemblgenomes.org/pub/release-44/metazoa/fasta/strigamia_maritima/pep/Strigamia_maritima.Smar1.pep.all.fa.gz	139451
Leptotrombidium deliense	N	../GCA/003/675/905/GCA_003675905.1_ASM367590v1/GCA_003675905.1_ASM367590v1_protein.faa.gz	2941
Dinothrombium tinctorium	N	../GCA/003/675/995/GCA_003675995.1_ASM367599v1/GCA_003675995.1_ASM367599v1_protein.faa.gz	16512
Tetranychus urticae	N	../GCF/000/239/435/GCF_000239435.1_ASM23943v1/GCF_000239435.1_ASM23943v1_protein.faa.gz	2993488
Euroglyphus maynei	N	../GCA/002/135/145/GCA_002135145.1_EurM1.0/GCA_002135145.1_EurM1.0_protein.faa.gz	788
Dermatophagoides pteronyssinus	N	../GCF/001/901/225/GCF_001901225.1_ASM190122v2/GCF_001901225.1_ASM190122v2_protein.faa.gz	450436
Sarcoptes scabiei	N	../GCA/000/828/355/GCA_000828355.1_SarSca1.0/GCA_000828355.1_SarSca1.0_protein.faa.gz	11557
Varroa destructor	N	../GCF/002/443/255/GCF_002443255.1_Vdes_3.0/GCF_002443255.1_Vdes_3.0_protein.faa.gz	58536683
Varroa jacobsoni	N	../GCF/002/532/875/GCF_002532875.1_vjacob_1.0/GCF_002532875.1_vjacob_1.0_protein.faa.gz	233810
Tropilaelaps mercedesae	N	../GCA/002/081/605/GCA_002081605.1_T._mercedesae_v01/GCA_002081605.1_T._mercedesae_v01_protein.faa.gz	28859
Galendromus occidentalis	N	../GCF/000/255/335/GCF_000255335.1_Mocc_1.0/GCF_000255335.1_Mocc_1.0_protein.faa.gz	896831

Ixodes scapularis	N	../GCF/002/892/825/GCF_002892825.2_ISE6_asm2.2_deduplicated/GCF_002892825.2_ISE6_asm2.2_deduplicated_protein.faa.gz	835681
Trichonephila clavipes	N	../GCA/002/102/615/GCA_002102615.1_NepCla1.0/GCA_002102615.1_NepCla1.0_protein.faa.gz	62959
Parasteatoda tepidariorum	N	../GCF/000/365/465/GCF_000365465.2_Ptep_2.0/GCF_000365465.2_Ptep_2.0_protein.faa.gz	4055356
Stegodyphus mimosarum	N	../GCA/000/611/955/GCA_000611955.2_Stegodyphus_mimosarum_v1/GCA_000611955.2_Stegodyphus_mimosarum_v1_protein.faa.gz	480636
Centruroides sculpturatus	N	../GCF/000/671/375/GCF_000671375.1_Cexi_2.0/GCF_000671375.1_Cexi_2.0_protein.faa.gz	537465
Limulus polyphemus	N	../GCF/000/517/525/GCF_000517525.1_Limulus_polyphemus-2.1.2/GCF_000517525.1_Limulus_polyphemus-2.1.2_protein.faa.gz	254089
Anoplodactylus insignis	O	https://datadryad.org/stash/dataset/doi:10.5061/dryad.293kp3d	n/a
Hypsibius dujardini	N	../GCA/002/082/055/GCA_002082055.1_nHd_3.1/GCA_002082055.1_nHd_3.1_protein.faa.gz	342180
Ramazzottius varieornatus	N	../GCA/001/949/185/GCA_001949185.1_Rvar_4.0/GCA_001949185.1_Rvar_4.0_protein.faa.gz	4740345
Peripatopsis overbergiensis	O	https://datadryad.org/stash/dataset/doi:10.5061/dryad.293kp3d	n/a
Peripatoides sp	O	https://datadryad.org/stash/dataset/doi:10.5061/dryad.293kp3d	n/a
Peripatus sp	O	https://datadryad.org/stash/dataset/doi:10.5061/dryad.293kp3d	n/a
Caenorhabditis sinica	W	../caenorhabditis_sinica/PRJNA194557/caenorhabditis_sinica.PRJNA194557.WBPS14.protein.fa.gz	25228
Caenorhabditis briggsae	O	ftp://ftp.wormbase.org/pub/wormbase/releases/WS271/species/c_briggsae/PRJNA10731/c_briggsae.PRJNA10731.WS271.protein.fa.gz	17485439
Caenorhabditis remanei	W	../caenorhabditis_remanei/PRJNA248909/caenorhabditis_remanei.PRJNA248909.WBPS14.protein.fa.gz	1522088
Caenorhabditis tropicalis	W	../caenorhabditis_tropicalis/PRJNA53597/caenorhabditis_tropicalis.PRJNA53597.WBPS14.protein.fa.gz	20921866
Caenorhabditis brenneri	W	../caenorhabditis_brenneri/PRJNA20035/caenorhabditis_brenneri.PRJNA20035.WBPS14.protein.fa.gz	381961
Caenorhabditis elegans	O	ftp://ftp.wormbase.org/pub/wormbase/releases/WS271/species/c_elegans/PRJNA13758/c_elegans.PRJNA13758.WS271.protein.fa.gz	17493829
Caenorhabditis japonica	O	ftp://ftp.wormbase.org/pub/wormbase/releases/WS271/species/c_japonica/PRJNA12591/c_japonica.PRJNA12591.WS271.protein.fa.gz	94149
Caenorhabditis angaria	W	../caenorhabditis_angaria/PRJNA51225/caenorhabditis_angaria.PRJNA51225.WBPS14.protein.fa.gz	79858
Diploscapter pachys	W	../diploscapter_pachys/PRJNA280107/diploscapter_pachys.PRJNA280107.WBPS14.protein.fa.gz	124241
Diploscapter coronatus	W	../diploscapter_coronatus/PRJDB3143/diploscapter_coronatus.PRJDB3143.WBPS14.protein.fa.gz	1007652
Angiostrongylus cantonensis	W	../angiostrongylus_cantonensis/PRJEB493/angiostrongylus_cantonensis.PRJEB493.WBPS14.protein.fa.gz	43900
Dictyocaulus viviparus	W	../dictyocaulus_viviparus/PRJNA72587/dictyocaulus_viviparus.PRJNA72587.WBPS14.protein.fa.gz	225748
Haemonchus contortus	W	../haemonchus_contortus/PRJEB506/haemonchus_contortus.PRJEB506.WBPS14.protein.fa.gz	47382676
Nippostrongylus brasiliensis	W	../nippostrongylus_brasiliensis/PRJEB511/nippostrongylus_brasiliensis.PRJEB511.WBPS14.protein.fa.gz	33527
Ancylostoma ceylanicum	W	../ancylostoma_ceylanicum/PRJNA231479/ancylostoma_ceylanicum.PRJNA231479.WBPS14.protein.fa.gz	668412
Necator americanus	W	../necator_americanus/PRJNA72135/necator_americanus.PRJNA72135.WBPS14.protein.fa.gz	211861
Pristionchus pacificus	O	ftp://ftp.wormbase.org/pub/wormbase/releases/WS271/species/p_pacificus/PRJNA12644/p_pacificus.PRJNA12644.WS271.protein.fa.gz	23915096
Loa loa	W	../loa_loa/PRJNA246086/loa_loa.PRJNA246086.WBPS14.protein.fa.gz	180288
Brugia malayi	O	ftp://ftp.wormbase.org/pub/wormbase/releases/WS271/species/b_malayi/PRJNA10729/b_malayi.PRJNA10729.WS271.protein.fa.gz	14214749
Litomosoides sigmodontis	W	../litomosoides_sigmodontis/PRJEB3075/litomosoides_sigmodontis.PRJEB3075.WBPS14.protein.fa.gz	45863
Onchocerca volvulus	O	ftp://ftp.wormbase.org/pub/wormbase/releases/WS271/species/o_volvulus/PRJEB513/o_volvulus.PRJEB513.WS271.protein.fa.gz	25485961
Dirofilaria immitis	W	../dirofilaria_immitis/PRJEB1797/dirofilaria_immitis.PRJEB1797.WBPS14.protein.fa.gz	71281
Thelazia callipaeda	W	../thelazia_callipaeda/PRJEB1205/thelazia_callipaeda.PRJEB1205.WBPS14.protein.fa.gz	51228
Dracunculus medinensis	W	../dracunculus_medinensis/PRJEB500/dracunculus_medinensis.PRJEB500.WBPS14.protein.fa.gz	665026
Toxocara canis	W	../toxocara_canis/PRJEB533/toxocara_canis.PRJEB533.WBPS14.protein.fa.gz	31192
Ascaris suum	W	../ascaris_suum/PRJNA62057/ascaris_suum.PRJNA62057.WBPS14.protein.fa.gz	4646302
Syphacia muris	W	../syphacia_muris/PRJEB524/syphacia_muris.PRJEB524.WBPS14.protein.fa.gz	60730
Globodera pallida	W	../globodera_pallida/PRJEB123/globodera_pallida.PRJEB123.WBPS14.protein.fa.gz	120481
Meloidogyne hapla	W	../meloidogyne_hapla/PRJNA29083/meloidogyne_hapla.PRJNA29083.WBPS14.protein.fa.gz	37608

Bursaphelenchus xylophilus	W	../bursaphelenchus_xylophilus/PRJEA64437/bursaphelenchus_xylophilus.PRJEA64437.WBPS14.protein.fa.gz	949830
Panagrellus redivivus	W	../panagrellus_redivivus/PRJNA186477/panagrellus_redivivus.PRJNA186477.WBPS14.protein.fa.gz	262414
Strongyloides ratti	O	ftp://ftp.wormbase.org/pub/wormbase/releases/WS271/species/s_ratti/PRJEB125/s_ratti.PRJEB125.WS271.protein.fa.gz	11693564
Rhabditophanes sp.	W	../rhabditophanes_kr3021/PRJEB1297/rhabditophanes_kr3021.PRJEB1297.WBPS14.protein.fa.gz	537195
Plectus sambesii	W	../plectus_sambesii/PRJNA390260/plectus_sambesii.PRJNA390260.WBPS14.protein.fa.gz	23450
Trichuris trichiura	W	../trichuris_trichiura/PRJEB535/trichuris_trichiura.PRJEB535.WBPS14.protein.fa.gz	70602
Trichuris suis	W	../trichuris_suis/PRJNA179528/trichuris_suis.PRJNA179528.WBPS14.protein.fa.gz	1322386
Trichuris muris	O	ftp://ftp.wormbase.org/pub/wormbase/releases/WS271/species/t_muris/PRJEB126/t_muris.PRJEB126.WS271.protein.fa.gz	28941788
Trichinella nelsoni	W	../trichinella_nelsoni/PRJNA257433/trichinella_nelsoni.PRJNA257433.WBPS14.protein.fa.gz	293867
Trichinella spiralis	W	../trichinella_spiralis/PRJNA12603/trichinella_spiralis.PRJNA12603.WBPS14.protein.fa.gz	6373445
Trichinella britovi	W	../trichinella_britovi/PRJNA257433/trichinella_britovi.PRJNA257433.WBPS14.protein.fa.gz	147150
Soboliphyme baturini	W	../soboliphyme_baturini/PRJEB516/soboliphyme_baturini.PRJEB516.WBPS14.protein.fa.gz	19774
Romanomermis culicivorax	W	../romanomermis_culicivorax/PRJEB1358/romanomermis_culicivorax.PRJEB1358.WBPS14.protein.fa.gz	17632
Priapulus caudatus	N	../GCF/000/485/595/GCF_000485595.1_Priapulus_caudatus-5.0.1/GCF_000485595.1_Priapulus_caudatus-5.0.1_protein.faa.gz	209727
Nematostella vectensis	N	../GCF/000/209/225/GCF_000209225.1_ASM20922v1/GCF_000209225.1_ASM20922v1_protein.faa.gz	472588
Hydra vulgaris	N	../GCF/000/004/095/GCF_000004095.1_Hydra_RP_1.0/GCF_000004095.1_Hydra_RP_1.0_protein.faa.gz	96317
Trichoplax adhaerens	N	../GCF/000/150/275/GCF_000150275.1_v1.0/GCF_000150275.1_v1.0_protein.faa.gz	5978658
Trichoplax H2	N	../GCA/003/344/405/GCA_003344405.1_TrispH2_1.0/GCA_003344405.1_TrispH2_1.0_protein.faa.gz	376320
Amphimedon queenslandica	N	../GCF/000/090/795/GCF_000090795.1_v1.0/GCF_000090795.1_v1.0_protein.faa.gz	120365

Table 1: The given url is the link to the predicted protein data. The abbreviations in the second colum stand for the following databases: N - NCBI, W - Wormbase, E - ENSEMBL, O - Other. In case of datadryad.org no additional data was available. For the NCBI, Wormbase and ENSEMBL, genome and CDS data was downloaded for the same version of the respective species. The N50 value in the last column corresponds to the respective genome assembly. Due to their length some URL are shortened. If they are from one of the following database they start with the given address: NCBI - ftp://ftp.ncbi.nlm.nih.gov/genomes/all/..; ENSEMBL - ftp://ftp.ensemblgenomes.org/pub/metazoa/release-44/fasta/..; Wormbase ParaSite - ftp://ftp.ebi.ac.uk/pub/databases/wormbase/parasite/releases/WBPS14/species/..;

	meanLow	meanHigh	meanDist	%low	%high	AIC-N1	AIC-N2	best-N
Drosophila melanogaster R	0.8821	0.8969	0.0148	0.75	0.25	-40614.0	-42101.0	2
Drosophila melanogaster S	0.9977	1.0044	0.0068	0.81	0.19	-59169.0	-63335.0	2
Aedes aegypti R	0.9909	1.0483	0.0574	0.17	0.83	-38714.0	-40625.0	2
Aedes aegypti S	0.9976	1.0047	0.0071	0.76	0.24	-56734.0	-58786.0	1
Anopheles gambiae R	1.0761	1.1576	0.0815	0.15	0.85	-18272.0	-20001.0	2
Anopheles gambiae S	0.9714	1.0248	0.0534	0.43	0.57	-25799.0	-25744.0	2
Ctenocephalides felis R	0.5807	0.9071	0.3264	0.59	0.41	-520.0	-1282.0	2
Ctenocephalides felis S	0.9923	1.0114	0.0191	0.78	0.22	-23484.0	-24789.0	2
Bombyx mori R	0.6792	1.0644	0.3852	0.17	0.83	-1612.0	-4685.0	2
Bombyx mori S	0.9627	1.0333	0.0707	0.47	0.53	-33312.0	-33240.0	2
Danaus plexippus R	0.8341	0.9409	0.1068	0.18	0.82	-2117.0	-2726.0	2
Danaus. plexippus S	0.9943	1.002	0.0077	0.77	0.23	-15771.0	-17469.0	2
Operophtera brumata R	0.7509	1.0148	0.2639	0.37	0.63	-3391.0	-3449.0	2
Operophtera brumata S	0.9947	1.0064	0.0117	0.79	0.21	-21101.0	-22342.0	2
Heliconius melpomene R	0.6317	0.989	0.3573	0.27	0.73	1566.0	1151.0	2
Heliconius melpomene S	0.9968	1.0028	0.006	0.77	0.23	-13385.0	-14744.0	2
Melitaea cinxia R	0.81	1.0781	0.2682	0.38	0.62	2936.0	3054.0	1
Melitaea cinxia S	0.9895	1.0138	0.0243	0.78	0.22	-12302.0	-13517.0	2
Papilio xuthus R	0.635	1.0218	0.3869	0.26	0.74	288.0	-2301.0	2
Papilio xuthus S	0.9957	1.0046	0.0088	0.77	0.23	-30799.0	-33352.0	2
Plutella xylostella R	0.6925	0.9776	0.2851	0.18	0.82	-8635.0	-11257.0	2
Plutella xylostella S	0.9972	1.0036	0.0064	0.79	0.21	-37885.0	-40107.0	2
Limnephilus lunatus R	0.712	0.97	0.258	0.76	0.24	-3676.0	-4244.0	2
Limnephilus lunatus S	0.9504	1.0483	0.0979	0.55	0.45	-14309.0	-14261.0	1
Agrilus planipennis R	0.5931	0.9587	0.3656	0.33	0.67	3379.0	2937.0	2
Agrilus planipennis S	0.9964	1.0078	0.0114	0.78	0.22	-25718.0	-27605.0	2
Nicrophorus vespilloides R	0.6703	1.0345	0.3642	0.15	0.85	-1041.0	-4283.0	2
Nicrophorus vespilloides S	0.9975	1.0036	0.0062	0.79	0.21	-30183.0	-32165.0	2
Onthophagus taurus R	0.9982	1.3052	0.307	0.52	0.48	6428.0	6569.0	1
Onthophagus taurus S	0.9934	1.0072	0.0138	0.78	0.22	-23845.0	-25528.0	2
Oryctes borbonicus R	0.8902	1.1407	0.2505	0.48	0.52	-1326.0	-1265.0	1
Oryctes borbonicus S	0.9488	1.0539	0.1051	0.61	0.39	-9392.0	-9371.0	1
Anoplophora glabripennis R	0.6929	0.9067	0.2138	0.37	0.63	-8719.0	-8579.0	1
Anoplophora glabripennis S	0.995	1.0097	0.0147	0.79	0.21	-26064.0	-28138.0	2
Leptinotarsa decemlineata R	0.5782	0.8382	0.26	0.5	0.5	-6981.0	-6930.0	1
Leptinotarsa decemlineata S	0.9581	1.0483	0.0902	0.61	0.39	-22738.0	-22671.0	1

Diabrotica virgifera R	0.5855	0.8197	0.2342	0.36	0.64	-4880.0	-4618.0	1
Diabrotica virgifera S	0.9915	1.0111	0.0196	0.78	0.22	-24750.0	-26712.0	2
Dendroctonus ponderosae R	0.7556	0.8908	0.1352	0.34	0.66	-21172.0	-21158.0	1
Dendroctonus ponderosae S	0.965	1.0372	0.0722	0.62	0.38	-32452.0	-32420.0	1
Aethina tumida R	0.6285	0.9818	0.3533	0.26	0.74	2109.0	1244.0	2
Aethina tumida S	0.9644	1.0443	0.0799	0.67	0.33	-23733.0	-23693.0	1
Tribolium castaneum R	0.9525	1.0383	0.0859	0.17	0.83	-16000.0	-16764.0	2
Tribolium castaneum S	0.9967	1.0041	0.0074	0.76	0.24	-36402.0	-38548.0	2
Apis mellifera R	0.5419	1.1252	0.5833	0.39	0.61	16718.0	13973.0	2
Apis mellifera S	0.9969	1.0047	0.0078	0.78	0.22	-28545.0	-32653.0	2
Bombus impatiens R	0.6185	1.1139	0.4954	0.41	0.59	11622.0	9929.0	2
Bombus impatiens S	0.9601	1.032	0.0719	0.37	0.63	-33530.0	-33435.0	1
Atta cephalotes R	0.8562	1.1293	0.2731	0.29	0.71	-2254.0	-2341.0	2
Atta cephalotes S	0.9973	1.0031	0.0057	0.76	0.24	-13923.0	-14731.0	2
Acromyrmex echinatior R	0.8943	1.1276	0.2333	0.25	0.75	-5621.0	-5783.0	2
Acromyrmex echinatior S	0.9967	1.0086	0.012	0.77	0.23	-28553.0	-30530.0	2
Harpegnathos saltator R	0.7203	1.1555	0.4352	0.2	0.8	1321.0	-3135.0	2
Harpegnathos saltator S	0.9971	1.0019	0.0049	0.78	0.22	-40703.0	-45261.0	2
Solenopsis invicta R	0.9069	1.159	0.2521	0.2	0.8	-4006.0	-4652.0	2
Solenopsis invicta S	0.9983	1.0048	0.0065	0.77	0.23	-33840.0	-37296.0	2
Polistes dominula R	0.7716	1.0758	0.3042	0.46	0.54	725.0	817.0	1
Polistes dominula S	0.996	1.0064	0.0105	0.78	0.22	-25826.0	-27854.0	2
Polistes canadensis R	0.7951	1.1347	0.3396	0.47	0.53	2243.0	2267.0	1
Polistes canadensis S	0.9952	1.0043	0.0091	0.77	0.23	-24609.0	-26526.0	2
Nasonia vitripennis R	0.6322	1.0678	0.4356	0.29	0.71	3075.0	139.0	2
Nasonia vitripennis S	0.9987	1.0042	0.0056	0.77	0.23	-37434.0	-41106.0	2
Cephus cinctu R	0.7523	0.9752	0.2228	0.32	0.68	-10574.0	-10516.0	1
Cephus cinctu S	0.9679	1.0305	0.0627	0.56	0.44	-51870.0	-51789.0	1
Orussus abietinus R	0.8359	1.0057	0.1698	0.32	0.68	-14136.0	-14074.0	1
Orussus abietinus S	0.997	1.0059	0.0089	0.8	0.2	-33577.0	-35864.0	2
Athalia rosae R	0.7706	1.1847	0.4142	0.55	0.45	2890.0	1136.0	2
Athalia rosae S	0.9668	1.0348	0.0679	0.56	0.44	-38291.0	-38188.0	1
Pediculus humanus R	0.8129	1.4414	0.6285	0.46	0.54	13942.0	13839.0	2
Pediculus humanus S	0.9973	1.0085	0.0112	0.79	0.21	-6993.0	-8183.0	2
Nilaparvata lugens R	0.6324	0.8293	0.1969	0.26	0.74	-7964.0	-8114.0	2
Nilaparvata lugens S	0.9968	1.0015	0.0047	0.76	0.24	-33249.0	-35331.0	2
Laodelphax striatellus R	0.6916	0.8939	0.2023	0.42	0.58	-3304.0	-3180.0	1
Laodelphax striatellus S	0.9971	1.0104	0.0133	0.77	0.23	-20164.0	-21803.0	2

Rhodnius prolixus R	0.6692	0.8115	0.1423	0.84	0.16	-4706.0	-5951.0	2
Rhodnius prolixus S	0.9941	1.0041	0.01	0.78	0.22	-8638.0	-10053.0	2
Cimex lectularius R	0.6679	0.9707	0.3028	0.59	0.41	-6116.0	-6551.0	2
Cimex lectularius S	0.9952	1.0068	0.0116	0.78	0.22	-37178.0	-38860.0	2
Halyomorpha halys R	0.4156	0.6886	0.273	0.6	0.4	-11942.0	-13129.0	2
Halyomorpha halys S	0.9937	1.0097	0.0159	0.79	0.21	-31459.0	-33333.0	2
Bemisia tabaci R	0.6263	0.9585	0.3322	0.53	0.47	-6507.0	-7936.0	2
Bemisia tabaci S	0.9967	1.0039	0.0072	0.77	0.23	-35368.0	-37439.0	2
Aphis gossypii R	0.5746	1.1019	0.5273	0.56	0.44	9959.0	7048.0	2
Aphis gossypii S	0.9954	1.0124	0.017	0.79	0.21	-18291.0	-19944.0	2
Rhopalosiphum maidis R	0.5866	1.1268	0.5402	0.55	0.45	10692.0	7192.0	2
Rhopalosiphum maidis S	0.9926	1.009	0.0164	0.81	0.19	-21714.0	-23492.0	2
Acyrthosiphon pisum R	0.6174	1.1094	0.492	0.55	0.45	12100.0	8805.0	2
Acyrthosiphon pisum S	0.9946	1.0098	0.0152	0.78	0.22	-27606.0	-30418.0	2
Diuraphis noxia R	0.5685	1.0885	0.52	0.57	0.43	8249.0	5188.0	2
Diuraphis noxia S	0.9945	1.0088	0.0143	0.79	0.21	-16297.0	-18701.0	2
Myzus persicae R	0.5943	1.0882	0.4939	0.56	0.44	9372.0	5890.0	2
Myzus persicae S	0.995	1.0032	0.0082	0.75	0.25	-25357.0	-27604.0	2
Diaphorina citri R	0.492	0.7772	0.2852	0.36	0.64	2088.0	2300.0	1
Diaphorina citri S	0.9548	1.0506	0.0959	0.63	0.37	-27216.0	-27160.0	1
Frankliniella occidentalis R	0.4363	0.9468	0.5105	0.51	0.49	6820.0	-4511.0	2
Frankliniella occidentalis S	0.9978	1.0045	0.0067	0.78	0.22	-42812.0	-45274.0	2
Zootermopsis nevadensis R	0.4138	0.7914	0.3775	0.64	0.36	-2955.0	-6575.0	2
Zootermopsis nevadensis S	0.9966	1.0055	0.0089	0.77	0.23	-50545.0	-52540.0	2
Cryptotermes secundus R	0.3791	0.7219	0.3427	0.72	0.28	-11369.0	-16854.0	2
Cryptotermes secundus S	0.9682	1.0381	0.0699	0.67	0.33	-49410.0	-49356.0	1
Blattela germanica R	0.4925	0.7884	0.2959	0.58	0.42	3962.0	3022.0	2
Blattela germanica S	0.9944	1.0117	0.0174	0.77	0.23	-12275.0	-15669.0	2
Orchesella cincta R	0.7126	0.7712	0.0587	0.83	0.17	-20668.0	-21698.0	2
Orchesella cincta S	0.9542	1.0445	0.0903	0.58	0.42	-23376.0	-23327.0	1
Folsomia candida R	0.8272	1.0294	0.2022	0.64	0.36	-19957.0	-20121.0	2
Folsomia candida S	0.9618	1.0457	0.0839	0.64	0.36	-40694.0	-40666.0	1
Daphnia pulex R	0.7112	1.032	0.3208	0.27	0.73	1991.0	807.0	2
Daphnia pulex S	0.9953	1.0087	0.0134	0.76	0.24	-29880.0	-32634.0	2
Daphnia magna R	0.7586	1.0347	0.2761	0.27	0.73	6281.0	6319.0	1
Daphnia magna S	0.9962	1.0012	0.0051	0.73	0.27	-16914.0	-20384.0	2
Tigriopus californicus R	0.7744	0.9042	0.1298	0.49	0.51	-15922.0	-15810.0	1
Tigriopus californicus S	0.9671	1.0354	0.0683	0.64	0.36	-25960.0	-25912.0	1

Lepeophtheirus salmonis R	0.647	0.6939	0.0469	0.82	0.18	-8925.0	-9673.0	2
Lepeophtheirus salmonis S	0.9947	1.0014	0.0066	0.8	0.2	-9992.0	-11473.0	2
Eurytemora affinis R	0.439	0.5797	0.1407	0.62	0.38	-17007.0	-17373.0	2
Eurytemora affinis S	0.9578	1.038	0.0802	0.52	0.48	-38120.0	-38027.0	1
Calanus finmarchicus	n/a							
Hyalella azteca R	0.7	0.8795	0.1794	0.18	0.82	-8931.0	-11284.0	2
Hyalella azteca S	0.9958	1.0071	0.0112	0.76	0.24	-43920.0	-45245.0	2
Armadillidium vulgare R	0.6067	0.8187	0.2121	0.87	0.13	3276.0	1518.0	2
Armadillidium vulgare S	0.9897	1.0193	0.0297	0.78	0.22	-6608.0	-8110.0	2
Penaeus vannamei R	0.4245	0.8915	0.467	0.47	0.53	8693.0	3754.0	2
Penaeus vannamei S	0.9975	1.0064	0.0089	0.78	0.22	-55465.0	-58379.0	2
Glomeris marginata	n/a							
Eudigraphis taiwaniensis	n/a							
Strigamia maritima R	0.8691	1.2129	0.3439	0.46	0.54	8221.0	8341.0	1
Strigamia maritima S	0.9947	1.0081	0.0134	0.79	0.21	-14336.0	-16130.0	2
Leptotrombidium deliense R	1.0744	1.291	0.2167	0.73	0.27	1760.0	1786.0	1
Leptotrombidium deliense S	0.9945	1.0087	0.0142	0.78	0.22	-9178.0	-10309.0	2
Dinothrombium tinctorium R	1.1702	1.4561	0.2859	0.67	0.33	3690.0	3579.0	2
Dinothrombium tinctorium S	0.9946	1.0061	0.0115	0.78	0.22	-18044.0	-19411.0	2
Tetranychus urticae R	0.6677	0.7239	0.0562	0.81	0.19	-16406.0	-16959.0	2
Tetranychus urticae S	0.9527	1.0526	0.0998	0.61	0.39	-18343.0	-18287.0	1
Euroglyphus maynei R	1.1494	1.3728	0.2234	0.65	0.35	5291.0	5267.0	2
Euroglyphus maynei S	0.9812	1.022	0.0409	0.79	0.21	-2143.0	-2939.0	2
Dermatophagoides pteronyssinus R	1.1084	1.2321	0.1236	0.83	0.17	1621.0	1165.0	2
Dermatophagoides pteronyssinus S	0.9875	1.0094	0.0219	0.78	0.22	-10255.0	-11128.0	2
Sarcoptes scabiei R	1.1094	1.3187	0.2093	0.74	0.26	2131.0	2127.0	2
Sarcoptes scabiei S	0.9891	1.0155	0.0263	0.78	0.22	-6067.0	-7014.0	2
Varroa destructor R	1.0253	1.1135	0.0882	0.45	0.55	-42435.0	-42330.0	1
Varroa destructor S	0.9707	1.0331	0.0625	0.63	0.37	-59163.0	-59103.0	1
Varroa jacobsoni R	1.0567	1.0785	0.0218	0.21	0.79	-32590.0	-34157.0	2
Varroa jacobsoni S	0.9636	1.0349	0.0713	0.5	0.5	-47544.0	-47425.0	1
Tropilaelaps mercedesae R	1.0649	1.1108	0.0459	0.14	0.86	-8487.0	-10582.0	2
Tropilaelaps mercedesae S	0.9958	1.0057	0.01	0.8	0.2	-18343.0	-20270.0	2
Galendromus occidentalis R	1.104	1.1641	0.0601	0.2	0.8	-17301.0	-17762.0	2
Galendromus occidentalis S	0.9975	1.0021	0.0045	0.75	0.25	-22238.0	-22942.0	2
Ixodes scapularis R	0.7496	0.9484	0.1988	0.36	0.64	-23418.0	-23527.0	2
Ixodes scapularis S	0.969	1.03	0.061	0.48	0.52	-66048.0	-65882.0	1
Trichonephila clavipes R	0.4915	0.974	0.4824	0.41	0.59	9948.0	8235.0	2

Trichonephila clavipes S	0.9965	1.0091	0.0126	0.78	0.22	-15201.0	-17566.0	2
Parasteatoda tepidariorum R	0.4274	0.7049	0.2775	0.51	0.49	-10353.0	-10914.0	2
Parasteatoda tepidariorum S	0.9923	1.0128	0.0205	0.79	0.21	-29863.0	-31499.0	2
Stegodyphus mimosarum R	0.3925	0.8096	0.4171	0.49	0.51	8317.0	6503.0	2
Stegodyphus mimosarum S	0.9913	1.0169	0.0256	0.79	0.21	-7466.0	-10807.0	2
Centruroides sculpturatus R	0.4519	0.994	0.5421	0.39	0.61	25356.0	23532.0	2
Centruroides sculpturatus S	0.9934	1.0079	0.0146	0.77	0.23	-30967.0	-33142.0	2
Limulus polyphemus R	0.3044	0.7184	0.414	0.59	0.41	1075.0	-7233.0	2
Limulus polyphemus S	0.9548	1.0455	0.0907	0.54	0.46	-50009.0	-49853.0	1
Anoplodactylus insignis	n/a							
Hypsibius dujardini real	1.0558	1.0677	0.0118	0.82	0.18	-21732.0	-24760.0	2
Hypsibius dujardini S	0.9985	1.0051	0.0066	0.75	0.25	-34845.0	-36446.0	2
Ramazzottius varieornatus real	0.9257	1.0307	0.105	0.34	0.66	-23199.0	-23082.0	1
Ramazzottius varieornatus S	0.9962	1.007	0.0108	0.79	0.21	-29507.0	-31248.0	2
Peripatopsis overbergiensis	n/a							
Peripatoides sp	n/a							
Peripatus sp	n/a							
Caenorhabditis briggsae R	0.853	1.0433	0.1903	0.64	0.36	-8025.0	-7995.0	1
Caenorhabditis briggsae S	0.961	1.0458	0.0848	0.65	0.35	-28067.0	-28012.0	1
Caenorhabditis remanei R	0.8693	1.098	0.2287	0.76	0.24	-3731.0	-4304.0	2
Caenorhabditis remanei S	0.994	1.0127	0.0186	0.8	0.2	-23788.0	-25979.0	2
Caenorhabditis brenneri R	0.7786	0.9973	0.2187	0.62	0.38	-9050.0	-9063.0	2
Caenorhabditis brenneri S	0.9947	1.0067	0.012	0.76	0.24	-33447.0	-35460.0	2
Caenorhabditis elegans R	0.841	1.0388	0.1978	0.66	0.34	-8377.0	-8417.0	2
Caenorhabditis elegans S	0.9972	1.0084	0.0112	0.79	0.21	-31991.0	-34446.0	2
Caenorhabditis japonica R	1.0072	1.2004	0.1932	0.46	0.54	-5894.0	-5665.0	1
Caenorhabditis japonica S	0.9951	1.0087	0.0136	0.76	0.24	-34642.0	-36824.0	2
Caenorhabditis angaria R	0.8582	1.2171	0.3588	0.4	0.6	10018.0	9891.0	2
Caenorhabditis angaria S	0.9921	1.0155	0.0233	0.79	0.21	-21346.0	-23985.0	2
Diploscapter pachys R	0.9622	1.1743	0.2121	0.68	0.32	-21290.0	-21258.0	1
Diploscapter pachys S	0.9665	1.037	0.0705	0.64	0.36	-62993.0	-62967.0	1
Diploscapter coronatus R	1.0207	1.0501	0.0294	0.78	0.22	-14668.0	-16250.0	2
Diploscapter coronatus S	0.9972	1.0037	0.0065	0.78	0.22	-38387.0	-42957.0	2
Angiostrongylus cantonensis R	1.0189	1.0912	0.0723	0.81	0.19	-5486.0	-5955.0	2
Angiostrongylus cantonensis S	0.9505	1.0532	0.1027	0.65	0.35	-12882.0	-12904.0	1
Dictyocaulus viviparus R	0.9707	1.1471	0.1765	0.82	0.18	-2027.0	-2699.0	2
Dictyocaulus viviparus S	0.9952	1.0078	0.0126	0.77	0.23	-10861.0	-11662.0	2
Haemonchus contortus R	0.9522	0.9951	0.043	0.21	0.79	-14409.0	-15539.0	2

Haemonchus contortus S	0.959	1.047	0.088	0.66	0.34	-27223.0	-27170.0	1
Nippostrongylus brasiliensis R	1.0322	1.1763	0.1441	0.72	0.28	-12473.0	-12565.0	2
Nippostrongylus brasiliensis S	0.9509	1.0505	0.0996	0.58	0.42	-25009.0	-24935.0	1
Ancylostoma ceylanicum R	0.947	1.0193	0.0723	0.82	0.18	-33736.0	-36398.0	2
Ancylostoma ceylanicum S	0.9519	1.0538	0.1018	0.64	0.36	-65364.0	-65323.0	1
Necator americanus R	0.9313	1.1439	0.2126	0.76	0.24	-6216.0	-6440.0	2
Necator americanus S	0.9906	1.013	0.0224	0.77	0.23	-16090.0	-17125.0	2
Pristionchus pacificus R	0.9174	1.0988	0.1814	0.23	0.77	-11352.0	-11316.0	1
Pristionchus pacificus S	0.998	1.0008	0.0028	0.75	0.25	-33158.0	-35969.0	1
Loa loa R	0.9893	4.5344	3.5451	0.99	0.01	35035.0	-2779.0	2
Loa loa S	0.9985	1.1798	0.1813	0.98	0.02	-2531.0	-9150.0	2
Brugia malayi R	0.9528	0.9829	0.0301	0.81	0.19	-4975.0	-6683.0	2
Brugia malayi S	0.9969	1.0052	0.0083	0.8	0.2	-11405.0	-13214.0	2
Litomosoides sigmodontis R	1.0101	1.0816	0.0714	0.83	0.17	-5561.0	-6136.0	2
Litomosoides sigmodontis S	0.994	1.0067	0.0127	0.75	0.25	-10487.0	-11226.0	2
Onchocerca volvulus R	1.016	1.0724	0.0564	0.82	0.18	-1194.0	-2392.0	2
Onchocerca volvulus S	0.9943	1.023	0.0287	0.82	0.18	-6628.0	-8256.0	2
Dirofilaria immitis R	1.0267	1.1377	0.111	0.86	0.14	1925.0	157.0	2
Dirofilaria immitis S	0.9928	1.0109	0.018	0.8	0.2	-4549.0	-6411.0	2
Thelazia callipaeda R	0.8854	0.9424	0.0569	0.82	0.18	-4100.0	-4996.0	2
Thelazia callipaeda S	0.9913	1.019	0.0276	0.79	0.21	-8304.0	-9119.0	2
Dracunculus medinensis R	1.0509	1.1842	0.1333	0.84	0.16	977.0	85.0	2
Dracunculus medinensis S	0.9894	1.0151	0.0257	0.77	0.23	-6359.0	-7026.0	2
Toxocara canis R	1.1608	1.1794	0.0186	0.79	0.21	-7772.0	-10082.0	2
Toxocara canis S	0.9525	1.0537	0.1011	0.66	0.34	-21090.0	-21057.0	1
Ascaris suum R	1.1592	1.16	0.0008	0.96	0.04	2394.0	-24231.0	2
Ascaris suum S	0.9968	1.0043	0.0075	0.77	0.23	-56583.0	-63687.0	2
Syphacia muris R	0.9503	1.0709	0.1206	0.84	0.16	-1252.0	-2296.0	2
Syphacia muris S	0.988	1.0174	0.0294	0.79	0.21	-8619.0	-9454.0	2
Globodera pallida R	1.0844	1.1078	0.0234	0.22	0.78	-12815.0	-13614.0	2
Globodera pallida S	0.9572	1.0407	0.0836	0.49	0.51	-21181.0	-21106.0	1
Meloidogyne hapla R	0.6016	0.7938	0.1921	0.75	0.25	237.0	-204.0	2
Meloidogyne hapla S	0.9933	1.0156	0.0223	0.79	0.21	-2386.0	-3946.0	2
Bursaphelenchus xylophilus R	0.8642	1.0546	0.1904	0.56	0.44	-10736.0	-10622.0	1
Bursaphelenchus xylophilus S	0.9946	1.0045	0.01	0.77	0.23	-21639.0	-22788.0	2
Panagrellus redivivus R	1.0351	1.1462	0.111	0.37	0.63	-23461.0	-23373.0	1
Panagrellus redivivus S	0.964	1.0435	0.0795	0.71	0.29	-33170.0	-33165.0	1
Strongyloides ratti R	0.3352	0.588	0.2528	0.75	0.25	-3767.0	-5175.0	2

Strongyloides ratti S	0.9865	1.0168	0.0303	0.77	0.23	-2003.0	-2804.0	2
Rhabditophanes sp. R	0.6383	0.7723	0.134	0.61	0.39	-10520.0	-10470.0	1
Rhabditophanes sp. S	0.989	1.0183	0.0293	0.8	0.2	-10875.0	-11675.0	2
Plectus sambesii R	1.0937	1.2408	0.147	0.13	0.87	-15972.0	-21776.0	2
Plectus sambesii S	0.9964	1.0057	0.0093	0.76	0.24	-59200.0	-61656.0	2
Trichuris trichiura R	0.9837	1.1873	0.2037	0.4	0.6	-5005.0	-4944.0	1
Trichuris trichiura S	0.9953	1.0068	0.0115	0.77	0.23	-13328.0	-13945.0	2
Trichuris suis R	0.9438	1.1327	0.1889	0.33	0.67	-5474.0	-5468.0	1
Trichuris suis S	0.9943	1.0127	0.0184	0.8	0.2	-11671.0	-12614.0	2
Trichuris muris R	1.0193	1.1009	0.0816	0.15	0.85	-8152.0	-9779.0	2
Trichuris muris S	0.9637	1.0406	0.0769	0.67	0.33	-21171.0	-21165.0	1
Trichinella nelsoni R	0.9116	1.0537	0.1422	0.18	0.82	-666.0	-1451.0	2
Trichinella nelsoni S	0.9965	1.0082	0.0116	0.8	0.2	-12158.0	-15200.0	2
Trichinella spiralis R	0.8703	1.113	0.2427	0.36	0.64	-1039.0	-970.0	1
Trichinella spiralis S	0.9942	1.0059	0.0117	0.74	0.26	-11895.0	-13102.0	2
Trichinella britovi R	0.8932	1.0855	0.1924	0.26	0.74	1518.0	1648.0	1
Trichinella britovi S	0.9946	1.0103	0.0157	0.78	0.22	-11779.0	-15789.0	2
Soboliphyme baturini R	1.0866	1.2181	0.1315	0.54	0.46	-8034.0	-7965.0	1
Soboliphyme baturini S	0.9958	1.0051	0.0093	0.76	0.24	-14162.0	-14986.0	2
Romanomermis culicivorax R	0.8998	1.4784	0.5786	0.33	0.67	55447.0	55450.0	1
Romanomermis culicivorax S	0.9346	1.0717	0.1371	0.65	0.35	-19332.0	-19418.0	2
Priapulus caudatus R	0.6974	1.1195	0.4221	0.46	0.54	6976.0	6512.0	2
Priapulus caudatus S	0.9687	1.0295	0.0608	0.53	0.47	-35826.0	-35768.0	1
Nematostella vectensis R	0.5104	0.9095	0.3991	0.4	0.6	2073.0	-32.0	2
Nematostella vectensis S	0.9651	1.0393	0.0742	0.66	0.34	-54573.0	-54550.0	2
Hydra vulgaris R	0.472	0.8406	0.3686	0.69	0.31	4449.0	2100.0	2
Hydra vulgaris S	0.9835	1.0231	0.0396	0.79	0.21	-13764.0	-14857.0	2
Trichoplax adhaerens R	0.8998	0.9123	0.0125	0.23	0.77	-4917.0	-5894.0	2
Trichoplax adhaerens S	0.9848	1.0223	0.0376	0.79	0.21	-10882.0	-11366.0	2
Trichoplax H2 R	0.901	0.9493	0.0483	0.8	0.2	-7923.0	-8665.0	2
Trichoplax H2 S	0.9919	1.0125	0.0206	0.79	0.21	-12258.0	-13212.0	2
Amphimedon queenslandica R	0.3458	0.7611	0.4153	0.95	0.05	-6072.0	-14014.0	2
Amphimedon queenslandica S	0.9925	1.0134	0.0209	0.79	0.21	-27515.0	-28959.0	2

Table 2: Parameters of the GMM modelling for each species real and shuffled data. The letter after the species name stands for real ('R') and shuffled ('S') data. The parameters are: meanLow - the mean of the component with lower mean O/E CpG, meanHigh - the mean of the component with higher mean O/E CpG, meanDist - the distance of the two components, %low - the relative amount of data points in the component with lower mean O/E CpG, %high - the relative amount of data points in the component with higher mean O/E CpG, AIC-N1 - the AIC for a GMM with 1 component, AIC-N2 - the AIC for a GMM with 2 components, best-N - the amount of components which results in a lower AIC

	mLow	mHigh	Dist	%low	%high	mean	SD	skew	mLow-m	mHigh-m
Drosophila melanogaster	0.88	0.9	0.01	0.75	0.25	0.89	0.12	0.21	0.01	0.01
Aedes aegypti	0.99	1.05	0.06	0.17	0.83	1.03	0.12	-0.56	0.04	0.02
Anopheles gambiae	1.07	1.16	0.08	0.15	0.85	1.13	0.13	-0.61	0.06	0.03
Ctenocephalides felis	0.58	0.91	0.33	0.59	0.41	0.73	0.24	0.46	0.15	0.18
Bombyx mori	0.68	1.06	0.39	0.17	0.83	0.98	0.23	-0.77	0.3	0.08
Danaus plexippus	0.83	0.94	0.11	0.19	0.81	0.9	0.23	-0.04	0.07	0.04
Operophtera brumata	0.75	1.01	0.26	0.37	0.63	0.9	0.22	-0.2	0.15	0.11
Heliconius melpomene	0.63	0.99	0.36	0.27	0.73	0.87	0.26	-0.32	0.24	0.12
Melitaea cinxia	0.8	1.07	0.27	0.36	0.64	0.96	0.26	0.04	0.16	0.11
Papilio xuthus	0.64	1.02	0.39	0.26	0.74	0.91	0.24	-0.64	0.27	0.11
Plutella xylostella	0.69	0.98	0.28	0.18	0.82	0.9	0.2	-0.8	0.21	0.08
Limnephilus lunatus	0.71	0.98	0.27	0.78	0.22	0.8	0.21	0.5	0.09	0.18
Agrilus planipennis	0.59	0.96	0.37	0.32	0.68	0.82	0.26	-0.21	0.23	0.14
Nicrophorus vespilloides	0.67	1.03	0.37	0.15	0.85	0.96	0.24	-0.94	0.29	0.07
Onthophagus taurus	1.0	1.31	0.31	0.52	0.48	1.15	0.28	0.05	0.15	0.16
Oryctes borbonicus	0.89	1.14	0.25	0.48	0.52	1.02	0.22	-0.01	0.13	0.12
Anoplophora glabripennis	0.69	0.91	0.21	0.38	0.62	0.82	0.2	0.02	0.13	0.09
Leptinotarsa decemlineata	0.58	0.84	0.26	0.5	0.5	0.71	0.2	0.08	0.13	0.13
Diabrotica virgifera	0.59	0.82	0.23	0.35	0.65	0.72	0.22	0.17	0.13	0.1
Dendroctonus ponderosae	0.76	0.89	0.13	0.36	0.64	0.83	0.14	-0.15	0.07	0.06
Aethina tumida	0.63	0.98	0.35	0.26	0.74	0.86	0.26	-0.42	0.23	0.12
Tribolium castaneum	0.95	1.04	0.08	0.18	0.82	1.01	0.17	-0.27	0.06	0.03
Apis mellifera	0.54	1.13	0.58	0.39	0.61	0.89	0.35	-0.13	0.35	0.24
Bombus impatiens	0.62	1.11	0.5	0.41	0.59	0.91	0.31	-0.11	0.29	0.2
Atta cephalotes	0.86	1.13	0.27	0.29	0.71	1.03	0.22	-0.21	0.17	0.1
Acromyrmex echinatior	0.89	1.13	0.24	0.24	0.76	1.04	0.21	-0.21	0.15	0.09
Harpegnathos saltator	0.72	1.16	0.44	0.2	0.8	1.05	0.25	-0.8	0.33	0.11
Solenopsis invicta	0.91	1.16	0.25	0.2	0.8	1.08	0.22	-0.32	0.17	0.08
Polistes dominula	0.77	1.08	0.3	0.46	0.54	0.94	0.25	0.23	0.17	0.14
Polistes canadensis	0.8	1.13	0.34	0.47	0.53	0.98	0.26	0.17	0.18	0.15
Nasonia vitripennis	0.63	1.07	0.43	0.29	0.71	0.93	0.26	-0.51	0.3	0.14
Cephus cinctu	0.75	0.98	0.22	0.32	0.68	0.89	0.2	-0.1	0.14	0.09
Orussus abietinus	0.84	1.01	0.17	0.32	0.68	0.94	0.17	-0.11	0.1	0.07
Athalia rosae	0.77	1.19	0.41	0.55	0.45	0.96	0.26	0.25	0.19	0.23
Pediculus humanus	0.81	1.44	0.63	0.46	0.54	1.15	0.46	0.29	0.34	0.29
Nilaparvata lugens	0.63	0.83	0.2	0.26	0.74	0.75	0.21	-0.18	0.12	0.08

Laodelphax striatellus	0.69	0.89	0.2	0.42	0.58	0.8	0.22	0.09	0.11	0.09
Rhodnius prolixus	0.67	0.81	0.14	0.84	0.16	0.71	0.21	0.78	0.04	0.1
Cimex lectularius	0.67	0.97	0.3	0.59	0.41	0.8	0.21	0.22	0.13	0.17
Halyomorpha halys	0.42	0.69	0.27	0.59	0.41	0.53	0.19	0.49	0.11	0.16
Bemisia tabaci	0.63	0.96	0.33	0.53	0.47	0.78	0.21	0.2	0.15	0.18
Aphis gossypii	0.57	1.1	0.53	0.56	0.44	0.81	0.32	0.38	0.24	0.29
Rhopalosiphum maidis	0.59	1.13	0.54	0.55	0.45	0.83	0.32	0.29	0.24	0.3
Acyrthosiphon pisum	0.62	1.11	0.49	0.55	0.45	0.84	0.3	0.26	0.22	0.27
Diuraphis noxia	0.57	1.09	0.52	0.57	0.43	0.79	0.31	0.35	0.22	0.3
Myzus persicae	0.59	1.09	0.49	0.56	0.44	0.82	0.29	0.27	0.23	0.27
Diaphorina citri	0.5	0.78	0.28	0.37	0.63	0.66	0.25	0.4	0.16	0.12
Frankliniella occidentalis	0.44	0.95	0.51	0.51	0.49	0.68	0.28	0.1	0.24	0.27
Zootermopsis nevadensis	0.41	0.79	0.38	0.64	0.36	0.56	0.23	0.48	0.15	0.23
Cryptotermes secundus	0.38	0.72	0.34	0.72	0.28	0.48	0.2	0.82	0.1	0.24
Blattela germanica	0.49	0.79	0.3	0.58	0.42	0.63	0.26	0.8	0.14	0.16
Orchesella cincta	0.71	0.77	0.06	0.83	0.17	0.73	0.15	0.42	0.02	0.04
Folsomia candida	0.83	1.03	0.2	0.64	0.36	0.91	0.16	0.28	0.08	0.12
Daphnia pulex	0.71	1.03	0.32	0.27	0.73	0.92	0.25	-0.42	0.21	0.11
Daphnia magna	0.77	1.04	0.27	0.31	0.69	0.93	0.27	-0.06	0.16	0.11
Tigriopus californicus	0.77	0.9	0.13	0.49	0.51	0.84	0.15	-0.03	0.07	0.06
Lepeophtheirus salmonis	0.65	0.69	0.05	0.82	0.18	0.66	0.17	0.41	0.01	0.03
Eurytemora affinis	0.44	0.58	0.14	0.62	0.38	0.51	0.18	0.68	0.07	0.07
Calanus finmarchicus	n/a									
Hyalella azteca	0.7	0.88	0.18	0.18	0.82	0.82	0.2	-0.45	0.12	0.06
Armadillidium vulgare	0.61	0.82	0.21	0.87	0.13	0.67	0.26	0.92	0.06	0.15
Penaeus vannamei	0.42	0.89	0.47	0.47	0.53	0.67	0.28	0.06	0.25	0.22
Glomeris marginata	n/a									
Eudigraphis taiwaniensis	n/a									
Strigamia maritima	0.86	1.21	0.35	0.43	0.57	1.05	0.32	0.05	0.19	0.16
Leptotrombidium deliense	1.07	1.29	0.22	0.74	0.26	1.16	0.26	0.07	0.09	0.13
Dinothrombium tinctorium	1.17	1.45	0.28	0.66	0.34	1.29	0.27	0.28	0.12	0.16
Tetranychus urticae	0.67	0.72	0.06	0.82	0.18	0.69	0.14	0.44	0.02	0.03
Euroglyphus maynei	1.15	1.38	0.22	0.69	0.31	1.25	0.29	0.21	0.1	0.13
Dermatophagoides pteronyssinus	1.11	1.23	0.12	0.83	0.17	1.15	0.26	0.38	0.04	0.08
Sarcoptes scabiei	1.11	1.32	0.21	0.75	0.25	1.2	0.27	0.14	0.09	0.12
Varroa destructor	1.02	1.11	0.09	0.44	0.56	1.07	0.12	-0.12	0.05	0.04
Varroa jacobsoni	1.06	1.08	0.02	0.21	0.79	1.07	0.13	-0.03	0.01	0.01
Tropilaelaps mercedesae	1.06	1.11	0.05	0.14	0.86	1.1	0.18	-0.18	0.04	0.01

Galendromus occidentalis	1.1	1.16	0.06	0.2	0.8	1.14	0.12	-0.41	0.04	0.02
Ixodes scapularis	0.75	0.95	0.2	0.36	0.64	0.87	0.17	-0.22	0.12	0.08
Trichonephila clavipes	0.49	0.97	0.48	0.41	0.59	0.77	0.3	-0.14	0.28	0.2
Parasteatoda tepidariorum	0.43	0.71	0.28	0.52	0.48	0.57	0.2	0.34	0.14	0.14
Stegodyphus mimosarum	0.39	0.81	0.42	0.49	0.51	0.61	0.28	0.54	0.22	0.2
Centruroides sculpturatus	0.45	0.99	0.54	0.4	0.6	0.78	0.35	0.1	0.33	0.21
Limulus polyphemus	0.3	0.72	0.41	0.59	0.41	0.48	0.25	0.57	0.18	0.24
Anoplodactylus insignis	n/a									
Hypsibius dujardini	1.06	1.07	0.01	0.82	0.18	1.06	0.14	0.55	0.0	0.01
Ramazzottius varieornatus	0.93	1.03	0.1	0.36	0.64	0.98	0.15	0.09	0.05	0.05
Peripatopsis overbergiensis	n/a									
Peripatoides sp	n/a									
Peripatus sp	n/a									
Caenorhabditis briggsae	0.85	1.04	0.19	0.63	0.37	0.94	0.2	0.18	0.09	0.1
Caenorhabditis remanei	0.87	1.1	0.23	0.76	0.24	0.95	0.22	0.38	0.08	0.15
Caenorhabditis brenneri	0.78	1.0	0.22	0.62	0.38	0.88	0.21	0.26	0.1	0.12
Caenorhabditis elegans	0.84	1.04	0.2	0.68	0.32	0.93	0.21	0.21	0.09	0.11
Caenorhabditis japonica	1.01	1.2	0.19	0.46	0.54	1.11	0.22	0.07	0.1	0.09
Caenorhabditis angaria	0.86	1.22	0.36	0.39	0.61	1.06	0.28	-0.18	0.2	0.16
Diploscapter pachys	0.96	1.17	0.21	0.67	0.33	1.05	0.18	0.15	0.09	0.12
Diploscapter coronatus	1.02	1.05	0.03	0.78	0.22	1.03	0.19	0.23	0.01	0.02
Angiostrongylus cantonensis	1.02	1.09	0.07	0.81	0.19	1.05	0.2	0.31	0.03	0.04
Dictyocaulus viviparus	0.97	1.15	0.18	0.82	0.18	1.03	0.22	0.54	0.06	0.12
Haemonchus contortus	0.95	1.0	0.04	0.21	0.79	0.98	0.17	-0.1	0.03	0.02
Nippostrongylus brasiliensis	1.03	1.17	0.14	0.71	0.29	1.09	0.18	0.23	0.06	0.08
Ancylostoma ceylanicum	0.95	1.02	0.07	0.82	0.18	0.97	0.19	0.32	0.02	0.05
Necator americanus	0.93	1.14	0.21	0.76	0.24	1.01	0.21	0.27	0.08	0.13
Pristionchus pacificus	0.92	1.1	0.18	0.23	0.77	1.03	0.19	-0.01	0.11	0.07
Loa loa	0.99	4.53	3.55	0.99	0.01	1.02	0.99	49.25	0.03	3.51
Brugia malayi	0.95	0.98	0.03	0.81	0.19	0.96	0.2	0.39	0.01	0.02
Litomosoides sigmodontis	1.01	1.08	0.07	0.83	0.17	1.04	0.18	0.28	0.03	0.04
Onchocerca volvulus	1.02	1.07	0.06	0.82	0.18	1.03	0.23	0.47	0.01	0.04
Dirofilaria immitis	1.03	1.13	0.11	0.85	0.15	1.06	0.26	0.8	0.03	0.07
Thelazia callipaeda	0.89	0.94	0.06	0.81	0.19	0.91	0.2	0.6	0.02	0.03
Dracunculus medinensis	1.05	1.19	0.13	0.84	0.16	1.09	0.25	0.62	0.04	0.1
Toxocara canis	1.16	1.18	0.02	0.81	0.19	1.17	0.2	0.89	0.01	0.01
Ascaris suum	1.16	1.33	0.17	0.99	0.01	1.16	0.25	18.11	0.0	0.17
Syphacia muris	0.95	1.07	0.12	0.84	0.16	0.99	0.23	1.07	0.04	0.08

Globodera pallida	1.08	1.11	0.02	0.24	0.76	1.1	0.16	-0.16	0.02	0.01
Meloidogyne hapla	0.6	0.79	0.19	0.75	0.25	0.68	0.24	0.7	0.08	0.11
Bursaphelenchus xylophilus	0.86	1.05	0.19	0.55	0.45	0.95	0.18	0.15	0.09	0.1
Panagrellus redivivus	1.03	1.15	0.11	0.34	0.66	1.09	0.15	-0.06	0.06	0.06
Strongyloides ratti	0.34	0.59	0.25	0.76	0.24	0.42	0.21	1.05	0.08	0.17
Rhabditophanes sp.	0.64	0.77	0.13	0.58	0.42	0.7	0.16	0.13	0.06	0.07
Plectus sambesii	1.09	1.24	0.15	0.13	0.87	1.2	0.2	-0.96	0.11	0.04
Trichuris trichiura	0.98	1.19	0.2	0.39	0.61	1.1	0.19	-0.08	0.12	0.09
Trichuris suis	0.94	1.13	0.19	0.33	0.67	1.05	0.18	-0.18	0.11	0.08
Trichuris muris	1.02	1.1	0.08	0.15	0.85	1.07	0.18	-0.91	0.05	0.03
Trichinella nelsoni	0.91	1.05	0.14	0.18	0.82	1.0	0.24	-0.28	0.09	0.05
Trichinella spiralis	0.87	1.11	0.24	0.37	0.63	1.01	0.23	-0.08	0.14	0.1
Trichinella britovi	0.89	1.08	0.19	0.24	0.76	1.01	0.25	0.24	0.12	0.07
Soboliphyme baturini	1.09	1.22	0.13	0.54	0.46	1.15	0.18	0.07	0.06	0.07
Romanomermis culicivorax	0.9	1.48	0.58	0.33	0.67	1.26	0.43	-0.04	0.36	0.22
Priapulus caudatus	0.7	1.12	0.42	0.46	0.54	0.92	0.29	0.02	0.22	0.2
Nematostella vectensis	0.51	0.91	0.4	0.4	0.6	0.75	0.25	-0.15	0.24	0.16
Hydra vulgaris	0.47	0.84	0.37	0.69	0.31	0.61	0.27	1.09	0.14	0.23
Trichoplax adhaerens	0.9	0.91	0.01	0.25	0.75	0.91	0.2	-0.13	0.01	0.0
Trichoplax H2	0.9	0.95	0.05	0.81	0.19	0.92	0.17	0.33	0.02	0.03
Amphimedon queenslandica	0.35	0.77	0.43	0.96	0.04	0.38	0.21	1.84	0.03	0.39

Table 3: Additional parameters of the GMM modelling but only the real data. The first 5 columns are identical to SupTable2, rounded to two positions behind the decimal point. In addition it contains mean - the mean of the whole O/E CpG distributions, SD - the (sample) standard deviation of the whole distribution, skew - the skewness of the whole distribution, mLow-m - distance between meanLow and mean, mHigh-m distance between meanHigh and mean

Species	Engelhardt et al.			Rošić et al. (2018)		Exp. data
	DNMT1	DNMT3	Methyl.	DNMT1	DNMT3	
Nematoda						
R. culicivorax	X	X	X	X	X	X (Rošić et al., 2018)
**T. spiralis**	O	X	**O**	O	X	X (Rošić et al., 2018)
**T. muris**	O	X	**O**	O	X	X (Rošić et al., 2018)
**P. sambesii**	X	O	**O**	X	O	X (Rošić et al., 2018)
P. redivivus	O	O	O	O	O	n/a
B. xylophilus	O	O	O	O	O	n/a
M. hapla	O	O	O	O	O	n/a
G. pallida	O	O	O	O	O	n/a
A. suum	O	O	O	O	O	n/a
D. immitis	O	O	O	O	O	n/a
O. volvulus	O	O	O	O	O	n/a
B. malayi	O	O	O	O	O	n/a
N. brasiliensis	O	O	O	O	O	O (Rošić et al., 2018)
C. briggsae	O	O	O	O	O	O (Rošić et al., 2018)

Table 4: The table contains all species analyzed in this study which have been analyzed as well in either Bewick et al. (2017), Provataris et al. (2018), Rošić et al. (2018) or if experimental verification of DNA methylation is available. X - indicates presence; O - indicates absence; DNMT1/DNMT3 means the occurence of at least one paralog of the respective enzyme. Methyl. means if the respective study defines the genome as containing DNA methylation or not according to the O/E CpG content (In case of *Provataris et al.* 'X' is 'Unimodal, indicative of methylation' and 'XX' is 'bimodal depleted'). If the species name is bold there is a contradiction in DNMT occurences or methylation status between our data and another study. The column of our study which is contradicting is bold as well.

Species	Engelhardt et al.			Bewick et al. (2017)			Provataris et al. (2018)			Exp. data
	DNMT1	DNMT3	Methyl.	DNMT1	DNMT3	Methyl.	DNMT1	DNMT3	Methyl.	
Arthropoda										
L. polyphemus	X	X	X							X (Lewis et al., 2020)
P. tepidariorum	X	X	X							X (Lewis et al., 2020)
**Ixodes scapularis**	X	**X**	**O**				X	O	O	X (Lewis et al., 2020)
**Strigamia maritima**	X	X	**X**				X	X	O	X (de Mendoza et al., 2019b
**P. vannamei**	**X**	**X**	X				O	O	XX	n/a
**A. vulgare**	O	O	**O**							X (Lewis et al., 2020)
L. salmonis	X	O	O				X	O	O	n/a
D. pulex	X	X	X	X	X	X	X	X	X	X (Kvist et al., 2018)
D. magna	X	X	X							X (Kvist et al., 2018)
F. candida	O	O	O				O	O	O	n/a
O. cincta	O	O	O				O	O	O	n/a
**Z. nevadensis**	**X**	X	X	O	X	X	X	X	XX	n/a
**B. germanica**	X	**X**	X	X	O	X				X (Bewick et al., 2017)
**N. lugens**	X	**X**	O				X	O	O	n/a
**H. halys**	X	**X**	X	X	O	X				n/a
R. prolixus	X	O	**O**	X	O	X	X	O	O	n/a
C. lectularius	X	O	X	X	O	X				n/a
B. tabaci	X	X	X				X	X	XX	n/a
D. citri	X	O	X	X	O	X				n/a
A. pisum	X	X	X	X	X	X	X	X	XX	X (Lewis et al., 2020)
A. gossypii	X	X	X				X	X	XX	n/a
P. humanus	X	O	X	X	O	X	X	O	XX	n/a
A. rosae	X	X	X	X	X	X				n/a
**O. abietinus**	X	X	**O**	X	X	X	X	X	O	n/a
N. vitripennis	X	X	X	X	X	X	X	X	XX	X (Bewick et al., 2017)
P. dominula	X	O	X							X (Standage et al., 2016)
P. canadensis	X	O	X	X	O	X				X (Standage et al., 2016)
A. mellifera	X	X	X	X	X	X	X	X	XX	X (Bewick et al., 2017)
B. impatiens	X	X	X	X	X	X	X	X	XX	n/a
**H. saltator**	X	X	**X**	X	X	X	X	X	O	X (Bonasio et al., 2012)
S. invicta	X	X	X	X	X	X	X	X	X	X (Hunt et al., 2013)
A. echinatior	X	X	O	X	X	X	X	X	O	n/a
**A. cephalotes**	X	X	**X**	X	X	X	X	X	O	n/a
A. planipennis	X	X	X	X	X	X				n/a
N. vespilloides	X	X	X	X	X	X				X (Bewick et al., 2017)
O. taurus	X	X	X	X	X	X				n/a
T. castaneum	X	O	O	X	O	O	X	O	O	O (Bewick et al., 2017)
D. ponderosae	O	O	O	O	O	O	O	O	O	n/a
**A. glabripennis**	X	O	**O**	X	O	X				n/a
L. decemlineata	X	O	X	X	O	X				n/a
C. felis	X	O	X				X	O	O	n/a
A. aegypti	O	O	O	O	O	O	O	O	O	O (Bewick et al., 2017)
A. gambiae	O	O	O	O	O	O	O	O	O	O (Bewick et al., 2017)
D. melanogaster	O	O	O	O	O	O	O	O	O	O (Bewick et al., 2017)
L. lunatus	X	O	X	X	O	X				n/a
**P. xylostella**	X	O	**X**	X	O	X	X	O	O	n/a
B. mori	X	O	X	X	O	X	X	O	X	X (Bewick et al., 2017)
Operophtera brumata	X	O	X	X	O	X				n/a
P. xuthus	X	O	X	X	O	X	X	O	X	n/a
**D. plexippus**	X	O	**O**	X	O	O	X	O	X	n/a
H. melpomene	X	O	X	X	O	X	X	O	XX	X (Lewis et al., 2020)
**M. cinxia**	X	O	**X**	X	O	O	X	O	O	n/a

Table 5: For caption see Tab. 4

Species	Engelhardt et al.			de Mendoza et al. (2019a)		Lewis et al. (2020)		Exp. data
	DNMT1	DNMT3	Methyl.	DNMT1	DNMT3	DNMT1	DNMT3	
A. queenslandica	X	X	X	X	X			X de Mendoza et al. (2019a)
T. adhaerens	O	O	O	O	O			n/a
N. vectensis	X	X	X	X	X			X (Zemach et al., 2010)
T. spiralis	O	X	O	O	X			X (Rošić et al., 2018)
C. elegans	O	O	O	O	O			O (Simpson et al., 1986)
L. polyphemus	X	X	X			X	X	X (Lewis et al., 2020)
P. tepidariorum	X	X	X			X	X	X (Lewis et al., 2020)
**I. scapularis**	X	X	**O**			X	O	X (Lewis et al., 2020)
**T. urticae**	X	X	**O**	X	X			X* (Grbić et al., 2011)
S. maritima	X	X	X	X	X	X	X	X (de Mendoza et al., 2019b)
**A. vulgare**	**O**	O	O			X	O	X (Lewis et al., 2020)
D. pulex	X	X	X	X	X	X	X	X (Kvist et al., 2018)
D. magna	X	X	X	X	X	X	X	X (Kvist et al., 2018)
B. germanica	X	X	X			X	X	X (Lewis et al., 2020)
Z. nevadensis	X	X	X	O	X			n/a
A. pisum	X	X	X	X	X	X	X	X (Lewis et al., 2020)
N. vitripennis	X	X	X			X	X	X (Bewick et al., 2017)
P. canadensis	X	0	X			X	O	X (Standage et al., 2016)
A. mellifera	X	X	X	X	X	X	X	X (Bewick et al., 2017)
H. saltator	X	X	X			X	X	X (Bonasio et al., 2012)
N. vespilloides	X	X	X			X	X	X(Bewick et al., 2017)
T. castaneum	X	O	O	X	O	X	O	O (Bewick et al., 2017)
A. aegypti	O	O	O	O	O	O	O	O (Bewick et al., 2017)
A. gambiae	O	O	O			O	O	O (Bewick et al., 2017)
D. melanogaster	O	O	O	O	O	O	O	O (Bewick et al., 2017)
B. mori	X	O	X	X	O	X	O	X (Bewick et al., 2017)
H. melpomene	X	O	X			X	O	X (Lewis et al., 2020)

Table 6: For caption see Tab. 4.

Species	d-value	Prediction (0.2/0.25/0.3)	Exp. data
A. queenslandica	0.42	X/X/X	X de Mendoza et al. (2019a)
N. vectensis	0.4	X/X/X	X (Zemach et al., 2010)
R. culicivorax	0.58	X/X/X	X (Rošić et al., 2018)
**T. spiralis**	0.24	X/**O**/**O**	X (Rošić et al., 2018)
**T. muris**	0.08	**O**/**O**/**O**	X (Rošić et al., 2018)
**P. sambesii**	0.15	**O**/**O**/**O**	X (Rošić et al., 2018)
N. brasiliensis	0.14	O/O/O	O (Rošić et al., 2018)
C. briggsae	0.19	O/O/O	O (Rošić et al., 2018)
**C. elegans**	0.2	**X**/O/O	O (Simpson et al., 1986)
L. polyphemus	0.41	X/X/X	X (Lewis et al., 2020)
**P. tepidariorum**	0.28	X/X/**O**	X (Lewis et al., 2020)
**I. scapularis**	0.2	X/**O**/**O**	X (Lewis et al., 2020)
**T. urticae**	0.06	**O**/**O**/**O**	X* (Grbić et al., 2011)
S. maritima	0.35	X/X/X	X (de Mendoza et al., 2019b)
**A. vulgare**	0.21	X/**O**/**O**	X (Lewis et al., 2020)
D. pulex	0.32	X/X/X	X (Kvist et al., 2018)
**D. magna**	0.27	X/X/**O**	X (Kvist et al., 2018)
B. germanica	0.3	X/X/X	X (Lewis et al., 2020)
A. pisum	0.49	X/X/X	X (Lewis et al., 2020)
N. vitripennis	0.43	X/X/X	X (Bewick et al., 2017)
P. dominula	0.3	X/X/X	X (Standage et al., 2016)
P. canadensis	0.34	X/X/X	X (Standage et al., 2016)
A. mellifera	0.58	X/X/X	X (Bewick et al., 2017)
H. saltator	0.44	X/X/X	X (Bonasio et al., 2012)
**S. invicta**	0.25	X/X/**O**	X (Hunt et al., 2013)
N. vespilloides	0.37	X/X/X	X(Bewick et al., 2017)
T. castaneum	0.08	O/O/O	O (Bewick et al., 2017)
A. aegypti	0.06	O/O/O	O (Bewick et al., 2017)
A. gambiae	0.08	O/O/O	O (Bewick et al., 2017)
D. melanogaster	0.01	O/O/O	O (Bewick et al., 2017)
B. mori	0.39	X/X/X	X (Bewick et al., 2017)
H. melpomene	0.36	X/X/X	X (Lewis et al., 2020)

Table 7: d value and experimental data. * - the study covered only a very small part of the genome, i.e. 5/330 analyzed CpGs were methylated. 32 species in total

	DNMT1-RFD	zf-CXXC	BAH	PWWP	ADD_DNMT3	DNA_methylase
DNMT1 full	≥ 1	≥ 1	≥ 1	0	0	1
DNMT1 partial	$\geq 1^*$	$\geq 1^*$	$\geq 1^*$	0	0	1
DNMT3 full	0	0	0	≥ 1	≥ 1	1
DNMT3 partial	0	0	0	$\geq 1^*$	$\geq 1^*$	1
DNMT2	0	0	0	0	0	1

Table 8: Classification of DNMT candidates according to the detected domains. If the numbers in multiple columns of one line are marked with an asterisk (*) the condition of only one of the columns has to be fulfilled.

References

Bewick AJ, Vogel KJ, Moore AJ, Schmitz RJ. 2017. Evolution of DNA methylation across insects. Molecular biology and evolution 34:654–665.

Bonasio R, Li Q, Lian J, Mutti NS, Jin L, Zhao H, Zhang P, Wen P, Xiang H, Ding Y, et al.. 2012. Genome-wide and caste-specific DNA methylomes of the ants Camponotus floridanus and Harpegnathos saltator. Current Biology 22:1755–1764.

de Mendoza A, Hatleberg WL, Pang K, Leininger S, Bogdanovic O, Pflueger J, Buckberry S, Technau U, Hejnol A, Adamska M, et al.. 2019a. Convergent evolution of a vertebrate-like methylome in a marine sponge. Nature ecology & evolution 3:1464–1473.

de Mendoza A, Pflueger J, Lister R. 2019b. Capture of a functionally active methyl-CpG binding domain by an arthropod retrotransposon family. Genome research 29:1277–1286.

Grbić M, Van Leeuwen T, Clark RM, Rombauts S, Rouzé P, Grbić V, Osborne EJ, Dermauw W, Ngoc PCT, Ortego F, et al.. 2011. The genome of tetranychus urticae reveals herbivorous pest adaptations. Nature 479:487–492.

Hunt BG, Glastad KM, Yi SV, Goodisman MA. 2013. Patterning and regulatory associations of DNA methylation are mirrored by histone modifications in insects. Genome biology and evolution 5:591–598.

Kvist J, Gonçalves Athan'asio C, Shams Solari O, Brown JB, Colbourne JK, Pfrender ME, Mirbahai L. 2018. Pattern of DNA methylation in Daphnia: evolutionary perspective. Genome biology and evolution 10:1988–2007.

Lewis S, Ross L, Bain S, Pahita E, Smith S, Cordaux R, Miska E, Lenhard B, Jiggins F, Sarkies P. 2020. Widespread conservation and lineage-specific diversification of genome-wide DNA methylation patterns across arthropods. PLoS genetics 16:e1008864.

Provataris P, Meusemann K, Niehuis O, Grath S, Misof B. 2018. Signatures of DNA methylation across insects suggest reduced DNA methylation levels in Holometabola. Genome biology and evolution 10:1185–1197.

Rošić S, Amouroux R, Requena CE, Gomes A, Emperle M, Beltran T, Rane JK, Linnett S, Selkirk ME, Schiffer PH, et al.. 2018. Evolutionary analysis indicates that DNA alkylation damage is a byproduct of cytosine DNA methyltransferase activity. Nature genetics 50:452–459.

Simpson VJ, Johnson TE, Hammen RF. 1986. Caenorhabditis elegans dna does not contain 5-methylcytosine at any time during development or aging. Nucleic acids research 14:6711–6719.

Standage DS, Berens AJ, Glastad KM, Severin AJ, Brendel VP, Toth AL. 2016. Genome, transcriptome and methylome sequencing of a primitively eusocial wasp reveal a greatly reduced DNA methylation system in a social insect. Molecular Ecology 25:1769–1784.

Zemach A, McDaniel IE, Silva P, Zilberman D. 2010. Genome-wide evolutionary analysis of eukaryotic DNA methylation. Science 328:916–919.
